# Nicotine-Inspired, De Novo-Designed SARS-CoV‑2 Main Protease Inhibitors Reveal Unique Chemistry for Covalently Conjugating Both Cysteine and Histidine Residues in the Catalytic Dyad

**DOI:** 10.1021/jacs.6c01119

**Published:** 2026-04-14

**Authors:** Sandeep Atla, Veerabhadra Vulupala, Yugendar R. Alugubelli, Lauren R. Blankenship, Kai Yang, Satyanarayana Nyalata, Kaustav Khatua, Demonta Coleman, Dorsa Rabie, Xuejiao Guo, Chia-Chuan D. Cho, Sathish Kumar, Lai Hoang Son Le, Banumathi Sankaran, Justin K. Kalugin, Shivangi Sharma, Benjamin W. Neuman, Shiqing Xu, Wenshe Ray Liu

**Affiliations:** † Texas A&M Drug Discovery Center and Department of Chemistry, 14736 Texas A&M University, College Station, Texas 77843, United States; ‡ Department of Biology, College of Arts and Sciences, Texas A&M University, College Station, Texas 77843, United States; § Texas A&M Global Health Research Complex, Texas A&M University, College Station, Texas 77843, United States; ∥ Molecular Biophysics and Integrated Bioimaging, Berkeley Center for Structural Biology, 1666 Laurence Berkeley National Laboratory, Berkeley, California 94720, United States; ⊥ Division of Chemical Biology and Medicinal Chemistry, 12330 The University of Texas, Austin, Texas 78712, United States; # Department of Pharmaceutical Sciences, Irma Lerma College of Pharmacy, Texas A&M University, College Station, Texas 77843, United States; ¶ Institute of Biosciences and Technology and Department of Translational Medical Sciences, College of Medicine, Texas A&M University, Houston, Texas 77030, United States; ∇ Department of Biochemistry and Biophysics, College of Agriculture and Life Sciences, Texas A&M University, College Station, Texas 77843, United States; ○ Department of Cell Biology and Genetics, College of Medicine, Texas A&M University, College Station, Texas 77843, United States

## Abstract

Anecdotal reports about smokers with low SARS-CoV-2 infection rates prompted a search for nicotine and its pyrolysis products as SARS-CoV-2 main protease (M^Pro^) inhibitors. From this search, 3-vinylpyridine was discovered as a weak binder for the M^Pro^ S1 subsite and was used subsequently as a de novo starting point for covalent inhibitor design that quickly yielded a highly potent inhibitor, SR-A-174, with an IC_50_ value of 60 nM. Representing a novel class of M^Pro^ inhibitors, SR-A-174 features an *N,N*-diaryl-α,α-dichloroacetamide scaffold that facilitated rapid exploration of alternative covalent warheads and various N-substituents, leading to the identification of multiple inhibitors with potent antiviral activity. Eight such M^Pro^ inhibitor structures were determined, all demonstrating covalent binding to catalytic Cys145 of M^Pro^. In six determined structures, binding is dominated by the covalent bond plus van der Waals contacts, which contrasts with the extensive hydrogen bond networks formed with peptidomimetic inhibitors such as nirmatrelvir. Strikingly, two *N*,*N*-diaryl-α,α-dichloroacetamide inhibitors exhibit an unprecedented dual covalent modification mode of the catalytic dyad, forming bonds to both Cys145 and His41 with a concomitant loss of both chlorides and displacing the inhibitors from the S1 subsite. This dyad-targeting reactivity suggests a novel route for bioconjugation of both cysteine and histidine.

## Graphical abstract

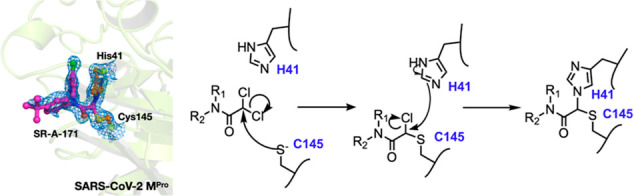

## Introduction

Coronavirus disease 2019 (COVID-19) is an infectious disease caused by severe acute respiratory syndrome coronavirus 2 (SARS-CoV-2).^1^ First emerging in late 2019, the disease quickly spread in 2020, resulting in the COVID-19 pandemic.^2^ As a positive-strand RNA virus, SARS-CoV-2 shares several core enzymes with other coronaviruses (CoVs), including RNA-dependent RNA polymerase (RdRp or nsp12), main protease (M^Pro^ or nsp5), papain-like protease (PL^Pro^ or nsp3), and helicase/ATPase (nsp13).^3^ Given the history of SARS-CoV, MERS-CoV, and SARS-CoV-2, targeting conserved gene products with broad-spectrum antivirals remains a cornerstone of preparedness for future CoV pandemics. RdRp is an optimal target for function and conservation. It has ∼96% sequence identity shared between SARS-CoV and SARS-CoV-2.^4^ However, directly targeting RdRp with non-nucleotide small molecules is challenging because the enzyme has a large, relatively shallow RNA-binding interface. Accordingly, the approved RdRp-targeting therapeutics are nucleotide analogs that are incorporated into viral RNA for either terminating synthesis or inducing lethal mutagenesis.^5^ By comparison, nsp13 is more conserved with ∼99.8% amino acid sequence identity between SARS-CoV and SARS-CoV-2.^6^ In principle, nsp13 is an appealing pan-coronavirus target. Substantial drug discovery work is ongoing,^7^ but no nsp13 inhibitors have reached clinical approval and none are currently in clinical trials. Key hurdles include the protein’s highly dynamic 5-domain protein architecture and a large charge-rich nucleic acid-binding groove, both of which complicate the design of potent, selective small molecule inhibitors. PL^Pro^ is a cysteine protease that processes pp1a and pp1ab and exerts deISGylase/deubiquitinase activity to modulate the host innate immunity. Potent noncovalent and covalent PL^Pro^ inhibitors have been developed.^8,9^ One inhibitor, HL-21, has entered Phase II clinical testing in China.^10^ However, PL^Pro^ is less conserved across β-CoVs and has only 83% amino acid sequence identity between SARS-CoV and SARS-CoV-2, which may limit truly broad-spectrum coverage.^3^ In addition, PL^Pro^ recognizes an LXGG motif and cleaves after Gly–Gly, creating a narrow active site cleft that poses extra design constraints. Among these enzymes, M^Pro^ remains a particularly attractive target for pan-CoV inhibitor development. M^Pro^ active site residues are highly conserved across most β-CoVs, and this conservation extends into alpha, gamma, and delta families.^11^ This combination of functional essentiality and active site conservation underpins the success of current M^Pro^-targeting antivirals and supports continued efforts toward broad-spectrum inhibitors. When Paxlovid (a combination therapy of nirmatrelvir and ritonavir) was authorized, there were understandable concerns about the development of M^Pro^ resistance, and in vitro selection studies indeed produced variants with reduced susceptibility to nirmatrelvir.^12^ Sporadic resistant mutations have also been detected in clinical isolates.^13^ However, these cases remain uncommon and often carry fitness costs. This is in sharp contrast to the extensive antigenic drift in Spike that has repeatedly eroded Spike-directed vaccines and antibodies.^14^ Taken together, the functional constraint and genetic stability of M^Pro^ support its robustness as an antiviral target. This therapeutic potential has been validated by the global clinical approval of several M^Pro^-targeting antivirals. Prominent examples include nirmatrelvir, a peptidomimetic inhibitor coadministered with ritonavir in Paxlovid, alongside other approved peptidomimetic and small-molecule agents.^15–19^ Despite prominent success, approved M^Pro^-targeting antivirals still present challenges, including viral resistance, potential drug–drug interactions, administrative constraints, and modest reductions in symptom duration.^20–23^ Therefore, there is still a need for novel M^Pro^ inhibitors that potentially resolve these limitations and serve as antiviral reserves for the preparedness of future coronavirus pandemics.

M^Pro^ contains a canonical catalytic dyad (Cys145-His41) and cleaves substrates after the P1 Gln residue. As defined by Schechter-Berger nomenclature, the active site consists of subsites S1–S4 and S1 (Figure 1A). These regions bind corresponding substrate residues P1–P4 and P1′, orienting the P1–P1′ scissile bond for hydrolysis.^24^ S1 imposes a strict preference for P1 Gln; S2 is a hydrophobic pocket that tolerates bulky residues; S3 is largely solvent-exposed and contributes little specificity; S4 is a compact pocket favoring small hydrophobic residues such as Val; and although S1′ is relatively spacious, current evidence suggests a limited role in substrate selectivity. Most peptidomimetic inhibitors occupy S1, S2, and S4, with some engaging S3 and/or extending into S1′.^25,26^ To enhance affinity and metabolic stability, the S1 element is commonly a γ-lactam that recapitulates the P1 Gln hydrogen bond network with His163 and Glu166.^27^ Many inhibitors, including nirmatrelvir, incorporate an electrophilic warhead to engage Cys145 covalently. Nonpeptidomimetic small molecule inhibitors, exemplified by the Japanese-approved drug ensitrelvir,^16^ also exploit binding combinations of S1–S4 and S1. High potency in nonpeptidomimetic inhibitors typically necessitates higher molecular weights, exemplified by ensitrelvir (531 Da). Following the serendipitous identification of the nicotine derivative 3-vinylpyridine as an MPro S1 binder, we developed a class of potent, compact nonpeptidomimetic covalent inhibitors. In contrast to peptidomimetics that depend on extended hydrogen bonding, these inhibitors derive their potency from a covalent bond with Cys145 supplemented by van der Waals contacts. Crystal structures of two M^Pro^-inhibitor complexes reveal dual covalent bonds formed with both Cys145 and His41, implying a novel chemistry to covalently cross-link cysteine and histidine simultaneously in proteins.

**Figure 1:**
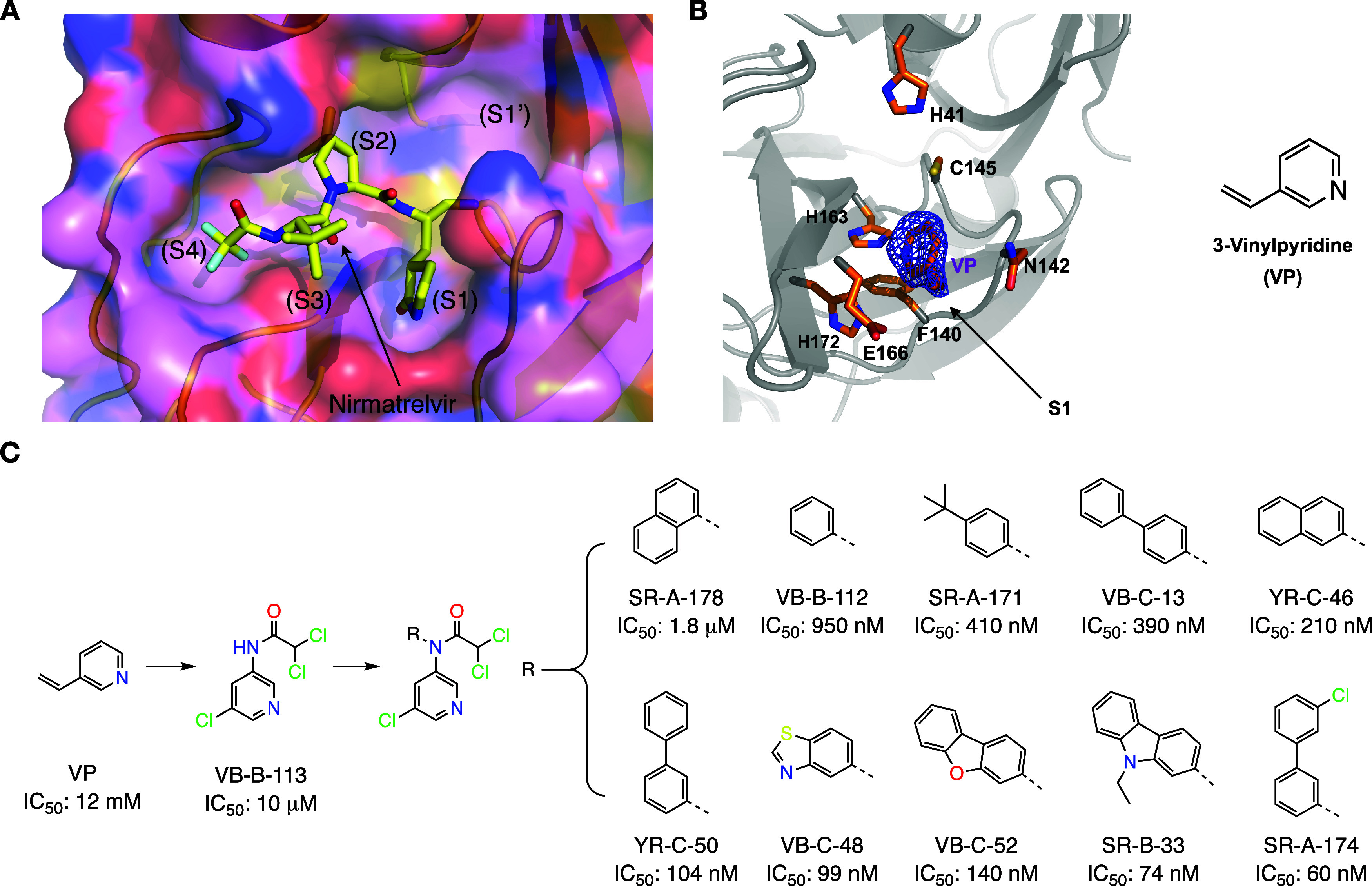
(A) The five active site pockets, as defined by the Schechter–Berger nomenclature and labeled as S1, S2, S3, S4, and S1′ in parentheses, of M^Pro^. The partially transparent contoured surface and secondary structure of M^Pro^ are based on a pdb entry: 7RFS, which is the crystal structure for M^Pro^ complexed with nirmatrelvir. Nirmatrelvir is presented in a stick representation. (B) 3-Vinylpyridine (VP) and its binding to the S1 pocket of M^Pro^. 2Fo-Fc electron density map around VP is contoured at the 1σ level. The structure is based on a pdb entry: 9BS7. (C) A quick de novo design scheme from VP to potent M^Pro^ inhibitors using a dichloroacetamide warhead to covalently engage M^Pro^ at its active site cysteine Cys145.

## Results and Discussion

### From 3-Vinylpyridine (VP), a Pyrolysis Product of Nicotine to SR-A-174, a Potent M^Pro^ Inhibitor

During the early phase of the COVID-19 pandemic, reports suggested that active smokers had lower rates of SARS-CoV-2 infection.^28,29^ Motivated by this observation, we evaluated nicotine and its pyrolysis products as potential M^Pro^ inhibitors. We previously crystallized apo-M^Pro^ and used apo-M^Pro^ crystals to soak with inhibitors to obtain M^Pro^-inhibitor complex structures.^30–32^ We adopted this crystallography method to conduct fragment-based drug discovery by screening nicotine and its pyrolysis products, including myosmine, 3-methylpyridine, 3-ethylpyridine, 3-vinylpyridine (VP), 3-pyridinecarbonitrile, β-nicotyrine, nornicotine, nicotyrine, anabasine, and cotinine. Only VP was found bound in the M^Pro^ active site. As shown in Figure 1B, the electron density unambiguously places the VP in the S1 pocket. In this pose, the VP pyridine ring is enclosed by Phe140, Asn142, Cys145, His163, Glu166, and His172, filling the pocket with minimal void volume. Its ring nitrogen forms a hydrogen bond with the His163 *N*ε atom. The vinyl group of VP lies above the Glu166 carboxylate, suggesting a favorable π stacking interaction that may explain the selective binding of VP. The VP pyridine superimposes the γ-lactam of nirmatrelvir in the M^Pro^-nirmatrelvir complex (Figure 1A), with its C5 approximately colocalized with the P1 γ-carbon of nirmatrelvir. The Cys145 thiolate sits directly above VP. Using a validated M^Pro^ enzymatic assay,^33^ we determined VP with an IC_50_ of 12 mM. Inspired by the M^Pro^-VP structure and to recapitulate the covalent mechanism observed for nirmatrelvir and other peptidomimetic/nonpeptidomimetic inhibitors while retaining a carbonyl group capable of binding the oxyanion hole in M^Pro^, we installed a 2,2-dichloroacetamide at the pyridine C3 position, and for synthetic simplicity, replaced the vinyl group with chloride. Prior work by Wang et al. has described 2,2-dichloroacetamide as a covalent warhead to engage Cys145.^34^ Given its comparatively low intrinsic toxicity and its presence in chloramphenicol, a widely used antibiotic, we install this warhead to make VB-B-113, which showed an IC_50_ of 10 μM. This 1000-fold more potent improvement over VP indicates successful covalent engagement of Cys145 by the 2,2-dichloroacetamide.

VB-B-113 features a 5-chloropyridine that occupies the M^Pro^ S1 pocket and an α,α-dichloroacetamide warhead that covalently engages Cys145. In this binding model, the other subsites in the M^Pro^ active site are unoccupied, leaving a large space for conducting de novo designs to reach novel inhibitors. Because VB-B-113 uses a 3-amino group to append the 2,2-dichloroacetyl moiety, and this amino group colocalizes with the P1 Cα in the M^Pro^-nirmatrelvir structure, we reasoned that adding an additional substituent at this position could reach S2 and potentially S1′ and S4 to enhance binding. This amino group also permits straightforward synthetic diversification. Given that S2 is a large, hydrophobic pocket that accommodates residues such as leucine and phenylalanine and the transition space between S1 and S2 pockets is a flat interface, we focused on installing aromatic substituents at the 3-amino position of VB-C-113 and rapidly synthesized ten analogs (Figure 1C). Bicyclic aromatic rings were tested for a potentially large interaction interface with both S2 and the bridging space between S1 and S2. On the contrary, different biphenyl groups were examined for reaching deep into the S2 pocket. Among all 10 molecules, all but SR-A-178 showed IC_50_ below 1 μM. Three compounds, VB-C-48, SR-B-33, and SR-A-174, exhibited an IC_50_ value below 100 nM, and SR-A-174 has the lowest IC_50_ of 60 nM among this group. With this high potency, these molecules are among the most potent inhibitors that have been developed for M^Pro^ so far. This group of molecules is *N*,*N*-diaryl-2,2-dichloroacetamides, representing a structurally very simple and completely novel group of potential antivirals for SARS-CoV-2.

### Exploration of a Large Variety of Covalent Warheads to Engage Cys145 of M^Pro^

VB-B-112 was the first inhibitor in this compound series to achieve an IC_50_ value below 1 μM. After realizing that its *N,N*-diaryl-2,2-dichloroacetamide scaffold enables rapid exploration of various covalent warheads by substituting the 2,2-dichloroacetamide moiety, we synthesized five new inhibitors as shown in Figure 2A, bearing 2-ketoamide, Michael acceptor, cyanamide, or carbamate, respectively, for potentially reacting with M^Pro^ Cys145. Among them, only VB-C-20 has a determined IC_50_ value of 3.5 μM, and the others all had IC_50_ values above 20 μM, indicating substantially weaker potency than VB-B-112.

**Figure 2:**
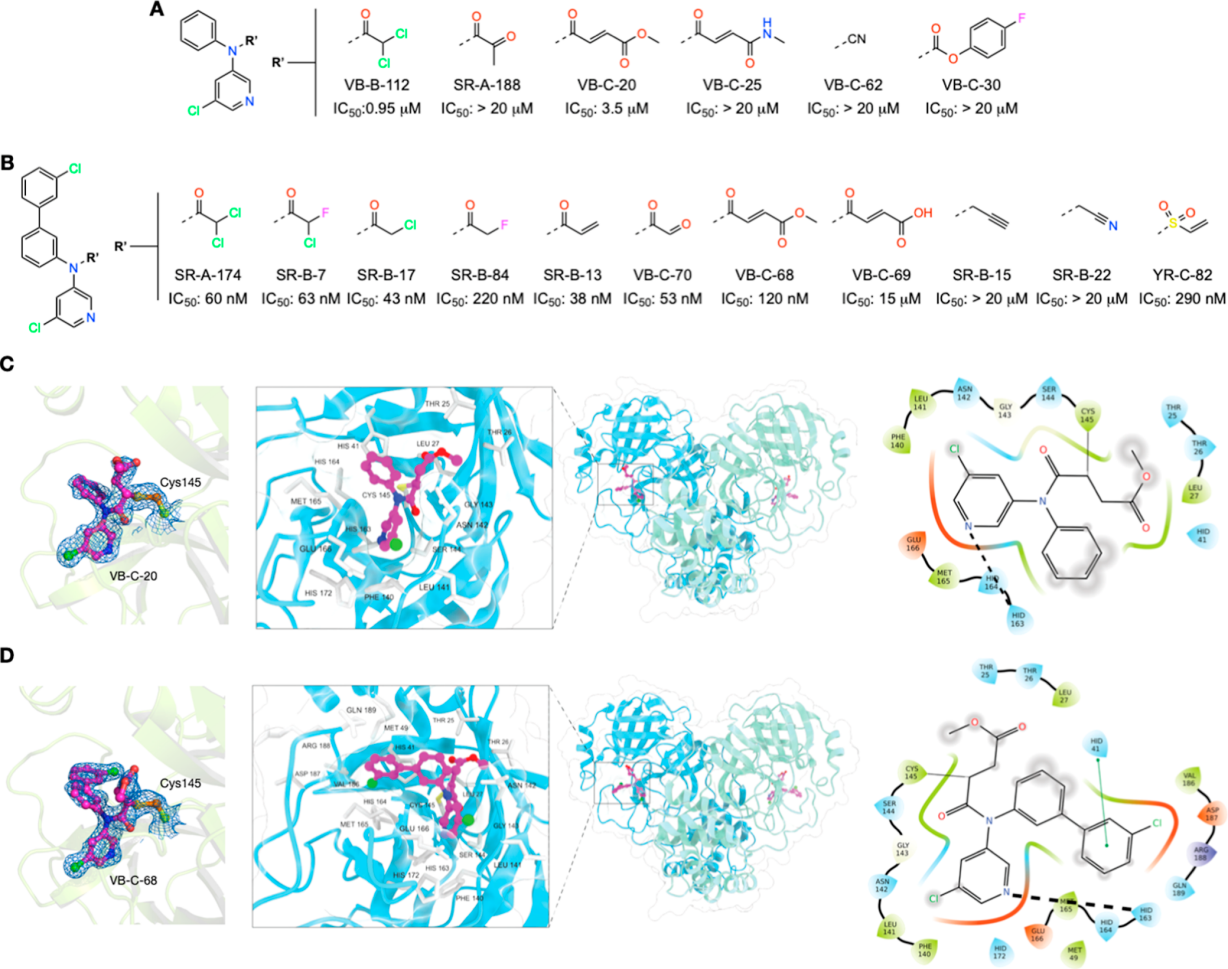
(A) A search of various covalent warheads to engage M^Pro^ at its Cys145 based on an initial hit compound VB-B-112. (B) A similar but expanded search based on a more potent hit compound, SR-A-174. (C) Crystal structure of M^Pro^ complexed with VB-C-20. 2Fo-Fc electron density map around VB-C-20 and Cya145 side chain is contoured at the 1σ level and shown at the left side. Carbon atoms of VB-C-20 are colored in purple, which is different from orange for a Cys145 side chain carbon. Shown in the middle are an overall M^Pro^ dimer and a zoomed-in active site showing binding of VB-C-20 at the M^Pro^ active site. Carbon atoms of VB-C-20 are colored in orange, and M^Pro^ residues surrounding VB-C-20 are colored in gray. Shown on the right is a 2D interaction map between VB-C-20 and surrounding M^Pro^ residues in the M^Pro^ active site. (D) Crystal structure of M^Pro^ complexed with VB-C-68 presented similarly to that of C.

Discovery of SR-A-174 as a more potent inhibitor than VB-B-112 prompted us to adopt SR-A-174 as a core for further covalent warhead optimization. Based on SR-A-174, we designed and synthesized 10 additional inhibitors, as shown in Figure 2B. The covalent groups examined included 2-chloro-2-fluoroacetamide, 2-chloroacetamide, 2-fluoroacetamide, acrylamide, glyoxamide, *O*-methyl-fumaryl, fumaryl, propargylamine, cyanomethylamine, and vinylsulfonamide. Notably, 2,2-dichloroacetamide and 2-chloro-2-fluoroacetamide have both appeared in recent covalent inhibitor designs.^35–37^ Replacing one chloride in SR-A-174 with a less reactive fluoride to afford SR-B-7 was expected to reduce potency. However, SR-B-7 retained essentially the same activity (IC_50_: 63 nM) as SR-A-174, suggesting that the smaller fluoride may reduce steric hindrance for binding to the M^Pro^ active site and partially offset its lower intrinsic reactivity. SR-B-17 carries 2-chloroacetamide, a moiety known to react efficiently with cysteine. It exhibited improved potency with an IC_50_ of 43 nM. In contrast, SR-B-84, with a 2-fluoroacetamide that is considerably less reactive than 2-chloroacetamide, showed reduced potency (IC_50_: 220 nM). The drop, however, was smaller than expected from calculating the intrinsic electrophilicity difference between the two warheads, implying that 2-fluoroacetamide may contribute more favorable noncovalent binding at the M^Pro^ active site.

SR-B-13 contains an acrylamide warhead, a motif present in several FDA-approved covalent drugs.^38,39^ It has an IC_50_ of 38 nM, indicating a stronger potency than SR-A-174. VB-C-70 has a glyoxamide. Its aldehyde is substantially more reactive toward cysteine than the ketone in SR-A-188. VB-C-70 displayed an IC_50_ of 53 nM, slightly better than that of SR-A-174. Compared to SR-B-13, VB-C-68 has an additional methyoxycarbonyl cap on its acrylamide warhead. Adding this cap was expected to temper nonspecific reactivity but introduce steric hindrance. Accordingly, VB-C-68 showed an IC_50_ of 120 nM, about 3-fold higher than that of SR-B-13. In VB-C-69, a free carboxylate was conjugated to the acrylamide double bond. At physiological pH, this carboxylate is deprotonated, rendering the Michael acceptor more electron-rich and thus less electrophilic. Correspondingly, VB-C-69 had an IC_50_ of 15 μM. We also evaluated propargyl (SR-B-15) and cyanomethyl (SR-B-22) as alternative cysteine-targeting warheads, but both compounds have weak potency (IC_50_: >20 μM). Finally, YR-C-82 features a vinylsulfonamide that is intrinsically more reactive than acrylamide toward cysteine. Nevertheless, YR-C-82 was less potent than SR-B-13 and showed an IC_50_ of 290 nM. This weaker potency is likely caused by the bulky sulfonyl group compromising the formation of the initial Michaelis complex required for productive covalent bond formation.

Using the previously described soaking strategy,^30^ we determined crystal structures of M^Pro^ complexed with five new inhibitors, including VB-C-20, VB-C-68, SR-B-7, SR-B-13, and VB-C-70. As shown in Figure 2C, the two aromatic rings of VB-C-20 were unambiguously modeled in the active site of M^Pro^. Electron density maps placed a chloropyridine in the S1 subsite, a phenyl group in the bridging space between S1 and S2, and a fumarate moiety covalently linked with Cys145. The Cys145 thiolate was added to the α-carbon next to the core amide group. As in most M^Pro^ structures, the enzyme dimer constitutes the asymmetric unit. In S1, the chloropyridine fits tightly, with its chloride nestled between Asn142 and Glu166, and its ring nitrogen forms a hydrogen bond with His163 Nε. The amide oxygen of VB-C-20 occupies the oxyanion hole formed by the backbone NHs of Gly143, Ser144, and Cys145. Beyond the Cys145 covalent linkage, the oxyanion hole interaction, and the hydrogen bond to His163, the remaining contacts are primarily van der Waals. The phenyl ring of VB-C-20 lies within van der Waals distance of Met165 but does not form close interactions with other residues. While spanning the bridging space between S1 and S2, VB-C-20 does not penetrate deeply into S2. The S2 subsite, as well as S3, S4, and S1′, remains completely unoccupied, indicating clear vectors for potency optimization.

Compared to VB-C-20, VB-C-68 carried an additional distal *meta*-chlorophenyl group on a proximal *N*-phenyl substituent, enabling deeper engagement with S2. This was confirmed by the determined M^Pro^-VB-C-68 structure (Figure 2D), in which electron density maps at the active site permitted unambiguous modeling of VB-C-68. As shown in this determined structure, VB-C-68 adopts an overall pose similar to that of VB-C-20, but the distal *meta*-chlorophenyl group projects deep into S2, forming extensive van der Waals contacts with side chains of His41, Met49, His164, Met165, and Asp187, and backbones of Arg188 and Gln189. The chloride atom of *meta*-chlorophenyl sits tightly in a small hydrophobic pocket formed by His41, His164, Met165, and Asp187 without steric clashes. Compounds exploiting this S2 subpocket have likewise emerged from the COVID Moonshot project.^40^ In contrast to nirmatrelvir, which combines a covalent interaction, an extended hydrogen bond network, and engaging S1, S2, S3, and S4 pockets to achieve strong binding to M^Pro^, VB-C-68 achieves strong inhibition with a covalent bond, one hydrogen bond, and primarily occupancy of S1 and S2, leaving S3, S4, and S1′ completely available for further potency and pharmacology gains. Crystal structures for the remaining three complexes are shown in Figures S1–S3. Apart from differences at the covalent warhead position, SR-B-7, SR-B-13, and VB-C-70 displayed binding modes at the M^Pro^ active site that are essentially the same as that for VB-C-68.

### Exploring Different S1 Binders for Potency Optimization

Using SR-A-174 as a core scaffold, we sought alternative S1 binders that may enhance the M^Pro^ inhibition. We prioritized heteroaromatic rings that were expected to preserve the His163 hydrogen bond and also evaluated the presence of a propanamide motif in native M^Pro^ substrates. In total, 15 distinct S1 binders were incorporated to generate compounds, as shown in Figure 3. These new S1 binders included pyridine and pyrimidine variants, diazine, oxazole, thiadiazole, triazole, and isoquinoline. Determined IC_50_ values together with the finally afforded inhibitors are presented in Figure 3. VB-C-199, JK36, and VB-D-24 likely disrupt the His163 hydrogen bond and consequently have low potency. VB-C-79 has weaker potency than SR-A-174, indicating a suboptimal S1 fit for 2-choropyrimidine. VB-C-86, VB-C-90, and VB-C-98 all bear five-membered heteroaromatics. They have poor activity, indicating that oxazole/diazole-type rings are disfavored S1 binders. The propanamide in YR-C-73 yields a low potency, reinforcing the preference for heteroaromatic or lactam moieties at S1.

**Figure 3:**
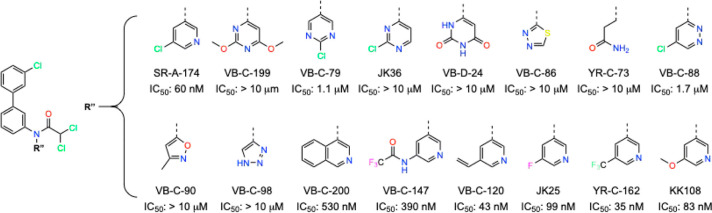
A search of chemical moieties with a primary focus on heteroaromatic rings that can bind to the M^Pro^ S1 pocket.

VB-C-88 employs a chlorodiazine as its S1 binder. Although replacing chloropyridine with the more electron-deficient chlorodiazine can potentially increase electrophilicity at the warhead and thus facilitate reaction with Cys145, VB-C-88 is a markedly weaker inhibitor compared with SR-A-174 and has an IC_50_ of 1.7 μM. This may reflect a weaker hydrogen bond interaction with His163 and/or a higher desolvation penalty from its increased polarity compared to SR-A-174. VB-C-200 has an isoquinoline as its S1 binder. It is about 8-fold less potent than SR-A-174. This weaker potency is likely due to steric mismatch of the large bicyclic system in S1. The remaining five analogs, VB-C-147, VB-C-120, JK25, YR-C-162, and KK108, all featured meta-substituted pyridines as S1 binders. Except for VB-C-147, all exhibited IC_50_ below 100 nM. Notably, VB-C-120 (IC_50_: 43 nM) and YR-C-162 (IC_50_: 35 nM) have potency better than that of SR-A-174. The vinyl group in VB-C-120 likely contributes to strong binding by forming a favorable π stacking interaction with Glu166. For YR-C-162, its trifluoromethyl group is strongly electron-withdrawing. This strong electron-withdrawing effect is expected to increase electrophilicity of the warhead for covalent conjugation with Cys145. Although VB-C-120 and YR-C-162 are more potent than SR-A-174, their overall potencies are comparable in magnitude. Therefore, SR-A-174 was continuously used as a core scaffold to search for the optimal inhibitors for M^Pro^.

### A Campaign to Search for Optimal Binders for S2 and Potentially S3 or S4

VB-C-68, SR-B-7, SR-B-13, and VB-C-70 are structurally similar to those of SR-A-174. Crystal structures of their M^Pro^ complexes showed a distal *meta*-chlorophenyl group occupying S2 and a proximal *N*-phenyl group occupying the bridging space between S1 and S2. Because S3 and S4 are not engaged, we hypothesized that adding interactions with S3 and S4 would increase the potency. Crystal structures of this new series of compounds in their M^Pro^ complexes also indicate that the proximal *N*-phenyl ring forms only weak contacts with M^Pro^ residues, suggesting that replacing this group with moieties capable of broader interactions could further enhance potency. Guided by these two design principles, we synthesized 22 new inhibitors, as shown in Figure 4A. With the exception of SR-B-101, the top row compounds retained the SR-A-174 scaffold but incorporate substituents on the distal phenyl ring to potentially reach S4. SR-B-101 introduces a bridging furan oxygen that constrains the proximal and distal phenyl groups into a dibenzo­[*b*,*d*]­furan, a strategy intended to reduce conformational entropy loss upon binding. In this set, all compounds except VB-C-124 show IC_50_ values around or below 100 nM. Six inhibitors, including SR-B-120, VB-C-140, SR-B-101, YR-C-154, and YR-C-163, were more potent than SR-A-174. SR-B-120, VB-C-140, and YR-C-154, and each carries a small 5′-substituent on the distal phenyl ring that can extend toward S4. Their superior potency validates this design. By contrast, KK114, VB-C-171, VB-C-172, and VB-C-124 bear relatively large 5′-substituents and were less potent than SR-A-174, indicating that finer tuning of substituent size/chemistry is required for optimal S4 engagement. SR-B-101 outperforms SR-A-174, supporting the utility of conformational constraint to mitigate entropic penalties. YR-C-161 and YR-C-163 feature a fused 1,2-diazole ring on the distal aryl group. In each, one diazole nitrogen is methylated to both promote potential S4 interactions and decrease overall hydrophilicity. Both achieve IC_50_ values below 40 nM. Overall, six compounds surpass SR-A-174, with VB-C-140 demonstrating approximately twice the greater potency, validating distal ring modification as a practical route to improve M^Pro^ inhibition potency.

**Figure 4:**
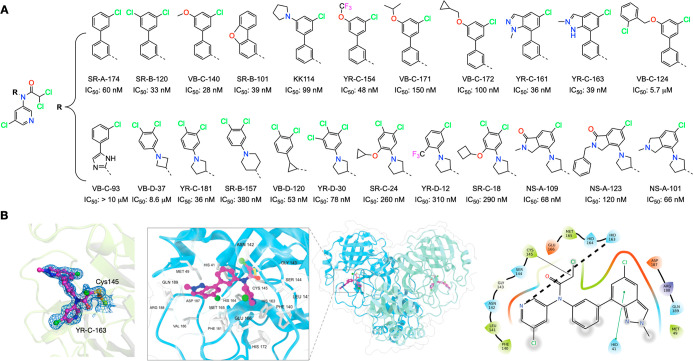
(A) A large medicinal chemistry campaign to identify optimal chemical moieties that can occupy M^Pro^ S2 pocket and potentially reach S4 pockets. (B) Crystal structure of M^Pro^ complexed with YR-C-163 presented similarly as in Figure 2C.

Since the proximal phenyl ring engages only weakly with M^Pro^, we replaced it with alternative cyclic moieties (bottom row compounds in Figure 4A). We first tested the imidazole. The resulting analog VB-C-93 was a weak inhibitor with an IC_50_ above 10 μM. Motivated by some COVID Moonshot compounds showing that a 3,4-dichlorophenyl group can penetrate deeply into S2,^40^ we adopted this motif while varying the proximal ring. We synthesized four new analogs, VB-C-27, YR-C-181, SR-B-157, and VB-C-120, featuring azetidine, pyrrolidine, piperidine, and cyclopropane, respectively. Among them, YR-C-181 and VB-D-120 outperform SR-A-174, with VB-D-120 being the most potent (IC_50_: 36 nM). We then used VB-D-120 as a lead scaffold to explore distal ring substitutions designed to reach S4. Seven new inhibitors were synthesized. However, none surpass VB-D-120, although three (YR-D-30, NS-A-109, and NS-A-101) match SR-A-174 in potency. As discussed above, further fine-tuning will be required to optimize S4 engagement.

We determined the crystal structure of M^Pro^ bound to YR-C-163. The ligand was unambiguously modeled in the active site that shows clear electron density for two chlorides binding at S1 and S2. Cys145 forms a covalent bond with the 2-carbon of the core acetamide (Figure 4B). YR-C-163 features 5-chloro-2-methyl-indazole as a distal aryl group that penetrates deep into S2, fully occupying this pocket. The additional 2-methyldiazole in the distal aryl group makes close contacts with the Arg188-Thr190 loop that transitions between S2 and S4. Compared with the M^Pro^-nirmatrelvir complex (pdb entry: 7RFS), this loop is displaced toward solvent to accommodate 2-methyldiazole. Although direct engagement of S4 is not observed, the distal aryl group extends into the corridor bridging S2 and S4, providing a clear path for further designs to access S4.

### Cellular M^Pro^ Inhibition and Antiviral Potency Tests of Newly Developed Inhibitors

When transiently expressed in HEK293T cells, M^Pro^ triggers acute cytotoxicity leading to cell death.^41^ Cellularly permeable inhibitors counter this effect and rescue host cells. Building on this observation, we developed a cell-based assay to quantify intracellular potency for M^Pro^ inhibitors. In this assay, HEK293T cells are transiently transfected to express M^Pro^-eGFP and cultured with the test compounds. Potent inhibitors suppress M^Pro^-mediated cytotoxicity, thereby improving cell survival and increasing M^Pro^-eGFP expression. The resulting rise in fluorescence is then measured by flow cytometry as a quantitative efficacy readout to determine the cellular IC_50_ values of the tested compounds. To date, this assay has been applied to more than 100 M^Pro^ inhibitors to prioritize M^Pro^ inhibitors for further preclinical characterizations.^42–45^ Using this assay, we assessed intracellular potency of all newly developed inhibitors with their enzymatic IC_50_ ≤ 100 nM. The correspondingly determined cellular IC_50_ values are summarized in Table 1. Fourteen compounds show cellular IC_50_ > 10 μM, indicating limited cellular activity/permeability. Four exhibit single digit micromolar cellular IC_50_ values and five achieve cellular IC_50_ < 1 μM. For all inhibitors with cellular IC_50_ < 10 μM, we measured antiviral potency in A549-hACE2 cells, a well-established model for SARS-CoV-2 infection, using the SARS-CoV-2 strainUSA_WA1/2020 strain.^32^ The resulting EC_50_ values are presented in Table 1. With the exception of SR-B-33 and KK-114 (EC_50_ = 7.1 and 3.5 μM, respectively), all tested inhibitors showed EC_50_ around 1 μM or below, indicating strong antiviral potency. These results warrant further evaluation of this series of compounds as SARS-CoV-2 antiviral candidates. There is a discrepancy between cellular and antiviral potency for SR-B-33 and SR-B-101. Their low antiviral potency for these two molecules could be the consequence of viral challenge of cells leading to a cell membrane structure change that leads to low cellular permeability for the two compounds. We have also tested general cytotoxicity of 5 inhibitors in HEK-293T cells. As shown in Figure Table 1, they display CC_50_ values equivalent to that for nirmatrelvir.

**Table 1:** Representative M^Pro^ Inhibitors and Their Characteristics

ID^a^	enzymatic IC_50_ (μM)	cellular IC_50_ (μM)	antiviral EC_50_ (μM)^b^	CC_50_ (μM)^d^
Nirmatrelvir^c^	0.066	3.4	0.044	31
VB-C-48	0.099	>10		
SR-B-33	0.074	0.096	7.1	
SR-A-174	0.060	>10		
SR-B-7	0.063	>10		
SR-B-17	0.043	>10		
SR-B-13	0.038	>10		
VB-C-70	0.053	>10		
VB-C-120	0.043	1.5	1.2	
JK-25	0.099	>10		
YR-C-162	0.035	>10		
KK-108	0.083	>10		
SR-B-120	0.033	>10		
VB-C-140	0.028	>10		
SR-B-101	0.039	0.080	1.2	
KK-114	0.098	3.6	3.5	
YR-C-154	0.048	>10		
VB-C-172	0.100	0.97	1.6	
YR-C-161	0.036	>10		
YR-C-163	0.039	1.6	0.21	29
YR-C-181	0.036	0.15	0.98	30
VB-D-120	0.053	>10		
YR-D-30	0.078	0.18	0.47	66
NS-A-109	0.068	1.7	0.85	38
NS-A-101	0.066	0.46	0.42	57

a All determined values are reported with two significant numbers.

b In A549-hACE2 cells, against the SARS-CoV-2 Delta variant.

c Data from ref 31.

d Measured in HEK-293T cells for 48 h incubation.

For the two inhibitors SR-A-174 and SR-B-7, we also measured their time-dependent inhibition of M^Pro^. 40 nM M^Pro^ and 10 μM substrate were used in this assay, and a linear increase of part of the production formation was recorded under the conditions without an inhibitor to represent a region without significant substrate concentration change. By adding SR-A-174, we measured the production formation immediately. An exponential increase curve was clearly observable and the enzyme quickly lose activity completely. Fitting this curve to a single exponential increase led to a measured *k*_app_ as 0.19 min^–1^, which represents a quick covalent inhibition process for this compound (Figure S4A). In comparison, SR-B-7 in a similar setting led to a slow inhibition process that cannot be characterized to determine *k*_app_ (Figure S4B). SR-A-174 has the α,α-dichloroacetamide warhead in contrast to a α-chloro-α-fluoroacetamide warhead in SR-B-7. If the fluoride in SR-B-7 serves as a leaving group, then a small kinetic process is expected. However, since SR-B-7 also contains a chloride leaving group, the slow inhibition kinetics is counterintuitive since a more fluoride substitute will make the α-carbon more electron poor for reacting with the active site cysteine. Deeper characterizations will be needed to fully understand the inhibition kinetics of the α-chloro-α-fluoroacetamide warhead.

### *N*,*N*-Diaryl-2,2-dichloroacetamides as Heterobifunctional Cross-Linkers for Both Cys145 and His41 at the M^Pro^ Active Site

VB-B-112 was the first compound in this nonpeptidomimetic inhibitor series to achieve an enzymatic IC_50_ below 1 μM. We initiated the crystallography study of this compound immediately after its biochemical characterization. Calculated electron density maps revealed additional active site electron density bridging both Cys145 and His41, indicating that two covalent bonds formed separately with the two catalytic dyad residues (Figure 5A). The electron density was best fit to *N*-(5-Chloropyridin-3-yl)-*N*-phenylacetamide, modeled with its core acetamide 2-carbon forming two covalent bonds with both Cys145 thiol and His41 Nε. Electron density around both *N*-aryl substituents was weak. In the refined model, the 5-chloropyridinyl substituent occupies S2 and its chloride atom points to the Val186-Thr190 loop, while the *N*-phenyl group projects into S1′, making van der Waals contact with Thr25. No hydrogen bond interactions were observed for either the core acetamide oxygen or the 5-chloropyridyl nitrogen. Although S2 and S1 are hydrophobic pockets, both *N*-substituents appear to make only modest hydrophobic contacts, and the attenuated electron density suggests some rotational flexibility of both rings (Figure 5A). For SR-A-171, we observed clear electron density in the active site indicating the same covalent attachments of the inhibitor to both Cys145 and His41 (Figure 5B). As with VB-B-112, the 5-chloropyridinyl moiety resides also in S2. Here, the electron density is sufficiently well-defined to place the ring with its chloride buried deep in S2. The *N*-4-*tert*-butyl-phenyl substituent occupies S1′ and positions the *tert*-butyl group near the Thr45-Glu47 loop. Compared with the M^Pro^-nirmatrelvir complex (pdb: 7RFS), this loop shifts away from Thr25 to create a more open S1′ pocket (Figure S5). Similar to the case for VB-B-112, no hydrogen bond is formed for SR-A-171 in the M^Pro^ active site. To confirm dual covalent bond formation, we incubated M^Pro^ with VB-B-112 and then conducted its molecular weight characterization using electrospray ionization mass spectrometry analysis (ESI-MS).

**Figure 5:**
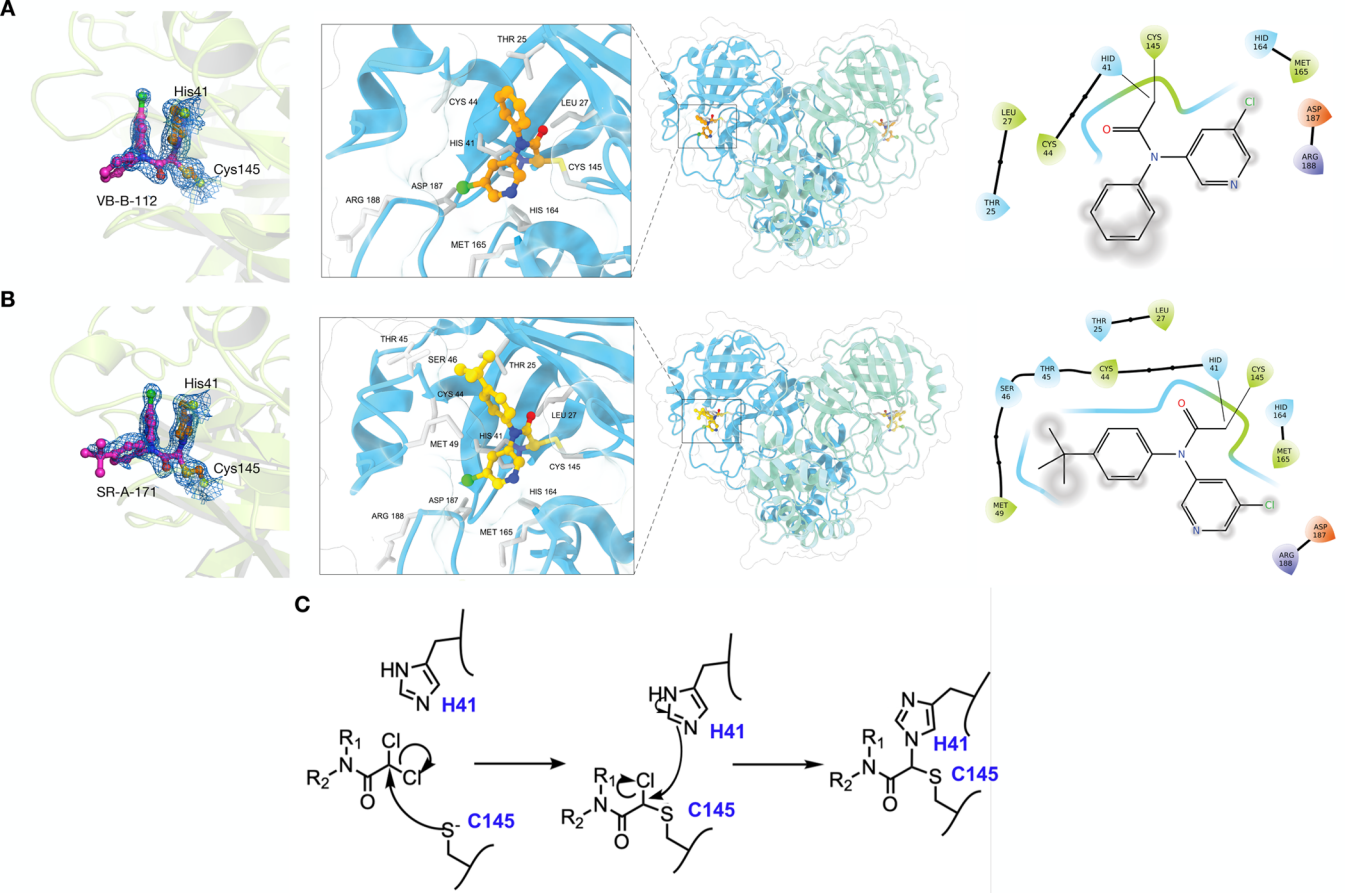
(A) Crystal structure of M^Pro^ complexed with VB-B-112. (B) Crystal structure of M^Pro^ complexed with SR-A-171. Both structures are presented similarly as in Figure 2C. (C) A proposed two-step reaction mechanism for a dichloroacetamide functional group to covalently engage both Cys145 and His41 at the active site of M^Pro^.

VB-C-20 differs from VB-B-112 only in the covalent warhead. It carries a Michael acceptor that enables the formation of a single covalent bond with Cys145. As shown in Figure 2C and described above, its *N*-5-chloropyridinyl substituent occupies S1 as desired, while its *N*-phenyl group spans a bridging space between S1 and S2 without forming strong contacts. Given the close similarity between VB-C-20 and VB-B-112, we anticipated that VB-B-112 would adopt a comparable pose if its warhead forms only a single covalent bond with Cys145. Indeed, a single-bond mechanism was observed for YR-C-163 (Figure 4B), which carries the same 2,2-dichloroacetamide warhead but a bulky *N*-substituent that anchors deeply in S2. However, our crystal structure of M^Pro^-VB-B-112 revealed bifunctional cross-linking to both Cys145 and His41 and dislodging the *N*-5-chloropyridinyl substituent from S1 to S2. A plausible mechanism (Figure 5C) is that VB-B-112 first reacts with the Cys145 thiolate via S_N_2 displacement of one chloride on the 2,2-dichloroacetamide to generate a 2-chlorothioether intermediate that initially mimics the VB-C-20/YR-C-163 pose. Because VB-B-112 bears a relatively small *N*-phenyl substituent occupying the open S1–S2 interface, this intermediate can reorient, dislodging the *N*-5-chloropyridinyl group from S1 toward S2 and positioning 2-chloride for a second S_N_2 reaction with His41 to form a covalent bond. By contrast, VB-C-20 lacks a second electrophile to support the follow-on reaction, and YR-C-163 is sterically locked by its large *N*-substituent in S2, preventing the reorientation required for His41 attack. A same mechanism applied to SR-A-171 as well. To further validate this mechanism, M^Pro^ was incubated with VB-B-112 and then subjected to electrospray ionization mass spectrometry (ESI-MS) analysis. The deconvoluted spectrum of the M^Pro^-VB-B-112 complex showed molecular weights of 34,098 Da, matching the theoretical molecular weight value of M^Pro^-VB-B-112 by forming two covalent bonds to replace the two chloride atoms in VB-B-112 (Figure S6). The free enzyme showed a molecular weight of 33,855 Da. The ESI-MS results strongly support the proposed mechanism shown in Figure 5C. An alternative mechanism is the formation of a methylene sulfonium intermediate by losing the second chloride following the first step of the S_N_2 reaction. Since this mechanism will not necessarily need a flexibly bound ligand, other inhibitors with a dichloroacetamide warhead are expected to form a similar intermediate. Since we did not observe histidine conjugation for inhibitors other than VB-B-112 and SR-A-171, we believe the two-step chloride replacement mechanism is more likely to be what really happens during M^Pro^ interactions with VB-B-112 and SR-A-171.

Histidine is one of the key nucleophilic residues in proteins and is frequently positioned in enzyme active sites, where it serves as a general acid/base, metal ligand, or catalytic dyad/triad partner. Although ligand-directed chemistries that modify histidine have been reported, selective histidine bioconjugation under physiological conditions remains uncommon.^46,47^ In practice, cysteine thiolates are intrinsically more reactive, and lysine side chain amines are also abundant and readily acylated. Both can outcompete histidine for most electrophiles. A small but growing toolkit targets histidine under mild aqueous conditions. Notable examples include thiophosphorodichloridates as histidine-directed phosphorylation mimicking reagents, 2-cyclohexenone as a histidine-based Michael acceptor, a ferritin-based metalloenzyme-catalyzed histidine aza-Michael addition, and visible-light-activated thioacetals that generate thionium electrophiles for *N*-alkylating of histidine.^48–51^ Our crystal structures of M^Pro^-VB-B-112 and M^Pro^-SR-A-171 and related ESI-MS results suggest an additional, previously unrecognized option: 2-alkylthio-2-chloroacetamides as covalent histidine modifiers. In both examples, the *N*-5-chloropyridinyl substituent relocates to the less favored S2 pocket, implying that the reaction of the 2-alkylthio-2-chloroacetamide intermediate with His41is energetically preferred. Although a full exploration is beyond the scope of the present study, these observations raise the possibility that 2-alkylthio-2-chloroacetamides or similar chemical groups could be engineered into a new, biocompatible class of histidine-targeting reagents and 2,2-dichloroacetamide can be applied as a cross-linking reagent for bioconjugating nearby cysteine and histidine.

## Conclusions

A large number of M^Pro^ inhibitors have been reported. The COVID-19 Moonshot project alone generated hundreds of compounds and publicly released their M^Pro^ cocrystal structures.^40^ Broadly, M^Pro^ inhibitors fall into two classes, peptidomimetics and nonpeptidomimetic small molecules, and each class can be further divided into noncovalent and covalent groups. Representative peptidomimetic inhibitors that have reached the clinic include nirmatrelvir (U.S.), simnotrelvir (China), atilotrelvir (China), and leritrelvir (China).^17–19,52^ All are structurally related and share a common mechanism of forming a reversible covalent bond with Cys145 in M^Pro^. To date, no noncovalent peptidomimetic inhibitors have advanced to clinical testing. Being a representative nonpeptidomimetic small molecule inhibitor of M^Pro^ that works via a noncovalent binding mechanism, ensitrelvir has been approved for clinical use in Japan.^16^ A related compound that inhibits M^Pro^ via a covalent binding mechanism has also entered clinical evaluation.^53^ In parallel, several research groups have used four-component Ugi reactions and other approaches to generate nonpeptidomimetic series.^34,54^ Among these, ISM3312 has progressed to Phase I trials in China.^55^ Even so, peptidomimetic scaffolds currently outnumber small molecules among the clinically tested M^Pro^ inhibitors. Since continued discovery is essential for future CoV pandemic preparedness, new chemotypes with distinct binding and inhibition mechanisms are especially valuable for counteracting drug resistance. Inspired by nicotine, we developed *N*,*N*-diarylacetamides that bear electrophiles at the acetamide 2-carbon as covalent, nonpeptidomimetic M^Pro^ inhibitors. Many members of this new series show high potency. The synthetic simplicity of this new inhibitor series enables a broad exploration of chemical space toward potent, pan-CoV antivirals, an opportunity that warrants continued investigation. In a parallel campaign that took advantage of the pyridine binding at S1, we developed a number of potent peptidomimetic inhibitors with strong potency for M^Pro^ mutants resistant to nirmatrelvir.^56^ Given that the current series uses the same S1 binding moiety, they are expected to be potent against these mutant M^Pro^ as well.

During structural analysis of inhibitors bound to M^Pro^, we serendipitously observed that the 2,2-dichloroacetamide warhead can covalently engage both Cys145 and His41 by replacing both chlorides, yielding a two-carbon bridge that links the two catalytic dyad residues. Because cysteine is the most nucleophilic amino acid, numerous methods exist for its covalent bioconjugation. By contrast, histidine modification approaches remain comparatively limited. Our findings suggest that 2-alkylthio-2-chloroacetamides may constitute a new class of histidine-reactive reagents under biocompatible conditions and 2,2-dichloroacetamide may be applied as a general cross-linking agent for nearby cysteine and histidine. Their related chemistry warrants systematic evaluation on scope, context dependence, selectivity, and kinetics. For example, the conjugation of both cysteine and histidine may be context dependent since compounds with the dichloroacetamide warhead have been developed for PL^Pro^ but no similar observation of bivalent conjugation was observed.^8^

## Supplementary Material



## Data Availability

All data are available in the main test or the Supporting Information. The crystal structures for M^Pro^ complexed with various inhibitors have been deposited into the Protein Data Bank with the following entry codes: 9BSG (VB-C-20), 9BSP (VB-C-68), 9BSI (SR-B-7), 9BSO (SR-B-13), 9BSQ (VB-C-70), 9BTR (YR-C-163), 9BSA (VB-B-112), and 9BSF (VB-C-68).

## Notes

Experimental procedures for expression and purification of SARS-CoV-2 M^Pro^, characterization of M^Pro^ inhibitors, cellular potency tests, antiviral potency tests, X-ray crystallography analysis, ESI-MS analysis of M^Pro^ bound with VB-B-112; supplementary figures; supplementary tables; and synthesis details and characterizations of compounds by NMR spectroscopy (PDF)
