# Supplementary material for: Nicotine-Inspired, De Novo-Designed SARS-CoV‑2 Main Protease Inhibitors Reveal Unique Chemistry for Covalently Conjugating Both Cysteine and Histidine Residues in the Catalytic Dyad
Source: J Am Chem Soc. 2026 Apr 14;148(16):16985–95. doi: 10.1021/jacs.6c01119 (PMC13133898; doi:10.1021/jacs.6c01119)
Supplement: Supplementary file 1 [file ja6c01119_si_001.pdf]

Supplementary Materials for

Nicotine-Inspired, De Novo-Designed SARS-CoV-2 Main Protease Inhibitors Reveal Unique Chemistry for Covalently Conjugating Both Cysteine and Histidine Residues in the Catalytic Dyad

Authors:

Sandeep Atla[1†], Veerabhadra Vulupala[1†], Yugendar R. Alugubelli[1†], Lauren R. Blankenship[1†], Kai Yang[1†], Satyanarayana Nyalata[1], Kaustav Khatua[1], Demonta Coleman[1], Dorsa Rabie[1], Xuejiao Guo[1], Chia-Chuan D. Cho[1], Sathish Kumar[2,3], Lai Hoang Son Le[1], Banumathi Sankaran[4], Justin K Kalugin[5], Shivangi Sharma[1], Benjamin W. Neuman[2*], Shiqing Xu[1,6*], and Wenshe Ray Liu[1,6,7,8,9*]

Affiliations:
[1]Texas A&M Drug Discovery Center and Department of Chemistry, Texas A&M University, College Station, TX 77843, USA.
[2]Department of Biology, College of Arts and Sciences, Texas A&M University, College Station, TX 77843, United States.
[3]Texas A&M Global Health Research Complex, Texas A&M University, College Station, TX 77843, United States.
[4]Molecular Biophysics and Integrated Bioimaging, Berkeley Center for Structural Biology, Laurence Berkeley National Laboratory, Berkeley, California, 94720, United States
[5]Division of Chemical Biology and Medicinal Chemistry, The University of Texas, Austin, TX 78712, United States
[6]Department of Pharmaceutical Sciences, Irma Lerma College of Pharmacy, Texas A&M University, College Station, TX 77843, United States
[7]Institute of Biosciences and Technology and Department of Translational Medical Sciences, College of Medicine, Texas A&M University, Houston, TX 77030, United States
[8]Department of Biochemistry and Biophysics, College of Agriculture and Life Sciences, Texas A&M University, College Station, TX 77843, United States
[9]Department of Cell Biology and Genetics, College of Medicine, Texas A&M University, College Station, TX 77843, United States

[†]Contribute equally.
[*]Corresponding author. Email: bneuman@tamu.edu (B.W.N.); shiqing.xu@tamu.edu (S.X.); wsliu2007@tamu.edu (W.R.L.)

The PDF file includes:

1. Supplementary Materials and Methods
2. Supplementary Figures (Figs. S1-S6)
3. Supplementary Tables (Table S1-S8)
4. Supplementary Synthesis Details
5. Representative NMR spectra (Synthesis Figs. S1-S112)

1. Supplementary Materials and Methods

Expression and Purification of SARS-CoV-2 M^{Pro}

The expression plasmid pET28a-His-SUMO-CoV-2 M^{Pro} was constructed in a previous study (*43*). We used this construct to transform *E. coli* BL21(DE3) cells. A single colony grown on an LB plate with 25 ⌠g/mL kanamycin was picked and grown in 5 mL LB media supplemented with 25 ⌠g/mL kanamycin overnight. We inoculated this overnight culture to 6 L 2YT media with 25 ⌠g/mL kanamycin. Cells were grown to OD_{600} as 0.8. At this point, we added 1 mM IPTG to induce the expression of His-SUMO-CoV-2 M^{Pro}. Induced cells were let grown for 3 h and then harvested by centrifugation at 12,000 rpm, 4 °C for 30 min. We resuspended cell pellets in 150 mL lysis buffer (20 mM Tris-HCl, 100 mM NaCl, 10 mM imidazole, pH 8.0) and lysed the cells by sonication on ice. We clarified the lysate by centrifugation at 16,000 rpm, 4 °C for 30 min. We decanted the supernatant and mixed with Ni-NTA resins (GenScript). We loaded the resins to a column, washed the resins with 10 volumes of lysis buffer, and eluted the bound protein using elution buffer (20 mM Tris-HCl, 100 mM NaCl, 250 mM imidazole, pH 8.0). We exchanged buffer of the elute to another buffer (20 mM Tris-HCl, 100 mM NaCl, 10 mM imidazole, 1 mM DTT, pH 8.0) using a HiPrep 26/10 desalting column (Cytiva) and digested the elute using 10 units SUMO protease overnight at 4 °C. The digested elute was subjected to Ni-NTA resins in a column to remove His-tagged SUMO protease, His-tagged SUMO tag, and undigested His-SUMO-CoV-2 M^{Pro}. We loaded the flow-through onto a Q-Sepharose column and purified CoV-2 M^{Pro} using FPLC by running a linear gradient from 0 to 500 mM NaCl in a buffer (20 mM Tris-HCl, 1 mM DTT, pH 8.0). Fractions eluted from the Q-Sepharose column was concentrated and loaded onto a HiPrep 16/60 Sephacryl S-100 HR column and purified using a buffer containing 20 mM Tris-HCl, 100 mM NaCl, 1 mM DTT, and 1 mM EDTA at pH 7.8. The final purified protein was concentrated and stored in a -80 °C freezer.

Characterization of M^{Pro} Inhibition by All Synthesized Inhibitors

For all inhibitors, we conducted the assay using 20 nM M^{Pro} and 10 ⌠M Sub3 (DABCYL-Lys-Thr- Ser-Ala-Val-Leu-Gln-Ser-Gly-Phe-Arg-Lys-Met-Glu-EDANS). We dissolved all inhibitors in DMSO as 10 mM stock solutions. Sub3 was dissolved in DMSO as a 1 mM stock solution and diluted 100 times in the final assay buffer containing 10 mM $Na_xH_yPO_4$, 10 mM NaCl, 0.5 mM EDTA, and 1.25% DMSO at pH 7.6. We incubated M^{Pro} and an inhibitor in the final assay buffer for 30 min before adding the substrate to initiate the reaction catalyzed by M^{Pro}. The production format was monitored in a fluorescence plate reader with excitation at 336 nm and emission at 490 nm. We calculated the initial rate according to the fluorescent intensity in the first 5 min by linear regression, which was then normalized according to the initial rate of positive and negative controls. We calculated the initial rate according to the fluorescent intensity in the first 5 min by linear regression, which was then normalized according to the initial rate of positive and negative controls.

Cellular Potency Tests for Selected Inhibitors

HEK293T cells were maintained in high-glucose DMEM supplemented with GlutaMAX and 10% fetal bovine serum in 10 cm culture plates at 37 °C and 5% CO_2 until reaching 80%–90% confluency. Cells were transfected with the pLVX-MPro-eGFP-2 plasmid (*43*) using polyethyleneimine (30 μg/mL) and 8 μg of plasmid DNA in 500 μL opti-MEM per transfection. The transfection mixture was incubated with

cells overnight. On the following day, the medium was removed, and cells were washed with PBS before enzymatic detachment using 0.05% trypsin-EDTA. The dissociated cells were resuspended in their original growth medium, and the density was adjusted to 5×10^5 cells/mL. A volume of 500 µL of the cell suspension was seeded into each well of a 48-well plate, followed by the addition of 100 µL of drug solution prepared in growth media. Cells were incubated under the same culture conditions for 72 hours with varying concentrations of inhibitors before flow cytometry analysis. Post-incubation, cells were resuspended in 500 µL PBS and centrifuged at 800 rpm for 5 minutes. The supernatant was discarded, and cell pellets were resuspended in 200 µL PBS. eGFP fluorescence was analyzed using a Cytoflex Beckman Flow Cytometer, with cells sorted based on side scatter (SSC-A, SSC-H) and forward scatter (FSC-A). Gating was performed sequentially using SSC-A/FSC-A followed by SSC-A/SSC-H. eGFP fluorescence was excited using a 488 nm blue laser, and emissions were recorded at FITC-A (525 nm). All processed data were plotted and fitted to a four-parameter Hill equation using GraphPad Prism 9.0 to determine EC_{50} values.

Antiviral Potency Tests for Selected Inhibitors

A549-hACE2 cells (BEI Resources, NR53821) were cultured in a 37ºC incubator with 5% CO_2 in DMEM supplemented with 10% fetal bovine serum, 1× antibiotic/antimycotic and puromycin dihydrochloride. SARS-CoV-2 strain WA1 (BEI Resources; USA-WA1/2020; NR52281) was cultured on Vero E6-ACE2-TMPRSS2. Infectious titer was determined by tissue culture infectious dose 50% ($TCID_{50}$) on Vero E6-ACE2-TMPRSS2 cells. Cells were inoculated at a multiplicity of 0.1 $TCID_{50}$ unit per cell, incubated for 1h at 37ºC to allow for virus adsorption, rinsed three times with phosphate buffered saline pH 7 to remove unbound inoculum, and treated by addition of DMEM with 10% fetal bovine serum and 1× antibiotic/antimycotic containing various concentrations of potential antivirals. Small aliquots of medium were collected at 48h and 72 h after inoculation, and the amount of virus growth was titrated using a Luna One-Step RT175 qPCR (NEB, Ipswich, MA, US) with the N1 primer set targeting the nucleoprotein gene of the virus, calibrated to a standard curve of inactivated viral RNA (BEI Resources, NR-52285).

X-Ray Crystallography Analysis of M^{Pro}-Inhibitor Complexes

The production of crystals of M^{pro}-inhibitor complexes was following the previous protocols (*32*). Data were collected on a Bruker Photon II detector or the Advanced Light Source (ALS) beamline 5.0.2 using a Pilatus3 S 6M detector. The diffraction data were indexed, integrated and scaled with iMosflm or PROTEUM3. All the structures were determined by molecular replacement using the structure model of the free enzyme of the SARS-CoV-2 M^{pro} [Protein Data Bank (PDB) ID code 7JPY] as the search model using Phaser in the Phenix package. *JLigand* and *Sketcher* from the CCP4 suite were employed for the generation of PDB and geometric restraints for the inhibitors. The inhibitors were built into the Fo-Fc density by using *Coot*. Refinement of all the structures was performed with Real-space Refinement in Phenix. Details of data quality and structure refinement are summarized in Tables S1-S8.

Electrospray Ionization Mass Spectrometry (ESI-MS) Analysis of M^{Pro} Bound with VB-B-112

Purified M^{Pro} protein (10 µM) was incubated with VB-B-112 inhibitor (50 µM; 5-fold molar excess) in [10 mM sodium phosphate, 10 mM sodium chloride, and 0.5 mM EDTA, pH 7.6] for 30 mins at 37°C prior to analysis. Samples were analyzed by an Orbitrap Exploris™ 480 mass spectrometer (Thermo Fisher Scientific, Inc., Waltham, MA) coupled with an EASY-nLC 1000 system (Thermo Fisher Scientific, Inc., Waltham, MA). Protein–inhibitor sample were separated by a C4 column packed with butyl-bonded silica, using solvents A (0.1% formic acid in 2% acetonitrile, 98% water) and B (0.1%

formic acid in 100% acetonitrile), with a continuous gradient from 2 to 95% B over 25 min.

High-resolution Orbitrap mass spectrometry revealed a clear mass shift upon incubation of the protein with inhibitor. The deconvoluted spectrum of the apo protein displayed a dominant species at 33,855 Da, consistent with the calculated molecular weight of the unmodified protein.
Upon incubation with VB-B-112, a dominant species was observed at 34,098 Da, corresponding to a mass increase of 243 Da, in close agreement with the expected mass of the covalently bound inhibitor. [Figure S5]

2. Supplementary Figures (Figs. S1-S6)

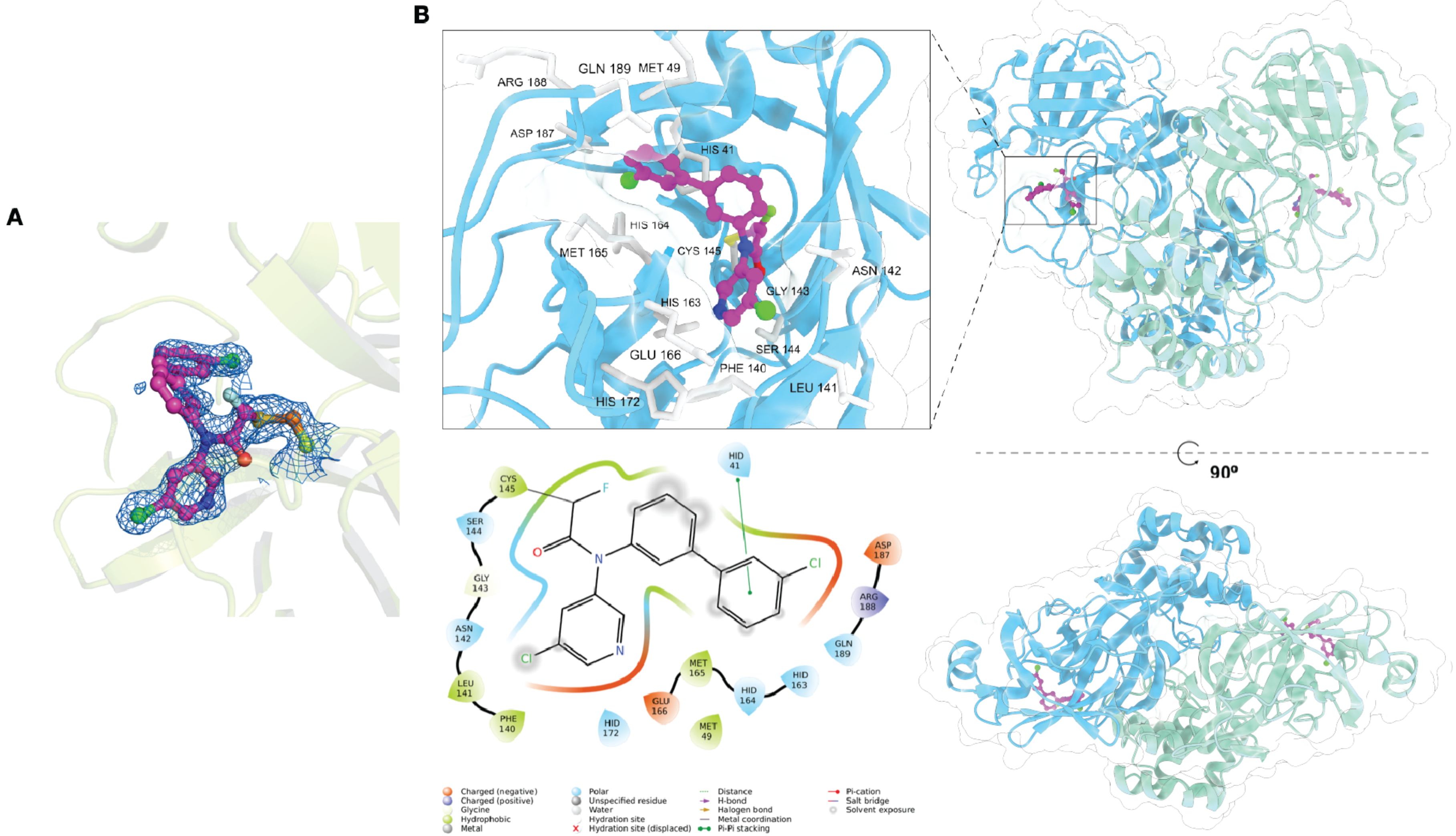

Figure S1. Crystal structure of M^{Pro} complexed with SR-B-7. (**A**) 2Fo-Fc electron density map around SR-B-7 and Cya145 side chain is contoured at the 1σ level and shown at the left side. Carbon atoms of SR-B-7 is colored in purple that is different from orange for a Cys145 side chain carbon. (**B**) An overall dimer structure of the complex viewed from two different angles, a zoomed-in active site showing SR-B-7 interactions with active site residues of M^{Pro}, and a 2D interaction map surrounding SR-B-7 at M^{Pro} active site.

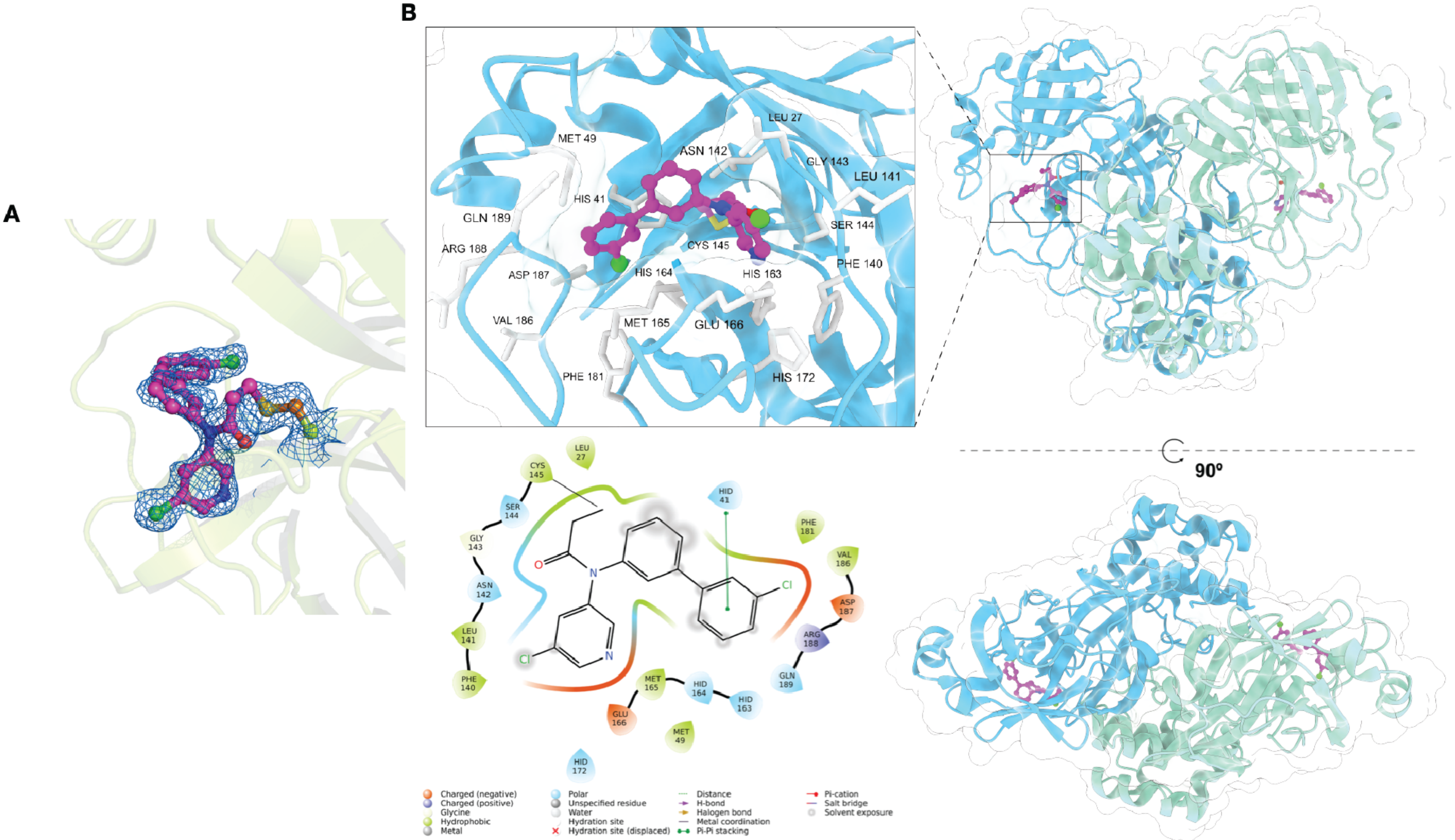

Figure S2. Crystal structure of M^{Pro} complexed with SR-B-13. (**A**) 2Fo-Fc electron density map around SR-B-13 and Cya145 side chain is contoured at the 1σ level and shown at the left side. Carbon atoms of SR-B-13 is colored in purple that is different from orange for a Cys145 side chain carbon. (**B**) An overall dimer structure of the complex viewed from two different angles, a zoomed-in active site showing SR-B-13 interactions with active site residues of M^{Pro}, and a 2D interaction map surrounding SR-B-13 at M^{Pro} active site.

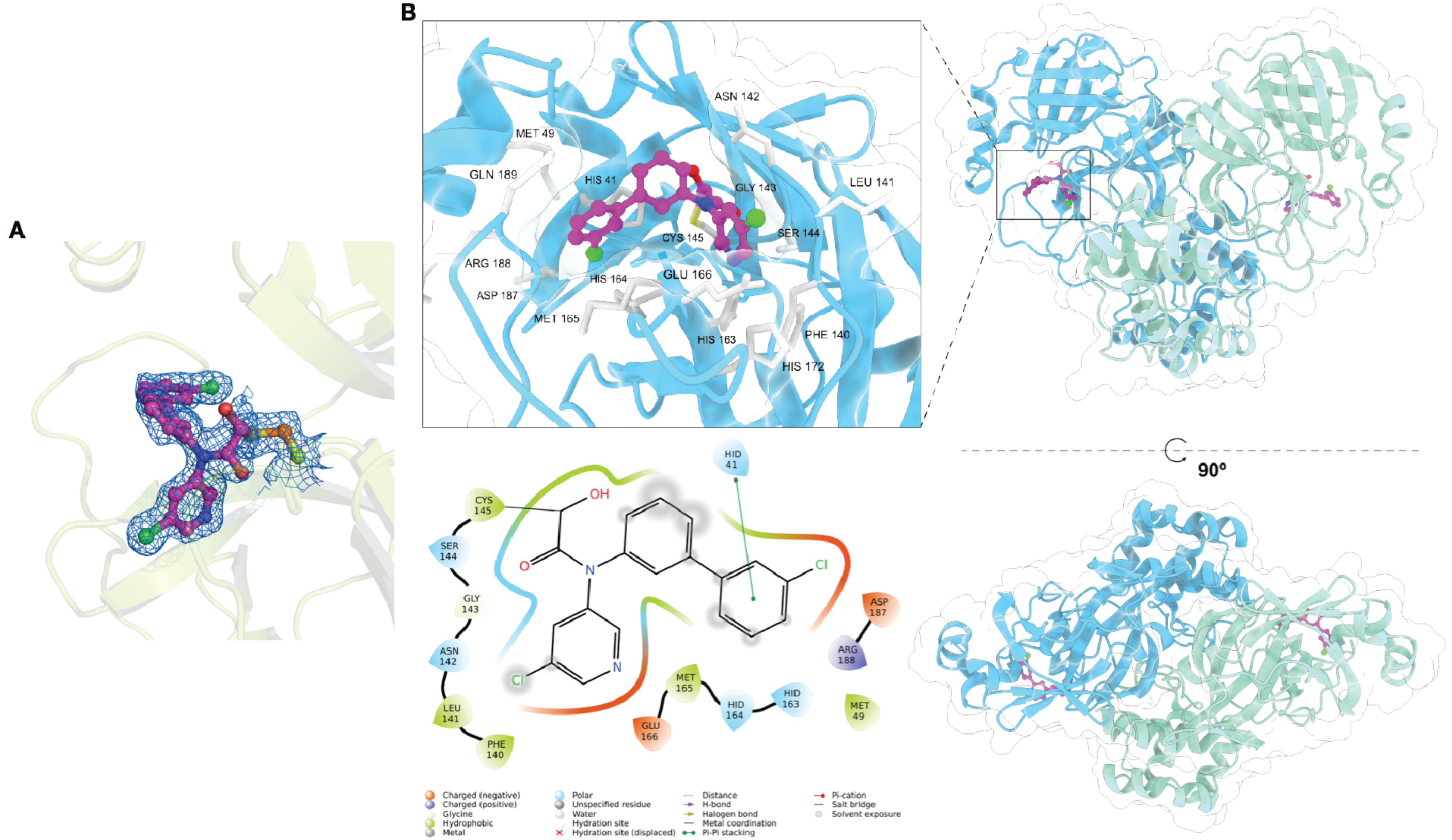

Figure S3. Crystal structure of M^{Pro} complexed with VB-C-70. (**A**) 2Fo-Fc electron density map around VB-C-70 and Cya145 side chain is contoured at the 1σ level and shown at the left side. Carbon atoms of VB-C-70 is colored in purple that is different from orange for a Cys145 side chain carbon. (**B**) An overall dimer structure of the complex viewed from two different angles, a zoomed-in active site showing VB-C-70 interactions with active site residues of M^{Pro}, and a 2D interaction map surrounding SR-B-7 at M^{Pro} active site.

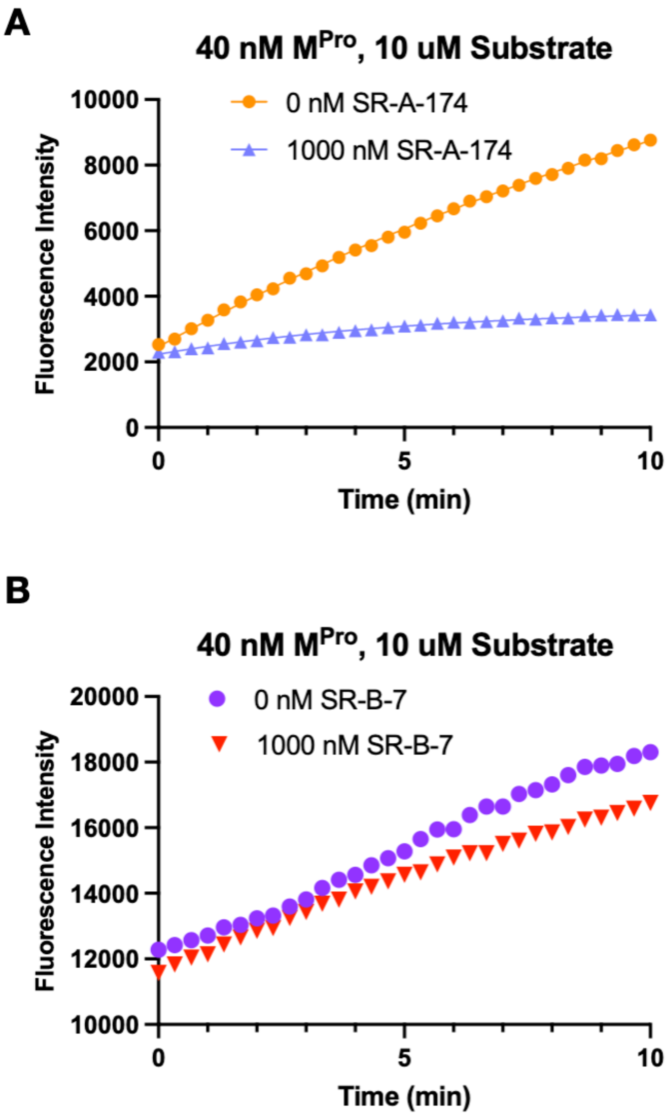

Figure S4. Inhibition kinetics of (**A**) SR-A-174 and (**B**) SR-B-7 on M^{Pro}. 40 nM M^{Pro} was mixed with 10 µM substrate with or without 1 µM SR-A-174 or SR-B-7 and measurement of the production formation was conducted immediately in a fluorescence plate reader for 10 minutes.

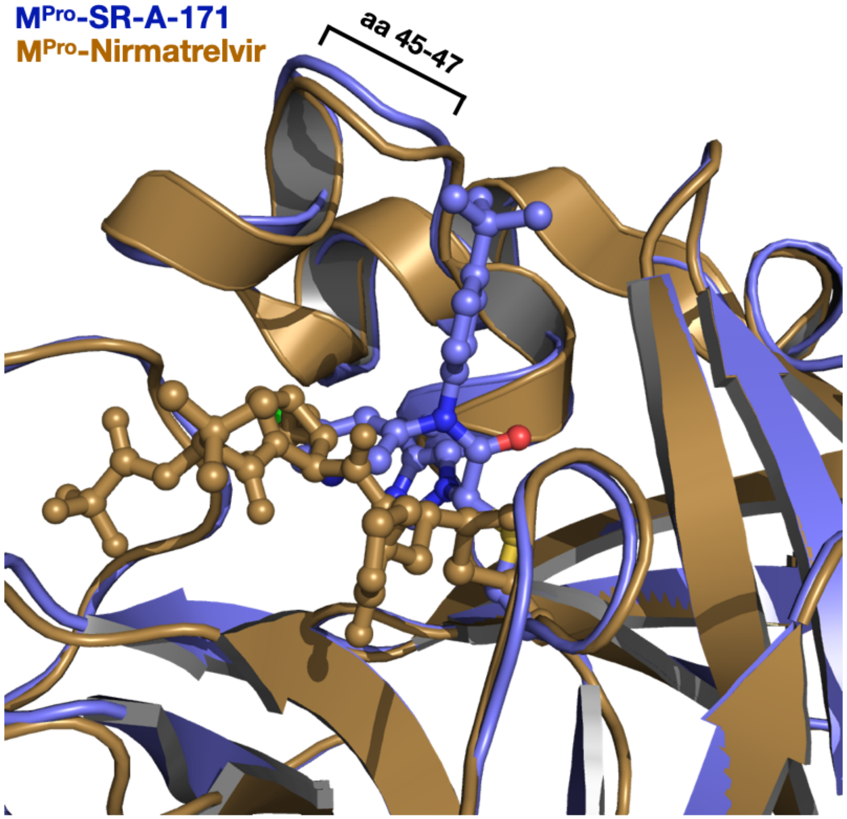

Figure S5. Structure comparison of M^{Pro}-SR-A-171 and M^{Pro}-nirmatrelvir. The Thr45-glu47 loop is indicated.

A

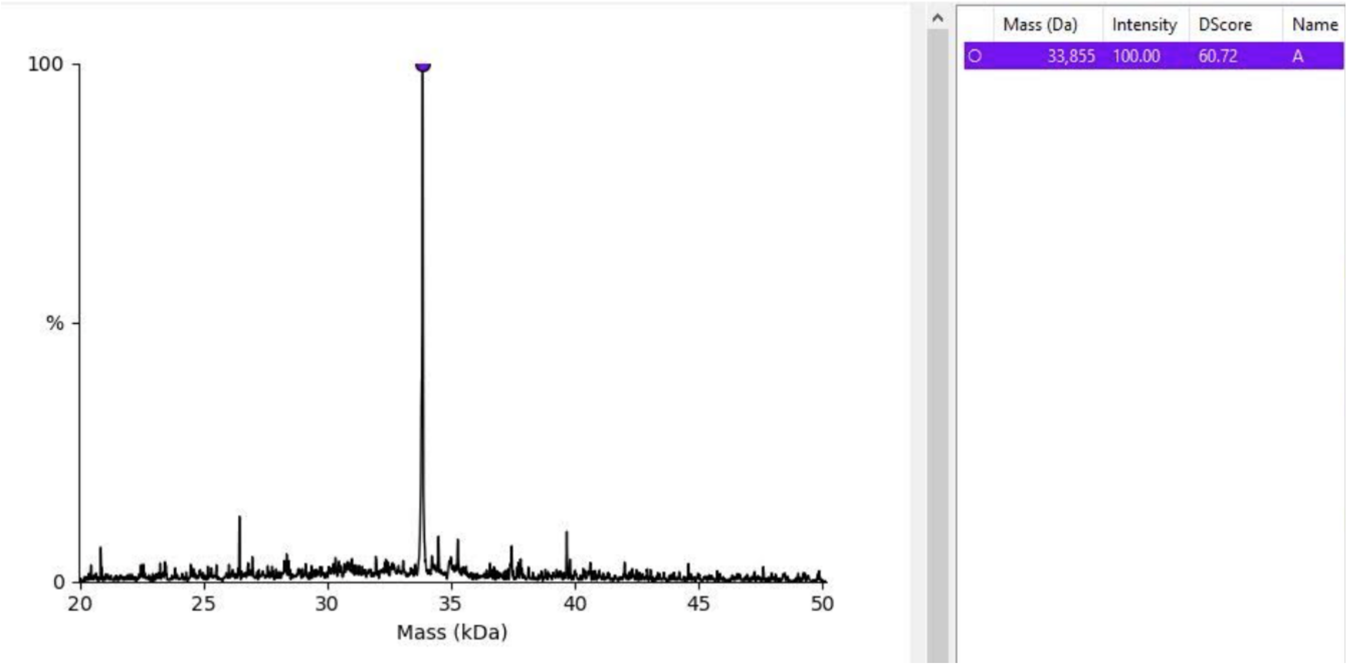

B

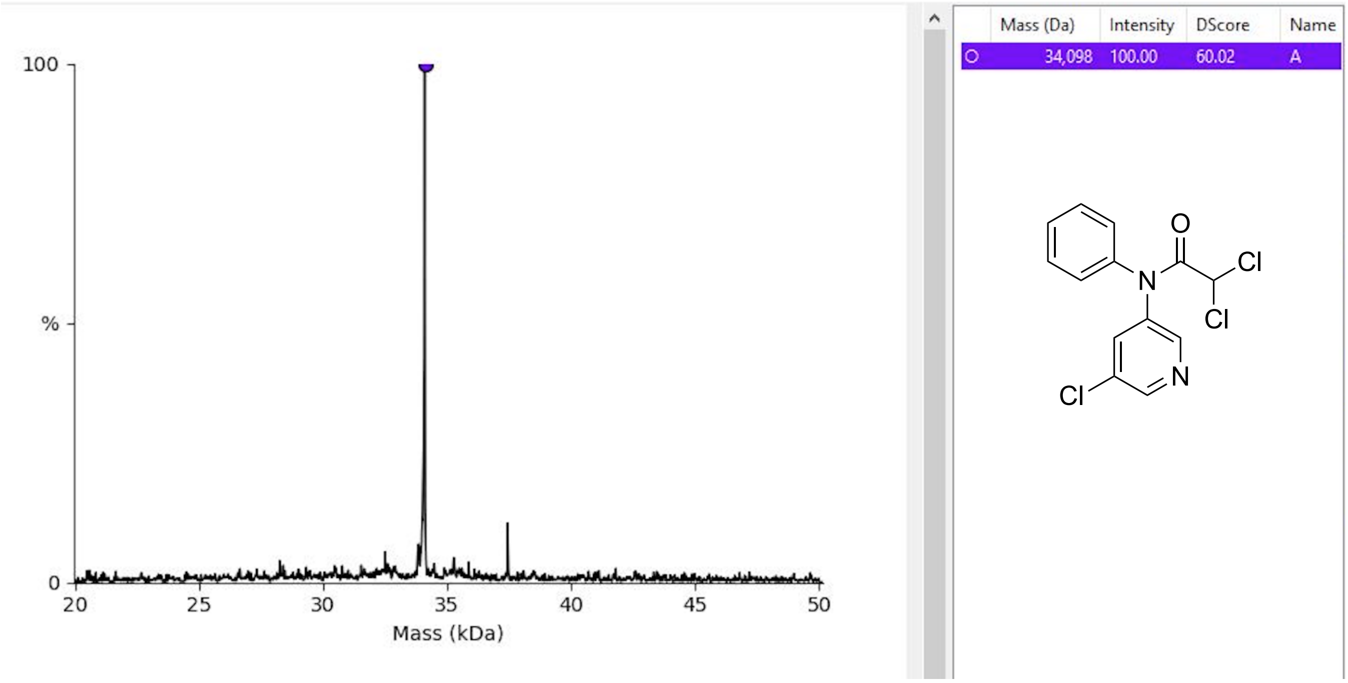

Fig. S6. Deconvoluted ESI-MS spectra of (**A**) M^{Pro} and (**B**) M^{Pro} bound with VB-B-112. The molecular weight difference between the two samples is 243 Da, matching exactly VB-B-112 with its two chlorides covalently replaced by His41 and Cys145.

3. Supplementary Tables (Table S1-S8)

Table S1. Data collection and refinement statistics for the M^{Pro}-VB-C-20 complex

PDB entry	9BSG
Resolution range (Å)	24.29 - 1.75 (1.813 - 1.75)
Space group	I 1 2 1
Unit cell	51.6423 81.3379 90.2308 90 96.6013 90
Total reflections	319066 (12439)
Unique reflections	35481 (3419)
Multiplicity	9.0 (3.6)
Completeness (%)	94.22 (91.52)
Mean I/sigma(I)	18.88 (3.14)
Wilson B-factor	15.57
R-merge	0.06357 (0.3998)
R-meas	0.06696 (0.4645)
R-pim	0.02038 (0.2292)
CC1/2	0.999 (0.935)
CC*	1 (0.983)
Reflections used in refinement	35229 (3410)
Reflections used for R-free	1817 (172)
R-work	0.2296 (0.3614)
R-free	0.2547 (0.3912)
CC(work)	0.953 (0.710)
CC(free)	0.941 (0.561)
Number of non-hydrogen atoms	2663
macromolecules	2367
ligands	22
solvent	274
Protein residues	306
RMS(bonds)	0.008
RMS(angles)	1.04
Ramachandran favored (%)	96.05
Ramachandran allowed (%)	3.95
Ramachandran outliers (%)	0.00
Rotamer outliers (%)	0.00
Clashscore	6.17
Average B-factor	25.48
macromolecules	24.84
ligands	31.82
solvent	30.51
Number of TLS groups	1

Table S2. Data collection and refinement statistics for the M^{Pro}-VB-C-68 complex

PDB entry	9BSP
Resolution range (Å)	24.29 - 1.6 (1.657 - 1.6)
Space group	I 1 2 1
Unit cell	51.6586 81.6651 90.1397 90 96.4132 90
Total reflections	448551 (17635)
Unique reflections	48841 (4752)
Multiplicity	9.2 (3.7)
Completeness (%)	98.81 (97.67)
Mean I/sigma(I)	15.40 (1.53)
Wilson B-factor	16.18
R-merge	0.07376 (1.224)
R-meas	0.07762 (1.414)
R-pim	0.02325 (0.6911)
CC1/2	0.999 (0.671)
CC*	1 (0.896)
Reflections used in refinement	48422 (4746)
Reflections used for R-free	2448 (240)
R-work	0.2094 (0.3143)
R-free	0.2360 (0.3262)
CC(work)	0.960 (0.734)
CC(free)	0.949 (0.676)
Number of non-hydrogen atoms	2713
macromolecules	2367
ligands	29
solvent	317
Protein residues	306
RMS(bonds)	0.008
RMS(angles)	0.96
Ramachandran favored (%)	97.37
Ramachandran allowed (%)	2.30
Ramachandran outliers (%)	0.33
Rotamer outliers (%)	0.00
Clashscore	2.97
Average B-factor	25.18
macromolecules	24.13
ligands	34.44
solvent	32.16
Number of TLS groups	1

Table S3. Data collection and refinement statistics for the M^{Pro}-SR-B-7 complex

PDB entry	9BSI
Resolution range (Å)	24.02 - 1.7 (1.761 - 1.7)
Space group	I 1 2 1
Unit cell	51.7178 81.53 90.2642 90 96.5794 90
Total reflections	228617 (12189)
Unique reflections	35107 (2575)
Multiplicity	6.5 (4.7)
Completeness (%)	85.01 (61.57)
Mean I/sigma(I)	12.94 (1.45)
Wilson B-factor	15.65
R-merge	0.07863 (1.042)
R-meas	0.08435 (1.163)
R-pim	0.02956 (0.5084)
CC1/2	0.998 (0.857)
CC*	1 (0.961)
Reflections used in refinement	34803 (2509)
Reflections used for R-free	1725 (122)
R-work	0.2283 (0.3447)
R-free	0.2477 (0.3847)
CC(work)	0.952 (0.677)
CC(free)	0.940 (0.655)
Number of non-hydrogen atoms	2668
macromolecules	2367
ligands	25
solvent	276
Protein residues	306
RMS(bonds)	.009
RMS(angles)	1.07
Ramachandran favored (%)	5.72
Ramachandran allowed (%)	4.28
Ramachandran outliers (%)	0.00
Rotamer outliers (%)	0.38
Clashscore	8.29
Average B-factor	3.34
macromolecules	2.76
ligands	0.06
solvent	7.66
Number of TLS groups	1

Table S4. Data collection and refinement statistics for the M^{Pro}-SR-B-13 complex

PDB entry	9BSO
Resolution range (Å)	24.22 - 1.7 (1.761 - 1.7)
Space group	I 1 2 1
Unit cell	51.7471 82.3596 89.9651 90 96.4583 90
Total reflections	199826 (14552)
Unique reflections	40208 (3874)
Multiplicity	5.0 (3.8)
Completeness (%)	97.44 (94.29)
Mean I/sigma(I)	10.68 (1.61)
Wilson B-factor	15.93
R-merge	0.07735 (0.7568)
R-meas	0.08457 (0.8761)
R-pim	0.03283 (0.4318)
CC1/2	0.998 (0.891)
CC*	0.999 (0.971)
Reflections used in refinement	40184 (3862)
Reflections used for R-free	2014 (210)
R-work	0.2478 (0.3784)
R-free	0.2703 (0.4348)
CC(work)	0.948 (0.693)
CC(free)	0.935 (0.576)
Number of non-hydrogen atoms	2633
macromolecules	2367
ligands	25
solvent	241
Protein residues	306
RMS(bonds)	0.009
RMS(angles)	1.05
Ramachandran favored (%)	97.04
Ramachandran allowed (%)	2.63
Ramachandran outliers (%)	0.33
Rotamer outliers (%)	0.00
Clashscore	8.29
Average B-factor	26.31
macromolecules	25.92
ligands	30.61
solvent	29.74
Number of TLS groups	1

Table S5. Data collection and refinement statistics for the M^{Pro}-VB-C-70 complex

PDB entry	9BSQ
Resolution range (Å)	24 - 1.8 (1.864 - 1.8)
Space group	I 1 2 1
Unit cell	51.0034 80.2229 89.1546 90 96.8538 90
Total reflections	215450 (10690)
Unique reflections	32145 (3051)
Multiplicity	6.7 (3.5)
Completeness (%)	96.74 (92.42)
Mean I/sigma(I)	11.81 (2.02)
Wilson B-factor	17.76
R-merge	0.08642 (0.6401)
R-meas	0.09277 (0.7463)
R-pim	0.03255 (0.3731)
CC1/2	0.998 (0.809)
CC*	0.999 (0.946)
Reflections used in refinement	32003 (3050)
Reflections used for R-free	1561 (141)
R-work	0.2269 (0.3066)
R-free	0.2474 (0.3262)
CC(work)	0.956 (0.739)
CC(free)	0.949 (0.578)
Number of non-hydrogen atoms	2626
macromolecules	2367
ligands	25
solvent	234
Protein residues	306
RMS(bonds)	0.009
RMS(angles)	1.10
Ramachandran favored (%)	97.37
Ramachandran allowed (%)	2.63
Ramachandran outliers (%)	0.00
Rotamer outliers (%)	0.38
Clashscore	5.74
Average B-factor	25.65
macromolecules	25.22
ligands	29.22
solvent	29.65
Number of TLS groups	1

Table S6. Data collection and refinement statistics for the M^{Pro}-YR-C-163 complex

PDB entry	9BTR
Resolution range (Å)	24.57 - 1.9 (1.968 - 1.9)
Space group	I 1 2 1
Unit cell	51.7274 80.8865 90.2992 90 96.9331 90
Total reflections	363780 (19405)
Unique reflections	29139 (2897)
Multiplicity	12.5 (6.7)
Completeness (%)	99.50 (99.97)
Mean I/sigma(I)	9.77 (1.26)
Wilson B-factor	17.56
R-merge	0.1802 (1.164)
R-meas	0.1877 (1.262)
R-pim	0.05087 (0.4827)
CC1/2	0.995 (0.731)
CC*	0.999 (0.919)
Reflections used in refinement	29007 (2896)
Reflections used for R-free	1399 (128)
R-work	0.2169 (0.2841)
R-free	0.2470 (0.3208)
CC(work)	0.951 (0.771)
CC(free)	0.922 (0.650)
Number of non-hydrogen atoms	2628
macromolecules	2367
ligands	29
solvent	232
Protein residues	306
RMS(bonds)	0.009
RMS(angles)	1.12
Ramachandran favored (%)	96.71
Ramachandran allowed (%)	2.96
Ramachandran outliers (%)	0.33
Rotamer outliers (%)	0.00
Clashscore	3.82
Average B-factor	24.82
macromolecules	24.26
ligands	31.94
solvent	29.56
Number of TLS groups	1

Table S7. Data collection and refinement statistics for the M^{Pro}-VB-B-112 complex

PDB entry	9BSA
Resolution range (Å)	24.11 - 1.7 (1.761 - 1.7)
Space group	I 1 2 1
Unit cell	51.7633 81.8669 90.358 90 96.3387 90
Total reflections	205118 (9154)
Unique reflections	34545 (2785)
Multiplicity	5.9 (3.3)
Completeness (%)	82.63 (65.98)
Mean I/sigma(I)	14.95 (2.29)
Wilson B-factor	16.28
R-merge	0.06485 (0.5061)
R-meas	0.07022 (0.6046)
R-pim	0.02613 (0.322)
CC1/2	0.998 (0.831)
CC*	1 (0.953)
Reflections used in refinement	34039 (2692)
Reflections used for R-free	1694 (141)
R-work	0.2329 (0.4185)
R-free	0.2496 (0.4394)
CC(work)	0.954 (0.697)
CC(free)	0.942 (0.626)
Number of non-hydrogen atoms	2639
macromolecules	2364
ligands	17
solvent	258
Protein residues	306
RMS(bonds)	0.009
RMS(angles)	1.01
Ramachandran favored (%)	97.04
Ramachandran allowed (%)	2.96
Ramachandran outliers (%)	0.00
Rotamer outliers (%)	0.00
Clashscore	3.20
Average B-factor	25.98
macromolecules	23.93
ligands	33.89
solvent	44.22
Number of TLS groups	1

Table S8. Data collection and refinement statistics for the M^{Pro}-SR-A-171 complex

PDB entry	9BSF
Resolution range (Å)	24.29 - 1.75 (1.813 - 1.75)
Space group	I 1 2 1
Unit cell	51.6438 81.3059 89.7181 90 96.8018 90
Total reflections	322136 (19406)
Unique reflections	37098 (3673)
Multiplicity	8.7 (5.3)
Completeness (%)	96.41 (96.94)
Mean I/sigma(I)	16.58 (1.54)
Wilson B-factor	20.29
R-merge	0.06833 (0.9098)
R-meas	0.07213 (1.007)
R-pim	0.02266 (0.4249)
CC1/2	0.999 (0.895)
CC*	1 (0.972)
Reflections used in refinement	35810 (3581)
Reflections used for R-free	1840 (185)
R-work	0.2042 (0.3100)
R-free	0.2362 (0.3365)
CC(work)	0.963 (0.851)
CC(free)	0.942 (0.790)
Number of non-hydrogen atoms	2685
macromolecules	2367
ligands	21
solvent	297
Protein residues	306
RMS(bonds)	0.009
RMS(angles)	1.02
Ramachandran favored (%)	97.37
Ramachandran allowed (%)	2.63
Ramachandran outliers (%)	0.00
Rotamer outliers (%)	0.38
Clashscore	4.47
Average B-factor	29.32
macromolecules	28.53
ligands	45.40
solvent	34.49
Number of TLS groups	1

4. Supplementary Synthesis Details

All reagents and solvents for synthesis were purchased from commercial sources and used without purification. All glassware was flame-dried prior to use. Thin layer chromatography (TLC) was carried out on aluminum plates coated with 60 F254 silica gel. TLC plates were visualized under UV light (254 or 365 nm) or stained with 5% phosphomolybdic acid. Normal phase column chromatography was carried out using a Yamazen Smart Flash AKROS system. Analytical reverse-phase high-pressure liquid chromatography (RP-HPLC) was carried out on a Shimadzu LC20 HPLC system with an analytical C_{18} column. The mobile phases were H_2O with 0.1% formic acid (A) and acetonitrile with 0.1% formic acid (B) if not mentioned otherwise. NMR spectra were recorded on a Bruker AVANCE Neo 400 MHz or Varian INOVA 300 MHz spectrometer in specified deuterated solvents. High-resolution electrospray ionization mass spectrometry (HRMS-ESI) was carried out on a Themo Scientific QExactive Focus system. Purities of assayed compounds were greater than 95% as determined by reverse-phase high performance liquid chromatography analysis.

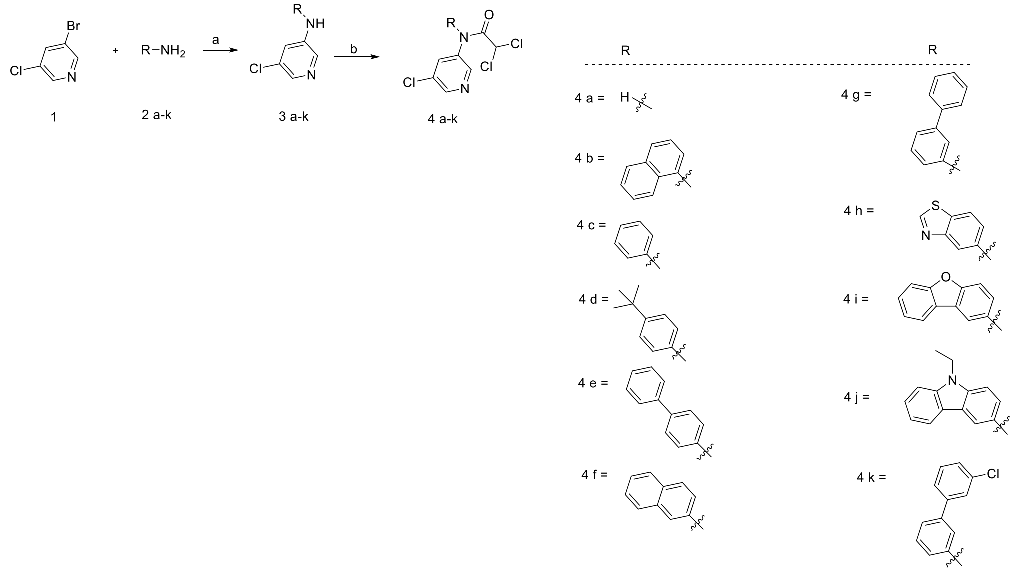

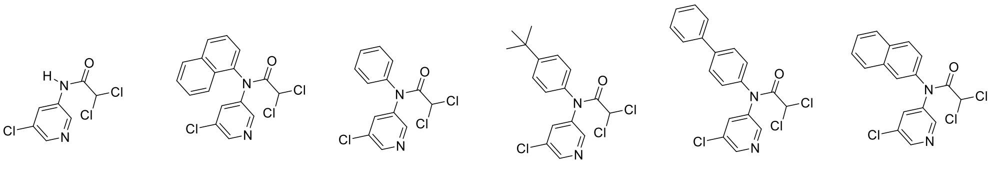

4a (VB-B-113) 4b (SR-A-178) 4c (VB-B-112) 4d (SR-A-171) 4e (VB-C-13) 4f YR-C-46)

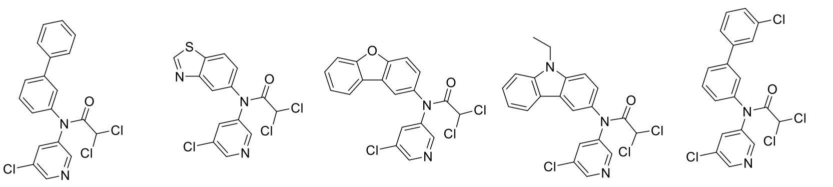

4g (YR-C-50) 4h (VB-C-48) 4i (VB-C-52) 4j (SR-B-33) 4k (SR-A-174)

Synthesis Scheme S1. The synthesis of VB-B-113 (4a), SR-A-178 (4b), VB-B-112 (4c), SR-A-171 (4d), VB-C-13 (9e), YR-C-46 (4f), YR-C-50 (4g), VB-C-48 (4h), VB-C-52 (4i), SR-B-33 (4j) and SR-A-74 (4k). Reagents and conditions: (a) CS_2CO_3, $Pd_2(dba)_3$, Dppf, Toluene, 100 °C, 24 h; (b) Py, DMAP, Dichloroacetyl chloride, 1,4-Dioxane, 60 °C, 1 h.

General procedure for Buchwald coupling (3b-k):

To a solution of R-NH_2 (0.5 mmol), 3-bromo-5-chloropyridine (0.75 mmol) in dry toluene was added CS_2CO_3 (3 eq, 1.5 mmol). The vessel was capped with a septum and degassed with nitrogen for 5 min. Then $Pd_2(dba)_3$ (0.05 equivalent, 0.025 mmol), DPPF (0.1 equivalent, 0.05 mmol) was added and again degas it for 5 min and heated at 100 ^{0}C for 24 hours. After the reaction is completed, filter the mixture on celite and wash with ethyl acetate (20 mL). Remove the mixture solvent under vacuum. Purify the residue by flash chromatography (0-100% EA in Hexane as the eluent) yielded resulting products in good yields.

General procedure for Amidation (4b-k):

To a stirred solution of 3b-k (1 eq) in 1,4-Dioxane was added Pyridine (2 eq), DMAP (0.1 eq) followed by Dichloroacetyl chloride (1.2 eq). The reaction mixture was stirred at 60 °C for 1 h. After completion of the reaction, the reaction mixture was concentrated and purified by flash chromatography (0-100% EA in Hexane as the eluent) to afford 4b-4k as a yellow gummy solid in good yields.

2,2-dichloro-*N*-(5-chloropyridin-3-yl)acetamide 4a (VB-B-113):

According to the general procedure for the synthesis of Amidation (4b-k), VB-B-113 compound was synthesized as a white solid (yield 63%).

^{1}H NMR (400 MHz, DMSO- *d6*) δ 11.11 (s, 1H), 8.68 (d, J = 2.2 Hz, 1H), 8.43 (d, J = 2.2 Hz, 1H), 8.22 (t, J = 2.2 Hz, 1H), 6.66 (s, 1H). ^{13}C NMR (101 MHz, DMSO-*d6*) δ 167.92, 149.01, 144.82, 140.49, 135.96, 131.52, 72.17. ESI-HRMS calculated for C7H6Cl3N2O (M+H+): 238.9546; found: 238.9603.

5-chloro-N-(naphthalen-1-yl)pyridin-3-amine (3b):

According to the general procedure for the synthesis of Buchwald coupling (3b-k), 3b compound was synthesized as a white solid (yield 85%).

^{1}H NMR (400 MHz, DMSO-*d6*) δ 8.73 – 8.65 (m, 1H), 8.26 (d, J = 2.3 Hz, 1H), 8.14 – 8.05 (m, 1H), 8.01 – 7.92 (m, 2H), 7.72 (t, J = 8.3 Hz, 1H), 7.55 (td, J = 5.4, 2.5 Hz, 2H), 7.52 – 7.42 (m, 2H), 7.23 (t, J = 2.2 Hz, 1H).^{13}C NMR (101 MHz, DMSO-*d6*) δ 143.94, 137.81, 137.29, 136.88, 134.89, 131.50, 128.83, 128.27, 126.87, 126.63, 126.31, 124.43, 123.07, 120.54, 118.05.

2,2-dichloro-N-(5-chloropyridin-3-yl)-N-(naphthalen-1-yl)acetamide 4b (SR-A-178):

According to the general procedure for the synthesis of Amidation (4b-k), SR-A-178 compound was synthesized as a white solid (yield 58%).

^{1}H NMR (400 MHz, $CDCl_3$) δ 8.35 (s, 1H), 8.32 (s, 1H), 7.99 (d, J = 7.3 Hz, 1H), 7.94 (dt, J = 6.9, 3.1 Hz, 1H), 7.82 (t, J = 2.1 Hz, 1H), 7.79 – 7.74 (m, 1H), 7.57 (dt, J = 10.9, 5.7 Hz, 4H), 5.69 (s, 1H). HRMS(ESI) calculated for C17H11Cl3N2O (M+H+): 364.9900; found: 364.9999.

2,2-dichloro-N-(5-chloropyridin-3-yl)-N-phenylacetamide 4c (VB-B-112):

According to the general procedure for the synthesis of Amidation (4b-k), VB-B-112 compound was

synthesized as a gummy solid (yield 61%).

^{1}H NMR (400 MHz, $CDCl_3$) δ 8.42 – 8.26 (m, 2H), 7.69 (t, J = 2.2 Hz, 1H), 7.48 (d, J = 6.7 Hz, 3H), 7.30 (d, J = 7.2 Hz, 2H), 5.93 (s, 1H). ^{13}C NMR (101 MHz, $CDCl_3$) δ 164.16, 146.45, 144.30, 139.07, 138.58, 132.46, 131.82, 130.94, 130.16, 128.52, 63.92. ESI-HRMS calculated for C13H10Cl3N2O (M+H+): 314.9859; found: 314.9845.

N-(4-(tert-butyl) phenyl)-5-chloropyridin-3-amine (3d):

According to the general procedure for the synthesis of Buchwald coupling (3b-k), 3d compound was synthesized as a white solid (yield 83%).

^{1}H NMR (400 MHz, DMSO-*d6*)) δ 8.54 (s, 1H), 8.23 (d, J = 2.4 Hz, 1H), 7.95 (d, J = 2.1 Hz, 1H), 7.38 (t, J = 2.3 Hz, 1H), 7.37 – 7.32 (m, 2H), 7.12 – 7.07 (m, 2H), 1.27 (s, 9H). 13C NMR (101 MHz, DMSO-*d6*)) δ 144.89, 142.51, 138.90, 137.66, 136.79, 131.54, 126.60, 119.80, 119.13, 34.42, 31.70.

N-(4-(tert-butyl) phenyl)-2-chloro-N-(5-chloropyridin-3-yl) acetamide 4d (SR-A-171):

According to the general procedure for the synthesis of Amidation (4b-k), SR-A-171 compound was synthesized as a white solid (yield 55%).

^{1}H NMR (400 MHz, $CDCl_3$) δ 8.35 (s, 1H), 8.28 (s, 1H), 7.71 (t, J = 2.0 Hz, 1H), 7.47 (d, J = 8.2 Hz, 2H), 7.20 (d, J = 7.4 Hz, 2H), 5.95 (s, 1H), 1.30 (s, 9H). HRMS(ESI) calculated for C17H17Cl3N2O (M+H+): 371.0400; found: 371.0470.

N-([1,1'-biphenyl]-4-yl)-5-chloropyridin-3-amine (3e):

According to the general procedure for the synthesis of Buchwald coupling (3b-k), 3e compound was synthesized as a white solid (yield 83%).

^{1}H NMR (400 MHz, DMSO-*d6*)) δ 8.77 (s, 1H), 8.32 (d, J = 2.4 Hz, 1H), 8.04 (d, J = 2.1 Hz, 1H), 7.69 – 7.61 (m, 4H), 7.54 – 7.40 (m, 3H), 7.37 – 7.21 (m, 3H). ^{13}C NMR (101 MHz, DMSO-*d6*) δ 141.73, 141.29, 140.23, 138.50, 137.49, 133.80, 131.58, 129.38, 128.15, 127.27, 126.51, 121.03, 118.94.

N-([1,1'-biphenyl]-4-yl)-2,2-dichloro-N-(5-chloropyridin-3-yl)acetamide 4e (VB-C-13):

According to the general procedure for the synthesis of Amidation (4b-k), VB-C-13 compound was synthesized as a gummy solid (yield 65%).

^{1}H NMR (400 MHz, DMSO-*d6*) δ 8.65 (s, 2H), 8.13 (d, J = 27.0 Hz, 1H), 7.85 – 7.64 (m, 5H), 7.54 – 7.30 (m, 4H), 6.70 (s, 1H). ESI-HRMS calculated for C19H14Cl3N2O (M+H+): 391.0172; found: 391.0160.

5-chloro-N-(naphthalen-2-yl)pyridin-3-amine (3f):

According to the general procedure for the synthesis of Buchwald coupling (3b-k), 3f compound was synthesized as a white solid (yield 88%).

^{1}H NMR (400 MHz, DMSO-*d6*) δ 8.86 (s, 1H), 8.40 (d, J = 2.4 Hz, 1H), 8.07 (d, J = 2.1 Hz, 1H), 7.87

(d, J = 8.8 Hz, 1H), 7.84 – 7.79 (m, 2H), 7.61 (d, J = 2.3 Hz, 1H), 7.59 (t, J = 2.3 Hz, 1H), 7.45 (ddd, J = 8.3, 6.8, 1.3 Hz, 1H), 7.38 – 7.30 (m, 2H).

2,2-dichloro-N-(5-chloropyridin-3-yl)-N-(naphthalen-2-yl)acetamide 4f (YR-C-46):

According to the general procedure for the synthesis of Amidation (4b-k), YR-C-46 compound was synthesized as a gummy solid (yield 65%).

^{1}H NMR (400 MHz, $CDCl_3$) δ 8.44 (s, 2H), 8.01 (d, J = 8.6 Hz, 1H), 7.92 (dt, J = 16.8, 3.7 Hz, 3H), 7.79 (t, J = 2.2 Hz, 1H), 7.63 (tt, J = 8.2, 3.9 Hz, 2H), 7.38 (dd, J = 8.7, 2.2 Hz, 1H), 6.06 (s, 1H). HRMS(ESI) calculated for C17H11Cl3N2O (M+H+): 364.9900; found: 364.9960.

N-([1,1'-biphenyl]-3-yl)-5-chloropyridin-3-amine (3g):

According to the general procedure for the synthesis of Buchwald coupling (3b-k), 3g compound was synthesized as a white solid (yield 88%).

^{1}H NMR (400 MHz, DMSO-*d6*) δ 8.74 (s, 1H), 8.33 (d, J = 2.4 Hz, 1H), 8.03 (d, J = 2.1 Hz, 1H), 7.64 (dd, J = 8.1, 1.4 Hz, 2H), 7.52 – 7.45 (m, 3H), 7.45 – 7.35 (m, 3H), 7.27 (ddd, J = 7.7, 1.8, 0.9 Hz, 1H), 7.18 (dd, J = 8.0, 2.2 Hz, 1H). ^{13}C NMR (101 MHz, DMSO-*d6*) δ 142.39, 142.09, 141.92, 140.57, 138.49, 137.37, 131.57, 130.58, 129.45, 128.10, 127.15, 121.12, 120.79, 117.63, 117.13.

N-([1,1'-biphenyl]-3-yl)-2,2-dichloro-N-(5-chloropyridin-3-yl)acetamide 4g (YR-C-50):

According to the general procedure for the synthesis of Amidation (4b-k), YR-C-50 compound was synthesized as a gummy solid (yield 61%).

^{1}H NMR (400 MHz, $CDCl_3$) δ 8.44 (d, J = 10.4 Hz, 2H), 7.80 (t, J = 2.2 Hz, 1H), 7.74 (s, 1H), 7.66 – 7.53 (m, 4H), 7.52 – 7.46 (m, 2H), 7.46 – 7.39 (m, 1H), 7.34 (d, J = 7.8 Hz, 1H), 6.09 (s, 1H). HRMS(ESI) calculated for C19H13Cl3N2O (M+H+): 391.0100; found: 391.0158.

N-(5-chloropyridin-3-yl)benzo[d]thiazol-5-amine (3h):

According to the general procedure for the synthesis of Buchwald coupling (3b-k), 3h compound was synthesized as a white solid (yield 85%).

^{1}H NMR (400 MHz, DMSO-*d6)* δ 9.19 (s, 1H), 8.87 (s, 1H), 8.35 (d, J = 2.4 Hz, 1H), 8.08 – 7.93 (m, 3H), 7.55 (t, J = 2.3 Hz, 1H), 7.31 (dd, J = 8.8, 2.3 Hz, 1H). ^{13}C NMR (101 MHz, DMSO-*d6*) δ 154.21, 148.86, 141.80, 139.77, 138.73, 137.48, 135.64, 131.64, 124.10, 121.11, 119.24, 110.47.

N-(benzo[d]thiazol-6-yl)-2,2-dichloro-N-(5-chloropyridin-3-yl)acetamide 4h (VB-C-48):

According to the general procedure for the synthesis of Amidation (4b-k), VB-C-48 compound was synthesized as a gummy solid (yield 55%).

^{1}H NMR (400 MHz, $CDCl_3$) δ 9.10 (s, 1H), 8.37 (d, J = 19.2 Hz, 2H), 8.27 – 8.16 (m, 1H), 7.95 (s, 1H), 7.70 (t, J = 2.2 Hz, 1H), 7.44 (d, J = 8.2 Hz, 1H), 5.95 (s, 1H). ^{13}C NMR (101 MHz, $CDCl_3$) δ 164.13, 156.98, 153.70, 146.64, 144.34, 138.58, 136.35, 135.78, 132.64, 132.02, 126.59, 125.77, 122.36, 63.91. ESI-HRMS calculated for C14H9Cl3N3OS (M+H+): 371.9532; found: 371.9525

5-chloro-N-(dibenzo[b,d]furan-3-yl)pyridin-3-amine (3i):

According to the general procedure for the synthesis of Buchwald coupling (3b-k), 3i compound was synthesized as a white solid (yield 88%).

^{1}H NMR (400 MHz, DMSO-*d6*) δ 8.90 (s, 1H), 8.31 (d, J = 2.4 Hz, 1H), 8.05 – 7.90 (m, 3H), 7.59 – 7.51 (m, 2H), 7.40 – 7.26 (m, 3H), 7.12 (dd, J = 8.4, 2.0 Hz, 1H). ^{13}C NMR (101 MHz, DMSO-*d6*) δ 157.18, 156.00, 142.07, 141.56, 138.97, 137.76, 131.64, 126.69, 124.32, 123.55, 122.32, 121.51, 120.65, 117.94, 114.84, 111.76, 100.84.

2,2-dichloro-N-(5-chloropyridin-3-yl)-N-(dibenzo[b,d]furan-3-yl)acetamide 4i (VB-C-52):

According to the general procedure for the synthesis of Amidation (4b-k), VB-C-52 compound was synthesized as a gummy solid (yield 59%).

^{1}H NMR (400 MHz, $CDCl_3$) δ 8.37 (s, 2H), 8.01 (d, J = 8.1 Hz, 1H), 7.93 (d, J = 7.7 Hz, 1H), 7.71 (t, J = 2.2 Hz, 1H), 7.62 – 7.45 (m, 3H), 7.39 – 7.31 (m, 1H), 7.31 – 7.21 (m, 1H), 6.00 (s, 1H). ^{13}C NMR (101 MHz, $CDCl_3$) δ 164.25, 157.30, 156.41, 138.72, 128.73, 123.70, 122.77, 121.21, 112.11, 63.99. ESI-HRMS calculated for C19H12Cl3N2O (M+H+): 404.9964; found: 404.9954.

3-((5-chloropyridin-3-yl)-l2-azaneyl)-9-ethyl-9H-carbazole (3j):

According to the general procedure for the synthesis of Buchwald coupling (3b-k), 3j compound was synthesized as a white solid (yield 86%).

^{1}H NMR (400 MHz, $CDCl_3$) δ 8.06 (d, J = 2.5 Hz, 1H), 7.97 (d, J = 7.8 Hz, 1H), 7.91 (t, J = 5.1 Hz, 1H), 7.83 (d, J = 2.1 Hz, 1H), 7.45 – 7.39 (m, 1H), 7.34 (dd, J = 8.4, 6.0 Hz, 2H), 7.21 (dd, J = 8.6, 2.1 Hz, 1H), 7.18 – 7.13 (m, 1H), 7.07 (t, J = 2.3 Hz, 1H), 5.75 (s, 1H), 4.31 (q, J = 7.2 Hz, 2H), 1.38 (t, J = 7.2 Hz, 3H).

2,2-dichloro-N-(5-chloropyridin-3-yl)-N-(9-ethyl-9H-carbazol-3-yl) acetamide 4j (SR-B-33):

According to the general procedure for the synthesis of Amidation (4b-k), SR-B-33 compound was synthesized as a gummy solid (yield 63%).

^{1}H NMR (400 MHz, $CDCl_3$) δ 8.42 (s, 1H), 8.32 (s, 1H), 8.00 (dd, J = 10.7, 4.8 Hz, 2H), 7.76 (t, J = 2.2 Hz, 1H), 7.52 – 7.39 (m, 3H), 7.35 – 7.30 (m, 1H), 7.23 (t, J = 7.4 Hz, 1H), 6.02 (d, J = 5.7 Hz, 1H), 4.44 – 4.27 (m, 2H), 1.43 (t, J = 7.2 Hz, 3H).^{13}C NMR (101 MHz, $CDCl_3$) δ 164.77, 145.90, 144.08, 140.84, 139.82, 139.40, 131.98, 131.66, 129.74, 127.29, 125.30, 124.28, 121.98, 120.89, 120.56, 120.02, 110.28, 109.20, 64.18, 37.96, 13.89. HRMS(ESI) calculated for C21H16Cl3N3O (M+H+): 432.0400; found: 432.0421.

5-chloro-N-(3'-chloro-[1,1'-biphenyl]-3-yl)pyridin-3-amine (3k):

According to the general procedure for the synthesis of Buchwald coupling (3b-k), 3k compound was synthesized as a white solid (yield 90%).

^{1}H NMR (400 MHz, DMSO-*d6*) δ 8.75 (s, 1H), 8.34 (d, J = 2.4 Hz, 1H), 8.03 (d, J = 2.1 Hz, 1H), 7.68

(t, J = 1.8 Hz, 1H), 7.62 – 7.58 (m, 1H), 7.52 – 7.47 (m, 2H), 7.46 – 7.37 (m, 3H), 7.30 – 7.25 (m, 1H), 7.23 – 7.18 (m, 1H). ^{13}C NMR (101 MHz, DMSO-*d6*) δ 142.75, 142.52, 141.80, 140.50, 138.60, 137.41, 134.18, 131.58, 131.24, 130.67, 127.90, 126.92, 125.87, 121.24, 120.79, 118.17, 117.16.

2,2-dichloro-N-(3'-chloro-[1,1'-biphenyl]-3-yl)-N-(5-chloropyridin-3-yl)acetamide 4k (SR-A-174):

According to the general procedure for the synthesis of Amidation (4b-k), SR-A-174 compound was synthesized as a gummy solid (yield 66%).

^{1}H NMR (400 MHz, $CDCl_3$) δ 8.36 (d, J = 19.0 Hz, 2H), 7.72 (t, J = 2.0 Hz, 1H), 7.64 (s, 1H), 7.56 (s, 1H), 7.48 (s, 2H), 7.41 – 7.28 (m, 4H), 5.99 (s, 1H). HRMS(ESI) calculated for C17H17Cl3N2O (M+H+): 424.9700; found: 424.9771.

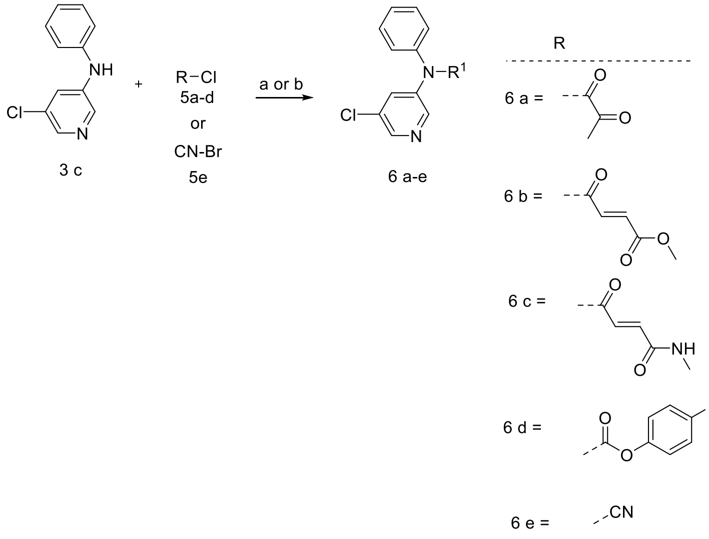

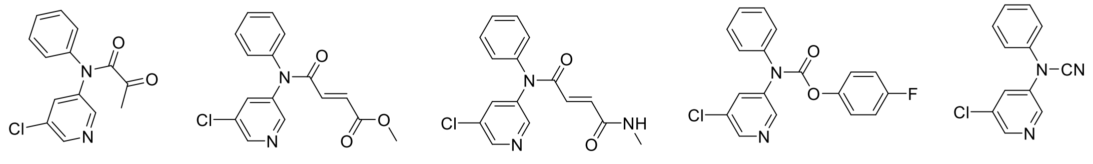

6a = SR-A-188 **6b = VB-C-20** **6c = VB-C-25** **6d = VB-C-30** **6e = VB-C-62**

Synthesis Scheme S2. The synthesis of SR-A-188 (6a), VB-C-20 (6b), VB-C-25 (6c), VB-C-30 (6d), VB-C-62 (6e). Reagents and conditions: (a) Py, DMAP, 5a-d, 1,4-Dioxane, 60 °C, 1 h; (b) 5e, LDA, THF, 0 °C to rt, 3 h.

N-(5-chloropyridin-3-yl)-2-oxo-N-phenylpropanamide 6a (SR-A-188):

According to the general procedure for the synthesis of Amidation, SR-A-188 compound was synthesized as a gummy solid (yield 70%).

^{1}H NMR (400 MHz, $CDCl_3$) δ 8.39 (s, 1H), 8.30 (s, 1H), 7.64 (s, 1H), 7.42 – 7.34 (m, 3H), 7.17 – 7.13 (m, 2H), 2.29 (s, 3H). HRMS(ESI) calculated for C14H11ClN2O2 (M+H+): 275.0500; found: 275.0575

Methyl (E)-4-((5-chloropyridin-3-yl)(phenyl)amino)-4-oxobut-2-enoate (VB-C-20):

According to the general procedure for the synthesis of Amidation, VB-C-20 compound was synthesized as a gummy solid (yield 69%).

$_1$H NMR (400 MHz, $CDCl_3$) δ 8.42 – 8.23 (m, 2H), 7.63 (s, 1H), 7.41 (dq, J = 11.7, 6.6 Hz, 3H), 7.19 – 7.12 (m, 2H), 6.98 – 6.75 (m, 3H), 3.67 (s, 3H). ^{13}C NMR (101 MHz, $CDCl_3$) δ 167.33, 165.57, 165.48, 164.18, 139.29, 134.07, 133.90, 133.87, 132.53, 131.85, 130.50, 129.25, 128.41, 52.28. ESI-HRMS calculated for C16H14ClN2O3 (M+H+): 317.0693; found: 317.0680.

N1-(5-chloropyridin-3-yl)-N4-methyl-N1-phenylfumaramide (VB-C-25):

According to the general procedure for the synthesis of Amidation **,** VB-C-25 compound was synthesized as a gummy solid (yield 62%).

^{1}H NMR (400 MHz, DMSO-*d6*) δ 8.54 (d, J = 15.5 Hz, 1H), 8.46 (d, J = 5.0 Hz, 1H), 8.03 (s, 1H), 7.50 (d, J = 15.8 Hz, 5H), 6.98 (d, J = 15.0 Hz, 1H), 6.62 (d, J = 15.0 Hz, 1H), 2.65 (d, J = 4.7 Hz, 3H). ^{13}C NMR (101 MHz, $CDCl_3$) δ 167.33, 165.57, 165.48, 164.18, 139.29, 134.07, 133.90, 133.87, 132.53, 131.85, 130.50, 129.25, 128.41, 42.37. ESI-HRMS calculated for C16H15ClN3O3 (M+H+): 316.0853; found: 316.0838.

N-(5-chloropyridin-3-yl)-N-phenylcyanamide (VB-C-62):

According to the general procedure for the synthesis of Amidation, VB-C-62 compound was synthesized as a gummy solid (yield 55%).

^{1}H NMR (400 MHz, $CDCl_3$) δ 8.35 (dd, J = 9.6, 2.2 Hz, 2H), 7.49 – 7.40 (m, 3H), 7.38 – 7.31 (m, 1H), 7.28 – 7.23 (m, 2H). ^{13}C NMR (101 MHz, $CDCl_3$) δ 145.51, 138.91, 137.23, 137.17, 132.56, 130.60, 128.32, 126.36, 123.17, 110.98. ESI-HRMS calculated for C12H9ClN3 (M+H+): 230.0485; found: 230.0473.

4-fluorophenyl (5-chloropyridin-3-yl)(phenyl)carbamate (VB-C-30):

According to the general procedure for the synthesis of Amidation VB-C-30 compound was synthesized as a gummy solid (yield 67%).

^{1}H NMR (400 MHz, $CDCl_3$) δ 8.39 (d, J = 2.3 Hz, 1H), 8.34 (d, J = 2.1 Hz, 1H), 7.69 (t, J = 2.3 Hz, 1H), 7.42 – 7.35 (m, 2H), 7.31 – 7.23 (m, 3H), 7.07 – 6.95 (m, 4H). ^{13}C NMR (101 MHz, $CDCl_3$) δ 161.51, 159.08, 152.62, 146.55, 146.52, 145.65, 140.47, 139.48, 131.66, 129.79, 127.98, 127.55, 122.85, 122.76, 116.19, 115.96. ESI-HRMS calculated for C18H13ClFN2O2 (M+H+): 343.0650; found: 343.0639.

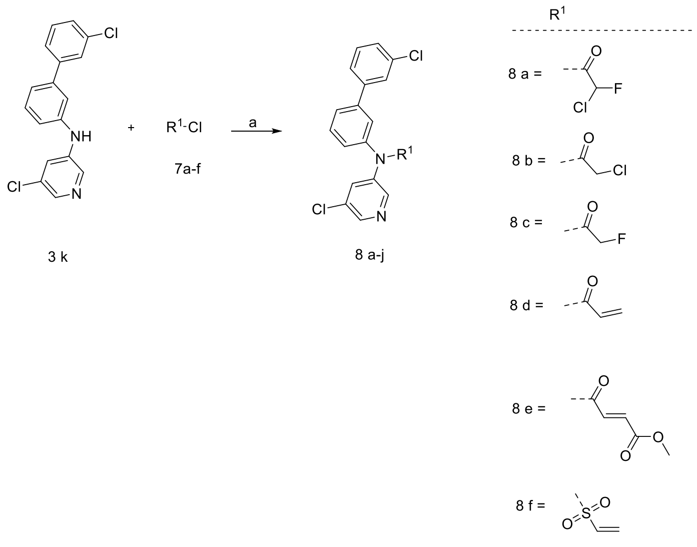

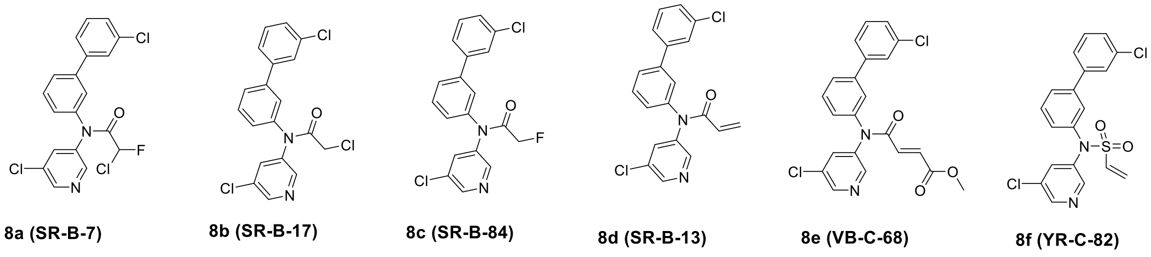

Synthesis Scheme S3. The synthesis of SR-B-7 (8a), SR-B-17 (8b), SR-B-84 (8c), SR-B-13 (8d), VB-C-68 (8e), YR-C-82 (8f). Reagents and conditions: (a) Py, DMAP, 7a-f, 1,4-Dioxane, 60 °C, 1 h.

2-chloro-N-(3'-chloro-[1,1'-biphenyl]-3-yl)-N-(5-chloropyridin-3-yl)-2-fluoroacetamide 8a (SR-B-7):

According to the general procedure for the synthesis of Amidation**,** SR-B-7 compound was synthesized as a gummy solid (yield 51%).

^{1}H NMR (400 MHz, $CDCl_3$) δ 8.39 (d, J = 7.2 Hz, 2H), 7.71 (d, J = 20.8 Hz, 1H), 7.56 (dd, J = 21.7, 14.3 Hz, 2H), 7.47 (s, 2H), 7.41 – 7.24 (m, 4H), 6.28 (d, J = 50.1 Hz, 1H). HRMS(ESI) calculated for C19H12Cl3FN2O (M+H+): 409.00; found: 409.0065.

2-chloro-N-(3'-chloro-[1,1'-biphenyl]-3-yl)-N-(5-chloropyridin-3-yl)acetamide 8b (SR-B-17):

According to the general procedure for the synthesis of Amidation**,** SR-B-17 compound was synthesized as a gummy solid (yield 54%).

^{1}H NMR (400 MHz, $CDCl_3$) δ 8.36 (s, 2H), 7.71 (dd, J = 11.2, 9.3 Hz, 1H), 7.59 (d, J = 7.0 Hz, 1H), 7.52 (t, J = 7.7 Hz, 1H), 7.46 (d, J = 12.5 Hz, 2H), 7.39 – 7.33 (m, 2H), 7.33 – 7.25 (m, 2H), 3.99 (s, 2H). HRMS(ESI) calculated for C19H13Cl3N2O (M+H+): 391.0100; found: 391.0160.

N-(3'-chloro-[1,1'-biphenyl]-3-yl)-N-(5-chloropyridin-3-yl)-2-fluoroacetamide 8c (SR-B-84):

According to the general procedure for the synthesis of Amidation**,** SR-B-84 compound was synthesized

as a gummy solid (yield 59%).

^{1}H NMR (400 MHz, $CDCl_3$) δ 8.37 (d, J = 4.1 Hz, 2H), 7.77 (s, 1H), 7.61 (d, J = 7.8 Hz, 1H), 7.54 (t, J = 7.8 Hz, 1H), 7.49 – 7.45 (m, 1H), 7.39 (dd, J = 6.3, 4.4 Hz, 1H), 7.36 (dt, J = 4.7, 1.6 Hz, 1H), 7.35 – 7.30 (m, 2H), 7.23 (d, J = 7.8 Hz, 1H), 4.77 (d, J = 46.9 Hz, 2H). HRMS(ESI) calculated for C19H13Cl2FN2O (M+H+): 375.0400; found: 375.0456.

N-(3'-chloro-[1,1'-biphenyl]-3-yl)-N-(5-chloropyridin-3-yl)acrylamide 8d (SR-B-13):

According to the general procedure for the synthesis of Amidation**,** SR-B-13 compound was synthesized as a white solid (yield 70%).

^{1}H NMR (400 MHz, $CDCl_3$) δ 8.35 (t, J = 3.6 Hz, 1H), 8.33 (d, J = 2.1 Hz, 1H), 7.66 (s, 1H), 7.55 – 7.46 (m, 3H), 7.38 – 7.28 (m, 4H), 7.18 – 7.14 (m, 1H), 6.48 (dd, J = 16.7, 1.6 Hz, 1H), 6.13 (dd, J = 16.7, 10.3 Hz, 1H), 5.68 (dd, J = 10.3, 1.6 Hz, 1H). HRMS(ESI) calculated for C20H14Cl2N2O (M+H+): 369.05; found: 369.0552.

Methyl (E)-4-((3'-chloro-[1,1'-biphenyl]-3-yl)(5-chloropyridin-3-yl)amino)-4-oxobut-2-enoate 8f (VB-C-68):

According to the general procedure for the synthesis of Amidation VB-C-68 compound was synthesized as a white solid (yield 70%).

^{1}H NMR (400 MHz, DMSO-*d6*) δ 8.59 (d, J = 13.4 Hz, 2H), 8.11 (s, 1H), 8.05 – 7.60 (m, 5H), 7.60 – 7.45 (m, 3H), 6.86 (d, J = 15.4 Hz, 1H), 6.76 (d, J = 15.4 Hz, 1H), 3.67 (s, 3H). ESI-HRMS calculated for C22H17Cl2N2O3 (M+H+): 427.0616; found: 427.0604.

N-(3'-chloro-[1,1'-biphenyl]-3-yl)-N-(5-chloropyridin-3-yl)ethenesulfonamide 8g (YR-C-82):

According to the general procedure for the synthesis of Amidation YR-C-82 compound was synthesized as a gummy solid (yield 59%).

^{1}H NMR (400 MHz, $CDCl_3$) δ 8.44 – 8.36 (m, 2H), 7.73 (t, J = 2.0 Hz, 1H), 7.56 – 7.37 (m, 4H), 7.41 – 7.28 (m, 4H), 6.73 (dd, J = 16.5, 9.8 Hz, 1H), 6.30 (d, J = 16.4 Hz, 1H), 6.09 (d, J = 9.8 Hz, 1H). HRMS(ESI) calculated for C19H14Cl2N2O2S (M+H+): 405.0200; found: 405.0217.

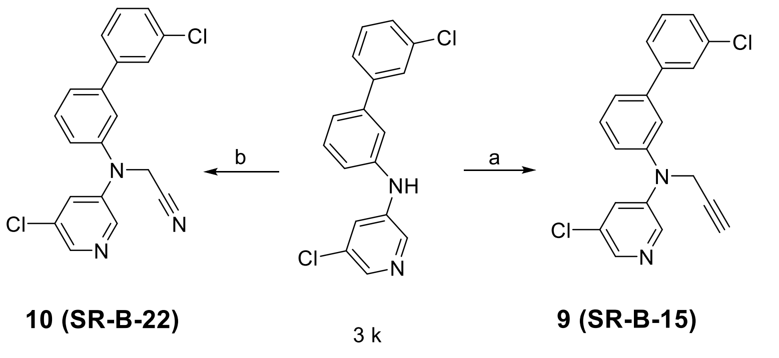

10 (SR-B-22) **9 (SR-B-15)**

Synthesis Scheme S4. The synthesis of SR-B-15 (9) & SR-B-22 (10). Reagents and conditions: (a) Propargyl chloride, NaH, DMF, 0 °C to rt, 3 h; (b) Bromoacetonitrile, NaH, DMF, 0 °C to rt, 3 h.

5-chloro-N-(3'-chloro-[1,1'-biphenyl]-3-yl)-N-(prop-2-yn-1-yl)pyridin-3-amine 9 (SR-B-15):

To a stirred solution of 3k (0.1 g, 0.28 mmol) in anhydrous DMF (5 mL) at 0 °C was added NaH (0.42 mmol, 17 mg, 60%). Stir for 15 minutes. Then Propargyl chloride (31.7 uL, 0.42 mmol) was added to reaction as drop wise at 0 °C. Raise the temperature slowly to room temperature and stir for 3h. The mixture was then poured into water (10 mL) and extracted with EtOAc (2×10 mL). The organic layer was dried over anhydrous Na2SO4 and then concentrated on vacuo. The residue was then purified with flash chromatography (0-100% EtOAc in Hexanes as the eluent) to afford SR-B-15 as a white solid (90 mg, 90%).

^{1}H NMR (400 MHz, $CDCl_3$) δ 8.12 (d, *J* = 2.5 Hz, 1H), 8.04 (d, *J* = 1.9 Hz, 1H), 7.48 (t, *J* = 1.6 Hz, 1H), 7.43 (dd, *J* = 12.7, 5.0 Hz, 1H), 7.39 – 7.29 (m, 4H), 7.29 – 7.25 (m, 1H), 7.19 – 7.14 (m, 2H), 4.37 (d, *J* = 2.4 Hz, 2H), 2.27 (t, *J* = 2.4 Hz, 1H). HRMS(ESI) calculated for C20H14Cl2N2 (M+H^+): 353.05; found: 353.0598.

2-((3'-chloro-[1,1'-biphenyl]-3-yl)(5-chloropyridin-3-yl)amino)acetonitrile 10 (SR-B-22):

To a stirred solution of 3k (0.1 g, 0.28 mmol) in anhydrous DMF (5 mL) at 0 °C was added NaH (0.42 mmol, 17 mg, 60%). Stir for 15 minutes. Then Bromoacetonitrile (29.7 uL, 0.42 mmol) was added to reaction as drop wise at 0 °C. Raise the temperature slowly to room temperature and stir for 3h. The mixture was then poured into water (10 mL) and extracted with EtOAc (2×10 mL). The organic layer was dried over anhydrous Na2SO4 and then concentrated on vacuo. The residue was then purified with flash chromatography (0-100% EtOAc in Hexanes as the eluent) to afford SR-B-22 as a white solid (85 mg, 85%).

^{1}H NMR (400 MHz, $CDCl_3$) δ 8.12 (d, J = 1.9 Hz, 1H), 8.09 (d, J = 2.5 Hz, 1H), 7.47 – 7.39 (m, 3H), 7.34 (dt, J = 7.3, 1.6 Hz, 1H), 7.31 – 7.23 (m, 3H), 7.15 – 7.10 (m, 2H), 4.48 (s, 2H). HRMS(ESI) calculated for C19H13Cl2N3 (M+H+): 354.05; found: 354.0554.

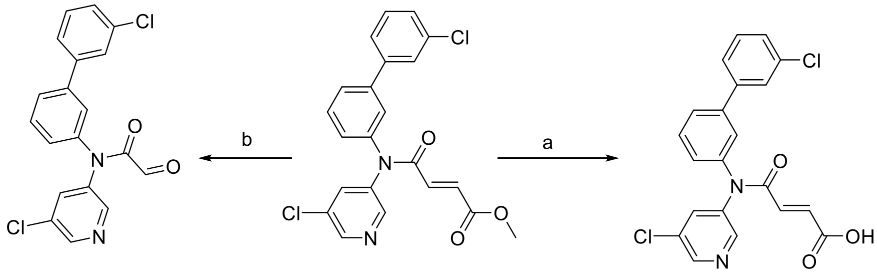

12 (VB-C-70) 8f **11 (VB-C-69)**

Synthesis Scheme S5. The synthesis of VB-C-69 (11) & VB-C-70 (12). Reagents and conditions: (a) $LiOH.H_2O$, $THF:H_2O$, 0 °C to rt, 16 h; (b) O_3, DCM, DMS, -78 °C to rt 12h.

(E)-4-((3'-chloro-[1,1'-biphenyl]-3-yl)(5-chloropyridin-3-yl)amino)-4-oxobut-2-enoic acid 11 (VB-C-69):

To a stirred solution of 8f (0.1 g, 0.23 mmol) in anhydrous $THF:H_2O$ (5 mL) at 0 °C was added $LiOH.H_2O$ (0.35 mmol, 15 mg). Stir at rt for 16h. After completion of the reaction, evaporate the solvent under reduced pressure and acidified with 1N HCl, extracted with water (10 mL) and EtOAc (2×10 mL). The organic layer was dried over anhydrous Na_2SO4 and then concentrated on vacuo to

afford VB-C-69 as a white solid (90 mg, 87%).

^{1}H NMR (400 MHz, DMSO-*d6)* δ 13.07 (s, 1H), 8.59 (d, J = 14.6 Hz, 2H), 8.10 (s, 1H), 7.88 (d, J = 46.3 Hz, 3H), 7.69 (dd, J = 11.6, 6.8 Hz, 1H), 7.65 – 7.58 (m, 1H), 7.55 – 7.45 (m, 3H), 6.79 (d, J = 15.3 Hz, 1H), 6.69 (d, J = 15.3 Hz, 1H). ESI-HRMS calculated for C21H15Cl2N2O3 (M+H+): 413.0460; found: 413.0446.

N-(3'-chloro-[1,1'-biphenyl]-3-yl)-N-(5-chloropyridin-3-yl)-2-oxoacetamide 12 (VB-C-70):

To a stirred solution of 8f (0.1 g, 0.23 mmol) in anhydrous DCM (5 mL) was bubbled with ozone at -78 °C until the blue color persisted, and the remaining ozone was removed bubbling nitrogen through the solution. Dimethyl sulfide (3.05 mL, 41.6 mmol) was added, and the mixture was left to attain 25 ^{0}C for 12 h. The solvent was evaporated under reduced vacuum, and the residue was then purified with flash chromatography (0-100% EtOAc in Hexanes as the eluent) to afford VB-C-70 as a white solid (60 mg, 69%).

^{1}H NMR (400 MHz, DMSO-*d6*) δ 8.61 (d, J = 32.8 Hz, 2H), 7.86 – 7.40 (m, 8H), 6.46 (d, J = 8.0 Hz, 1H), 5.23 – 4.93 (m, 1H). ESI-HRMS calculated for C19H13Cl2N2O2 (M+H+): 371.0354; found: 371.0343.

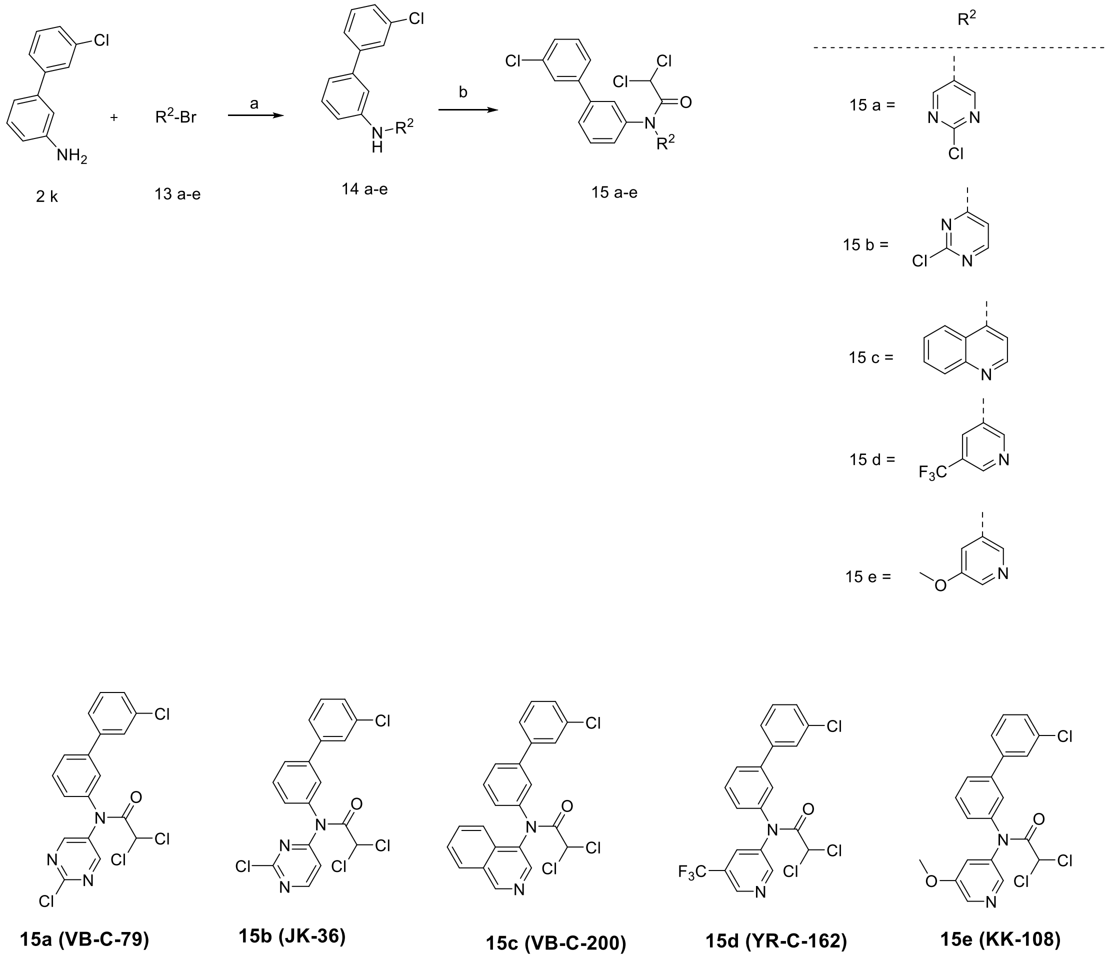

Synthesis Scheme S6. The synthesis of VB-C-79 (15a), JK-36 (15b), VB-C-200 (15c), YR-C-162 (15d), and KK-108 (15e). Reagents and conditions: (a) 13a-e, CS_2CO_3, $Pd_2(dba)_3$, Dppf, Toluene, 100

°C, 24 h; (b) Py, DMAP, Dichloroacetyl chloride, 1,4-Dioxane, 60 °C, 1 h.

2-chloro-N-(3'-chloro-[1,1'-biphenyl]-3-yl)pyrimidin-5-amine (14a):

According to the general procedure for the synthesis of Buchwald coupling (3b-k), 14a compound was synthesized as a white solid (yield 85%).

^{1}H NMR (400 MHz, DMSO-*d6*) δ 8.81 (s, 1H), 8.56 (s, 2H), 7.70 (t, J = 1.9 Hz, 1H), 7.64 – 7.58 (m, 1H), 7.53 – 7.37 (m, 4H), 7.28 (ddd, J = 7.7, 1.8, 1.0 Hz, 1H), 7.19 (ddd, J = 8.1, 2.3, 1.0 Hz, 1H).

2,2-dichloro-N-(3'-chloro-[1,1'-biphenyl]-3-yl)-N-(2-chloropyrimidin-5-yl)acetamide 15a (VB-C-79):

According to the general procedure for the synthesis of Amidation**,** VB-C-79 compound was synthesized as a white solid (yield 64%).

^{1}H NMR (400 MHz, $CDCl_3$) δ 8.63 (s, 2H), 7.66 (dd, J = 30.8, 7.8 Hz, 2H), 7.49 (dt, J = 8.6, 1.7 Hz, 2H), 7.41 – 7.30 (m, 5H), 5.99 (s, 1H). ^{13}C NMR (101 MHz, $CDCl_3$) δ 167.08, 164.20, 155.19, 143.41, 140.30, 135.79, 135.22, 131.79, 130.52, 128.77, 127.57, 127.35, 125.37, 65.16. ESI-HRMS calculated for C18H12Cl4N3O (M+H+): 427.9705; found: 427.9689.

2,2-dichloro-N-(3'-chloro-[1,1'-biphenyl]-3-yl)-N-(2-chloropyrimidin-4-yl)acetamide 15b (JK-36):

According to the general procedure for the synthesis of Amidation**,** JK-36 compound was synthesized as a yellow solid (yield 61%).

^{1}H NMR (400 MHz, DMSO-*d6*) δ 8.71 (d, J = 5.7 Hz, 1H), 7.92 – 7.81 (m, 3H), 7.75 – 7.65 (m, 2H), 7.56 – 7.43 (m, 4H), 6.91 (s, 1H). ^{13}C NMR (101 MHz, $CDCl_3$) δ 165.31, 161.66, 160.26, 142.43, 140.93, 137.94, 135.05, 131.00, 130.34, 128.80, 128.35, 127.59, 127.35, 125.37, 65.39. ESI-HRMS calculated for C18H11Cl4N3O (M+H+): 425.9700; found: 425.9712.

N-(3'-chloro-[1,1'-biphenyl]-3-yl)isoquinolin-4-amine (14c):

According to the general procedure for the synthesis of Buchwald coupling (3b-k), 14c compound was synthesized as a white solid (yield 81%).

^{1}H NMR (400 MHz, DMSO-*d6*) δ 8.98 (s, 1H), 8.46 (d, J = 14.3 Hz, 2H), 8.22 – 8.07 (m, 2H), 7.84 – 7.69 (m, 2H), 7.63 (t, J = 1.9 Hz, 1H), 7.56 (dt, J = 7.7, 1.5 Hz, 1H), 7.47 (t, J = 7.8 Hz, 1H), 7.42 (ddd, J = 7.9, 2.1, 1.2 Hz, 1H), 7.38 – 7.32 (m, 2H), 7.16 (dt, J = 8.0, 1.1 Hz, 1H), 7.12 – 7.05 (m, 1H). ^{13}C NMR (101 MHz, DMSO-*d6*) δ 146.34, 145.63, 143.09, 140.23, 134.25, 134.13, 133.14, 131.23, 130.42, 130.12, 129.86, 129.37, 129.33, 128.22, 128.07, 127.75, 126.78, 125.76, 122.38, 119.07, 116.86, 115.82.

2,2-dichloro-N-(3'-chloro-[1,1'-biphenyl]-3-yl)-N-(isoquinolin-4-yl)acetamide 15c (VB-C-200):

According to the general procedure for the synthesis of Amidation, VB-C-200 compound was synthesized as a gummy solid (yield 69%).

^{1}H NMR (400 MHz, DMSO-*d6*) δ 9.42 (d, J = 44.5 Hz, 2H), 8.89 (d, J = 93.4 Hz, 2H), 8.28 (d, J = 8.2 Hz, 1H), 8.17 (s, 1H), 7.80 (t, J = 7.6 Hz, 2H), 7.64 (s, 1H), 7.53 – 7.48 (m, 2H), 6.89 (s, 1H), 6.56 (s,

1H). ESI-HRMS calculated for C23H16Cl3N2O (M+H+): 441.0328; found: 441.0317.

N-(3'-chloro-[1,1'-biphenyl]-3-yl)-5-(trifluoromethyl)pyridin-3-amine (14d):

According to the general procedure for the synthesis of Buchwald coupling (3b-k), 14d compound was synthesized as a white solid (yield 85%).

^{1}H NMR (400 MHz, DMSO-*d6*) δ 8.90 (s, 1H), 8.65 (d, J = 2.6 Hz, 1H), 8.36 (d, J = 2.0 Hz, 1H), 7.70 – 7.66 (m, 2H), 7.62 (d, J = 7.6 Hz, 1H), 7.52 – 7.42 (m, 4H), 7.37 – 7.28 (m, 1H), 7.23 (dd, J = 7.9, 2.3 Hz, 1H). ^{13}C NMR (101 MHz, DMSO-*d6*) δ 142.67, 142.42, 142.27, 140.99, 140.55, 136.33, 136.28, 134.20, 131.27, 130.78, 127.95, 126.94, 126.07, 125.86, 125.75, 125.56, 122.84, 121.13, 118.47, 117.87, 117.83, 117.35.

2,2-dichloro-N-(3'-chloro-[1,1'-biphenyl]-3-yl)-N-(5-(trifluoromethyl)pyridin-3-yl)acetamide 15d (YR-C-162):

According to the general procedure for the synthesis of Amidation**,** YR-C-162 compound was synthesized as a gummy solid (yield 66%).

^{1}H NMR (400 MHz, $CDCl_3$) δ 8.75 (s, 1H), 8.68 (s, 1H), 8.04 (t, J = 2.3 Hz, 1H), 7.74 (d, J = 7.9 Hz, 1H), 7.66 (t, J = 7.9 Hz, 1H), 7.60 – 7.51 (m, 2H), 7.51 – 7.34 (m, 4H), 6.08 (s, 1H).^{13}C NMR (101 MHz, $CDCl_3$) δ 164.26, 143.12, 140.52, 139.38, 137.97, 135.17, 131.56, 130.46, 128.64, 127.36, 127.13, 125.36, 124.19, 121.47, 63.91. HRMS(ESI) calculated for C19H12Cl4N2O (M+H+): 424.9700; found: 424.9712.

N-(3'-chloro-[1,1'-biphenyl]-3-yl)-5-methoxypyridin-3-amine (14e):

According to the general procedure for the synthesis of Buchwald coupling (3b-k), 14e compound was synthesized as a white solid (yield 80%).

^{1}H NMR (400 MHz, $CDCl_3$) δ 7.99 (d, J = 2.3 Hz, 1H), 7.85 (d, J = 2.5 Hz, 1H), 7.51 – 7.41 (m, 1H), 7.40 – 7.22 (m, 4H), 7.18 (d, J = 4.0 Hz, 1H), 7.14 – 7.07 (m, 1H), 7.07 – 6.97 (m, 1H), 6.91 (t, J = 2.4 Hz, 1H), 5.93 (s, 1H), 3.76 (s, 3H).

2,2-dichloro-N-(3'-chloro-[1,1'-biphenyl]-3-yl)-N-(5-methoxypyridin-3-yl)acetamide 15e (KK-108):

According to the general procedure for the synthesis of Amidation, KK-108 compound was synthesized as a gummy solid (yield 59%).

^{1}H NMR (400 MHz, $CDCl_3$) δ 8.07 (d, J = 50.0 Hz, 2H), 7.76 – 7.21 (m, 9H), 6.01 (s, 1H), 3.80 (s, 3H).^{13}C NMR (100 MHz, $CDCl_3$) δ 171.18, 167.31, 164.06, 155.89, 142.77, 140.79, 140.21, 135.03, 131.27, 130.36, 128.47, 127.58, 127.33, 127.03, 125.40, 118.20, 65.90, 64.21, 60.41, 56.01, 29.72, 21.07, 14.22, 14.14.

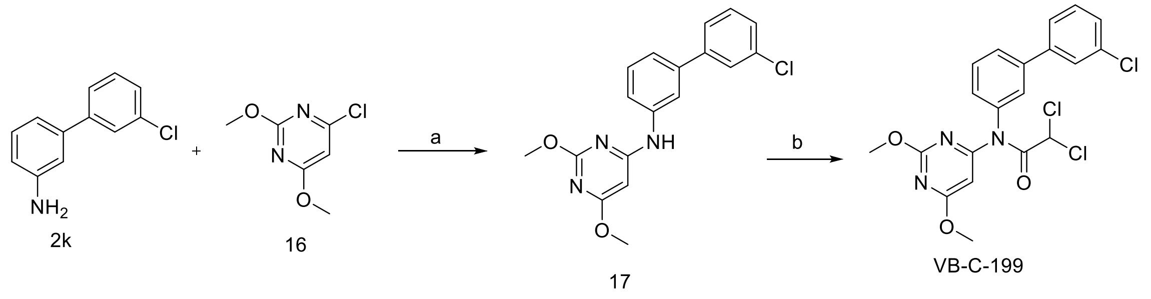

Synthesis Scheme S7. Synthesis of VB-C-199. Reagents and conditions: (a) 16, CS_2CO_3, $Pd_2(dba)_3$, Dppf, Toluene, 100 °C, 24 h; (b) Py, DMAP, Dichloroacetyl chloride, 1,4-Dioxane, 60 °C, 1 h.

N-(3'-chloro-[1,1'-biphenyl]-3-yl)-2,6-dimethoxypyrimidin-4-amine (17):

According to the general procedure for the synthesis of Buchwald coupling (3b-k), 17 compound was synthesized as a white solid (yield 80%).

^{1}H NMR (400 MHz, DMSO-*d6*) δ 9.55 (s, 1H), 8.03 (t, J = 2.0 Hz, 1H), 7.67 (t, J = 1.9 Hz, 1H), 7.64 – 7.58 (m, 2H), 7.51 (t, J = 7.8 Hz, 1H), 7.46 – 7.39 (m, 2H), 7.33 – 7.28 (m, 1H), 5.79 (s, 1H), 3.86 (d, J = 26.4 Hz, 6H).

2,2-dichloro-N-(3'-chloro-[1,1'-biphenyl]-3-yl)-N-(2,6-dimethoxypyrimidin-4-yl)acetamide (VB-C-199):

According to the general procedure for the synthesis of Amidation, VB-C-199 compound was synthesized as a gummy solid (yield 55%).

^{1}H NMR (400 MHz, DMSO-*d6*) δ 7.86 – 7.75 (m, 2H), 7.73 – 7.57 (m, 3H), 7.54 – 7.43 (m, 2H), 7.36 (dt, J = 7.9, 1.5 Hz, 1H), 7.11 (s, 1H), 6.35 (s, 1H), 3.87 (d, J = 12.5 Hz, 6H). ESI-HRMS calculated for C20H17Cl3N3O3 (M+H+): 452.0335; found: 452.0328.

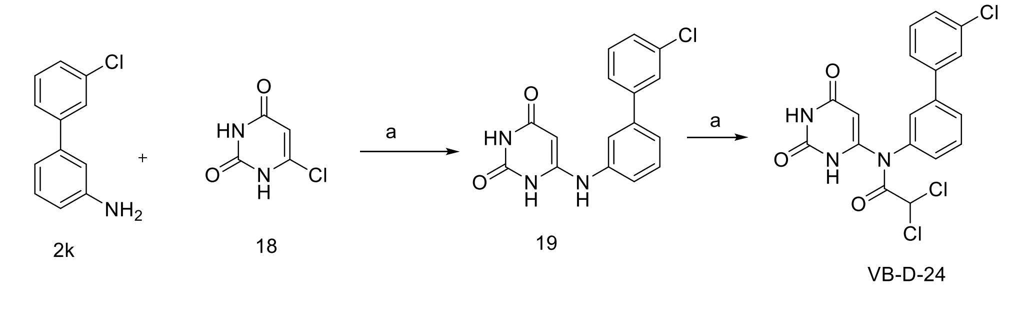

Synthesis Scheme S8. Synthesis of VB-D-24. Reagents and conditions: (a) EtOH, Microwave, 160 °C, 30 min; (b) Py, DMAP, Dichloroacetyl chloride, 1,4-Dioxane, 60 °C, 1 h.

6-((3'-chloro-[1,1'-biphenyl]-3-yl)amino)pyrimidine-2,4(1H,3H)-dione (19):

To a stirred solution of 2k (1 eq) in EtOH was added 18 (1.2 eq). The reaction mixture was stirred at 160 °C for 30 min under microwave. After completion of the reaction, the reaction mixture was concentrated and purified by flash chromatography (0-100% EA in Hexane as the eluent) to afford 19 as a yellow gummy solid in good yield (70%).

^{1}H NMR (400 MHz, DMSO-*d6*) δ 10.41 (d, J = 73.3 Hz, 2H), 8.36 (s, 1H), 7.72 (t, J = 1.9 Hz, 1H), 7.64 (dt, J = 7.7, 1.5 Hz, 1H), 7.56 – 7.42 (m, 5H), 7.26 (ddt, J = 6.6, 4.6, 2.1 Hz, 1H), 4.77 (t, J = 1.7 Hz, 1H). ^{13}C NMR (101 MHz, DMSO-*d6)* δ 164.87, 152.65, 151.38, 142.17, 140.33, 139.20, 134.25, 131.32, 130.58, 128.10, 126.97, 125.93, 123.60, 122.75, 121.47, 76.85.

2,2-dichloro-N-(3'-chloro-[1,1'-biphenyl]-3-yl)-N-(2,6-dioxo-1,2,3,6-tetrahydropyrimidin-4-yl)acetamide (VB-D-24):

According to the general procedure for the synthesis of Amidation, VB-D-24 compound was synthesized as a gummy solid (yield 61%).

^{1}H NMR (400 MHz, DMSO-*d6*) δ 12.49 (s, 1H), 11.14 (s, 1H), 7.97 (s, 1H), 7.90 – 7.82 (m, 2H), 7.79 – 7.69 (m, 2H), 7.64 – 7.39 (m, 5H). ESI-HRMS calculated for C18H11Cl3N3O3 (M-H): 423.9836; found: 423.9991.

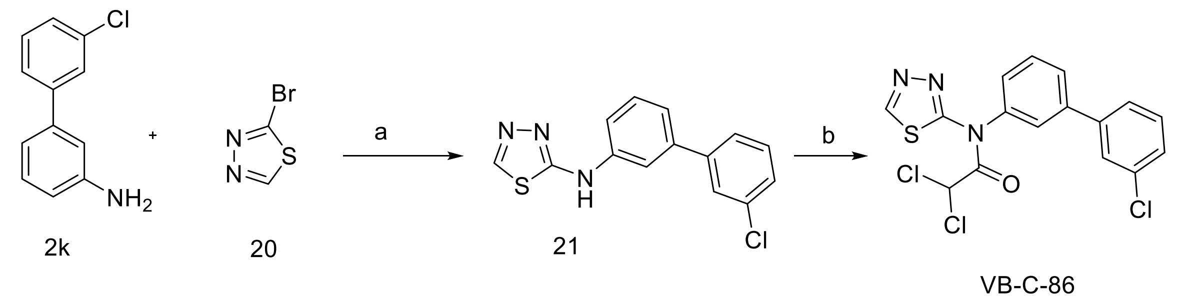

Synthesis Scheme S9. Synthesis of VB-C-86. Reagents and conditions: (a) EtOH, PTSA, 80 °C, 16 h; (b) Py, DMAP, Dichloroacetyl chloride, 1,4-Dioxane, 60 °C, 1 h.

3'-chloro-[1,1'-biphenyl]-3-yl)-1,3,4-thiadiazol-2-amine (21):

A mixture of 2-bromo-1,3,4-thiadiazole compound (0.5 g, 3.0 mmol), Compound 2k (0.62 g ,3.0 mmol) and p-toluenesulfonic acid (0.58 g, 3.0 mmol) in ethanol (35 mL) was refluxed overnight. Monitoring by thin layer chromatography (petrol ether: ethyl acetate=2:1) showed the reaction was complete. The mixture was partitioned between saturated aqueous NaHCO3 (50 mL) and ethyl acetate (50 mL) and extracted with ethyl acetate (50 m L x 2). The combined organic layers were dried and concentrated. The residue was recrystallized with ethyl acetate to give Compound 21 (0.3 g, 35%) as light-yellow solid.

^{1}H NMR (400 MHz, $CDCl_3$) δ 7.91 (s, 1H), 7.75 (t, J = 1.9 Hz, 1H), 7.65 – 7.46 (m, 4H), 7.43 (dt, J = 7.2, 1.7 Hz, 1H), 7.36 – 7.28 (m, 2H).

2,2-dichloro-N-(3'-chloro-[1,1'-biphenyl]-3-yl)-N-(1,3,4-thiadiazol-2-yl)acetamide (VB-C-86):

According to the general procedure for the synthesis of Amidation**,** VB-C-86 compound was synthesized as a gummy solid (yield **55**%).

^{1}H NMR (400 MHz, DMSO-*d6*) δ 9.32 (s, 1H), 8.02 (t, J = 1.9 Hz, 1H), 7.91 (dt, J = 7.6, 1.4 Hz, 1H), 7.76 (t, J = 1.9 Hz, 1H), 7.69 – 7.62 (m, 2H), 7.58 (ddd, J = 8.0, 2.1, 1.2 Hz, 1H), 7.49 – 7.38 (m, 2H),

6.51 (s, 1H). ^{13}C NMR (101 MHz, DMSO-*d6*) δ 161.20, 159.67, 149.93, 139.11, 138.61, 135.75, 132.17, 129.19, 128.89, 127.46, 126.80, 126.21, 126.16, 124.97, 123.88, 63.56. ESI-HRMS calculated for C16H11Cl3N3OS (M+H+): 397.9688; found: 397.9685.

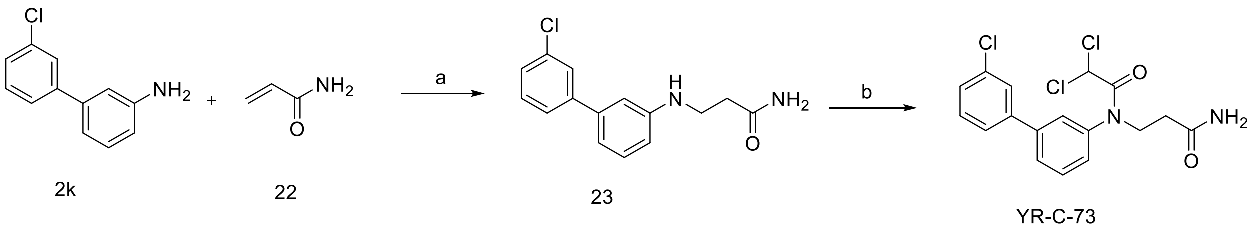

Synthesis Scheme S10. Synthesis of YR-C-73. Reagents and conditions: (a) Na_2CO_3, 100 °C, 16 h; (b) Py, DMAP, Dichloroacetyl chloride, 1,4-Dioxane, 60 °C, 1 h.

3-((3'-chloro-[1,1'-biphenyl]-3-yl)amino)propenamide (23):

To a stirred solution of 2k (1 eq) in THF was added 22 (1.2 eq) followed by Na_2CO_3 (2 eq). The reaction mixture was stirred at 100 °C for 16h. After completion of the reaction, the reaction mixture was concentrated and purified by flash chromatography (0-100% EA in Hexane as the eluent) to afford **23** as a yellow gummy solid in good yield (82%).

^{1}H NMR (400 MHz, DMSO-*d6*) δ 7.62 (t, J = 1.9 Hz, 1H), 7.59 – 7.54 (m, 1H), 7.47 (t, J = 7.8 Hz, 1H), 7.42 – 7.33 (m, 2H), 7.17 (t, J = 8.0 Hz, 1H), 6.87 – 6.77 (m, 3H), 6.65 – 6.58 (m, 1H), 5.78 – 5.67 (m, 1H), 3.30 (t, J = 6.5 Hz, 2H), 2.36 (t, J = 7.0 Hz, 2H). ^{13}C NMR (101 MHz, DMSO-*d6*) δ 173.34, 149.69, 143.82, 139.92, 133.99, 131.07, 130.06, 127.43, 126.73, 125.77, 114.81, 112.52, 110.64, 55.39, 35.35.

3-(2,2-dichloro-N-(3'-chloro-[1,1'-biphenyl]-3-yl)acetamido)propenamide (YR-C-73):

According to the general procedure for the synthesis of Amidation, YR-C-73 compound was synthesized as a gummy solid (yield 54%).

^{1}H NMR (400 MHz, $CDCl_3$) δ 7.70 (dt, J = 8.0, 1.4 Hz, 1H), 7.61 (t, J = 7.8 Hz, 1H), 7.57 (dq, J = 4.7, 1.7 Hz, 2H), 7.48 – 7.45 (m, 1H), 7.45 – 7.34 (m, 3H), 5.89 (s, 1H), 4.04 (t, J = 6.7 Hz, 2H), 2.82 (t, J = 6.7 Hz, 2H). ^{13}C NMR (101 MHz, $CDCl_3$) δ 164.49, 142.68, 140.79, 140.18, 135.11, 131.25, 130.45, 128.63, 128.47, 127.34, 127.05, 126.68, 125.37, 117.34, 63.38, 47.02, 36.67. HRMS(ESI) calculated for C17H15Cl3N2O2 (M+H+): 385.0200; found: 385.0272.

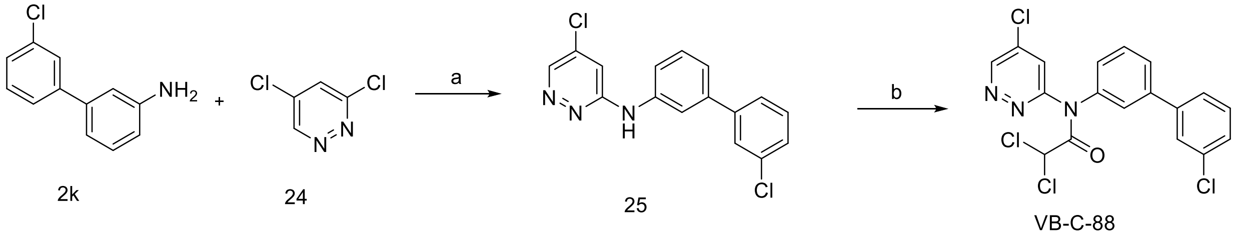

Synthesis Scheme S11. Synthesis of VB-C-88. Reagents and conditions: (a) EtOH, PTSA, 80 °C, 16 h; (b) Py, DMAP, Dichloroacetyl chloride, 1,4-Dioxane, 60 °C, 1 h.

5-chloro-N-(3'-chloro-[1,1'-biphenyl]-3-yl)pyridazin-3-amine (25):

The same procedure for the synthesis of compound 21 was followed except that the 20 was replaced with 24 to afford compound 25 as a white solid (yield 70%).

^{1}H NMR (400 MHz, DMSO-*d6*) δ 10.15 (s, 1H), 8.20 (d, J = 5.9 Hz, 1H), 7.92 (t, J = 2.1 Hz, 1H), 7.71 (t, J = 1.9 Hz, 1H), 7.64 (dt, J = 7.7, 1.5 Hz, 2H), 7.57 – 7.37 (m, 4H), 6.81 (d, J = 5.9 Hz, 1H).

2,2-dichloro-N-(3'-chloro-[1,1'-biphenyl]-3-yl)-N-(5-chloropyridazin-3-yl)acetamide (VB-C-88):

According to the general procedure for the synthesis of Amidation, VB-C-88 compound was synthesized as a gummy solid (yield 58%).

^{1}H NMR (400 MHz, DMSO-*d6*) δ 9.33 (d, J = 2.2 Hz, 1H), 8.20 (d, J = 2.2 Hz, 1H), 7.83 – 7.73 (m, 3H), 7.62 (dt, J = 7.6, 1.6 Hz, 1H), 7.56 (t, J = 7.8 Hz, 1H), 7.45 (d, J = 3.0 Hz, 1H), 7.44 – 7.38 (m, 2H), 6.71 (s, 1H). ^{13}C NMR (101 MHz, $CDCl_3$) δ 165.07, 157.65, 149.23, 142.48, 140.88, 139.97, 138.41, 135.00, 131.08, 130.34, 128.82, 128.35, 128.17, 127.60, 127.35, 125.42, 124.25, 64.50. ESI-HRMS calculated for C18H12Cl4N3O (M+H+): 427.9705; found: 427.9689.

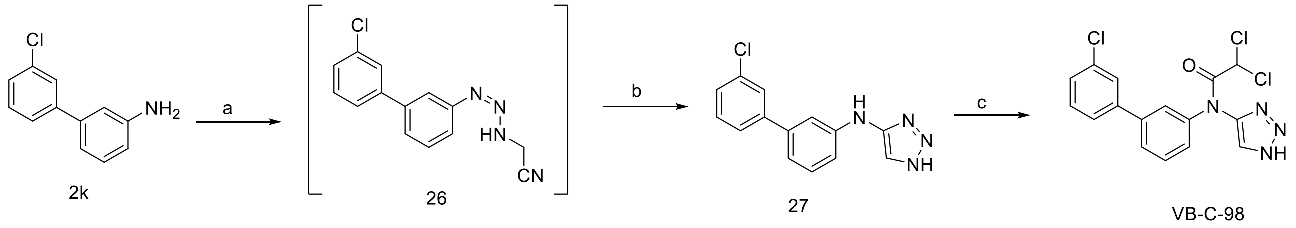

Synthesis Scheme S12. Synthesis of VB-C-98. Reagents and conditions: (a) Cyanomethylamine sulfate, NaN02, 2M HCl, NaOAc, 0 °C to rt, 2h; (b)EtOH, 80 °C, 4 h; (c) Py, DMAP, Dichloroacetyl chloride, 1,4-Dioxane, 60 °C, 1 h.

N-(3'-chloro-[1,1'-biphenyl]-3-yl)-1H-1,2,3-triazol-4-amine (27):

Dissolve 2k (500 mg, 2.46 mmol) in 6.03 mL of 2 M hydrochloric acid (12.06 mmol), diluted with 8 mL of water. Cool the mixture to 0°C. Add sodium nitrite (170 mg, 2.46 mmol) to the cooled solution. Stir the mixture at 0°C for 20 minutes. Add a solution of Cyanomethylamine sulfate (380 mg, 2.46 mmol) in 3 mL of water slowly. Stir the mixture for 10 minutes at 0°C. Add sodium acetate (3.0 g, 37.0 mmol). Allow the mixture to warm to room temperature. Stir the mixture at room temperature for 1 hour. Collect the resulting precipitate by filtration. Wash the filtrate with water to obtain intermediate 26. Dissolve the intermediate 26 (238 mg, 1.0 mmol) in 7 mL of ethanol. Heat the solution under reflux for 4 hours. Allow the mixture to cool to room temperature. Concentrate the filtrate under reduced pressure. Triturate the crude with CH2Cl2 to obtain a cream-colored solid. Purify the solid by flash chromatography (40% EtOAc in petroleum ether) to obtain 27 as a white solid. (yield 80%).

^{1}H NMR (400 MHz, DMSO-*d6*) δ 14.08 (s, 1H), 8.82 (s, 1H), 7.57 (dt, J = 4.0, 2.0 Hz, 2H), 7.51 (dt, J = 7.8, 1.4 Hz, 1H), 7.44 (d, J = 7.8 Hz, 1H), 7.39 – 7.35 (m, 1H), 7.27 – 7.20 (m, 2H), 7.01 (dt, J = 7.1, 1.7 Hz, 1H).

2,2-dichloro-N-(3'-chloro-[1,1'-biphenyl]-3-yl)-N-(1H-1,2,3-triazol-4-yl)acetamide (VB-C-98):

According to the general procedure for the synthesis of Amidation**,** VB-C-98 compound was synthesized as a gummy solid (yield 58%).

^{1}H NMR (400 MHz, DMSO-*d6*) δ 11.06 (s, 1H), 7.97 (s, 1H), 7.88 (ddd, J = 7.8, 1.9, 1.1 Hz, 1H), 7.84 (t, J = 1.9 Hz, 1H), 7.78 (t, J = 1.9 Hz, 1H), 7.70 – 7.64 (m, 2H), 7.57 (ddd, J = 7.9, 2.1, 1.1 Hz, 1H), 7.49 – 7.40 (m, 2H), 6.63 (s, 1H). ESI-HRMS calculated for C16H12Cl3N4O2 (M+H+): 381.0077; found: 381.0060.

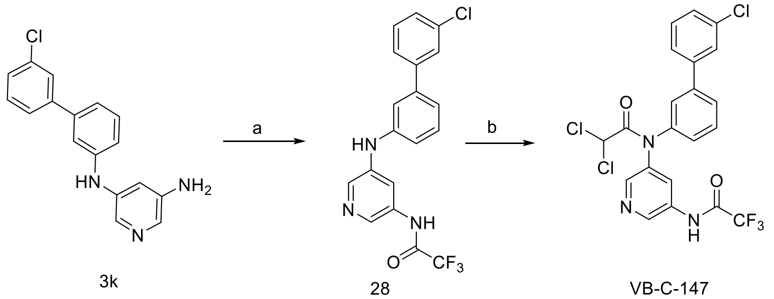

3k 28 VB-C-147

Synthesis Scheme S13. Synthesis of VB-C-147. Reagents and conditions: (a) CF_3COOMe, DMAP, MeOH, 0 °C to rt, 16h; (b) Py, DMAP, Dichloroacetyl chloride, 1,4-Dioxane, 60 °C, 1 h.

N-(5-((3'-chloro-[1,1'-biphenyl]-3-yl)amino)pyridin-3-yl)-2,2,2-trifluoroacetamide (28):

To a stirred solution of 3k (1 eq) in MeOH was added DMAP (2eq) followed by CF_3COOMe (1.2 eq). The reaction mixture was stirred at rt for 16h. After completion of the reaction, the reaction mixture was concentrated and purified by flash chromatography (0-100% EA in Hexane as the eluent) to afford **28** as a white solid in good yield (82%).

^{1}H NMR (400 MHz, DMSO-*d6*) δ 8.70 (s, 1H), 8.34 (d, *J* = 2.1 Hz, 1H), 8.22 (d, *J* = 2.5 Hz, 1H), 8.00 (t, *J* = 2.3 Hz, 1H), 7.74 – 7.57 (m, 2H), 7.57 – 7.35 (m, 4H), 7.21 (dddd, *J* = 30.1, 8.0, 2.1, 1.0 Hz, 2H).

2,2-dichloro-N-(3'-chloro-[1,1'-biphenyl]-3-yl)-N-(5-(2,2,2-trifluoroacetamido)pyridin-3-yl)acetamide (VB-C-147):

According to the general procedure for the synthesis of Amidation, VB-C-147 compound was synthesized as a gummy solid (yield 61%).

^{1}H NMR (400 MHz, DMSO-*d6*) δ 11.56 (s, 1H), 8.70 (d, J = 41.9 Hz, 1H), 8.44 (d, J = 38.2 Hz, 1H), 8.21 – 7.84 (m, 2H), 7.82 – 7.24 (m, 7H), 6.50 (d, J = 35.9 Hz, 1H). ESI-HRMS calculated for C21H14Cl3F3N3O2 (M+H+): 502.0104; found: 502.0100.

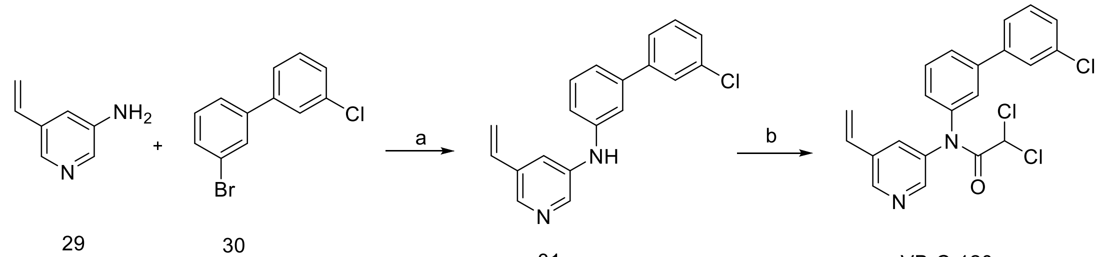

29 30 31 VB-C-120

Synthesis Scheme S14. Synthesis of VB-C-120. Reagents and conditions: (a) CS_2CO_3, $Pd_2(dba)_3$, Dppf, Toluene, 100 °C, 24 h; (b) Py, DMAP, Dichloroacetyl chloride, 1,4-Dioxane, 60 °C, 1 h.

N-(3'-chloro-[1,1'-biphenyl]-3-yl)-5-vinylpyridin-3-amine:

According to the general procedure for the synthesis of Buchwald coupling (3b-k), 31 compound was synthesized as a white solid (yield 82%).

^{1}H NMR (400 MHz, $CDCl_3$) δ 8.29 (dd, J = 41.4, 2.2 Hz, 2H), 7.57 (t, J = 1.8 Hz, 1H), 7.50 (t, J = 2.2 Hz, 1H), 7.45 (dt, J = 7.3, 1.6 Hz, 1H), 7.43 – 7.33 (m, 3H), 7.28 (dd, J = 4.3, 2.4 Hz, 1H), 7.21 (dt, J = 7.7, 1.2 Hz, 1H), 7.13 (ddd, J = 8.0, 2.3, 1.0 Hz, 1H), 6.70 (dd, J = 17.6, 11.0 Hz, 1H), 5.97 (s, 1H), 5.81 (d, J = 17.7 Hz, 1H), 5.40 (d, J = 11.0 Hz, 1H). ^{13}C NMR (101 MHz, $CDCl_3$) δ 142.67, 142.59, 141.46, 140.90, 139.56, 139.55, 134.69, 133.52, 133.45, 130.14, 130.04, 127.56, 127.28, 125.27, 120.92, 120.43, 117.56, 117.02, 116.46.

2,2-dichloro-N-(3'-chloro-[1,1'-biphenyl]-3-yl)-N-(5-vinylpyridin-3-yl)acetamide (VB-C-120):

According to the general procedure for the synthesis of Amidation, VB-C-120 compound was synthesized as a gummy solid (yield 58%).

^{1}H NMR (400 MHz, DMSO-*d6*) δ 8.60 (d, J = 43.5 Hz, 2H), 8.03 (s, 1H), 7.84 – 7.69 (m, 2H), 7.69 – 7.58 (m, 2H), 7.58 – 7.32 (m, 4H), 6.73 (dd, J = 17.8, 11.0 Hz, 1H), 6.58 (d, J = 18.4 Hz, 1H), 5.98 (d, J = 18.0 Hz, 1H), 5.40 (d, J = 10.9 Hz, 1H). ^{13}C NMR (101 MHz, $CDCl_3$) δ 167.29, 164.15, 142.79, 135.08, 134.36, 131.98, 130.39, 129.50, 128.50, 127.35, 126.38, 125.40, 118.74, 64.07. ESI-HRMS calculated for C21H16Cl3N2O2 (M+H+): 417.0328; found: 417.0314.

2,2-dichloro-N-(3'-chloro-[1,1'-biphenyl]-3-yl)-N-(5-(2,2,2-trifluoroacetamido)pyridin-3-yl)acetamide (JK-25):

According to the general procedure for the synthesis of Amidation**,** JK-25 compound was synthesized as a gummy solid (yield 58%).

^{1}H NMR (400 MHz, DMSO-*d6*) δ 8.61 (s, 2H), 8.07 (d, J = 32.2 Hz, 2H), 7.85 (s, 2H), 7.72 (s, 2H), 7.56 – 7.45 (m, 3H), 6.64 (m, 2H). ESI-HRMS calculated for C21H14Cl3F3N3O2 (M+H+): 502.0104; found: 502.0100.

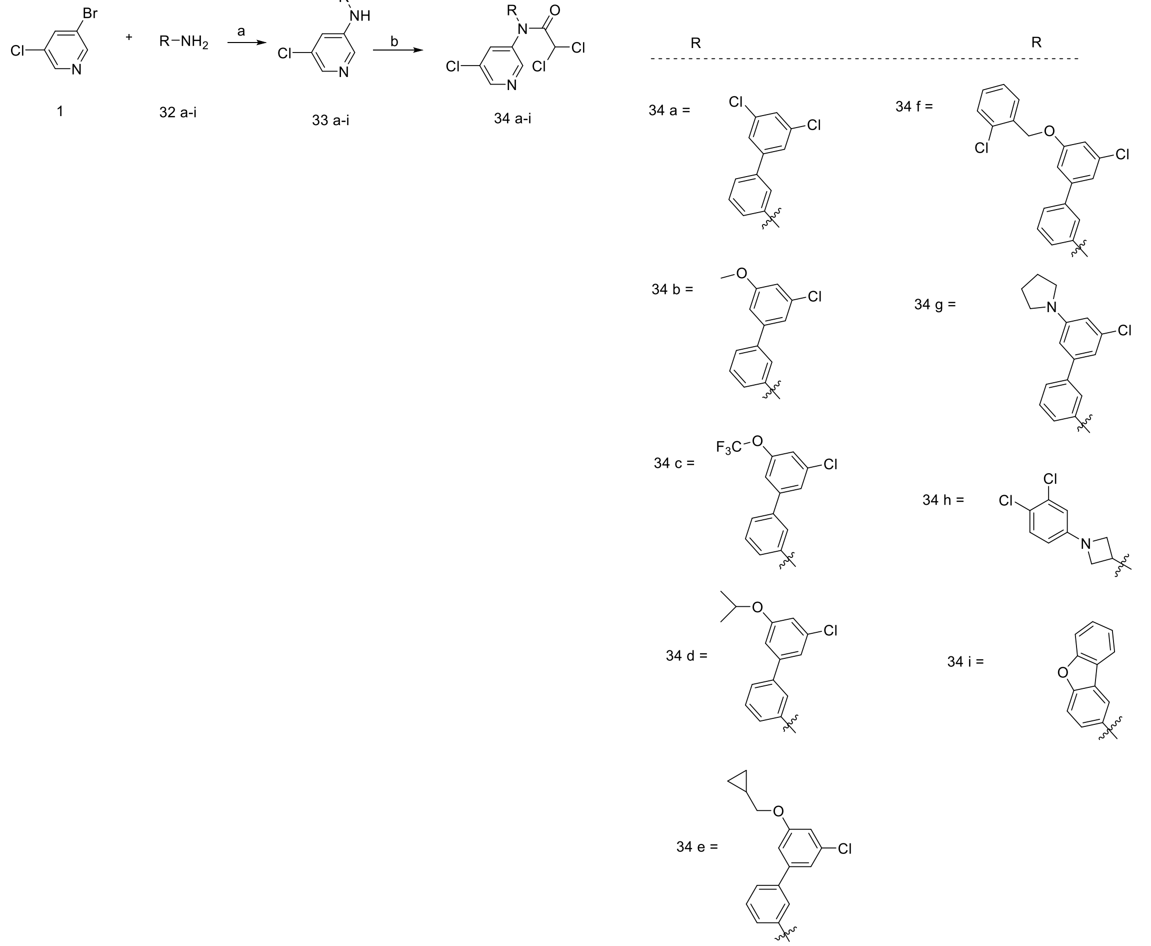

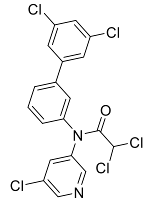
34a (SR-B-120)

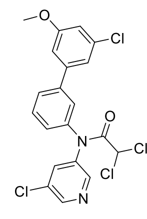
34b (VB-C-140)

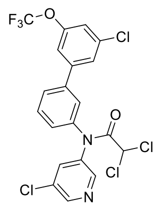
34c (YR-C-154)

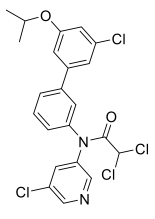
34d (VB-C-171)

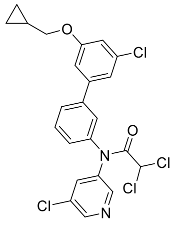
34e (VB-C-172)

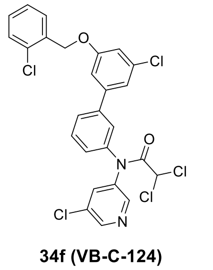
34f (VB-C-124)

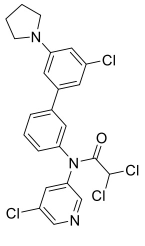
34g (KK114)

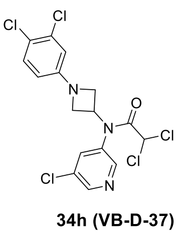
34h (VB-D-37)

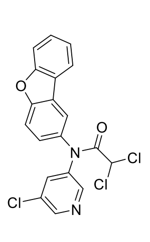
34i (SR-B-101)

Synthesis Scheme S15. The synthesis of SR-B-120 (34a), VB-C-140 (34b), YR-C-154 (34c), VB-C-171 (34d), VB-C-172 (34e), VB-C-124 (34f), KK114 (34g), VB-D-37 (34h), and SR-B-101(34i). Reagents and conditions: (a) 32a-i, CS_2CO_3, $Pd_2(dba)_3$, Dppf, Toluene, 100 °C, 24 h; (b) Py, DMAP, Dichloroacetyl chloride, 1,4-Dioxane, 60 °C, 1 h.

5-chloro-N-(3',5'-dichloro-[1,1'-biphenyl]-3-yl)pyridin-3-amine (33a):

According to the general procedure for the synthesis of Buchwald coupling (3b-k), 33a compound was synthesized as a white solid (yield 85%).

^{1}H NMR (400 MHz, DMSO-*d6*) δ 8.73 (d, J = 33.9 Hz, 1H), 8.32 (dd, J = 13.9, 5.8 Hz, 1H), 8.02 (s, 1H), 7.67 (s, 2H), 7.58 (s, 1H), 7.50 (s, 1H), 7.41 (t, J = 7.7 Hz, 2H), 7.32 – 7.18 (m, 2H).

2,2-dichloro-N-(5-chloropyridin-3-yl)-N-(3',5'-dichloro-[1,1'-biphenyl]-3-yl)acetamide 34a (SR-B-120):

According to the general procedure for the synthesis of Amidation, SR-B-120 compound was synthesized as a gummy solid (yield 65%).

^{1}H NMR (400 MHz, $CDCl_3$) δ 8.31 (dd, J = 54.1, 22.7 Hz, 2H), 7.74 (s, 1H), 7.60 (s, 2H), 7.45 (s, 1H), 7.41 – 7.32 (m, 4H), 5.96 (s, 1H). HRMS(ESI) calculated for C19H11Cl5N2O (M+H+): 458.9300; found: 458.9379

5-chloro-*N*-(3'-chloro-5'-methoxy-[1,1'-biphenyl]-3-yl)pyridin-3-amine (33b):

According to the general procedure for the synthesis of Buchwald coupling (3b-k), 33b compound was synthesized as a white solid (yield 80%).

^{1}H NMR (400 MHz, DMSO-*d6*) δ 8.74 (s, 1H), 8.33 (d, J = 2.4 Hz, 1H), 8.04 (d, J = 2.1 Hz, 1H), 7.51 (t, J = 2.3 Hz, 1H), 7.48 – 7.34 (m, 2H), 7.33 – 7.18 (m, 3H), 7.14 (dd, J = 2.4, 1.5 Hz, 1H), 7.05 (t, J = 2.1 Hz, 1H), 3.85 (s, 3H). ^{13}C NMR (101 MHz, DMSO-*d6*) δ 161.02, 143.57, 142.45, 141.81, 140.53, 138.59, 137.43, 134.78, 131.58, 130.62, 121.19, 120.88, 119.28, 118.29, 117.26, 113.57, 111.96, 56.17.

2,2-dichloro-N-(3'-chloro-5'-methoxy-[1,1'-biphenyl]-3-yl)-N-(5-chloropyridin-3-yl)acetamide 34b (VB-C-140):

According to the general procedure for the synthesis of Amidation, VB-C-140 compound was synthesized as a gummy solid (yield 61%).

^{1}H NMR (400 MHz, DMSO-*d6*) δ 9.04 – 8.50 (m, 2H), 8.16 (d, J = 27.6 Hz, 1H), 8.03 – 7.16 (m, 6H), 7.08 (t, J = 2.0 Hz, 1H), 6.60 (s, 1H), 3.86 (s, 3H). ESI-HRMS calculated for C20H15Cl4N2O2 (M+H+): 456.9858; found: 456.9851.

5-chloro-N-(3'-chloro-5'-(trifluoromethoxy)-[1,1'-biphenyl]-3-yl)pyridin-3-amine (33c):

According to the general procedure for the synthesis of Buchwald coupling (3b-k), 33c compound was synthesized as a white solid (yield 80%).

^{1}H NMR (400 MHz, DMSO-*d6*) δ 8.77 (s, 1H), 8.34 (d, J = 2.4 Hz, 1H), 8.04 (d, J = 2.1 Hz, 1H), 7.78 (t, J = 1.7 Hz, 1H), 7.65 – 7.58 (m, 1H), 7.55 (td, J = 2.0, 1.0 Hz, 1H), 7.52 (t, J = 2.3 Hz, 1H), 7.47 – 7.39 (m, 2H), 7.35 – 7.28 (m, 1H), 7.24 (ddd, J = 8.1, 2.3, 1.0 Hz, 1H). ^{13}C NMR (101 MHz, DMSO-*d6*) δ 149.65, 149.63, 149.61, 149.59, 144.35, 142.66, 141.66, 138.97, 138.71, 137.49, 135.15, 131.58, 130.78, 126.40, 124.28, 121.72, 121.35, 120.87, 120.78, 119.16, 118.80, 118.76, 117.19, 116.60.

2,2-dichloro-N-(3'-chloro-5'-(trifluoromethoxy)-[1,1'-biphenyl]-3-yl)-N-(5-chloropyridin-3-yl)acetamide 34c (YR-C-154):

According to the general procedure for the synthesis of Amidation**,** YR-C-154 compound was synthesized as a gummy solid (yield 55%).

^{1}H NMR (400 MHz, DMSO-*d6*) δ 8.59 (d, J = 35.2 Hz, 2H), 8.12 (s, 1H), 7.99 – 7.36 (m, 6H), 6.58 (s, 1H). HRMS(ESI) calculated for C20H11Cl4F3N2O2 (M+H+): 508.9500; found: 508.9570.

5-chloro-N-(3'-chloro-5'-isopropoxy-[1,1'-biphenyl]-3-yl)pyridin-3-amine (33d):

According to the general procedure for the synthesis of Buchwald coupling (3b-k), 33d compound was synthesized as a white solid (yield 75%).

^{1}H NMR (400 MHz, DMSO-*d6*) δ 8.73 (s, 1H), 8.33 (d, J = 2.4 Hz, 1H), 8.02 (dd, J = 8.1, 2.1 Hz, 1H), 7.51 (t, J = 2.2 Hz, 1H), 7.41 (d, J = 7.8 Hz, 1H), 7.38 (dd, J = 4.3, 2.1 Hz, 1H), 7.28 – 7.24 (m, 1H), 7.22 – 7.17 (m, 2H), 7.10 (t, J = 1.9 Hz, 1H), 7.00 (t, J = 2.1 Hz, 1H), 4.76 (hept, J = 6.0 Hz, 1H), 1.30 (s, 3H), 1.29 (s, 3H). ^{13}C NMR (101 MHz, DMSO-*d6*) δ 159.23, 143.63, 142.44, 141.79, 140.54, 138.58, 137.44, 134.81, 131.57, 130.60, 121.20, 120.81, 119.07, 118.23, 117.17, 115.05, 113.32, 70.39, 22.18.

2,2-dichloro-N-(3'-chloro-5'-isopropoxy-[1,1'-biphenyl]-3-yl)-N-(5-chloropyridin-3-yl)acetamide 34d (VB-C-171):

According to the general procedure for the synthesis of Amidation, VB-C-171 compound was synthesized as a gummy solid (yield 65%).

^{1}H NMR (400 MHz, DMSO-*d6*) δ 9.03 – 8.45 (m, 2H), 8.16 (d, J = 29.9 Hz, 1H), 7.98 – 7.78 (m, 1H), 7.78 – 7.17 (m, 5H), 7.05 (t, J = 2.0 Hz, 1H), 6.84 – 6.51 (m, 1H), 4.78 (m, 1H), 1.30 (d, J = 6.1 Hz, 6H). ESI-HRMS calculated for C22H19Cl4N2O2 (M+H+): 485.0171; found: 485.0159.

5-chloro-N-(3'-chloro-5'-(cyclopropylmethoxy)-[1,1'-biphenyl]-3-yl)pyridin-3-amine (33e):

According to the general procedure for the synthesis of Buchwald coupling (3b-k), 33e compound was synthesized as a white solid (yield 81%).

^{1}H NMR (400 MHz, DMSO-*d6*) δ 8.60 (s, 1H), 8.27 – 8.12 (m, 1H), 7.90 (d, J = 2.1 Hz, 1H), 7.37 (t, J = 2.3 Hz, 1H), 7.30 – 7.24 (m, 2H), 7.14 (dt, J = 7.9, 1.2 Hz, 1H), 7.11 – 7.04 (m, 2H), 7.01 – 6.97 (m, 1H), 6.88 (t, J = 2.0 Hz, 1H), 3.79 (d, J = 7.0 Hz, 2H), 1.13 – 1.09 (m, 1H), 0.53 – 0.41 (m, 2H), 0.26 – 0.15 (m, 2H).

2,2-dichloro-N-(3'-chloro-5'-(cyclopropylmethoxy)-[1,1'-biphenyl]-3-yl)-N-(5-chloropyridin-3-yl)acetamide 34e (VB-C-172):

According to the general procedure for the synthesis of Amidation, VB-C-172 compound was synthesized as a gummy solid (yield 63%).

^{1}H NMR (400 MHz, DMSO-*d6*) δ 9.04 – 8.59 (m, 2H), 8.22 (d, J = 27.8 Hz, 1H), 8.04 – 7.85 (m, 1H),

7.75 – 7.31 (m, 5H), 7.13 – 7.07 (m, 1H), 6.86 – 6.63 (m, 1H), 3.98 (d, J = 7.1 Hz, 2H), 1.32 – 1.26 (m, 1H), 0.74 – 0.58 (m, 2H), 0.40 (t, J = 5.1 Hz, 2H). ESI-HRMS calculated for C23H19Cl4N2O2 (M+H+): 497.0171; found: 497.0163.

N-(3'-chloro-5'-((2-chlorobenzyl)oxy)-[1,1'-biphenyl]-3-yl)-5-methoxypyridin-3-amine (33f):

According to the general procedure for the synthesis of Buchwald coupling (3b-k), 33f compound was synthesized as a white solid (yield 81%).

^{1}H NMR (400 MHz, DMSO-*d6*) δ 8.52 (s, 1H), 8.04 (d, J = 2.3 Hz, 1H), 7.81 (d, J = 2.5 Hz, 1H), 7.69 – 7.59 (m, 1H), 7.59 – 7.46 (m, 1H), 7.46 – 7.31 (m, 4H), 7.31 – 7.12 (m, 5H), 7.07 (t, J = 2.4 Hz, 1H), 5.26 (s, 2H), 3.80 (s, 3H). ^{13}C NMR (101 MHz, DMSO-*d6*) δ 159.90, 156.37, 143.88, 143.51, 141.12, 140.28, 134.80, 134.34, 133.32, 132.07, 130.92, 130.56, 130.50, 129.92, 128.79, 127.88, 119.81, 119.76, 117.46, 116.25, 114.35, 112.64, 107.92, 67.90, 55.81.

2,2-dichloro-N-(3'-chloro-5'-((2-chlorobenzyl)oxy)-[1,1'-biphenyl]-3-yl)-N-(5-methoxypyridin-3-yl)acetamide 34f (VB-C-124):

According to the general procedure for the synthesis of Amidation, VB-C-124 compound was synthesized as a gummy solid (yield 61%).

^{1}H NMR (400 MHz, DMSO-*d6*) δ 8.36 (d, J = 49.9 Hz, 1H), 8.12 (dd, J = 74.1, 12.9 Hz, 2H), 7.77 (s, 1H), 7.67 – 7.24 (m, 9H), 7.13 (d, J = 2.2 Hz, 1H), 6.52 (s, 1H), 5.21 (s, 2H), 3.79 (s, 3H). ESI-HRMS calculated for C26H18Cl5N2O2 (M+H+): 566.9781; found: 566.9764.

5-chloro-N-(3'-chloro-5'-(pyrrolidin-1-yl)-[1,1'-biphenyl]-3-yl)pyridin-3-amine (33g):

According to the general procedure for the synthesis of Buchwald coupling (3b-k), 33g compound was synthesized as a white solid (yield 87%).

^{1}H NMR (400 MHz, DMSO-*d6*) δ 8.74 (s, 1H), 8.32 (d, J = 2.4 Hz, 1H), 8.02 (d, J = 2.1 Hz, 1H), 7.50 (t, J = 2.3 Hz, 1H), 7.46 – 7.30 (m, 2H), 7.29 – 7.15 (m, 2H), 6.81 (t, J = 1.6 Hz, 1H), 6.64 (t, J = 1.8 Hz, 1H), 6.53 (t, J = 2.0 Hz, 1H), 3.33 – 3.20 (m, 4H), 2.02 – 1.89 (m, 4H). ^{13}C NMR (100 MHz, DMSO-*d6*) δ 149.39, 143.08, 142.27, 141.88, 141.75, 138.46, 137.39, 134.76, 131.55, 130.46, 120.98, 120.83, 117.89, 117.29, 113.37, 110.48, 109.05, 47.84, 25.43.

2,2-dichloro-N-(3'-chloro-5'-(pyrrolidin-1-yl)-[1,1'-biphenyl]-3-yl)-N-(5-chloropyridin-3-yl)acetamide 34g (KK114):

According to the general procedure for the synthesis of Amidation, KK-114 compound was synthesized as a gummy solid (yield **67**%).

^{1}H NMR (400 MHz, $CDCl_3$) δ 7.74 (s, 1H), 7.64 (s, 1H), 7.58 – 7.41 (m, 2H), 7.26 (d, J = 8.2 Hz, 2H), 6.75 (t, J = 1.7 Hz, 1H), 6.54 (d, J = 2.2 Hz, 2H), 6.00 (s, 2H), 3.27 (q, J = 4.3, 3.3 Hz, 4H), 2.04 – 1.91 (m, 4H).

5-chloro-N-(1-(3,4-dichlorophenyl)azetidin-3-yl)pyridin-3-amine (33h):

According to the general procedure for the synthesis of Buchwald coupling (3b-k), 33h compound was

synthesized as a white solid (yield 78%).

2,2-dichloro-N-(5-chloropyridin-3-yl)-N-(1-(3,4-dichlorophenyl)azetidin-3-yl)acetamide 34h (VB-D-37):

According to the general procedure for the synthesis of Amidation, VB-D-37 compound was synthesized as a gummy solid (yield 62%).

^{1}H NMR (400 MHz, DMSO-*d6*) δ 7.88 (d, J = 2.4 Hz, 1H), 7.78 – 7.66 (m, 3H), 7.37 (dd, J = 8.6, 2.5 Hz, 1H), 6.89 (d, J = 2.4 Hz, 1H), 6.44 (s, 1H), 6.26 (d, J = 8.7 Hz, 1H), 3.99 (p, J = 6.5 Hz, 2H), 3.80 (dt, J = 11.3, 3.2 Hz, 2H), 3.68 (dd, J = 11.4, 5.0 Hz, 1H). ^{13}C NMR (101 MHz, DMSO-*d6*) δ 164.14, 145.01, 140.17, 135.56, 134.67, 132.57, 132.24, 132.03, 131.71, 131.01, 129.03, 117.11, 66.31, 65.96, 52.52, 51.62, 46.37. ESI-HRMS calculated for C17H14Cl5N3O (M+H+): 436.9549; found: 437.9260.

5-chloro-N-(dibenzo[b,d]furan-2-yl)pyridin-3-amine (33i):

According to the general procedure for the synthesis of Buchwald coupling (3b-k), 33i compound was synthesized as a white solid (yield 88%).

^{1}H NMR (400 MHz, DMSO-*d6*) δ 8.69 (s, 1H), 8.29 (s, 1H), 8.18 (d, J = 7.6 Hz, 1H), 7.97 (s, 2H), 7.66 (t, J = 16.5 Hz, 2H), 7.52 (t, J = 7.7 Hz, 1H), 7.38 (t, J = 4.6 Hz, 2H), 7.32 (dd, J = 8.7, 1.8 Hz, 1H). ^{13}C NMR (101 MHz, DMSO-*d6*) δ 156.52, 152.00, 143.36, 137.62, 137.07, 136.33, 131.67, 128.18, 124.94, 124.03, 123.38, 121.99, 121.57, 119.55, 112.97, 112.68, 112.11.

2,2-dichloro-N-(5-chloropyridin-3-yl)-N-(dibenzo[b,d]furan-2-yl)acetamide 34i (SR-B-101):

According to the general procedure for the synthesis of Amidation, SR-B-101 compound was synthesized as a gummy solid (yield 65%).

^{1}H NMR (400 MHz, $CDCl_3$) δ 8.38 (s, 2H), 7.89 (d, J = 7.8 Hz, 2H), 7.74 (s, 1H), 7.65 (d, J = 8.5 Hz, 1H), 7.57 (d, J = 8.2 Hz, 1H), 7.49 (t, J = 7.7 Hz, 1H), 7.35 (dd, J = 13.5, 6.2 Hz, 2H), 5.98 (s, 1H). HRMS(ESI) calculated for C19H11Cl3N2O2 (M+H+): 404.9900; found: 404.9957.

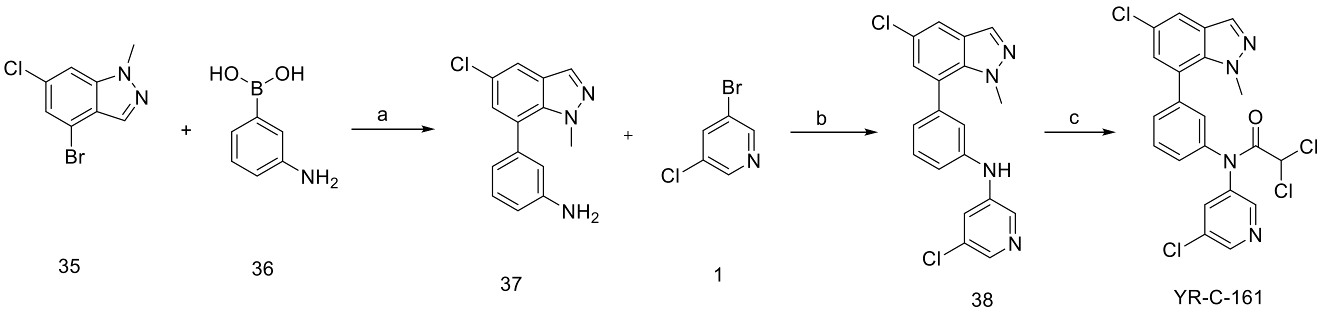

Synthesis Scheme S16. Synthesis of YR-C-161. Reagents and conditions: (a) Na_2CO_3, Pd(dppf)Cl_2, 1,4-Dioxane:H_2O, 100 ^{0}C, uw, 1h; (b) CS_2CO_3, $Pd_2(dba)_3$, Dppf, Toluene, 100 °C, 24 h; (b) Py, DMAP, Dichloroacetyl chloride, 1,4-Dioxane, 60 °C, 1 h.

3-(5-chloro-1-methyl-1H-indazol-7-yl)aniline (37):

To a stirred solution of compound 35 (1 equiv.), boronic acid 36 (1.2 equiv.), Na_2CO_3 (2 equiv.) in 1, 4-dioxane and water was added Pd(dppf)Cl2 (0.1 equiv.) and degas with nitrogen for 5 minutes and heated in microwave at 100 °C for 1 h. The catalyst was removed by filtration, and the filtrate was evaporated to dryness. Purification by column chromatography on silica (0- 100% EtOAc in Hexanes as the eluent) afforded the title compound 37 in a good yield. (86%).

^{1}H NMR (400 MHz, DMSO-*d6*) δ 8.13 (d, J = 1.0 Hz, 1H), 7.80 (dd, J = 1.6, 1.0 Hz, 1H), 7.22 – 7.14 (m, 2H), 6.96 (t, J = 2.0 Hz, 1H), 6.85 (ddd, J = 7.6, 1.8, 1.0 Hz, 1H), 6.66 (ddd, J = 8.0, 2.3, 1.0 Hz, 1H), 5.27 (s, 2H), 4.07 (s, 3H). ^{13}C NMR (101 MHz, DMSO-*d6*) δ 149.79, 141.02, 138.69, 137.08, 132.63, 131.78, 130.08, 120.86, 119.70, 116.13, 114.55, 114.00, 108.63, 36.16.

5-chloro-N-(3-(5-chloro-1-methyl-1H-indazol-7-yl)phenyl)pyridin-3-amine (38):

According to the general procedure for the synthesis of Buchwald coupling (3b-k), 38 compound was synthesized as a white solid (yield 85%).

^{1}H NMR (400 MHz, DMSO-*d6*) δ 8.81 (s, 1H), 8.35 (d, J = 2.4 Hz, 1H), 8.18 (s, 1H), 8.05 (d, J = 2.1 Hz, 1H), 7.86 (s, 1H), 7.56 (t, J = 2.3 Hz, 1H), 7.52 – 7.42 (m, 2H), 7.32 – 7.24 (m, 3H), 4.08 (s, 3H). ^{13}C NMR (101 MHz, DMSO-*d6*) δ 142.63, 141.62, 141.03, 139.34, 138.77, 137.68, 135.93, 132.35, 131.84, 131.58, 130.77, 121.90, 121.49, 120.81, 120.20, 118.29, 118.12, 109.21, 36.21.

2,2-dichloro-N-(3-(5-chloro-1-methyl-1H-indazol-7-yl)phenyl)-N-(5-chloropyridin-3-yl)acetamide (YR-C-161):

According to the general procedure for the synthesis of Amidation**,** YR-C-161 compound was synthesized as a gummy solid (yield **65**%).

^{1}H NMR (400 MHz, DMSO-*d6*) δ 8.64 (s, 1H), 8.29 (t, J = 2.1 Hz, 1H), 8.08 (d, J = 13.9 Hz, 2H), 7.82 (dt, J = 16.1, 1.3 Hz, 2H), 7.76 – 7.52 (m, 3H), 7.32 (s, 1H), 6.65 (s, 1H), 4.02 (s, 3H). HRMS(ESI) calculated for C21H14Cl4N4O (M+H+): 478.9900; found: 478.9925.

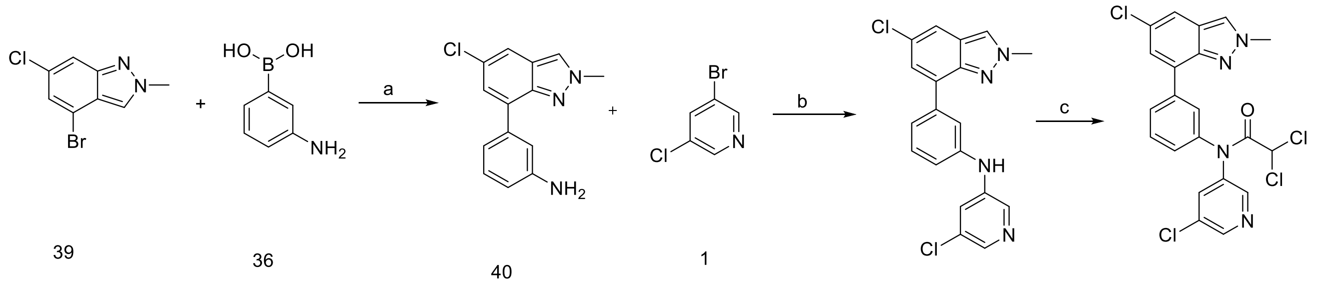

Synthesis Scheme S17. Synthesis of YR-C-163. Reagents and conditions: (a) Na_2CO_3, Pd(dppf)Cl_2, 1,4-Dioxane:H_2O, 100 ^{0}C, uw, 1h; (b) CS_2CO_3, $Pd_2(dba)_3$, Dppf, Toluene, 100 °C, 24 h; (b) Py, DMAP, Dichloroacetyl chloride, 1,4-Dioxane, 60 °C, 1 h.

3-(5-chloro-2-methyl-2H-indazol-7-yl)aniline (40):

The same procedure for the synthesis of compound 37 was followed except that 35 was replaced with 39 to afford compound 40 as a white solid (yield 80%).

^{1}H NMR (400 MHz, DMSO-*d6*) δ 8.49 (s, 1H), 7.65 – 7.60 (m, 1H), 7.15 (t, J = 7.8 Hz, 1H), 7.02 (d, J = 1.7 Hz, 1H), 6.91 (t, J = 2.0 Hz, 1H), 6.84 (dt, J = 7.8, 1.2 Hz, 1H), 6.68 – 6.60 (m, 1H), 5.22 (s, 2H), 4.17 (s, 3H). ^{13}C NMR (101 MHz, DMSO-*d6*) δ 149.74, 149.11, 139.35, 137.20, 130.80, 129.96, 125.99, 120.02, 119.49, 115.76, 114.76, 114.46, 113.56, 60.23.

5-chloro-N-(3-(5-chloro-2-methyl-2H-indazol-7-yl)phenyl)pyridin-3-amine (41):

According to the general procedure for the synthesis of Buchwald coupling (3b-k), 41 compound was synthesized as a white solid (yield 75%).

^{1}H NMR (400 MHz, DMSO-*d6*) δ 8.73 (s, 1H), 8.52 – 8.47 (m, 1H), 8.27 (q, J = 1.9 Hz, 3H), 7.97 (d, J = 2.1 Hz, 1H), 7.65 – 7.59 (m, 2H), 7.50 (d, J = 2.3 Hz, 1H), 7.40 (t, J = 7.8 Hz, 1H), 7.35 (t, J = 2.0 Hz, 1H), 7.25 (ddd, J = 7.7, 1.7, 1.0 Hz, 1H), 7.21 – 7.15 (m, 1H), 7.07 (d, J = 1.7 Hz, 1H), 4.12 (s, 3H). ^{13}C NMR (101 MHz, DMSO-*d6*) δ 149.09, 149.01, 145.89, 144.03, 143.44, 143.13, 142.56, 141.66, 140.77, 140.01, 138.73, 137.65, 136.07, 135.14, 131.75, 131.58, 130.85, 130.82, 130.68, 130.04, 125.80, 125.03, 121.58, 121.36, 121.04, 120.66, 119.44, 119.31, 118.16, 117.82, 115.35, 40.69.

2,2-dichloro-N-(3-(5-chloro-2-methyl-2H-indazol-7-yl)phenyl)-N-(5-chloropyridin-3-yl)acetamide (YR-C-163):

According to the general procedure for the synthesis of Amidation, YR-C-163 compound was synthesized as a gummy solid (yield 67%).

^{1}H NMR (400 MHz, $CDCl_3$) δ 8.45 (d, J = 23.1 Hz, 2H), 7.96 (s, 1H), 7.91 – 7.47 (m, 4H), 7.42 (s, 1H), 7.12 (d, J = 1.6 Hz, 1H), 6.12 (s, 1H), 4.22 (s, 3H). HRMS (ESI): m/z=480.9963 [M+H]+. HRMS(ESI) calculated for C21H14Cl4N4O (M+H+): 478.9900; found: 478.9937.

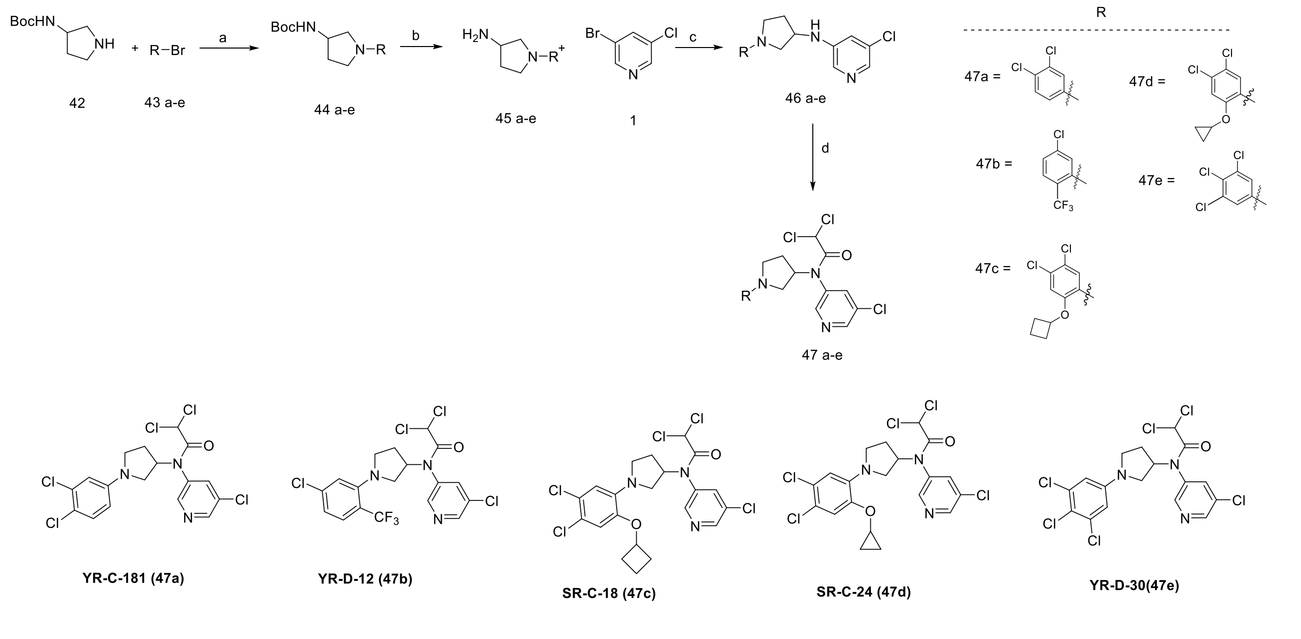

Synthesis Scheme S18. The synthesis of YR-C-181 (47a), YR-D-12 (47b), SR-C-18 (47c), SR-C-24 (47d), and YR-D-30 (47c). Reagents and conditions: (a) 43 a-e, CS_2CO_3, $Pd_2(dba)_3$, Dppf, Toluene, 100 °C, 24 h; (b) TFE, TMS-Cl, 0 ^{0}C to rt, 2h. (c) 1, *t*-BuONa, BrettPhos Pd G3, Toluene, uw, 140 °C, 1 h; (d) Py, DMAP, Dichloroacetyl chloride, 1,4-Dioxane, 60 °C, 1 h.

General procedure for Buchwald coupling (46a-e):

To a solution of R-NH_2 45a-e (0.5 mmol), 3-bromo-5-chloropyridine (0.75 mmol) in dry toluene was added t-BuONa (2 eq, 1.0 mmol). The vessel was capped with a septum and degassed with nitrogen for 5 min. Then BrettPhos PdG3 (0.05 equivalent, 0.025 mmol) was added and again degas it for 5 min and heated at 140 ^{0}C in microwave for 1 hour. After the reaction is completed, filter the mixture on celite and wash with ethyl acetate (20 mL). Remove the mixture solvent under vacuum. Purify the residue by flash chromatography (0-100% EA in Hexane as the eluent) yielded resulting products in good yields.

tert-butyl (1-(3,4-dichlorophenyl)pyrrolidin-3-yl)carbamate (44a):

According to the general procedure for the synthesis of Buchwald coupling (3b-k), 44a compound was synthesized as a white solid (yield 85%).

^{1}H NMR (400 MHz, DMSO-*d6*) δ 7.32 (d, J = 8.9 Hz, 1H), 7.23 – 7.15 (m, 1H), 6.66 (d, J = 2.8 Hz, 1H), 6.49 (dd, J = 9.0, 2.9 Hz, 1H), 4.13 (h, J = 6.5 Hz, 1H), 3.44 (dd, J = 9.9, 6.5 Hz, 1H), 3.36 (d, J = 6.8 Hz, 1H), 3.22 (ddd, J = 9.5, 7.8, 6.0 Hz, 1H), 3.03 (dd, J = 9.9, 4.9 Hz, 1H), 2.13 (ddt, J = 12.4, 7.7, 6.2 Hz, 1H), 1.88 (ddt, J = 12.4, 7.8, 6.1 Hz, 1H), 1.40 (s, 9H).

5-chloro-N-(1-(3,4-dichlorophenyl)pyrrolidin-3-yl)pyridin-3-amine (46a):

According to the general procedure for Buchwald coupling (46a-e), 46a compound was synthesized as a white solid (yield 85%).

^{1}H NMR (400 MHz, DMSO-*d6*) δ 7.96 (d, J = 2.5 Hz, 1H), 7.77 (d, J = 2.1 Hz, 1H), 7.34 (d, J = 8.9 Hz, 1H), 7.06 (t, J = 2.3 Hz, 1H), 6.73 (d, J = 2.8 Hz, 1H), 6.60 – 6.50 (m, 2H), 4.19 (h, J = 5.4 Hz, 1H), 3.63 (dd, J = 10.1, 6.0 Hz, 1H), 3.47 – 3.36 (m, 1H), 3.33 – 3.26 (m, 1H), 3.11 (dd, J = 10.1, 3.9 Hz, 1H), 2.36 – 2.23 (m, 1H), 1.95 (tt, J = 10.0, 3.7 Hz, 1H).

2,2-dichloro-N-(5-chloropyridin-3-yl)-N-(1-(3,4-dichlorophenyl)pyrrolidin-3-yl)acetamide 47a (YR-C-181):

According to the general procedure for the synthesis of Amidation, YR-C-181 compound was synthesized as a gummy solid (yield 62%).

^{1}H NMR (400 MHz, DMSO-*d6*) δ 8.89 – 8.46 (m, 2H), 8.20 (d, J = 48.0 Hz, 1H), 7.34 (d, J = 8.8 Hz, 1H), 6.63 (d, J = 2.8 Hz, 1H), 6.46 – 6.36 (m, 2H), 5.19 (p, J = 6.0 Hz, 1H), 3.59 (dd, J = 10.9, 6.6 Hz, 1H), 3.48 (s, 2H), 3.13 (q, J = 8.1 Hz, 1H), 2.81 (s, 1H), 2.37 (dt, J = 14.0, 6.8 Hz, 1H), 2.00 (dt, J = 13.5, 7.1 Hz, 1H). HRMS(ESI) calculated for C17H14Cl5N3O (M+H+): 451.9600; found: 451.9624.

tert-butyl (1-(5-chloro-2-(trifluoromethyl)phenyl)pyrrolidin-3-yl)carbamate (44b):

According to the general procedure for the synthesis of Buchwald coupling (3b-k), 44b compound was synthesized as a white solid (yield 80%).

^{1}H NMR (400 MHz, DMSO-*d6*) δ 7.57 (d, J = 8.6 Hz, 1H), 7.19 (d, J = 6.5 Hz, 1H), 6.99 (d, J = 2.0 Hz, 1H), 6.91 (dd, J = 8.5, 1.9 Hz, 1H), 4.10 – 3.99 (m, 1H), 3.57 – 3.47 (m, 1H), 3.41 (t, J = 8.2 Hz, 1H), 3.36 (s, 1H), 3.16 (dd, J = 10.0, 5.0 Hz, 1H), 2.14 – 2.02 (m, 1H), 1.85 (dq, J = 12.7, 6.4 Hz, 1H), 1.39 (s, 9H).

5-chloro-N-(1-(5-chloro-2-(trifluoromethyl)phenyl)pyrrolidin-3-yl)pyridin-3-amine (46b):

According to the general procedure for Buchwald coupling (46a-e), 46b compound was synthesized as a white solid (yield 75%).

^{1}H NMR (400 MHz, DMSO-*d6*) δ 7.95 (d, J = 2.5 Hz, 1H), 7.76 (d, J = 2.0 Hz, 1H), 7.59 (d, J = 8.6 Hz, 1H), 7.06 (q, J = 2.1 Hz, 2H), 6.93 (dd, J = 8.5, 2.0 Hz, 1H), 6.53 (d, J = 6.8 Hz, 1H), 4.14 (h, J = 5.8 Hz, 1H), 3.72 (dd, J = 10.0, 6.1 Hz, 1H), 3.50 (dt, J = 9.6, 7.0 Hz, 1H), 3.40 (dt, J = 9.6, 6.8 Hz, 1H), 3.19 (dd, J = 10.0, 4.4 Hz, 1H), 2.25 (dq, J = 12.9, 6.7 Hz, 1H), 1.90 (dq, J = 12.2, 6.0 Hz, 1H).

2,2-dichloro-N-(1-(5-chloro-2-(trifluoromethyl)phenyl)pyrrolidin-3-yl)-N-(5-chloropyridin-3-yl)acetamide 47b (YR-D-12):

According to the general procedure for the synthesis of Amidation**, YR-D-12** compound was synthesized as a gummy solid (yield **65**%).

^{1}H NMR (400 MHz, DMSO-*d6*) δ 8.69 (d, J = 2.2 Hz, 1H), 8.59 (d, J = 18.0 Hz, 1H), 8.16 (d, J = 18.3 Hz, 1H), 7.51 (d, J = 8.5 Hz, 1H), 6.92 (d, J = 8.0 Hz, 2H), 6.30 (s, 1H), 5.06 (s, 1H), 3.59 (dd, J = 10.8, 6.6 Hz, 1H), 3.15 (dd, J = 79.0, 49.0 Hz, 3H), 2.25 (dq, J = 13.6, 6.9 Hz, 1H), 1.93 (d, J = 39.4 Hz, 1H). HRMS(ESI) calculated for C18H14Cl4F3N3O (M+H+): 485.9800; found: 485.9843.

tert-butyl (1-(4,5-dichloro-2-cyclobutoxyphenyl)pyrrolidin-3-yl)carbamate (44c):

According to the general procedure for the synthesis of Buchwald coupling (3b-k), 44c compound was synthesized as a white solid (yield 82%).

^{1}H NMR (400 MHz, $CDCl_3$) δ 7.19 (s, 1H), 6.59 (s, 1H), 5.23 (s, 1H), 4.71 (s, 1H), 4.59 – 4.38 (m, 1H), 4.22 (s, 1H), 3.42 (dd, J = 10.0, 6.1 Hz, 2H), 3.13 (dd, J = 48.5, 6.4 Hz, 2H), 2.49 – 2.31 (m, 2H), 2.20 – 2.12 (m, 2H), 1.88 – 1.73 (m, 2H), 1.69 – 1.58 (m, 1H), 1.38 (s, 9H).

5-chloro-N-(1-(4,5-dichloro-2-cyclobutoxyphenyl)pyrrolidin-3-yl)pyridin-3-amine (46c):

According to the general procedure for Buchwald coupling (46a-e), 46c compound was synthesized as a white solid (yield 78%).

^{1}H NMR (400 MHz, $CDCl_3$) δ 7.87 (d, J = 7.7 Hz, 2H), 6.84 (s, 1H), 6.66 (s, 1H), 6.61 (s, 1H), 4.50 (p, J = 7.1 Hz, 1H), 4.13 (s, 1H), 4.07 – 4.00 (m, 1H), 3.55 – 3.43 (m, 2H), 3.32 (dd, J = 10.1, 2.5 Hz, 1H), 3.22 (td, J = 8.9, 5.7 Hz, 1H), 2.44 – 2.33 (m, 2H), 2.27 (dt, J = 13.9, 7.0 Hz, 1H), 2.13 – 1.99 (m, 2H), 1.94 – 1.73 (m, 2H), 1.62 (dt, J = 18.8, 9.4 Hz, 1H).

2,2-dichloro-N-(5-chloropyridin-3-yl)-N-(1-(4,5-dichloro-cyclobutoxyphenyl) pyrrolidin-3-yl)acetamide 47c (SR-C-18):

According to the general procedure for the synthesis of Amidation, SR-C-18 compound was synthesized as a gummy solid (yield 55%).

^{1}H NMR (400 MHz, $CDCl_3$) δ 8.62 (s, 1H), 8.41 (s, 1H), 7.67 (d, J = 53.5 Hz, 1H), 6.58 (s, 2H), 5.56 (s, 1H), 5.15 (s, 1H), 4.48 (d, J = 6.5 Hz, 1H), 3.43 (dd, J = 39.8, 33.5 Hz, 2H), 3.10 (d, J = 50.0 Hz, 2H),

2.47 – 2.31 (m, 2H), 2.21 (d, J = 7.1 Hz, 1H), 2.13 – 1.95 (m, 2H), 1.89 – 1.71 (m, 2H), 1.64 (dd, J = 19.1, 8.5 Hz, 1H). HRMS(ESI) calculated for C21H20Cl5N3O2 (M+H+): 522.0000; found: 522.0074.

tert-butyl (1-(4,5-dichloro-2-cyclopropoxyphenyl)pyrrolidin-3-yl)carbamate (44d):

According to the general procedure for the synthesis of Buchwald coupling (3b-k), 44d compound was synthesized as a white solid (yield 76%).

^{1}H NMR (400 MHz, $CDCl_3$) δ 7.14 (d, J = 6.3 Hz, 1H), 6.70 – 6.53 (m, 1H), 5.23 (s, 1H), 4.69 (s, 1H), 4.20 (s, 1H), 3.69 – 3.56 (m, 1H), 3.38 (dd, J = 10.2, 6.1 Hz, 2H), 3.12 (d, J = 7.6 Hz, 2H), 2.23 – 2.09 (m, 1H), 1.76 (d, J = 5.5 Hz, 1H), 1.38 (s, 9H), 0.79 – 0.70 (m, 4H).

5-chloro-N-(1-(4,5-dichloro-2-cyclopropoxyphenyl)pyrrolidin-3-yl)pyridin-3-amine (46d):

According to the general procedure for Buchwald coupling (46a-e), 46d compound was synthesized as a white solid (yield 79%).

^{1}H NMR (400 MHz, $CDCl_3$) δ 7.85 (s, 2H), 7.19 (s, 2H), 6.82 (t, J = 2.2 Hz, 1H), 6.65 (s, 1H), 4.02 (d, J = 4.4 Hz, 1H), 3.68 – 3.55 (m, 1H), 3.51 – 3.36 (m, 2H), 3.28 – 3.05 (m, 2H), 2.25 (dt, J = 14.4, 6.4 Hz, 1H), 1.85 (dt, J = 12.4, 8.2 Hz, 1H), 0.79 – 0.60 (m, 4H).

2,2-dichloro-N-(5-chloropyridin-3-yl)-N-(1-(4,5-dichloro-2-cyclopropoxyphenyl)yrrolidine-3-yl)acetamide 47d (SR-C-24):

According to the general procedure for the synthesis of Amidation**,** SR-C-24 compound was synthesized as a gummy solid (yield 63%).

^{1}H NMR (400 MHz, $CDCl_3$) δ 8.61 (d, J = 1.5 Hz, 1H), 8.39 (s, 1H), 7.65 (d, J = 44.0 Hz, 1H), 7.13 (s, 1H), 6.53 (s, 1H), 5.55 (s, 1H), 5.12 (s, 1H), 3.54 (d, J = 31.6 Hz, 1H), 3.52 – 3.25 (m, 2H), 3.04 (d, J = 49.4 Hz, 2H), 2.19 (s, 1H), 1.75 (s, 1H), 0.78 – 0.63 (m, 4H). HRMS(ESI) calculated for C20H18Cl5N3O2 (M+H+): 507.9800; found: 507.9819.

tert-butyl (1-(3,4,5-trichlorophenyl)pyrrolidin-3-yl)carbamate (44e):

According to the general procedure for the synthesis of Buchwald coupling (3b-k), 44e compound was synthesized as a white solid (yield 75%).

^{1}H NMR (400 MHz, DMSO-*d6*) δ 7.20 (d, J = 6.9 Hz, 1H), 6.69 (s, 2H), 4.13 (h, J = 6.3 Hz, 1H), 3.46 (dd, J = 10.1, 6.4 Hz, 1H), 3.39 – 3.34 (m, 1H), 3.23 (ddd, J = 9.7, 7.8, 5.9 Hz, 1H), 3.04 (dd, J = 10.1, 4.8 Hz, 1H), 2.12 (ddt, J = 12.5, 7.9, 6.3 Hz, 1H), 1.89 (dq, J = 12.9, 6.1 Hz, 1H), 1.40 (s, 9H). ^{13}C NMR (101 MHz, DMSO-*d6*) δ 155.67, 147.17, 133.45, 114.78, 112.13, 78.36, 53.65, 50.33, 46.30, 31.08, 28.70.

5-chloro-N-(1-(3,4,5-trichlorophenyl)pyrrolidin-3-yl)pyridin-3-amine (46e):

According to the general procedure for Buchwald coupling (46a-e), 46e compound was synthesized as a white solid (yield 80%).

^{1}H NMR (400 MHz, DMSO-*d6*) δ 7.95 (d, J = 2.5 Hz, 1H), 7.77 (d, J = 2.1 Hz, 1H), 7.06 (t, J = 2.3 Hz,

1H), 6.78 (s, 2H), 6.53 (d, J = 6.9 Hz, 1H), 4.18 (h, J = 5.5 Hz, 1H), 3.64 (dd, J = 10.3, 5.9 Hz, 1H), 3.49 – 3.34 (m, 1H), 3.13 (dd, J = 10.3, 3.8 Hz, 1H), 2.28 (dq, J = 13.5, 7.2 Hz, 1H), 1.99 – 1.91 (m, 1H). ^{13}C NMR (101 MHz, DMSO-*d6*) δ 147.28, 145.54, 135.11, 134.73, 133.49, 131.75, 117.17, 114.89, 112.32, 60.23, 51.90, 46.36, 31.05.

2,2-dichloro-N-(5-chloropyridin-3-yl)-N-(1-(3,4,5-trichlorophenyl)pyrrolidin-3-yl)acetamide 47e (YR-D-30):

According to the general procedure for the synthesis of Amidation**,** YR-D-30 compound was synthesized as a gummy solid (yield 61%).

^{1}H NMR (400 MHz, DMSO-*d6*) δ 8.70 – 8.36 (m, 2H), 8.08 (d, J = 46.1 Hz, 1H), 6.53 (s, 2H), 6.27 (s, 1H), 5.07 (s, 1H), 3.54 – 3.30 (m, 2H), 3.02 (s, 1H), 2.65 (d, J = 36.5 Hz, 1H), 2.26 – 2.18 (m, 1H), 1.95 – 1.82 (m, 1H). HRMS(ESI) calculated for C17H13Cl6N3O (M+H+): 485.9200; found: 485.9213.

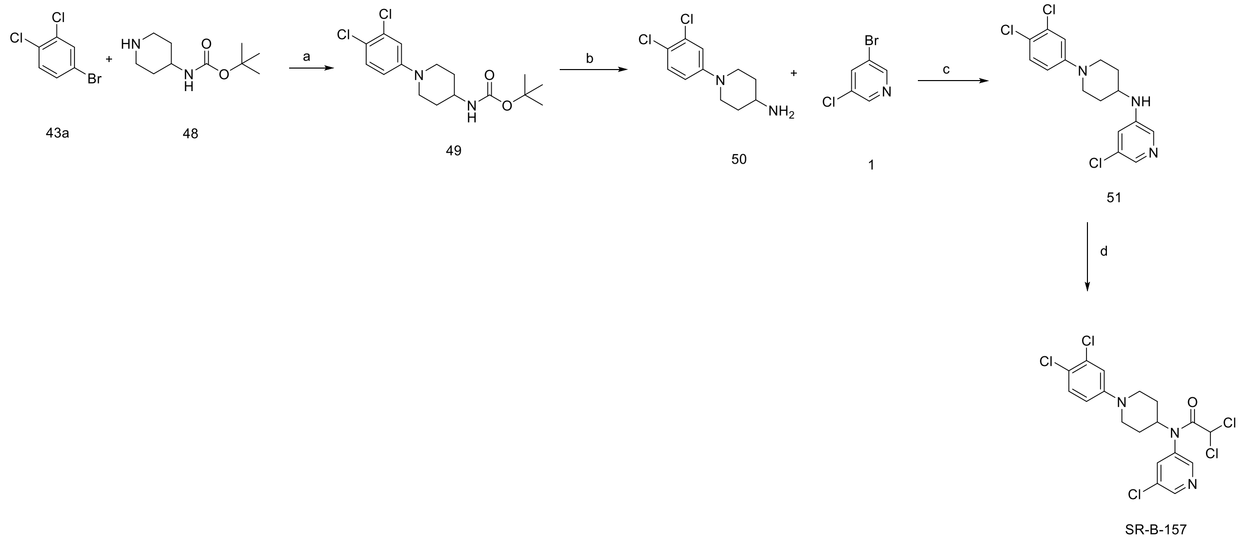

Synthesis Scheme S19. Synthesis of SR-B-157. Reagents and conditions: (a) CS_2CO_3, $Pd_2(dba)_3$, Dppf, Toluene, 100 °C, 24 h; (b) TFE, TMS-Cl, 0 ^{0}C to rt, 2h. (c) 1, *t*-BuONa, BrettPhos Pd G3, Toluene, uw, 140 °C, 1 h; (d) Py, DMAP, Dichloroacetyl chloride, 1,4-Dioxane, 60 °C, 1 h.

tert-butyl (1-(3,4-dichlorophenyl)piperidin-4-yl)carbamate (49):

According to the general procedure for the synthesis of Buchwald coupling (3b-k), 49 compound was synthesized as a white solid (yield 81%).

^{1}H NMR (400 MHz, $CDCl_3$) δ 7.17 (t, J = 6.8 Hz, 1H), 6.87 (d, J = 2.8 Hz, 1H), 6.65 (dd, J = 8.9, 2.8 Hz, 1H), 4.44 (s, 1H), 3.52 (d, J = 7.4 Hz, 1H), 3.47 (d, J = 12.9 Hz, 2H), 2.87 – 2.64 (m, 2H), 1.39 (d, J = 10.4 Hz, 9H), 0.84 – 0.74 (m, 4H).

5-chloro-N-(1-(3,4-dichlorophenyl)piperidin-4-yl)pyridin-3-amine (51):

According to the general procedure for Buchwald coupling (46a-e), 51 compound was synthesized as a white solid (yield 71%).

^{1}H NMR (400 MHz, $CDCl_3$) δ 7.17 (t, J = 6.8 Hz, 1H), 6.87 (d, J = 2.8 Hz, 1H), 6.65 (dd, J = 8.9, 2.8 Hz, 1H), 4.44 (s, 1H), 3.52 (d, J = 7.4 Hz, 1H), 3.47 (d, J = 12.9 Hz, 2H), 2.87 – 2.64 (m, 2H), 1.39 (d, J = 10.4 Hz, 9H), 0.84 – 0.74 (m, 4H). 1H NMR (400 MHz, CDCl3) δ 7.82 (s, 2H), 7.19 (d, J = 2.0 Hz, 1H), 6.91 (d, J = 2.9 Hz, 1H), 6.80 (t, J = 2.2 Hz, 1H), 6.69 (dd, J = 8.9, 2.9 Hz, 1H), 3.85 – 3.73 (m, 1H), 3.61 – 3.48 (m, 2H), 3.42 – 3.29 (m, 1H), 2.92 – 2.80 (m, 2H), 0.80 (dt, J = 5.9, 5.2 Hz, 4H).

2,2-dichloro-N-(5-chloropyridin-3-yl)-N-(1-(3,4-dichlorophenyl)piperidin-4-yl)acetamide (SR-B-157):

According to the general procedure for the synthesis of Amidation**,** SR-B-157 compound was synthesized as a gummy solid (yield 65%).

^{1}H NMR (400 MHz, DMSO-*d6*) δ 8.77 (d, J = 1.8 Hz, 1H), 8.52 (d, J = 1.6 Hz, 1H), 8.12 (s, 1H), 7.33 (d, J = 9.0 Hz, 1H), 7.08 (d, J = 2.9 Hz, 1H), 6.88 (dd, J = 9.0, 2.9 Hz, 1H), 6.31 (s, 1H), 4.50 (t, J = 12.3 Hz, 1H), 3.77 (d, J = 12.5 Hz, 2H), 2.80 (t, J = 12.2 Hz, 2H), 1.98 – 1.81 (m, 2H), 1.25 (d, J = 12.2 Hz, 2H). HRMS(ESI) calculated for C18H16Cl5N3O (M+H+): 465.9700; found: 465.9703.

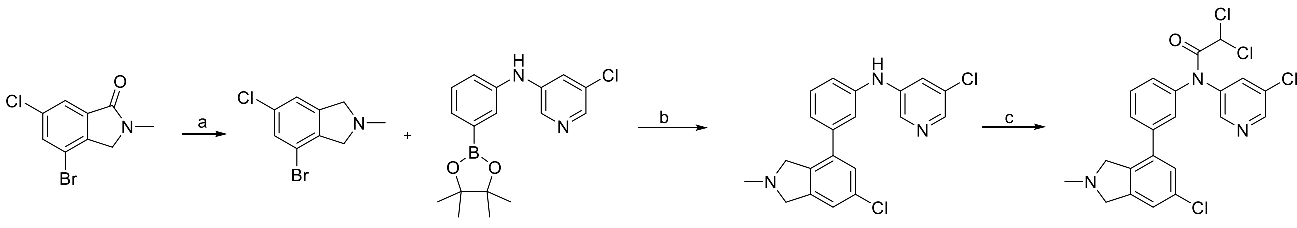

52 53 54 55 NS-A-101

Synthesis Scheme S20. Synthesis of NS-A-101. Reagents and conditions: (a) $NaBH_4$, $BF_3.Et_2O$, THF, 0 °C to 35 °C, 18 h; (b) Pd(dppf)Cl_2, Na_2CO_3, 1,4-Dioxane:H_2O, 100 ^{0}C to *u*w, 1h. (c) Py, DMAP, Dichloroacetyl chloride, 1,4-Dioxane, 60 °C, 1 h.

General procedure for Suzuki coupling:

To a stirred solution of bromo compounds (1 equiv.), boronic acid intermediates or borates (1.2 equiv.), Na_2CO_3 (2 equiv.) in 1, 4-dioxane and water was added Pd(dppf)Cl_2 (0.1 equiv.) and degas with nitrogen for 5 minutes and heated in microwave at 100 °C for 1 h. The catalyst was removed by filtration, and the filtrate was evaporated to dryness. Purification by column chromatography on silica (0- 100% EtOAc in Hexanes as the eluent) afforded the title compounds in a good yield (80%).

4-bromo-6-chloro-2-methylisoindoline (53):

The compound 52 (3 g, 11.58 mmol) was dissolved in THF (24 mL) and cool to the 0 °C. The Sodium borohydride (1.98 g, 52.12 mmol) was added to above reaction mixture in three portions followed by $BF_3.Et_2O$ (7.14 mL, 57.9 mmol) was added to the mixture over a period of 30 min to control the exotherm and keep the internal temperature below 30 °C. After complete addition of the $BF_3.Et_2O$, the mixture was warmed to 35 °C over 1.5 h and stirred for a period of 18 h. The mixture was slowly poured on 3 N HCl (24 mL) over a period of 30 min, while under a nitrogen atmosphere. The mixture was warmed to 50 °C and stirred for a period of 2 h. The mixture was cooled to 25 °C and diluted with ethyl acetate. The layers were separated, and the aqueous layer was back extracted with ethyl acetate (1x50 mL), combined organic layers and washed with 1N HCl twice. Combined aqueous layers were basified to pH 14 using 10 N NaOH, then extracted with ethyl acetate (2 x 50 mL). Combined organic layers

were washed with brine (2x50 mL). The organic layer was dried with Na_2SO_4, filtered and concentrated under reduced pressure to get crude product which was purified by column chromatography by using 0-50 % ethyl acetate and hexane to get pure 53 (2.27g, 80%) as a white solid.

^{1}H NMR (400 MHz, DMSO-*d6*) δ 7.53 – 7.51 (m, 1H), 7.36 (dd, J = 1.8, 0.9 Hz, 1H), 3.92 (t, J = 0.9 Hz, 2H), 3.79 (t, J = 1.9 Hz, 2H), 2.46 (s, 3H). 13C NMR (101 MHz, DMSO-*d6*) δ 144.60, 140.19, 132.29, 128.69, 121.95, 116.57, 60.87, 60.63, 41.46.

5-chloro-N-(3-(6-chloro-2-methylisoindolin-4-yl)phenyl)pyridin-3-amine (55):

According to the general procedure for Suzuki coupling, 55 compound was synthesized as a white solid (yield 85%).

^{1}H NMR (400 MHz, $CDCl_3$) δ 8.25 (d, J = 2.5 Hz, 1H), 8.11 (d, J = 2.0 Hz, 1H), 7.43 – 7.40 (m, 1H), 7.38 (d, J = 7.8 Hz, 1H), 7.20 (d, J = 1.8 Hz, 1H), 7.13 – 7.03 (m, 3H), 5.94 (s, 1H), 4.05 (s, 4H), 2.66 (s, 3H).

2,2-dichloro-N-(3-(6-chloro-2-methylisoindolin-4-yl)phenyl)-N-(5-chloropyridin-3-yl)acetamide (NS-A-101):

According to the general procedure for the synthesis of Amidation**,** NS-A-101 compound was synthesized as a white solid (yield 68%).

^{1}H NMR (400 MHz, $CDCl_3$) δ 8.43 (d, J = 29.4 Hz, 2H), 7.78 (d, J = 2.6 Hz, 1H), 7.57 (d, J = 36.8 Hz, 2H), 7.36 (s, 2H), 7.24 (s, 2H), 6.07 (s, 1H), 3.98 (d, J = 22.0 Hz, 4H), 2.62 (s, 3H). HRMS (ESI) calcd for C22H18Cl4N3O [M+H]+ , 480.0198; found, 480.0200.

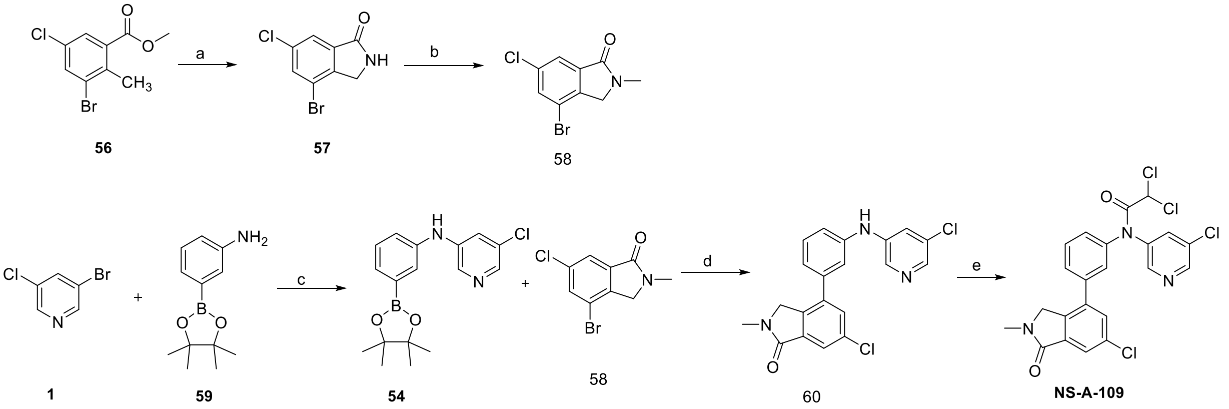

Synthesis Scheme S21. Synthesis of NS-A-109. Reagents and conditions: (a) i. NBS, AIBN, CCl_4, 90 °C 5 h ii. 7N NH_3, 90 °C 2 h (b) NaH, CH_3I, DMF, 0 °C to rt, 16 h (c) CS_2CO_3, $Pd_2(dba)_3$, Dppf, Toluene, 100 °C, 24 h (d) Pd(dppf)Cl_2, Na_2CO_3, 1,4-Dioxane:H_2O, 100 ^{0}C to *u*w, 1h. (e) Py, DMAP, Dichloroacetyl chloride, 1,4-Dioxane, 60 °C, 1 h.

4-bromo-6-chloroisoindolin-1-one (57):

To the stirred solution compound 56 (8.73 g, 33.19 mmol), N-bromosuccinimide (5.90 g, 33.19 mmol) in carbon tetrachloride (104 mL) at room temperature then azobisisobutyronitrile (5.45 g, 33.19 mmol) was added to the above reaction mixture at room temperature under nitrogen atmosphere and stirred at

90 °C for 5 h. The starting material consumption was confirmed by TLC. The system was cooled to room temperature and water was added to the reaction mixture, separated the organic and aqueous layer. The organic layer was washed with water (3x50 mL). The organic phase was dried over anhydrous sodium sulfate, filtered, and concentrated. This compound was used directly in the next step and dissolved in 7 M Ammonia/methanol solution (38.6 mL) in a sealed tube and the system was heated at 70° C for 2 h. A white solid was precipitated out, cooled to room temperature, filtered, and dried to give pure compound 57 (2.78 g, 50 %).

^{1}H NMR (400 MHz, DMSO-*d6*) δ 8.97 (s, 1H), 7.97 (s, 1H), 7.71 (s, 1H), 4.31 (s, 2H).

4-bromo-6-chloro-2-methylisoindolin-1-one (58):

Sodium hydride (60%) (0.898 g, 22.45 mmol) was suspended in DMF (51 mL) and then cooled to 0 °C. A solution of compound 57 (5.0 g, 20.41 mmol) in DMF (30 mL) was then added dropwise. The resulting mixture was stirred for 20-30 min until gas evolution ceased. Iodomethane (2.03 mL, 32.65 mmol) was added dropwise at that same temperature, the resulting mixture was warmed to room temperature and then stirred overnight. After consumption of the starting material (TLC control) the reaction mixture was poured into water. The mixture was extracted twice with MTBE (2X 50 mL). The organic phases were combined, washed with water, dried over sodium sulfate and concentrated to give crude compound which was purified by column chromatography by using ethyl acetate and hexane (30-35%) to get pure compound 58 (4.98 g, 94%).

^{1}H NMR (400 MHz, DMSO-*d6*) δ 7.96 (d, J = 1.7 Hz, 1H), 7.71 (d, J = 1.8 Hz, 1H), 4.40 (s, 2H), 3.08 (s, 3H).^{13}C NMR (101 MHz, DMSO-*d6)* δ 165.20, 140.82, 135.94, 133.93, 133.21, 121.97, 117.83, 51.64, 29.13.

5-chloro-N-(3-(4,4,5,5-tetramethyl-1,3,2-dioxaborolan-2-yl)phenyl)pyridin-3-amine (54):

According to the general procedure for the synthesis of Buchwald coupling (3b-k), 54 compound was synthesized as a white solid (yield 81%).

^{1}H NMR (400 MHz, $CDCl_3$) δ 8.20 (d, J = 2.4 Hz, 1H), 8.05 (d, J = 2.0 Hz, 1H), 7.55 – 7.49 (m, 2H), 7.35 (t, J = 7.7 Hz, 1H), 7.30 (t, J = 2.3 Hz, 1H), 7.26 – 7.22 (m, 1H), 5.84 (s, 1H), 1.34 (s, 12H).

6-chloro-4-(3-((5-chloropyridin-3-yl)amino)phenyl)-2-methylisoindolin-1-one (60):

According to the general procedure for Suzuki coupling, 55 compound was synthesized as a white solid (yield 75%).

^{1}H NMR (400 MHz, DMSO-*d6*) δ 8.79 (s, 1H), 8.32 (d, J = 2.4 Hz, 1H), 8.04 (d, J = 2.1 Hz, 1H), 7.71 (d, J = 2.0 Hz, 1H), 7.67 (d, J = 1.9 Hz, 1H), 7.54 (t, J = 2.3 Hz, 1H), 7.46 (t, J = 7.9 Hz, 1H), 7.35 (t, J = 2.0 Hz, 1H), 7.26 – 7.20 (m, 2H), 4.61 (s, 2H), 3.08 (s, 3H).

2,2-dichloro-N-(3-(6-chloro-2-methyl-1-oxoisoindolin-4-yl)phenyl)-N-(5-chloropyridin-3-yl)acetamide (NS-A-109):

According to the general procedure for the synthesis of Amidation**,** NS-A-109 compound was synthesized as a white solid (yield 62%).

^{1}H NMR (400 MHz, $CDCl_3$) δ 8.45 (d, J = 42.9 Hz, 2H), 7.89 – 7.77 (m, 2H), 7.75 – 7.38 (m, 5H), 6.07 (s, 1H), 4.39 (s, 2H), 3.20 (s, 3H). HRMS (ESI) calcd for C22H16Cl4N3O2 [M+H]+, 493.9991; found, 493.9988.

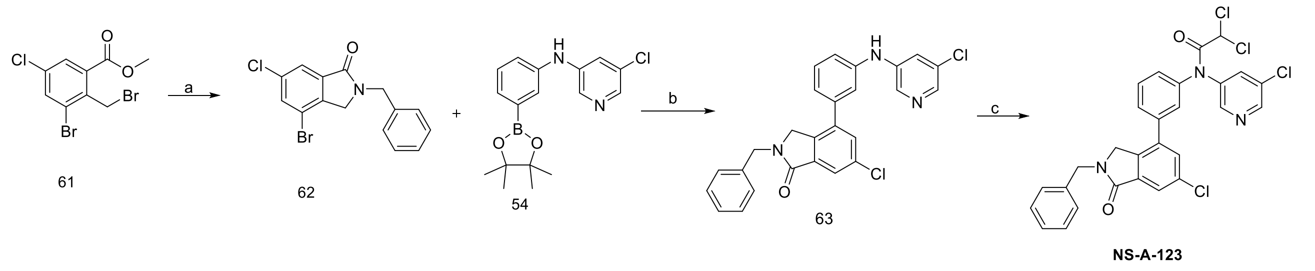

Synthesis Scheme S22. Synthesis of NS-A-123. Reagents and conditions: (a) Benzylamine, MeOH, 70 °C, 12 h; (b) Pd(dppf)Cl_2, Na_2CO_3, 1,4-Dioxane:H_2O, 100 ^{0}C to *u*w, 1h. (c) Py, DMAP, Dichloroacetyl chloride, 1,4-Dioxane, 60 °C, 1 h.

2-benzyl-4-bromo-6-chloroisoindolin-1-one (62):

Compound 61 (3.0 g, 8.74 mmol) was dissolved in methanol (10 mL) in a sealed tube, and benzylamine (11.22 g, 108.8 mmol) was added at room temperature. The reaction mixture was then heated to 70 °C and stirred for 12 h. Completion of the reaction was confirmed by TLC. The mixture was cooled to room temperature, and the solvent was evaporated under reduced pressure to obtain the crude product. The residue was purified by column chromatography using 0–50% ethyl acetate in hexane to afford the pure compound 62 (2.34 g, 80%) s a white solid.

^{1}H NMR (400 MHz, DMSO-*d6*) δ 7.98 (d, J = 1.8 Hz, 1H), 7.78 (d, J = 1.8 Hz, 1H), 7.41 – 7.25 (m, 5H), 4.74 (s, 2H), 4.30 (s, 2H).

2-benzyl-6-chloro-4-(3-((5-chloropyridin-3-yl)amino)phenyl)isoindolin-1-one (63):

According to the general procedure for Suzuki coupling, 63 compound was synthesized as a white solid (yield 83%).

^{1}H NMR (400 MHz, DMSO-*d6*) δ 8.76 (s, 1H), 8.29 (d, J = 2.4 Hz, 1H), 8.02 (d, J = 2.1 Hz, 1H), 7.73 (dd, J = 15.0, 2.0 Hz, 2H), 7.50 (t, J = 2.3 Hz, 1H), 7.41 (t, J = 7.9 Hz, 1H), 7.36 – 7.30 (m, 2H), 7.30 – 7.24 (m, 4H), 7.23 – 7.13 (m, 2H), 4.73 (s, 2H), 4.52 (s, 2H). ^{13}C NMR (101 MHz, DMSO-*d6*) δ 166.21, 142.14, 141.25, 138.68, 138.43, 138.08, 138.04, 137.22, 134.90, 133.77, 131.28, 130.92, 130.36, 128.83, 127.75, 127.54, 124.80, 121.91, 121.48, 121.06, 118.20, 117.69, 49.51, 45.76.

N-(3-(2-benzyl-6-chloro-1-oxoisoindolin-4-yl)phenyl)-2,2-dichloro-N-(5-chloropyridin-3-yl)acetamide (NS-A-123):

According to the general procedure for the synthesis of Amidation**,** NS-A-123 compound was synthesized as a white solid (yield 66%)^{1}H NMR (400 MHz, DMSO-*d6*) δ 8.63 (d, J = 23.7 Hz, 2H), 8.16 (s, 1H), 8.01 (s, 1H), 7.89 – 7.55 (m, 5H), 7.36 – 7.31 (m, 2H), 7.30 – 7.24 (m, 3H), 6.62 (s, 1H), 4.72 (s, 2H), 4.58 (s, 2H). HRMS (ESI) calcd for C28H20Cl4N3O2 [M+H]+ , 570.0304; found, 570.0303.

5. Representative NMR spectra (Synthesis Figs. S1-S112)

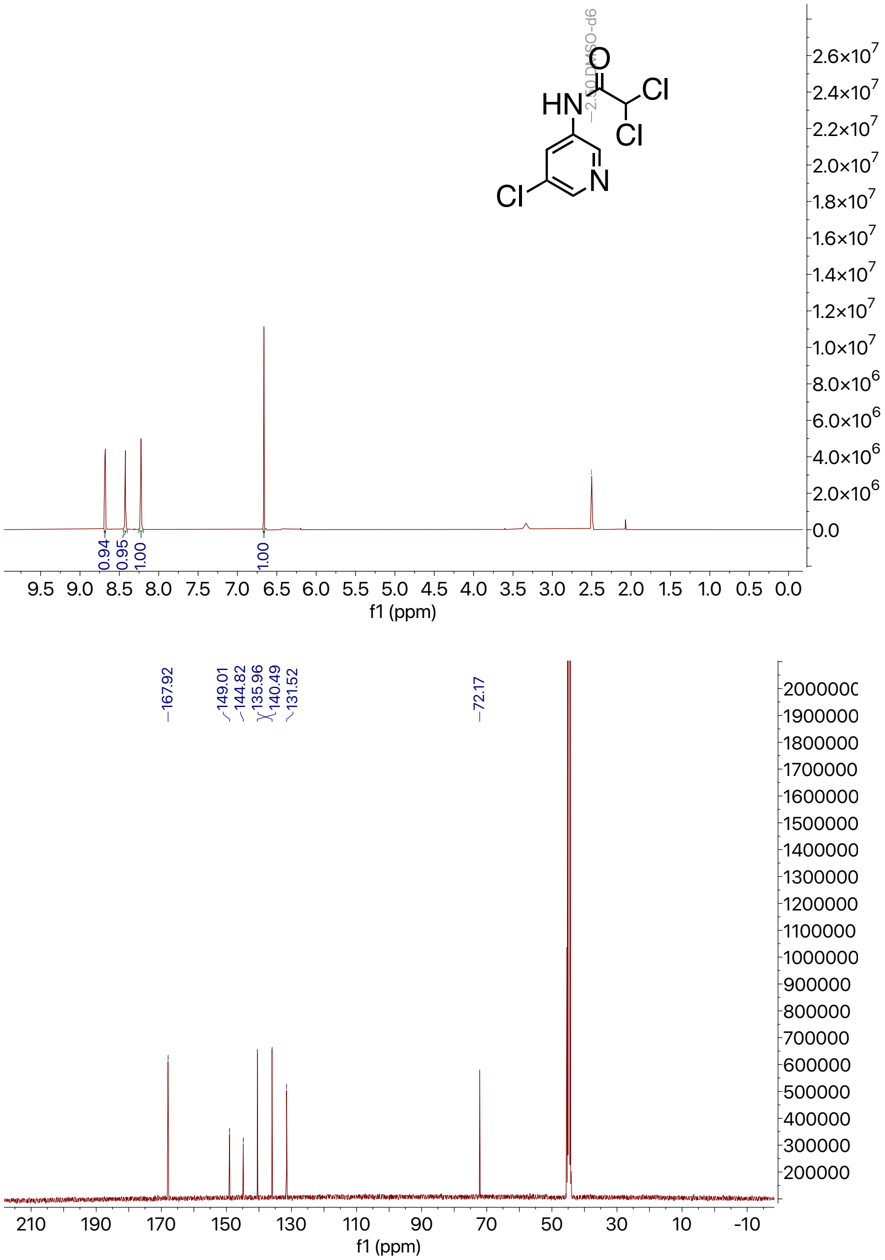

Synthesis Fig. S1. ^{1}H NMR and ^{13}C NMR spectra of VB-B-113 in DMSO-d_6

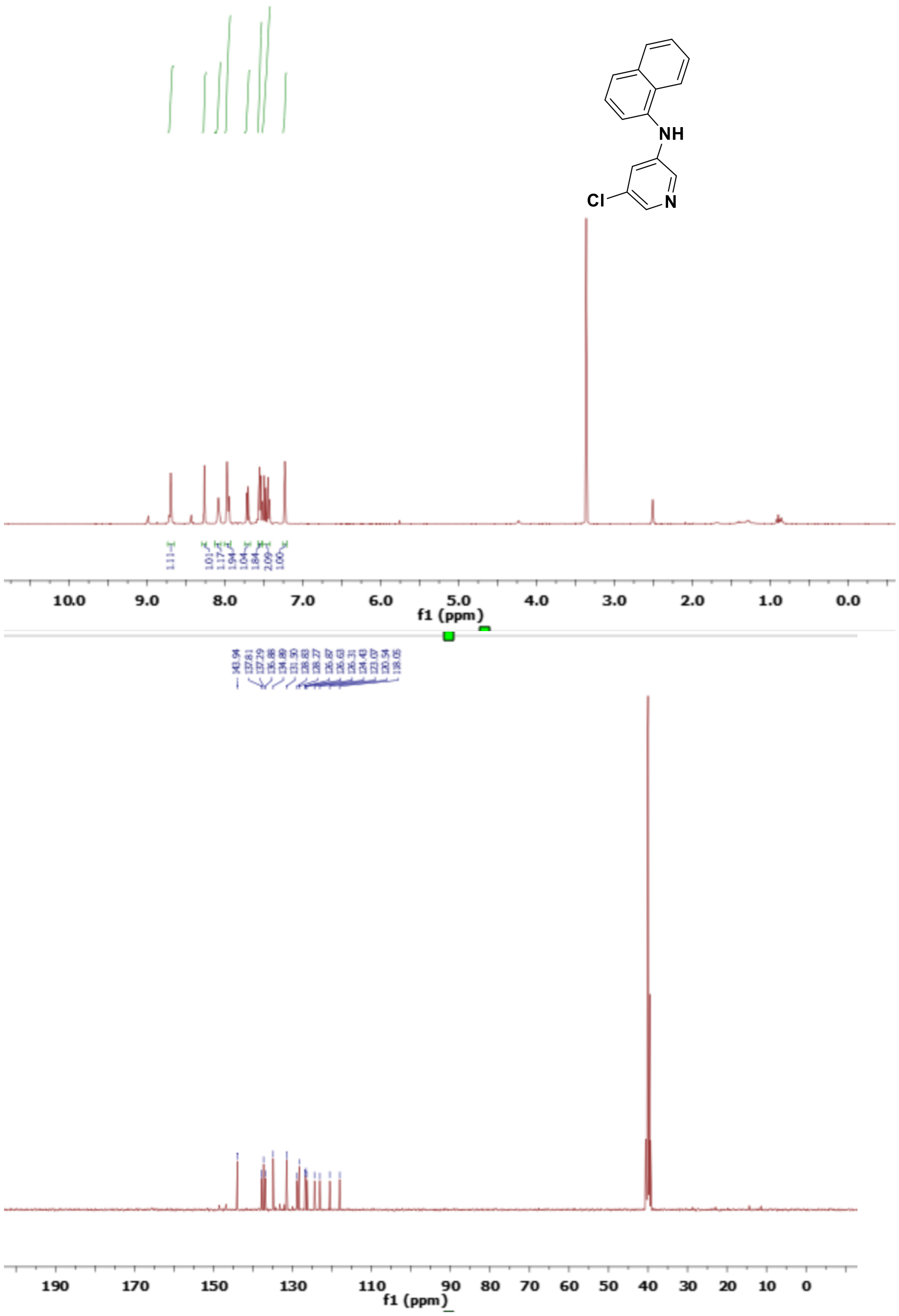

Synthesis Fig. S2. ^{1}H NMR and ^{13}C NMR spectra of 3b in $CDCl_3$

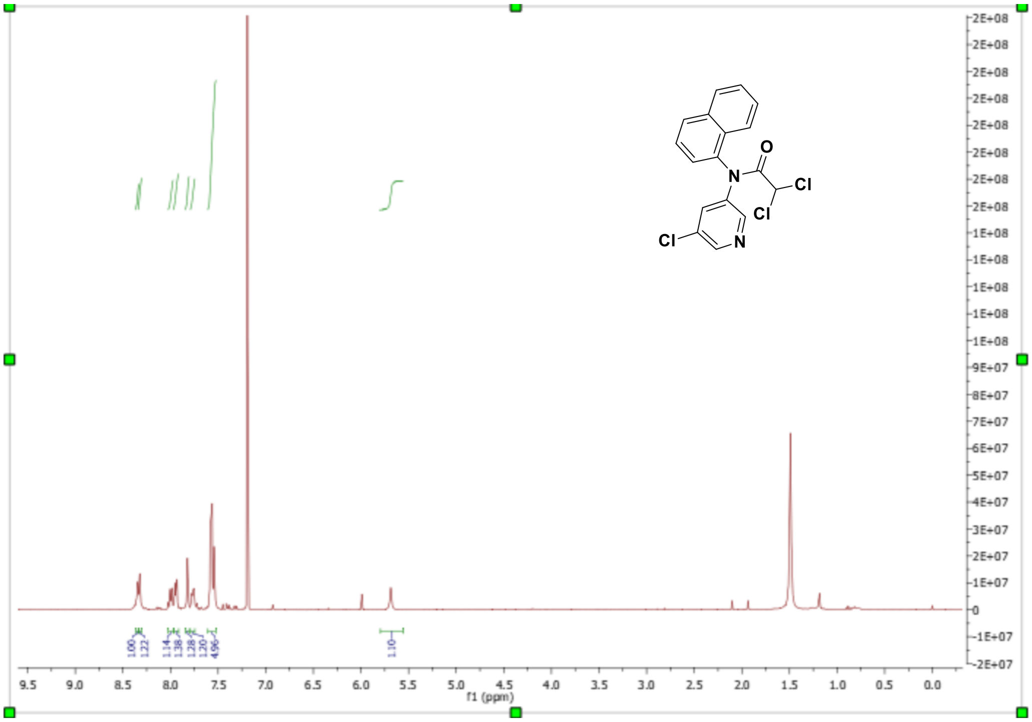

Synthesis Fig. S3. ^{1}H NMR spectra of SR-A-178 in $CDCl_3$

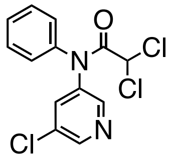

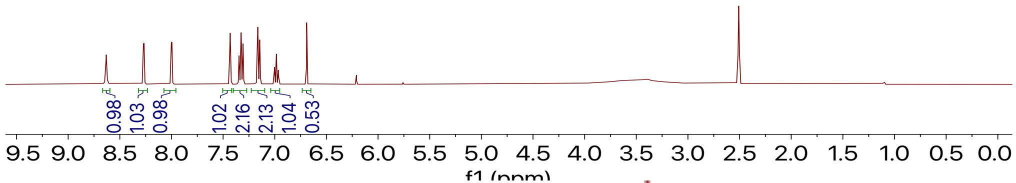

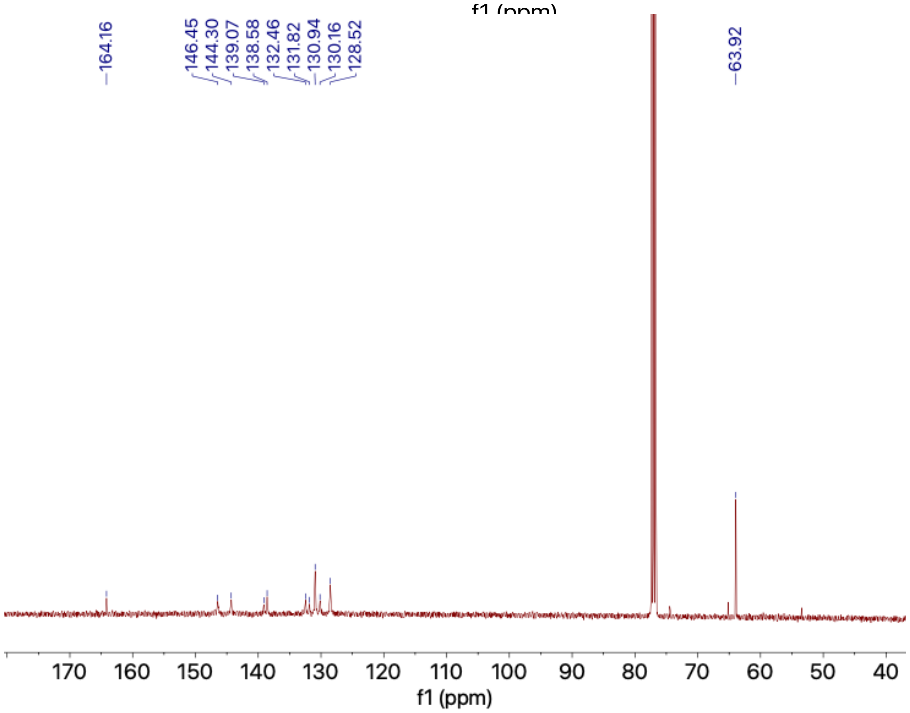

Synthesis Fig. S4. ^{1}H NMR spectra of VB-B-112 in DMSO-d_6 and ^{13}C NMR in $CDCl_3$

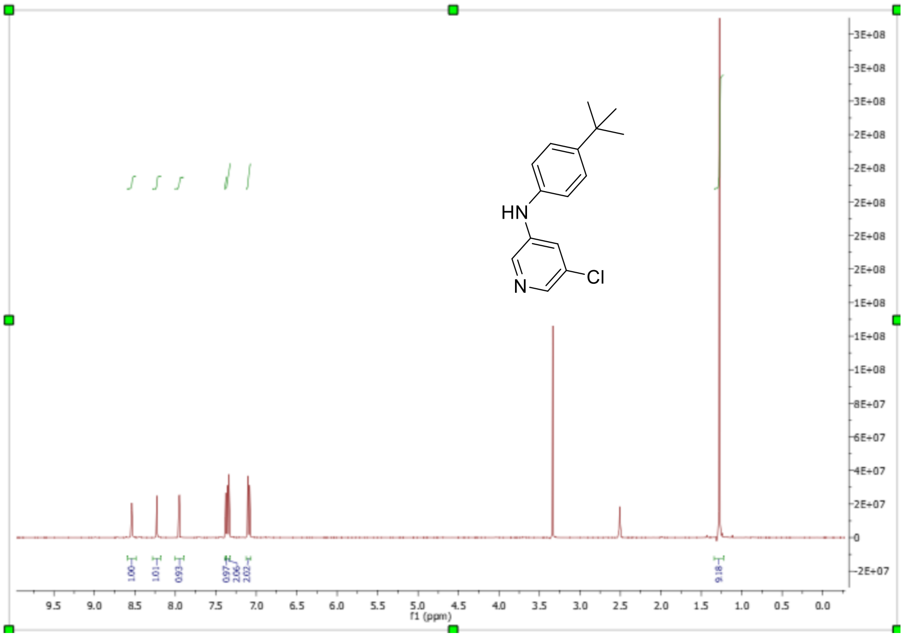

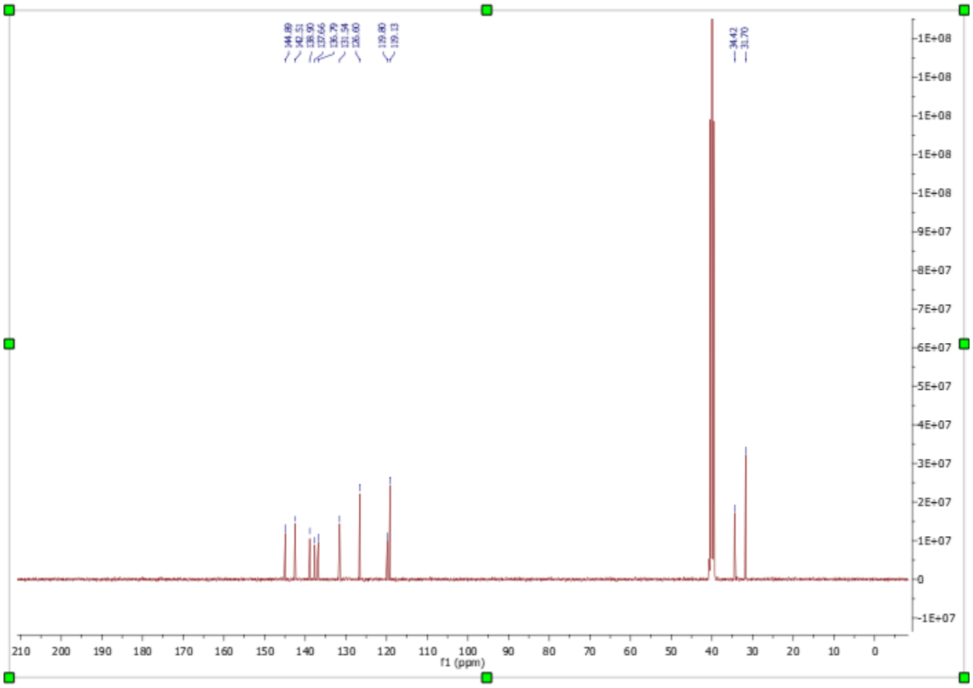

Synthesis Fig. S5. ^{1}H NMR and ^{13}C NMR spectra of 3d in DMSO-d_6

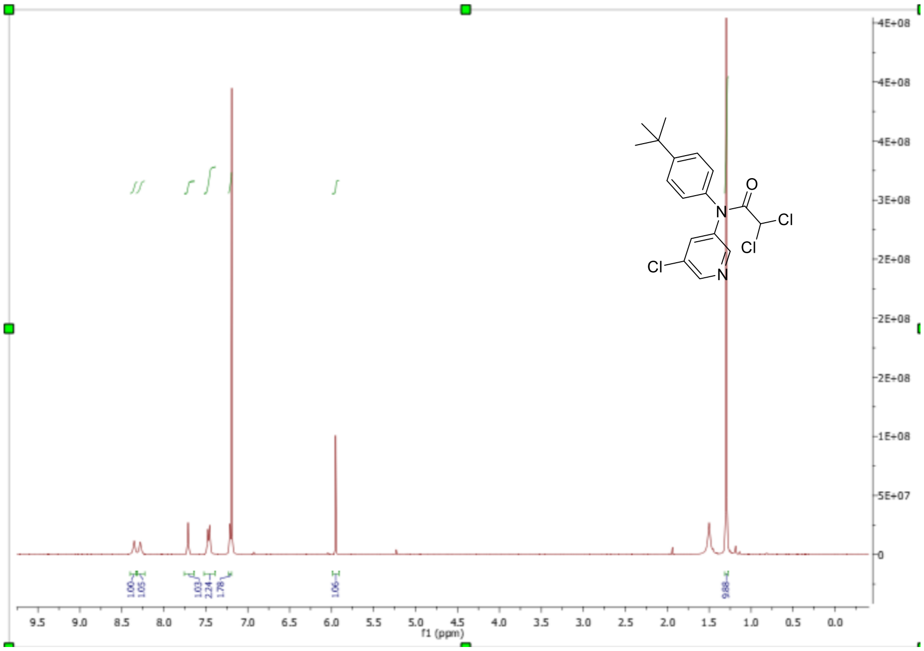

Synthesis Fig. S6. ^{1}H NMR spectra of SR-A-171 in DMSO-d_6

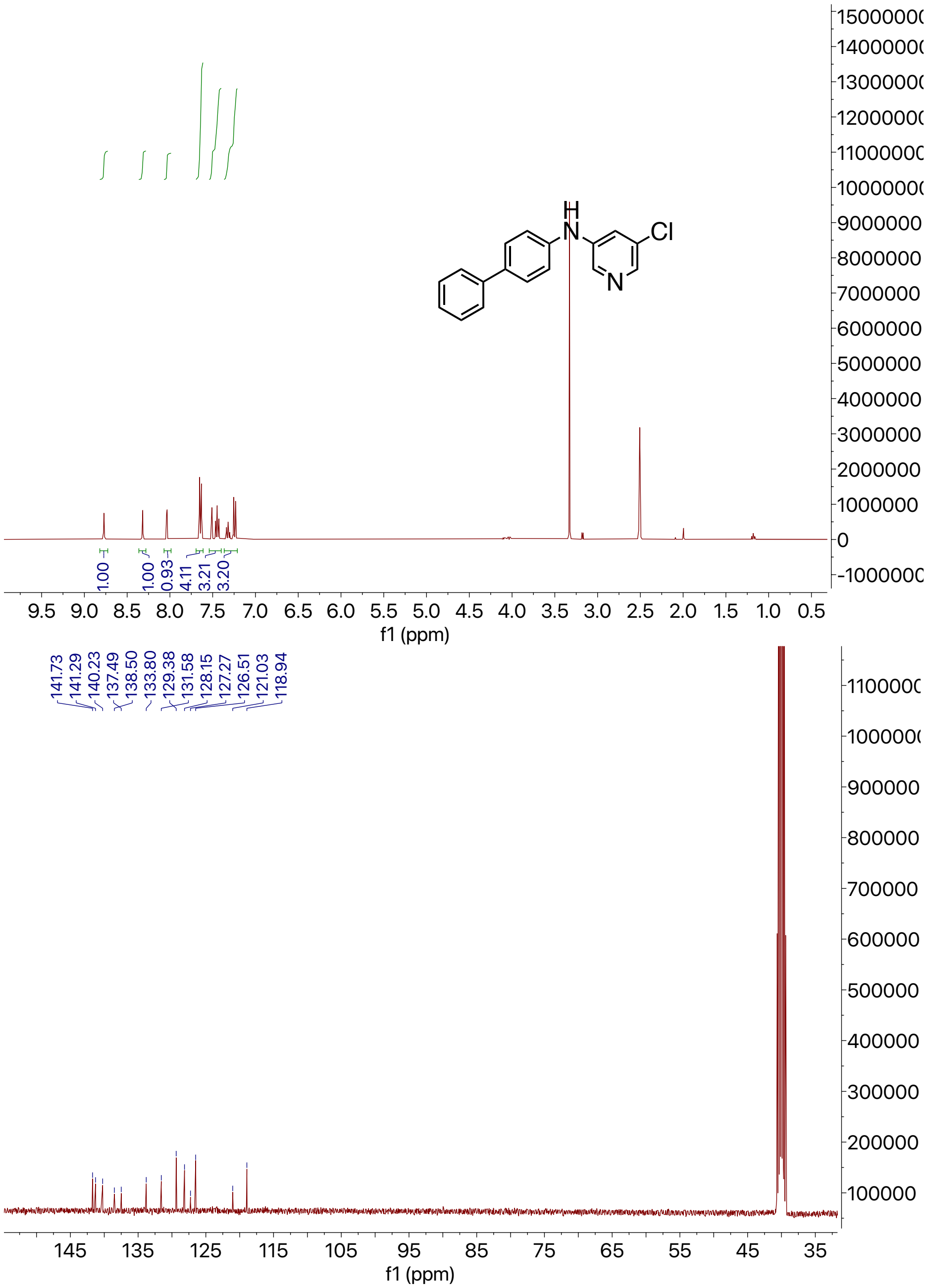

Synthesis Fig. S7. ^{1}H NMR and ^{13}C NMR spectra of 3e in DMSO-d_6

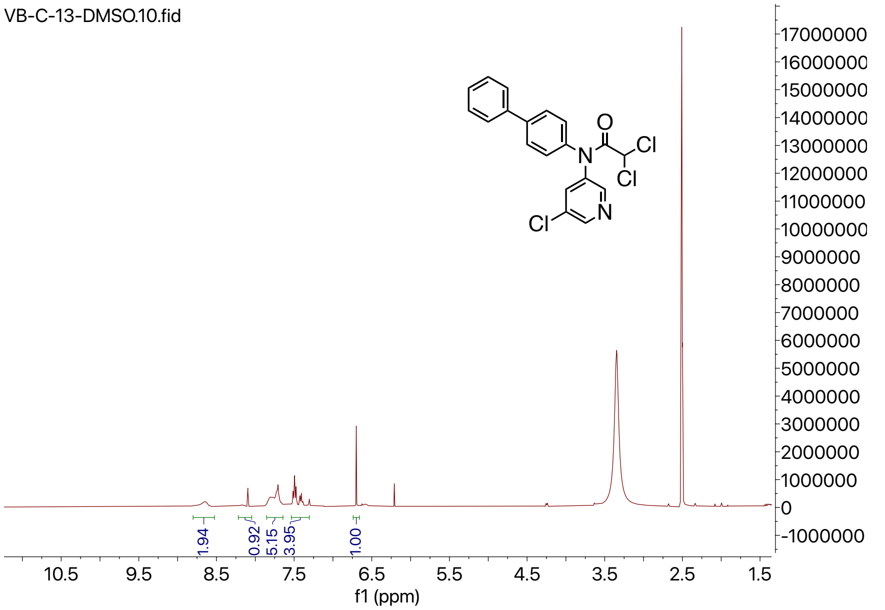

Synthesis Fig. S8. ^{1}H NMR spectra of VB-C-13 in DMSO-d_6

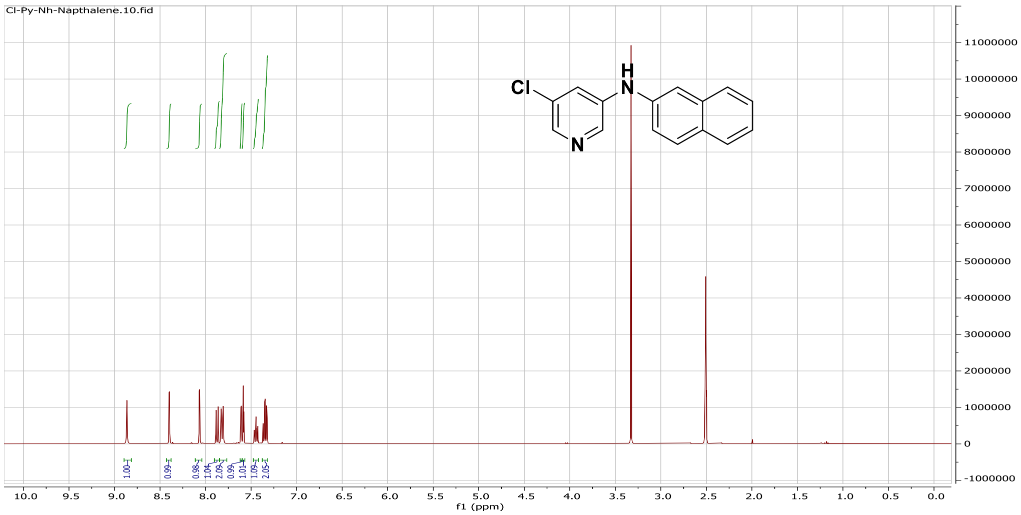

Synthesis Fig. S9. ^{1}H NMR spectra of 3f in DMSO-d_6

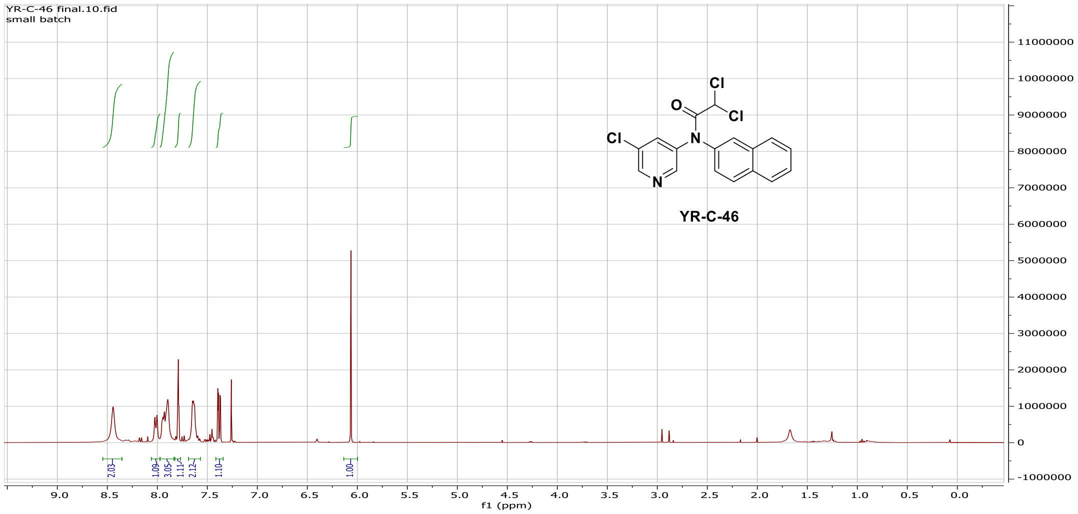

Synthesis Fig. S10. ^{1}H NMR spectra of YR-C-46 in $CDCl_3$

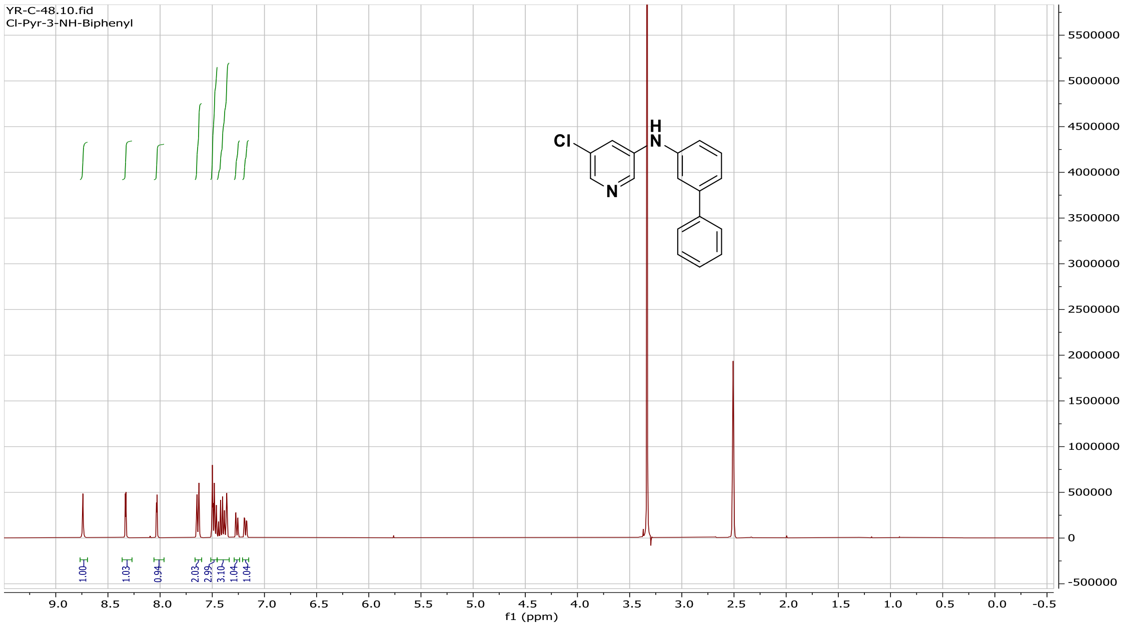

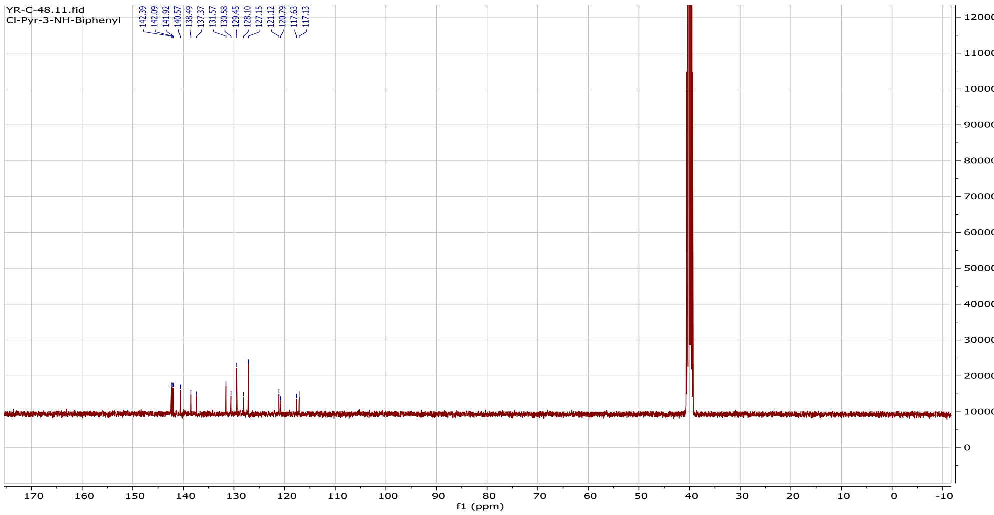

Synthesis Fig. S11. ^{1}H NMR spectra of 3g in $CDCl_3$ and ^{13}C NMR in DMSO-d_6

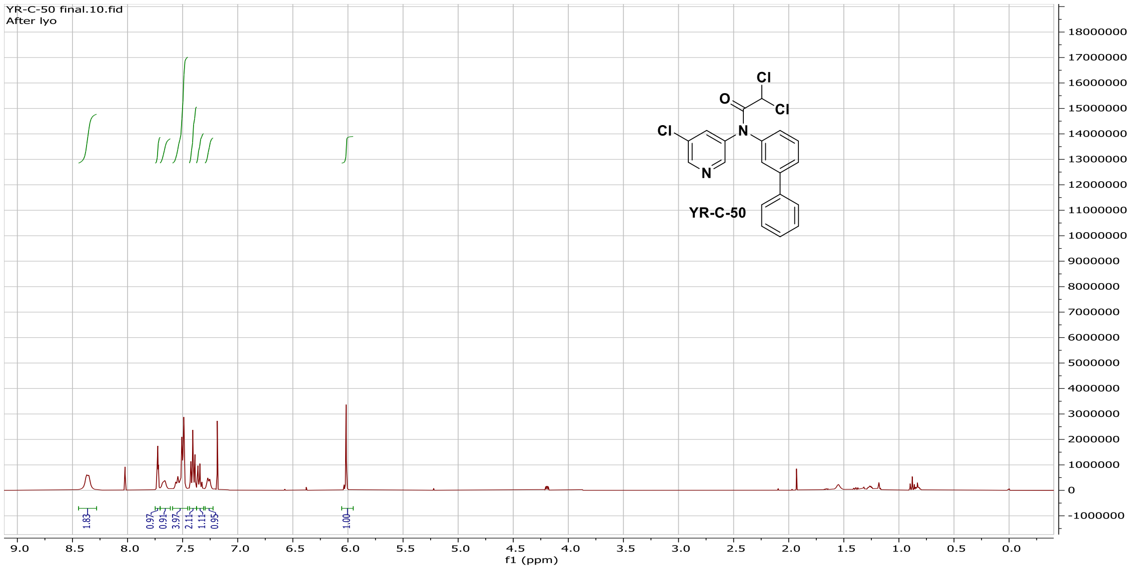

Synthesis Fig. S12. ^{1}H NMR spectra of YR-C-50 in $CDCl_3$

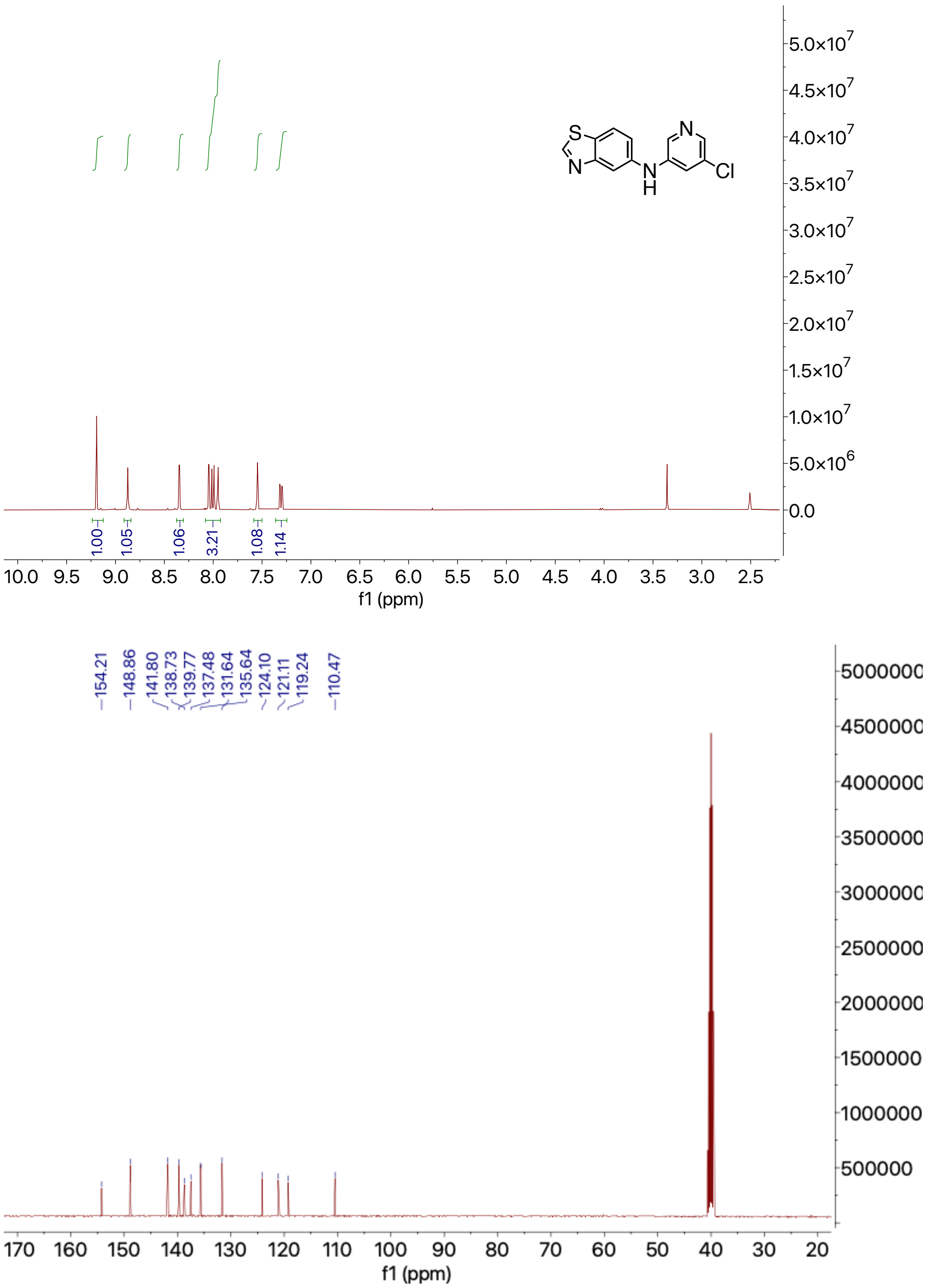

Synthesis Fig. S12. ^{1}H NMR and ^{13}C NMR spectra of 3h in DMSO-d_6

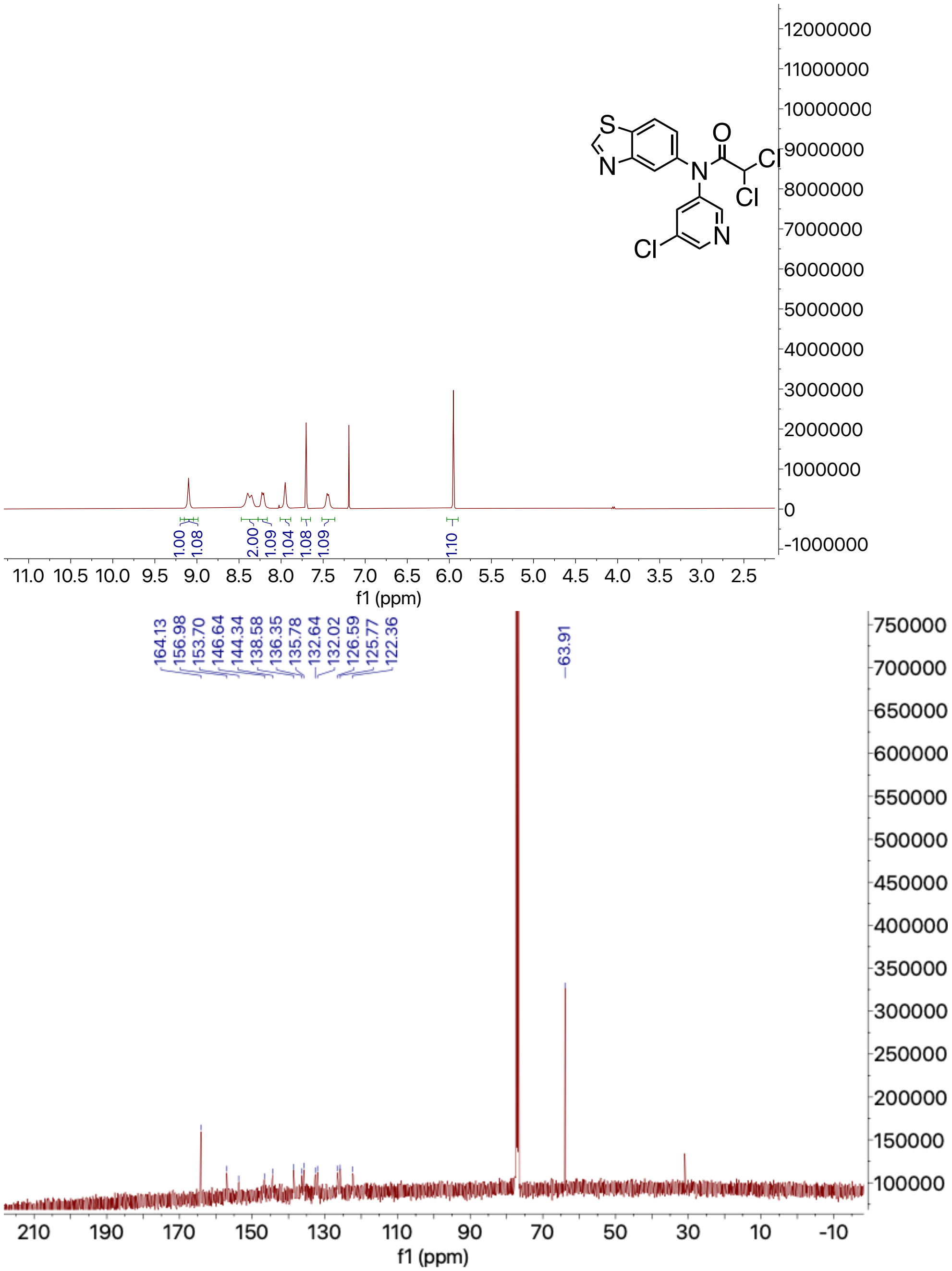

Synthesis Fig. S13. ^{1}H NMR and ^{13}C NMR spectra of VB-C-48 in $CDCl_3$

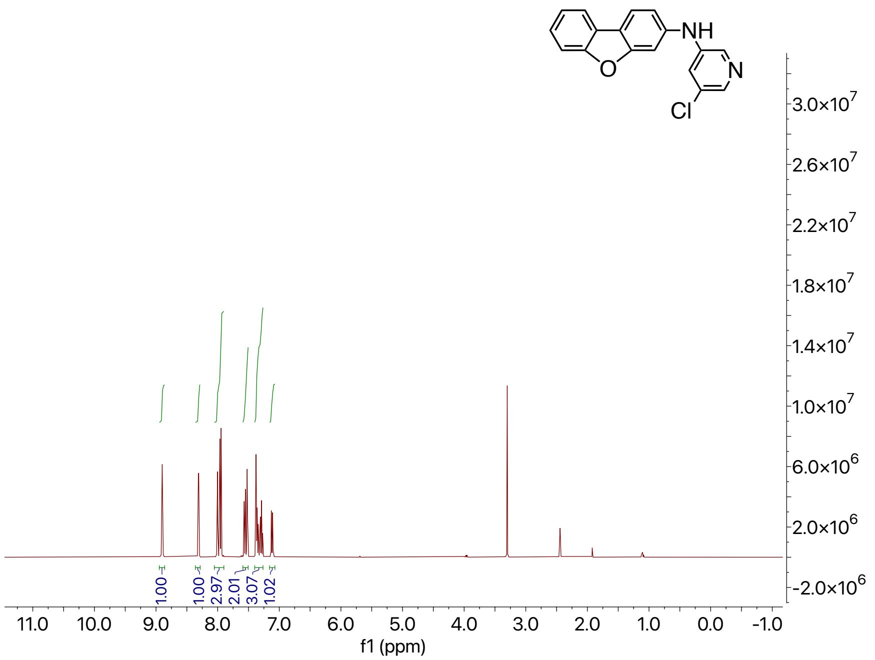

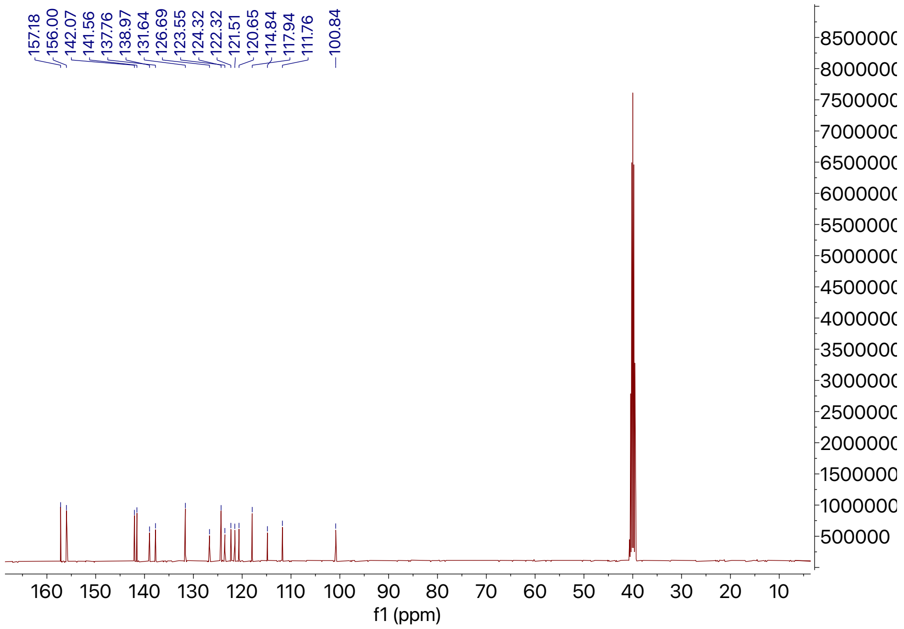

Synthesis Fig. S14. ^{1}H NMR and ^{13}C NMR spectra of 3i in DMSO-d_6

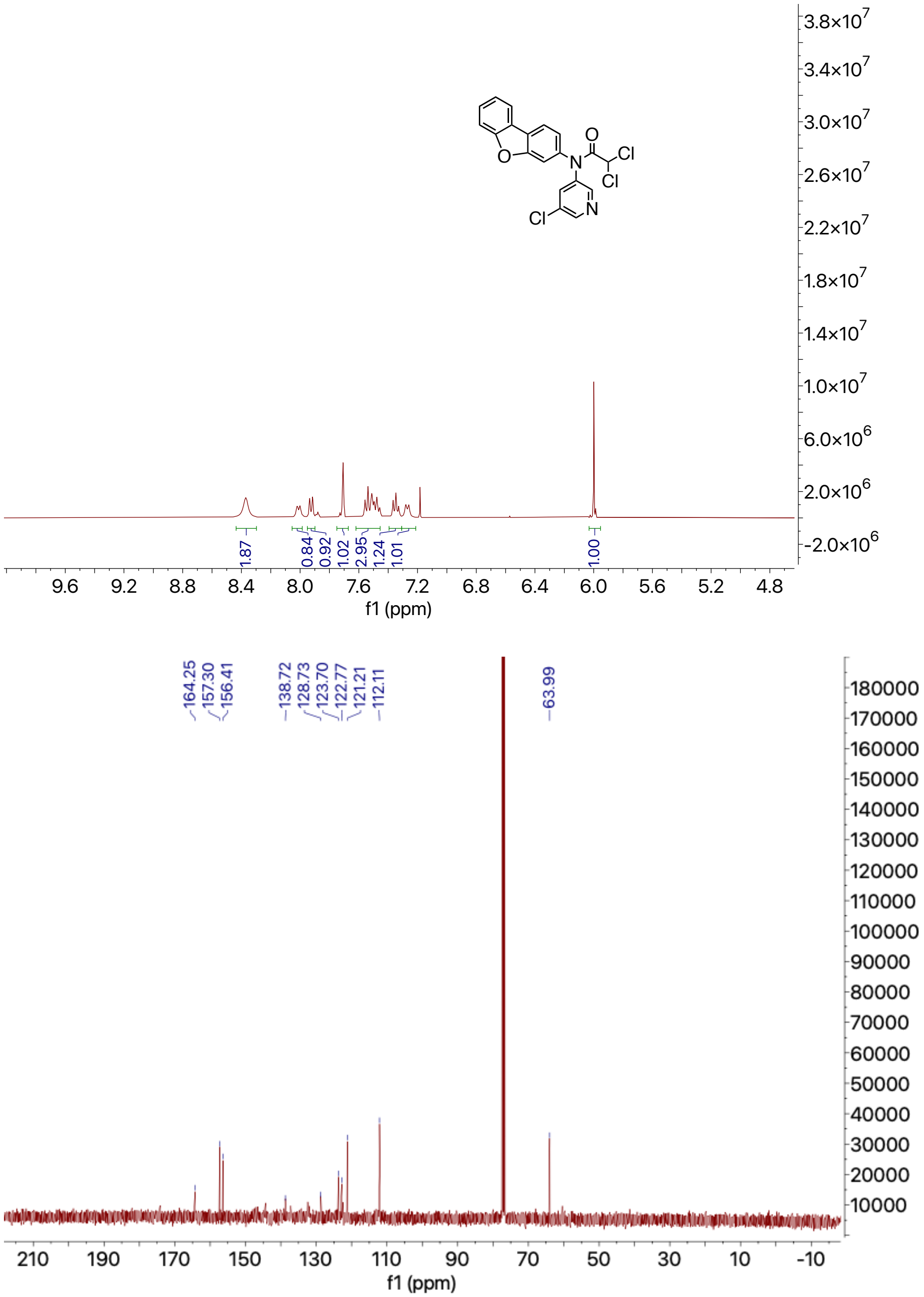

Synthesis Fig. S15. ^{1}H NMR and ^{13}C NMR spectra of VB-C-52 in $CDCl_3$

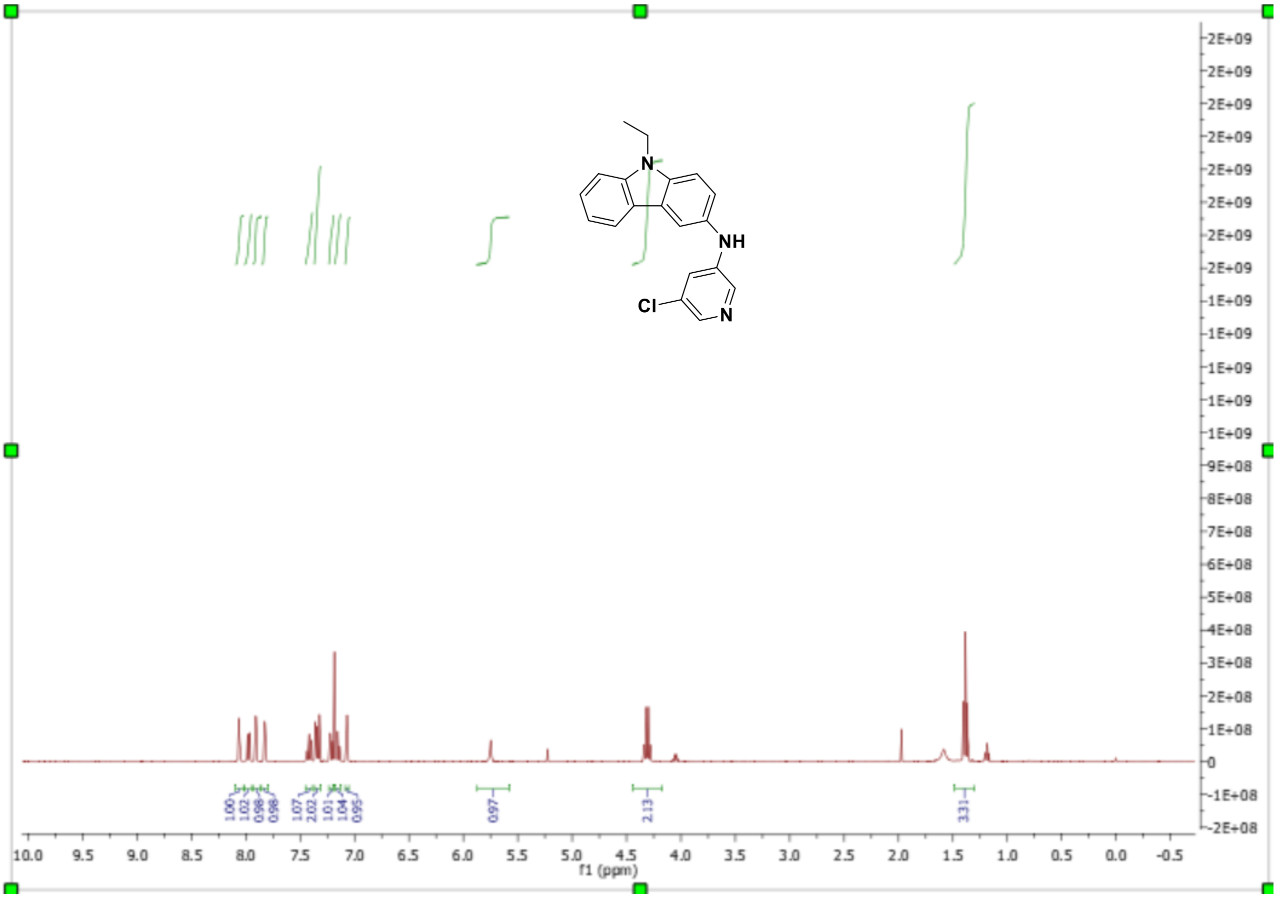

Synthesis Fig. S16. ^{1}H NMR spectra of 3j in $CDCl_3$

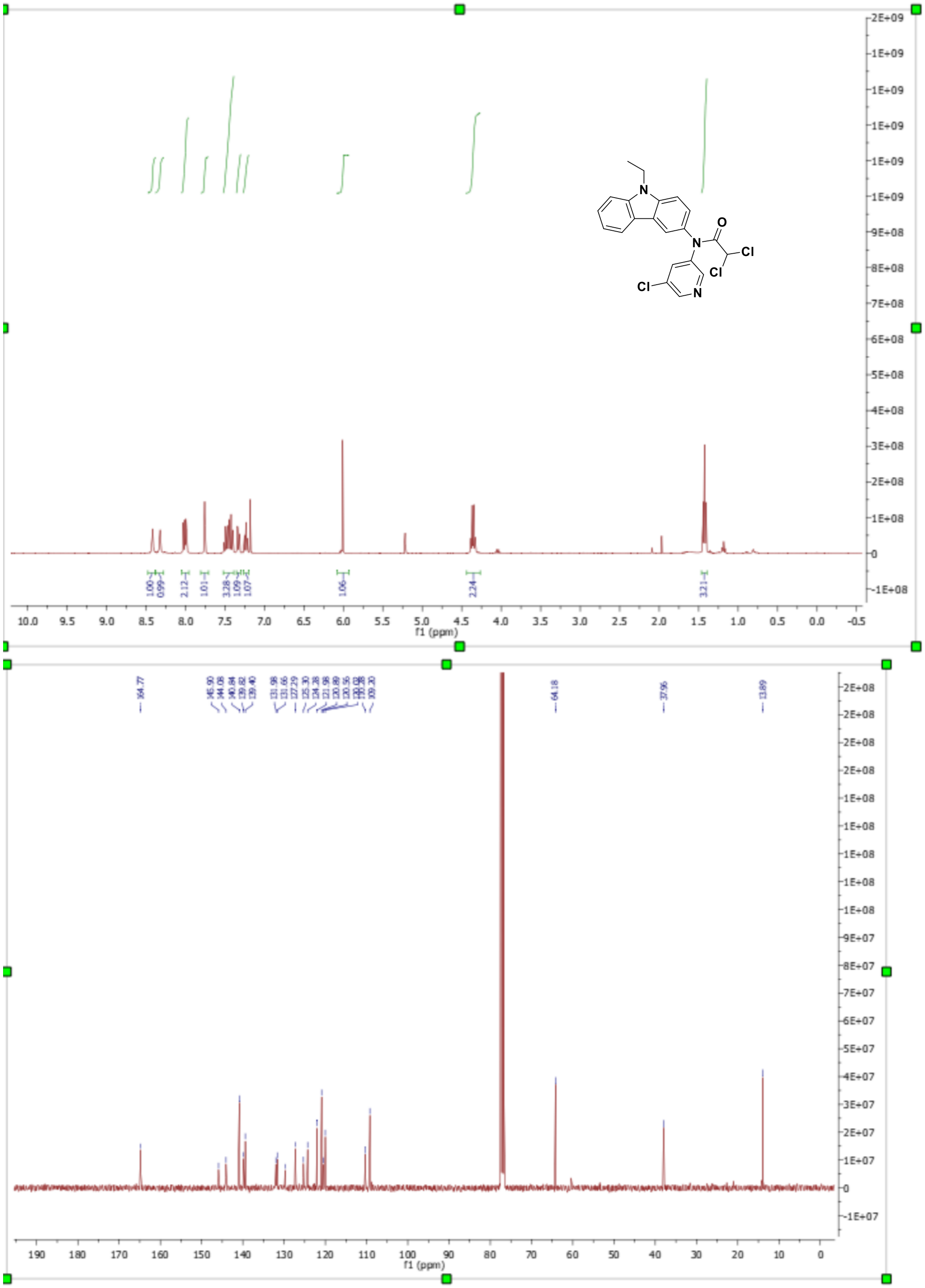

Synthesis Fig. S17. ^{1}H **NMR and** ^{13}C **NMR spectra of SR-B-33 in** $CDCl_3$

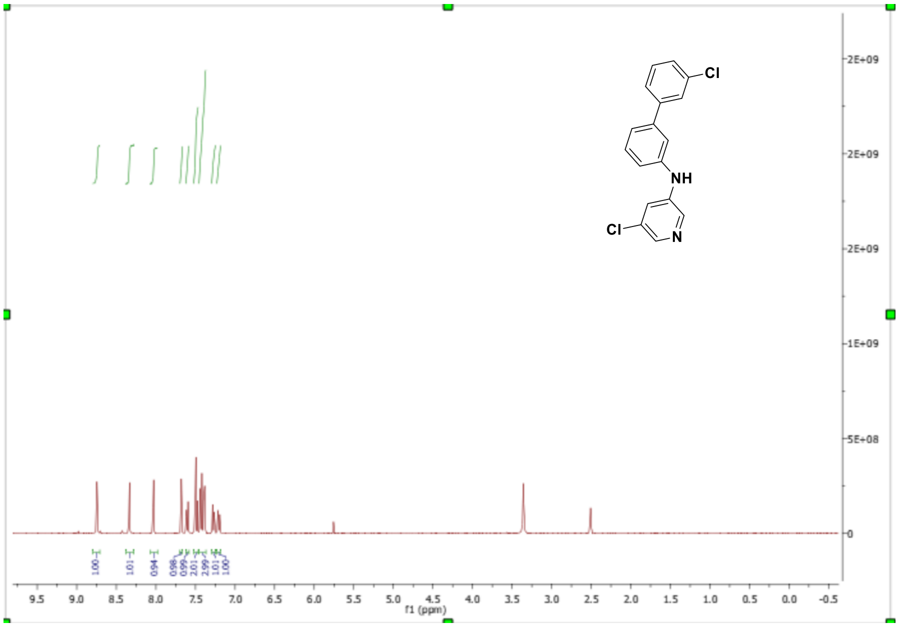

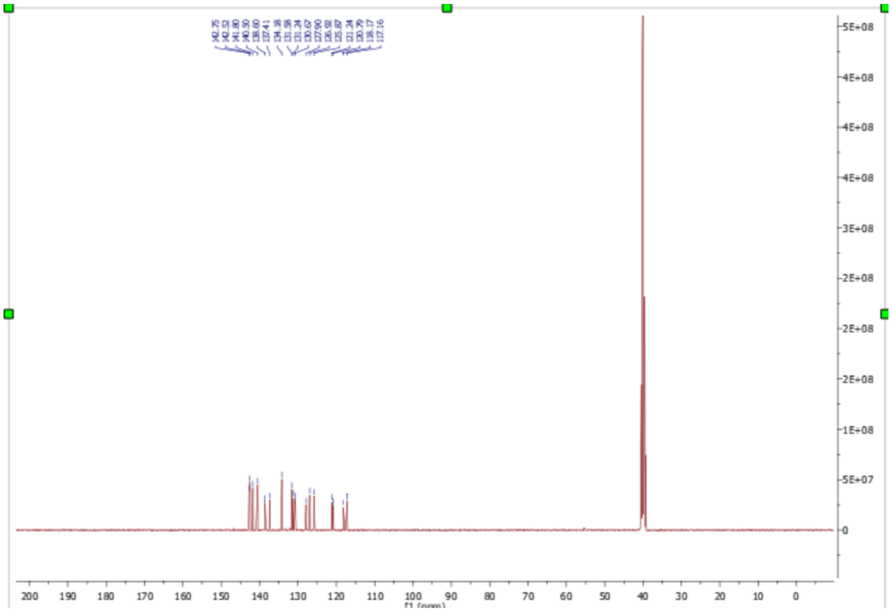

Synthesis Fig. S18. ^{1}H NMR and ^{13}C NMR spectra of 3k in DMSO-d_6

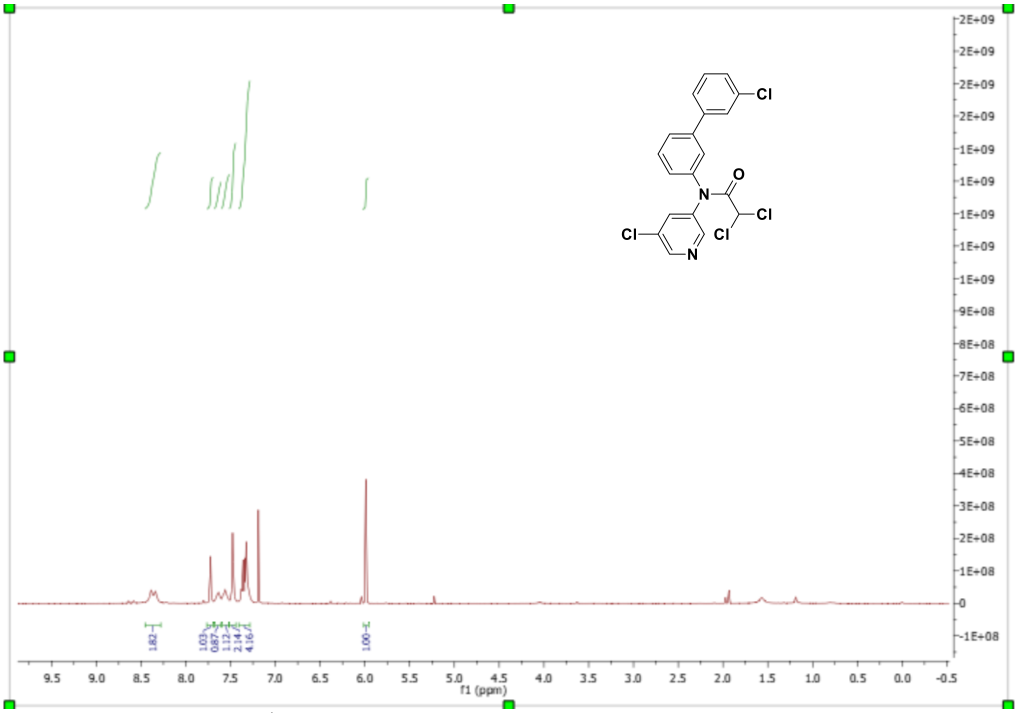

Synthesis Fig. S19. ^{1}H NMR Spectra of SR-A-174 in $CDCl_3$

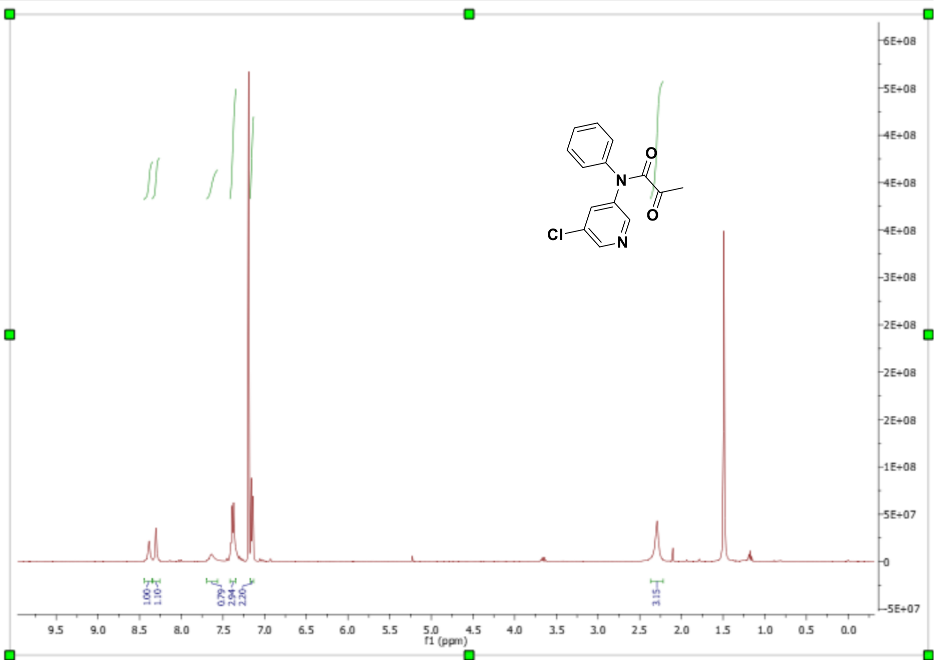

Synthesis Fig. S20. ^{1}H NMR Spectra of SR-A-188 in $CDCl_3$

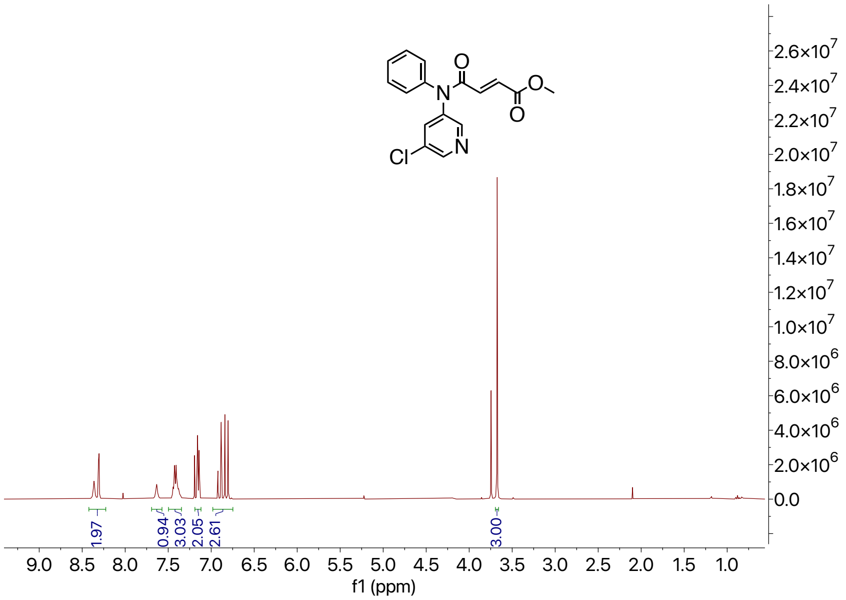

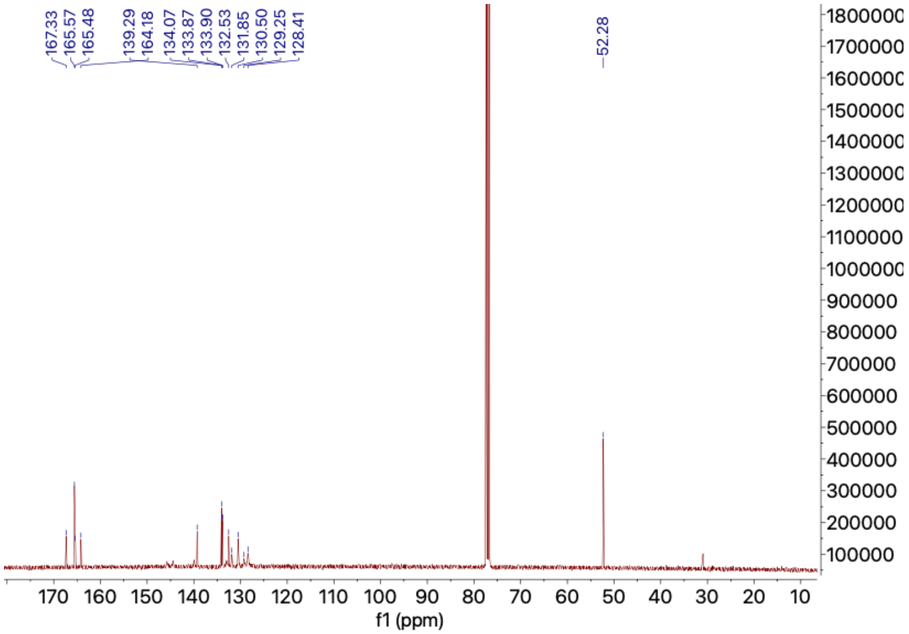

Synthesis Fig. S21. ^{1}H NMR and ^{13}C NMR Spectra of VB-C-20 in $CDCl_3$

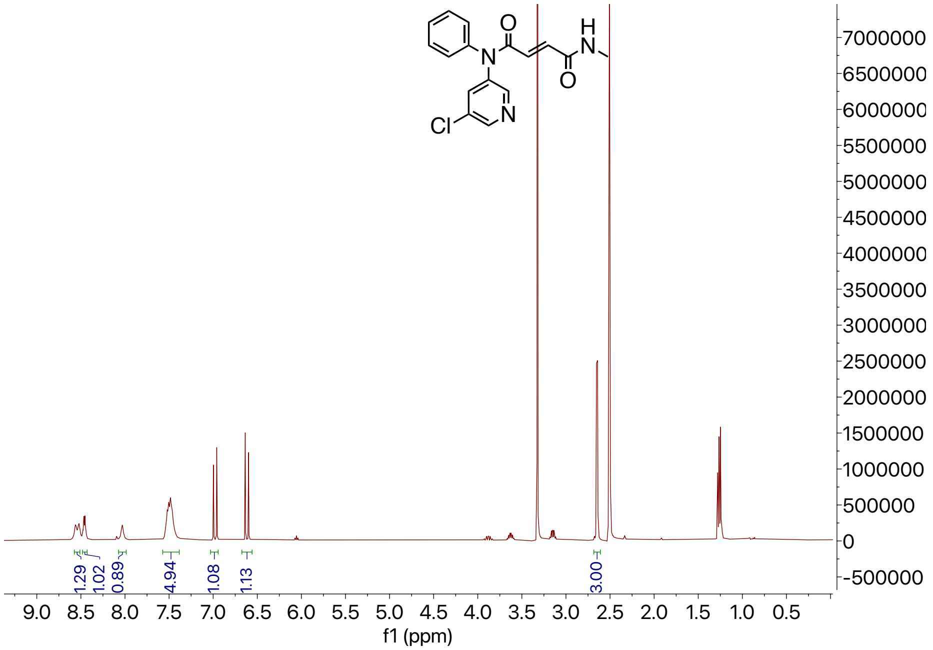

Synthesis Fig. S22. ^{1}H NMR Spectra of VB-C-25 in DMSO-*d*$_6$

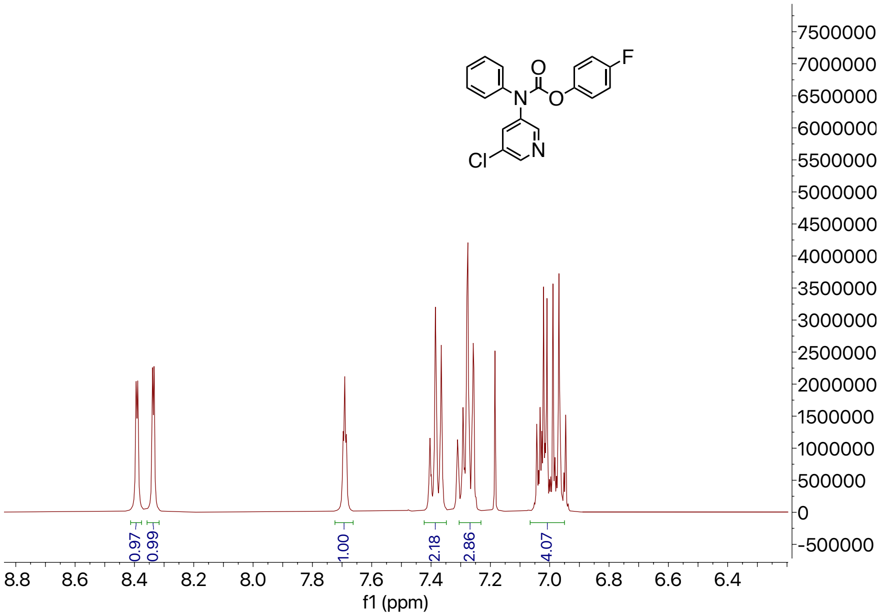

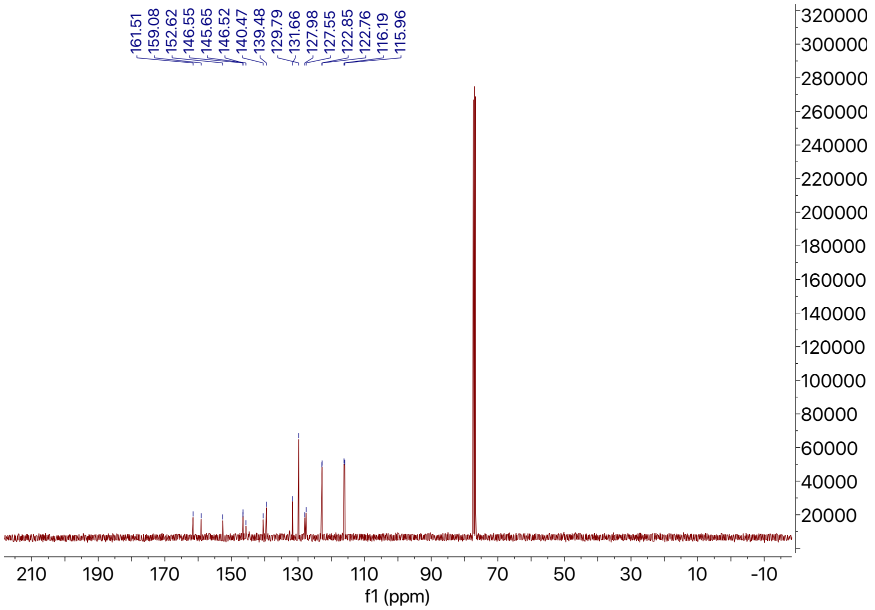

Synthesis Fig. S23. ^{1}H NMR and ^{13}C NMR Spectra of VB-C-30 in $CDCl_3$

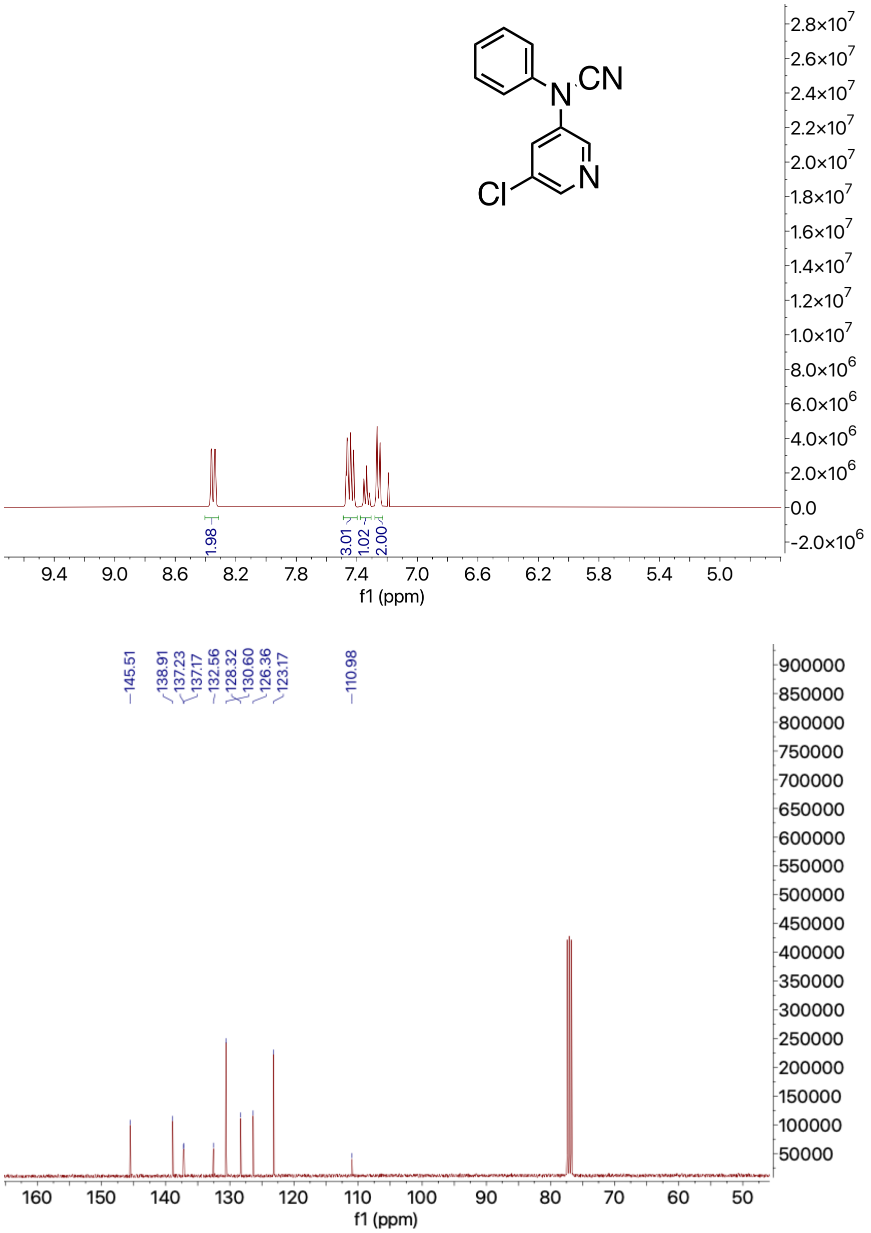

Synthesis Fig. S24. ^{1}H NMR and ^{13}C NMR Spectra of VB-C-62 in $CDCl_3$

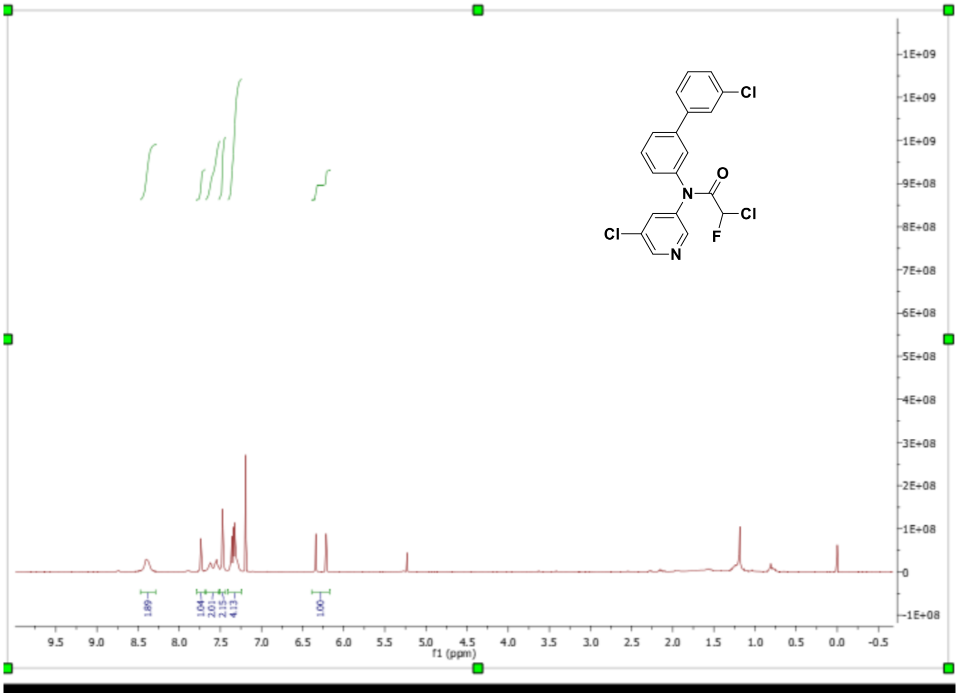

Synthesis Fig. S25. ^{1}H NMR Spectra of SR-B-7 in $CDCl_3$

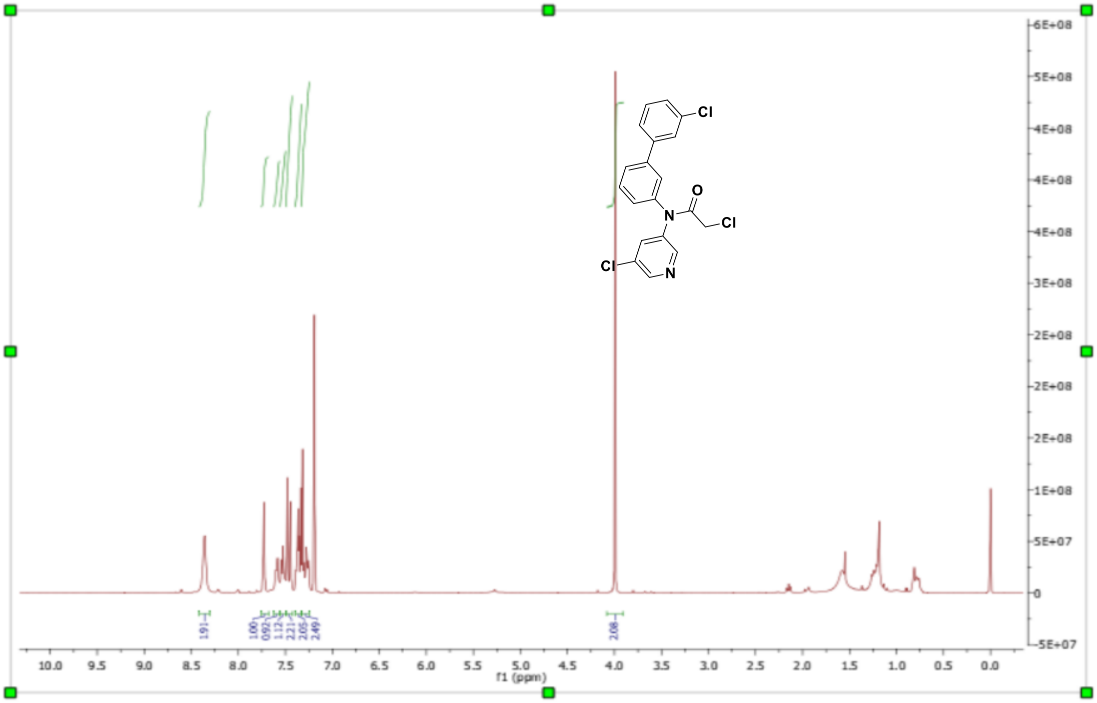

Synthesis Fig. S26. ^{1}H NMR Spectra of SR-B-17 in $CDCl_3$

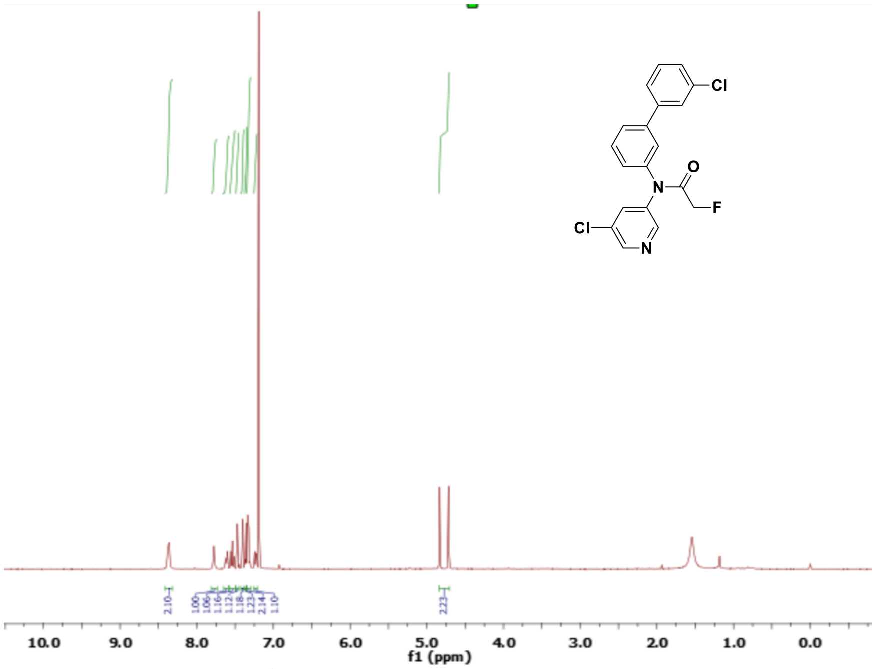

Synthesis Fig. S27. ^{1}H NMR Spectra of SR-B-84 in $CDCl_3$

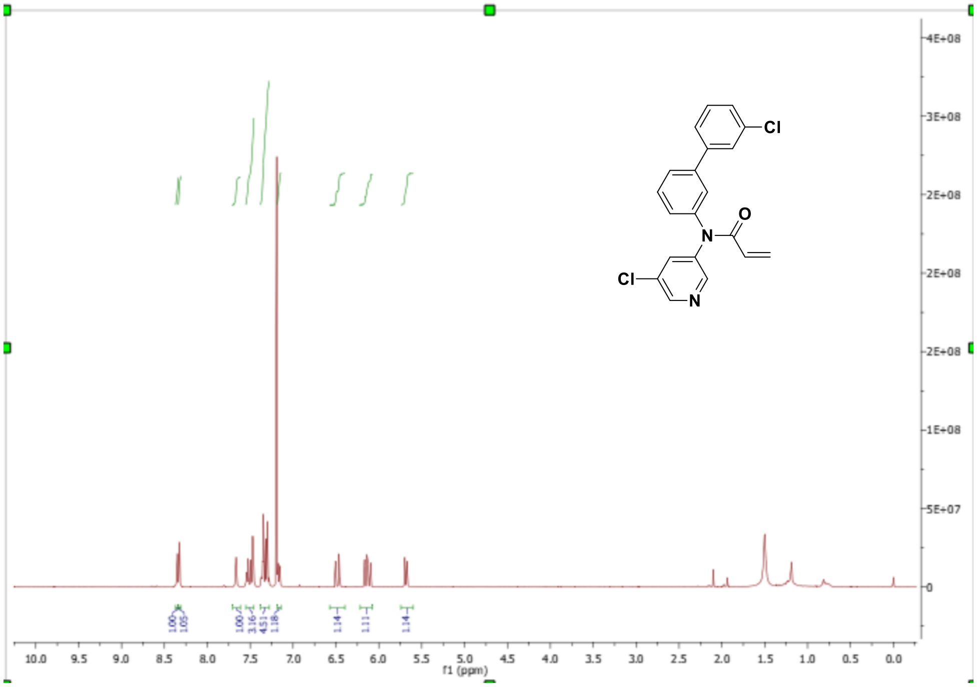

Synthesis Fig. S28. ¹H NMR Spectra of SR-B-13 in $CDCl_3$

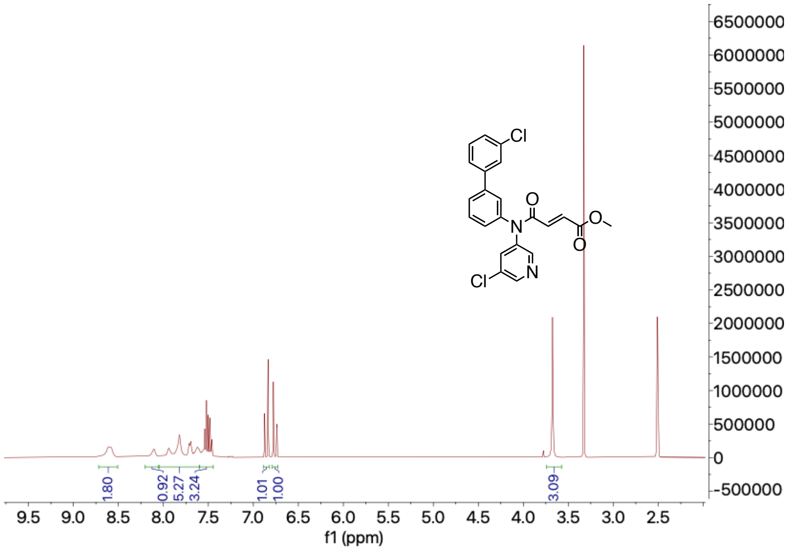

Synthesis Fig. S29. ¹H NMR Spectra of VB-C-68 in DMSO-d_6

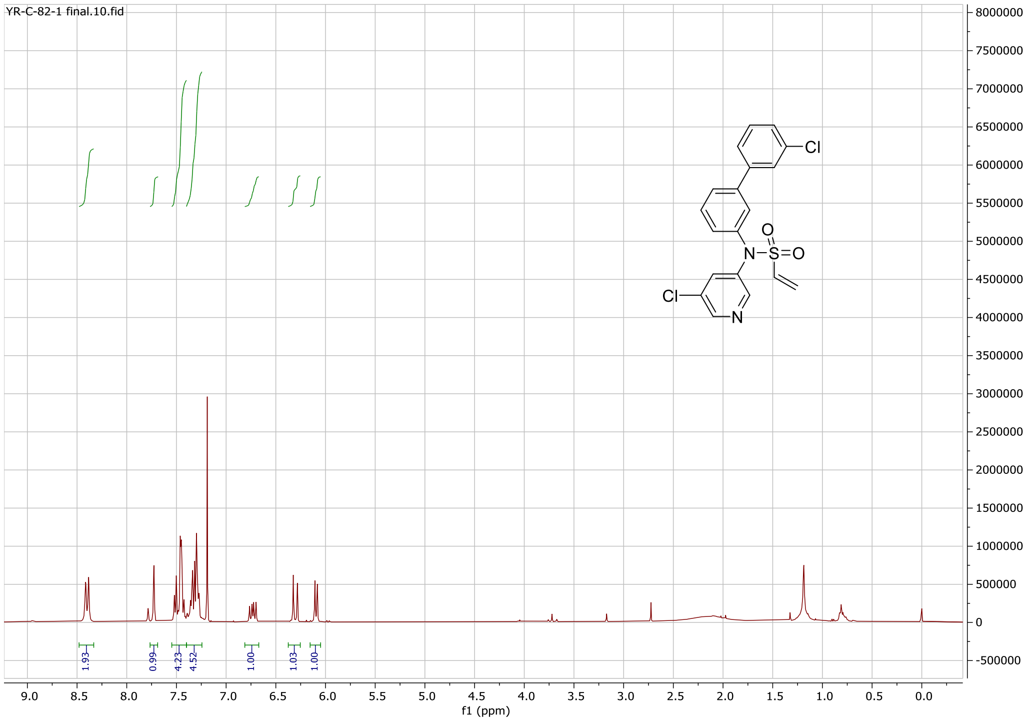

Synthesis Fig. S30. ^{1}H NMR Spectra of YR-C-82 in $CDCl_3$

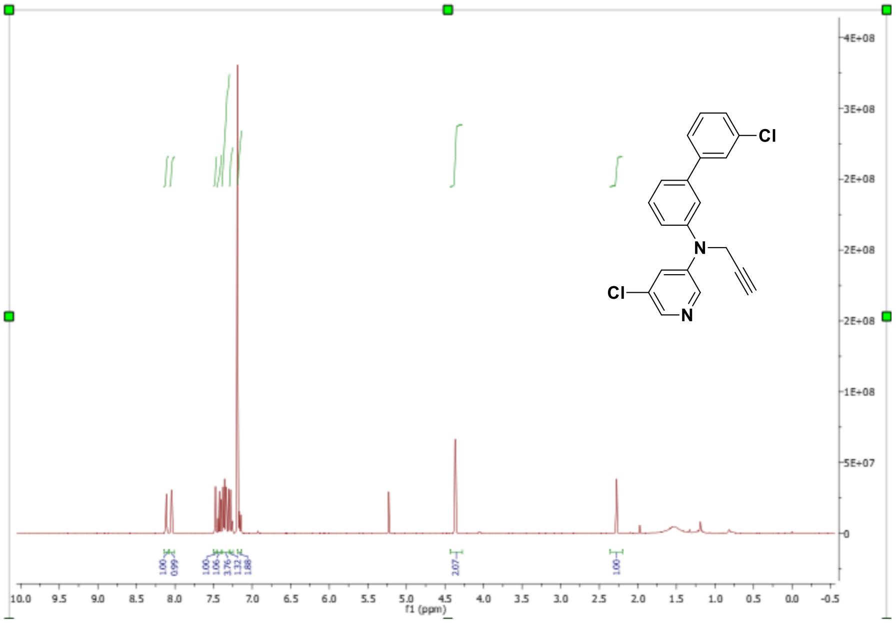

Synthesis Fig. S31. ^{1}H NMR Spectra of SR-B-15 in $CDCl_3$

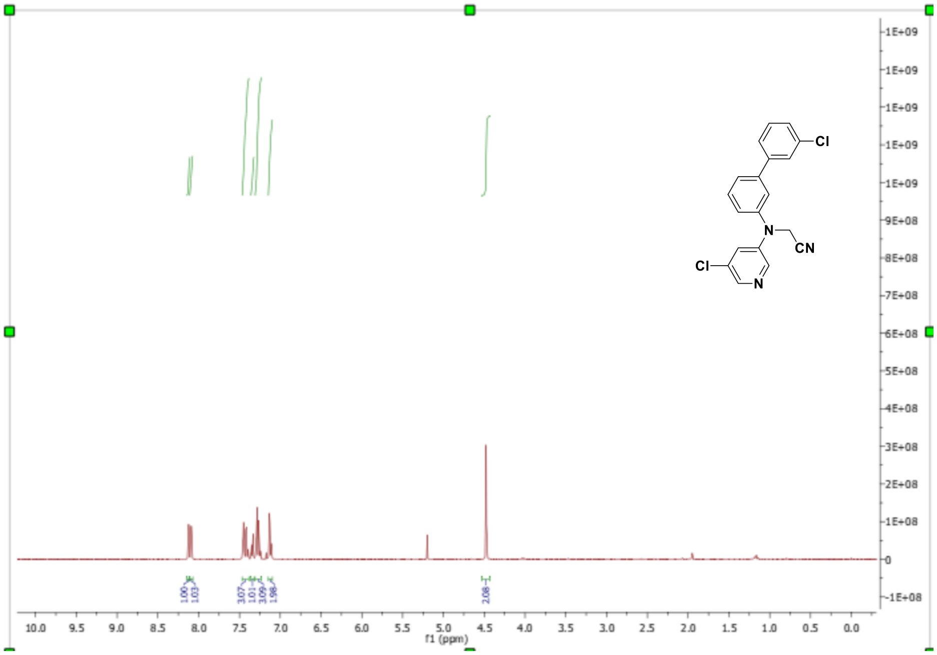

Synthesis Fig. S32. ^{1}H NMR Spectra of SR-B-22 in $CDCl_3$

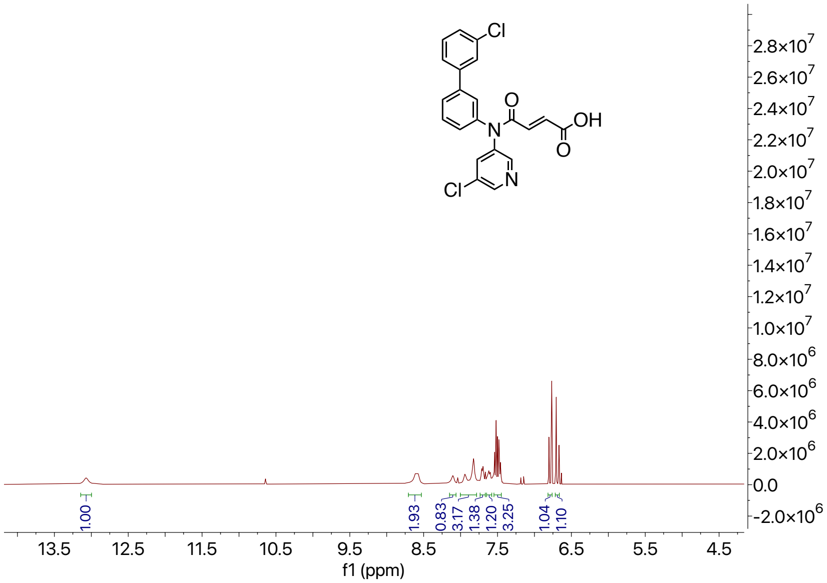

Synthesis Fig. S33. ^{1}H NMR Spectra of VB-C-69 in $CDCl_3$

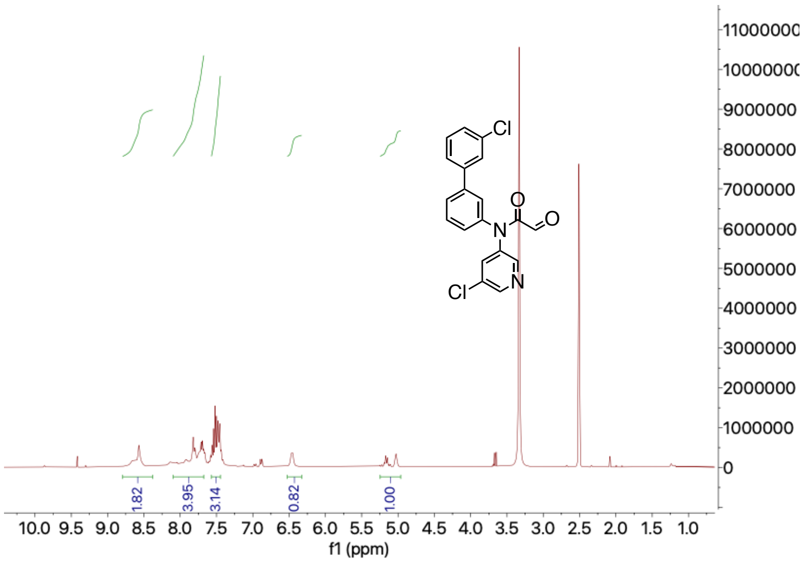

Synthesis Fig. S34. ^{1}H NMR Spectra of VB-C-70 in DMSO-d_6

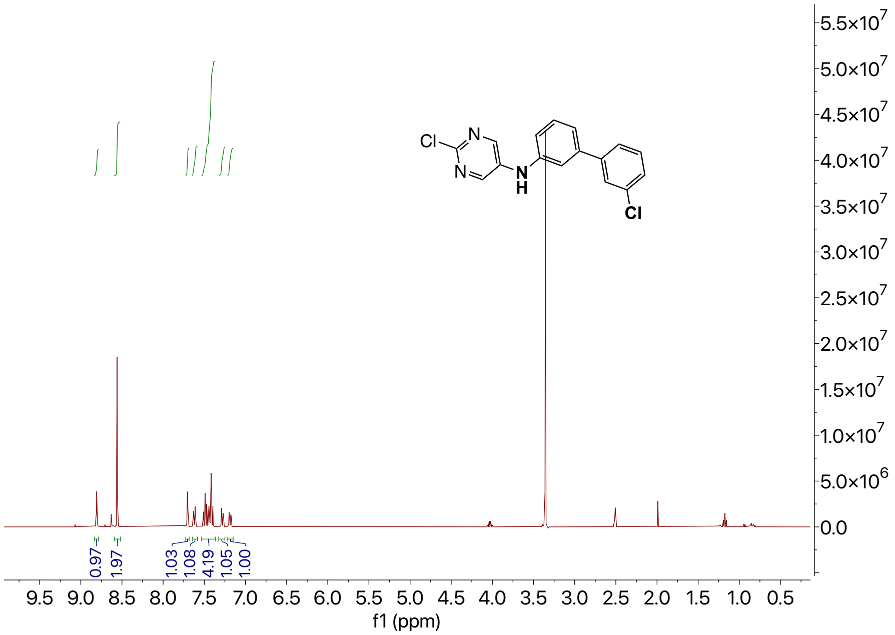

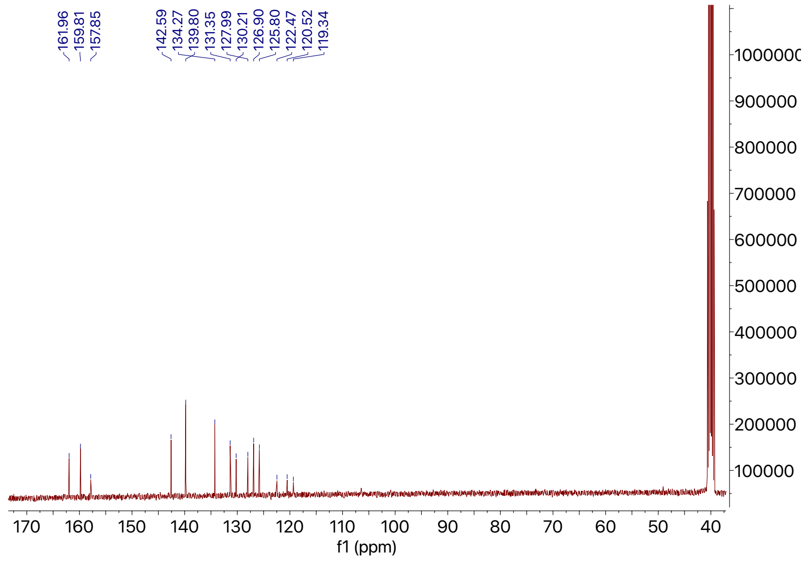

Synthesis Fig. S35. ^{1}H NMR and ^{13}C NMR Spectra of 14a in DMSO-d_6

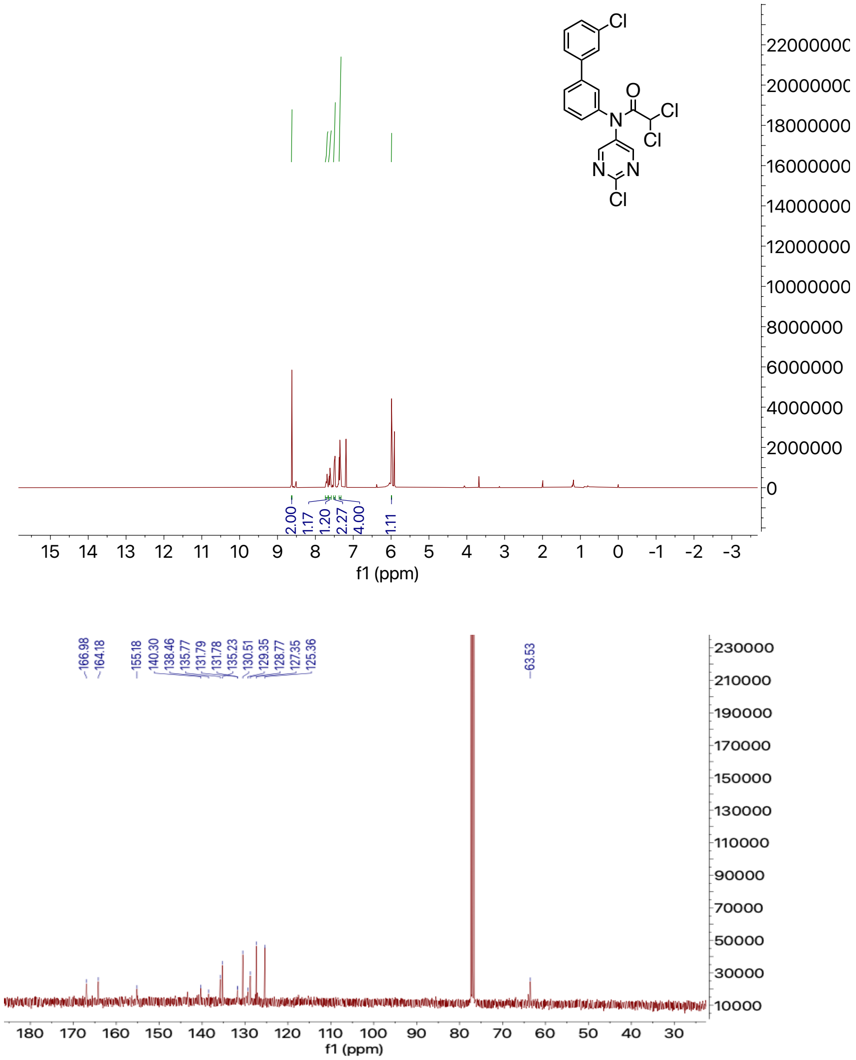

Synthesis Fig. S36. ^{1}H NMR and ^{13}C NMR Spectra of VB-C-79 in DMSO-d_6

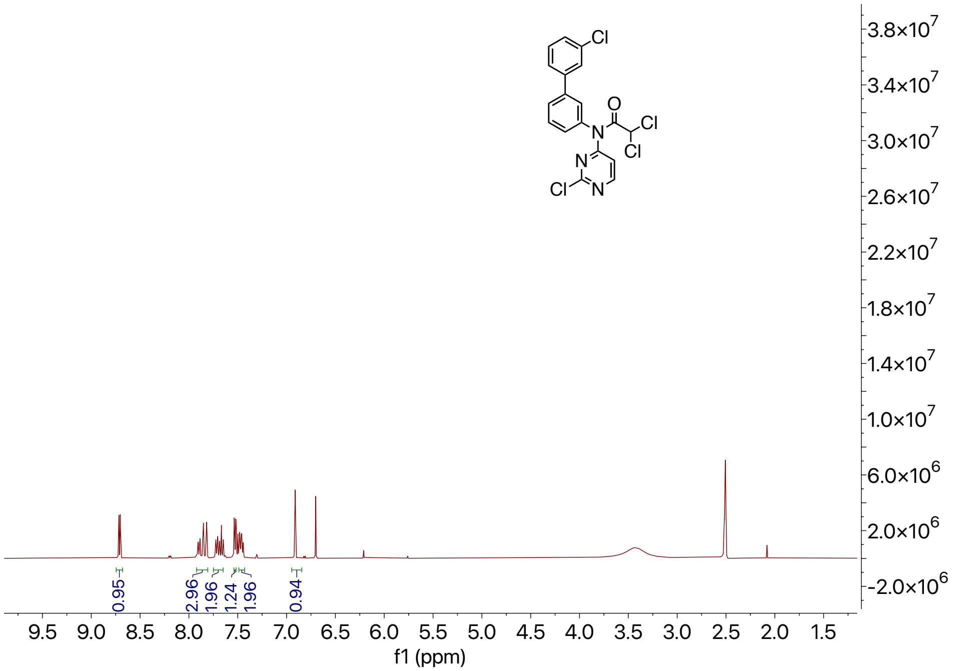

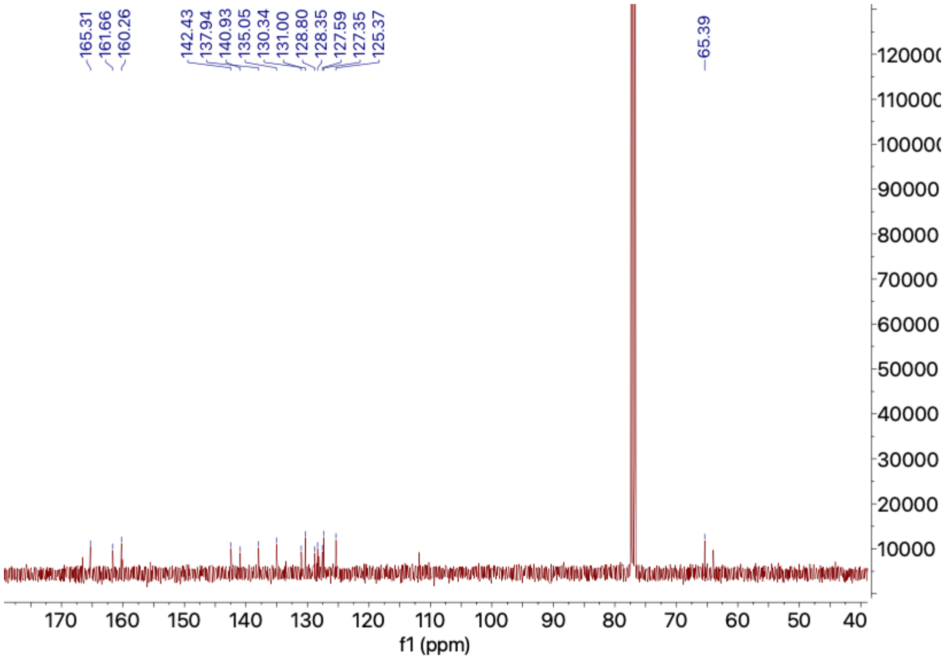

Synthesis Fig. S37. ^{1}H NMR Spectra of JK-36 in DMSO-d_6 and ^{13}C NMR in $CDCl_3$

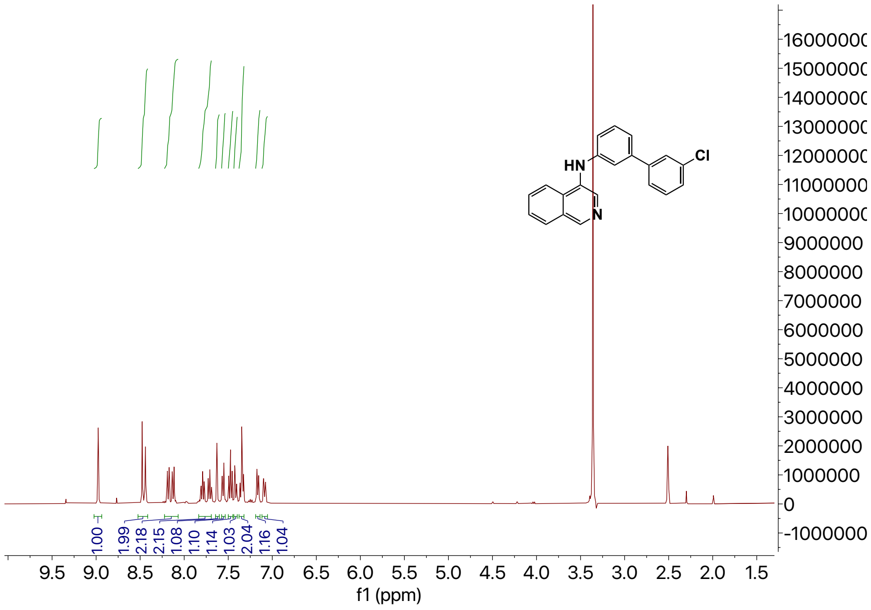

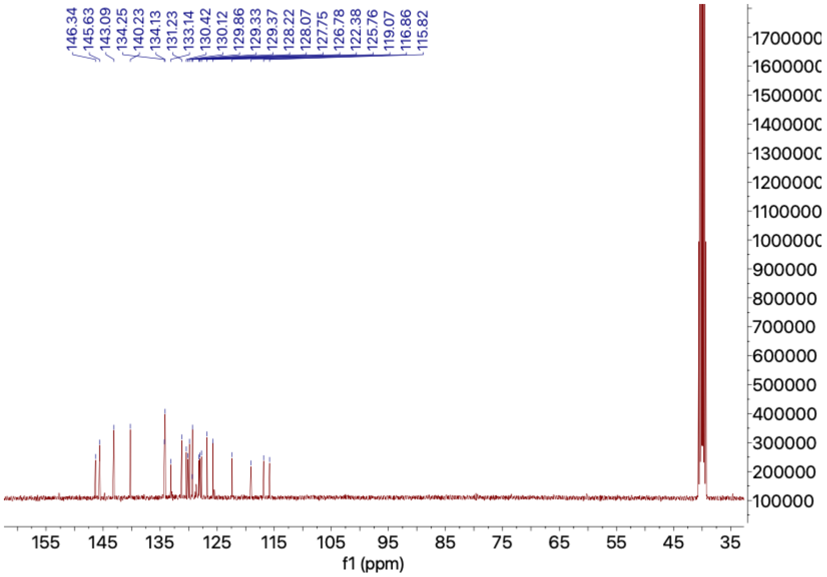

Synthesis Fig. S38. ^{1}H NMR and ^{13}C NMR Spectra of 14c in DMSO-d_6

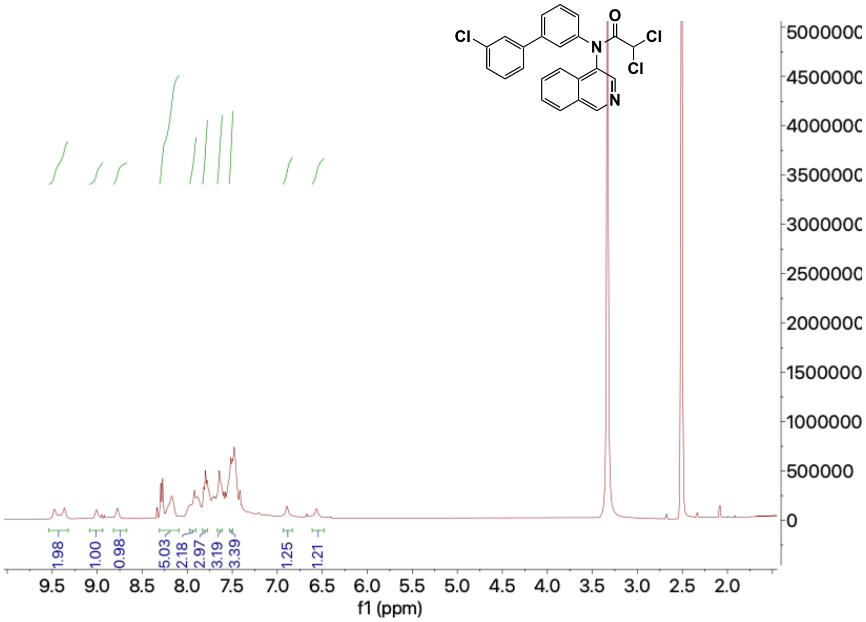

Synthesis Fig. S39. ^{1}H NMR Spectra of VB-C-200 in DMSO-d_6

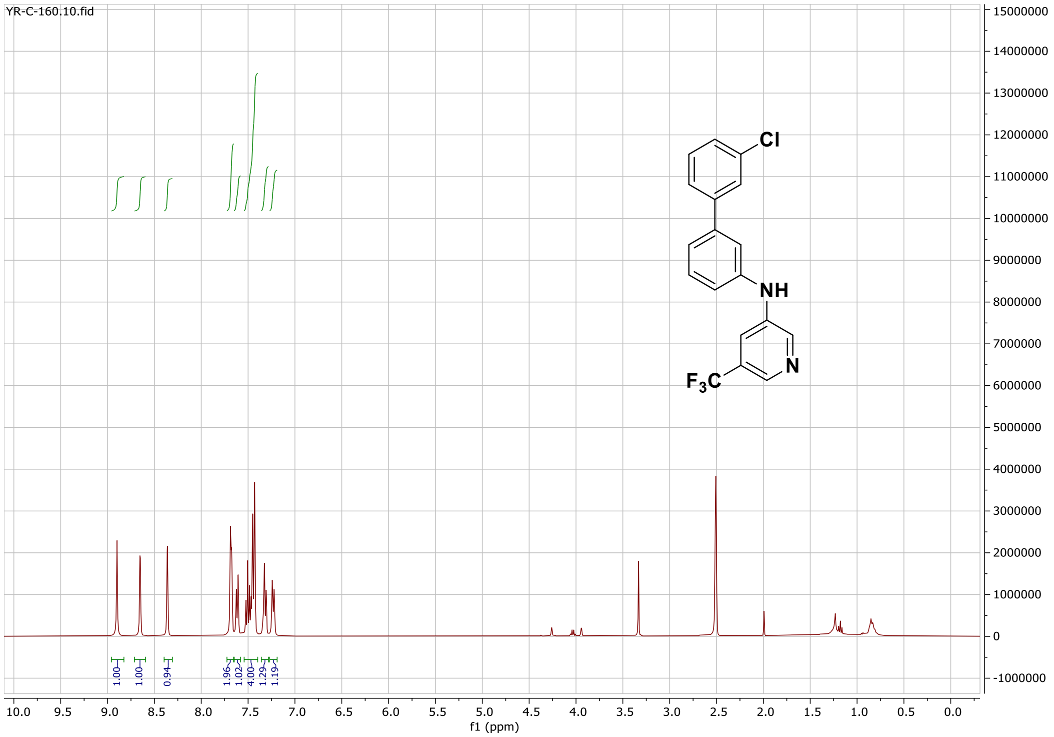

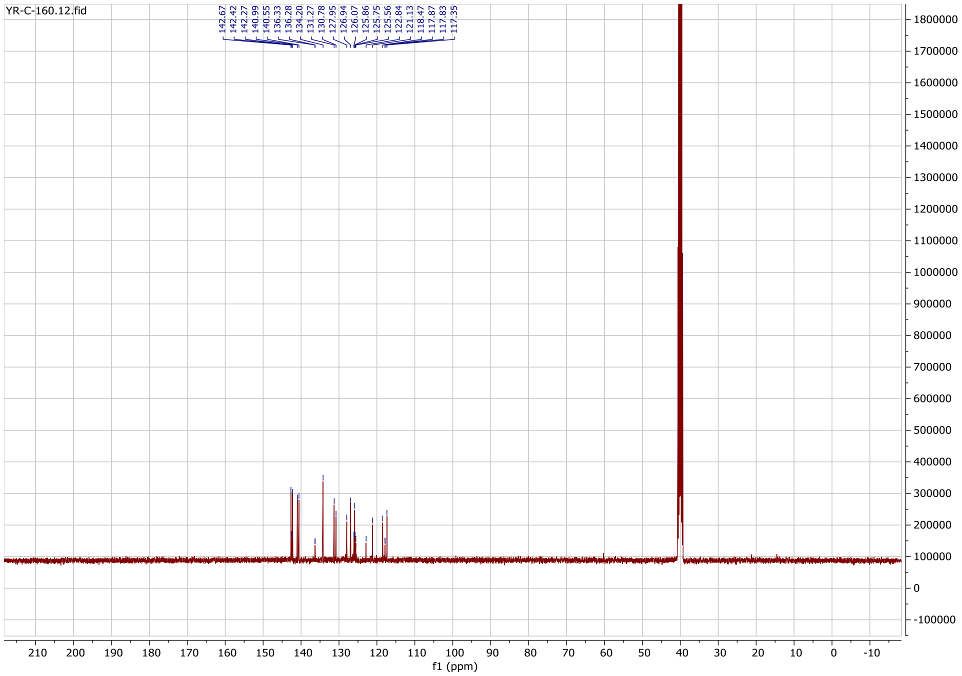

Synthesis Fig. S40. ^{1}H NMR and ^{13}C NMR Spectra of 14d in DMSO-d_6

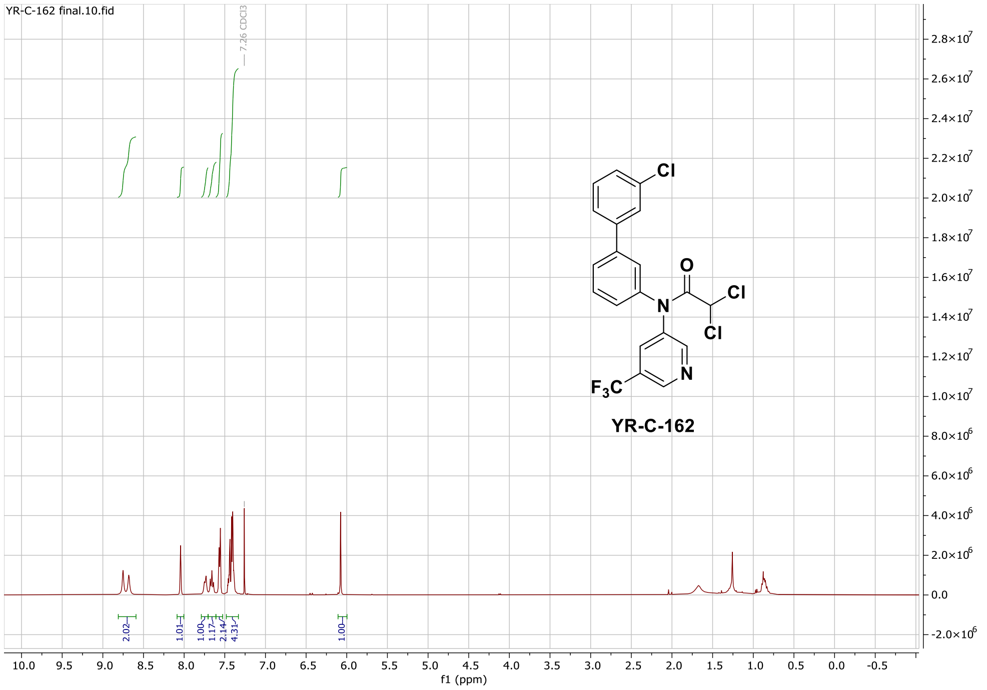

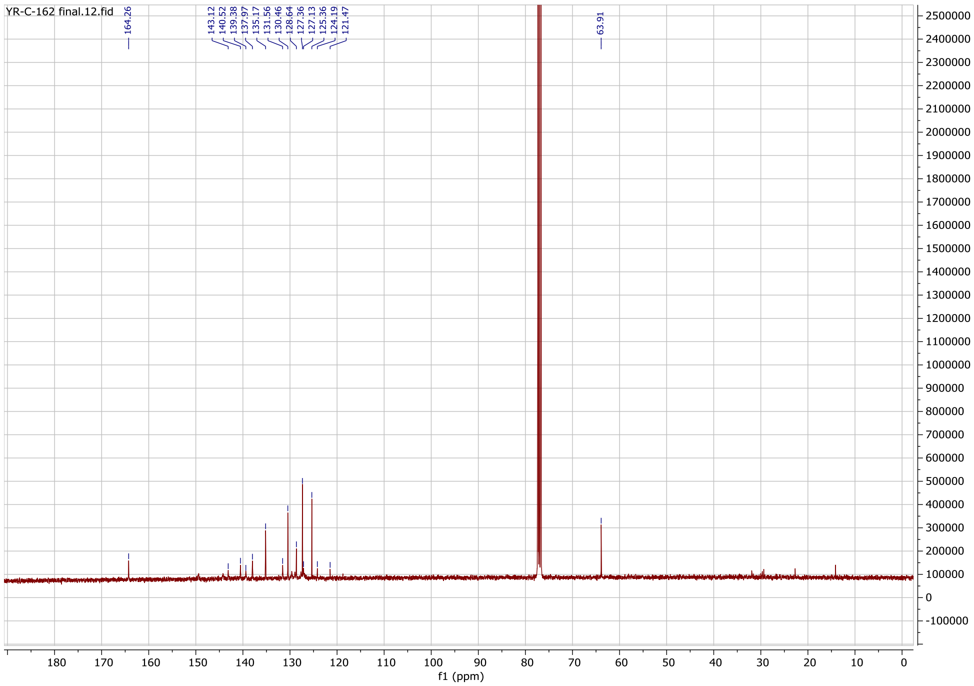

Synthesis Fig. S41. ^{1}H NMR and ^{13}C NMR Spectra of YR-C-162 in DMSO-d_6

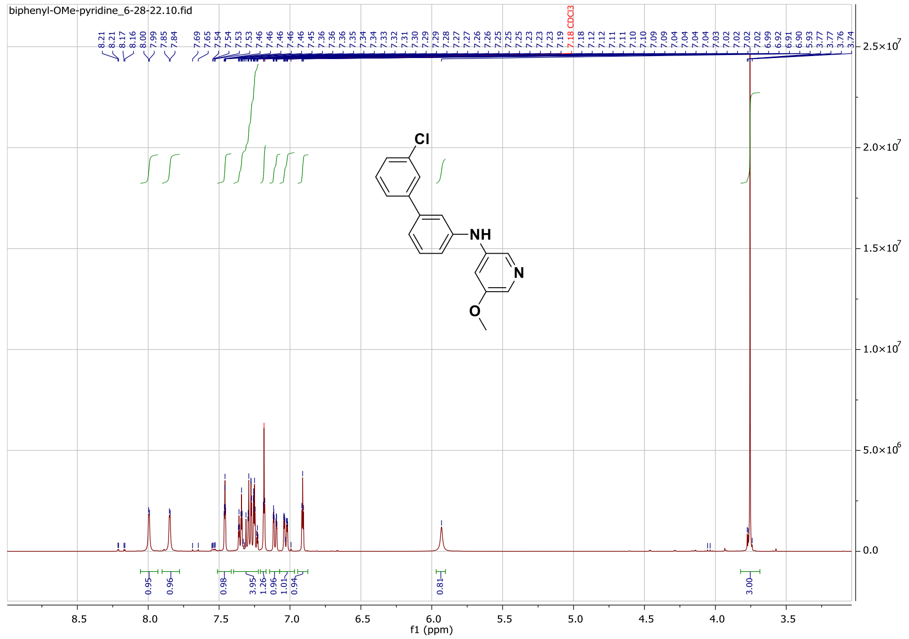

Synthesis Fig. S42. ^{1}H NMR Spectra of 14e in DMSO-d_6

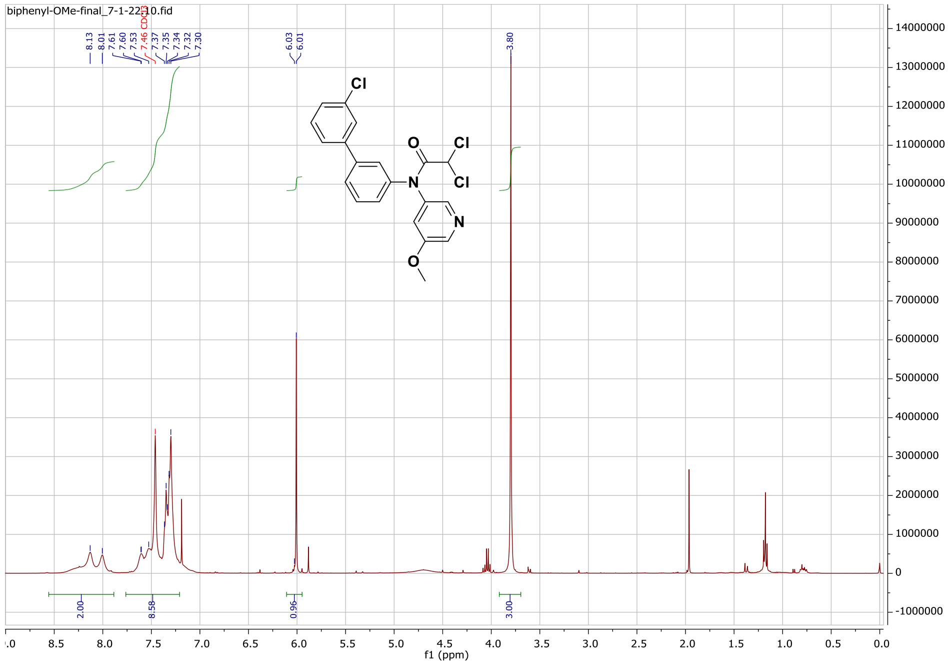

Synthesis Fig. S43. ¹H NMR Spectra of KK108 in DMSO-d_6

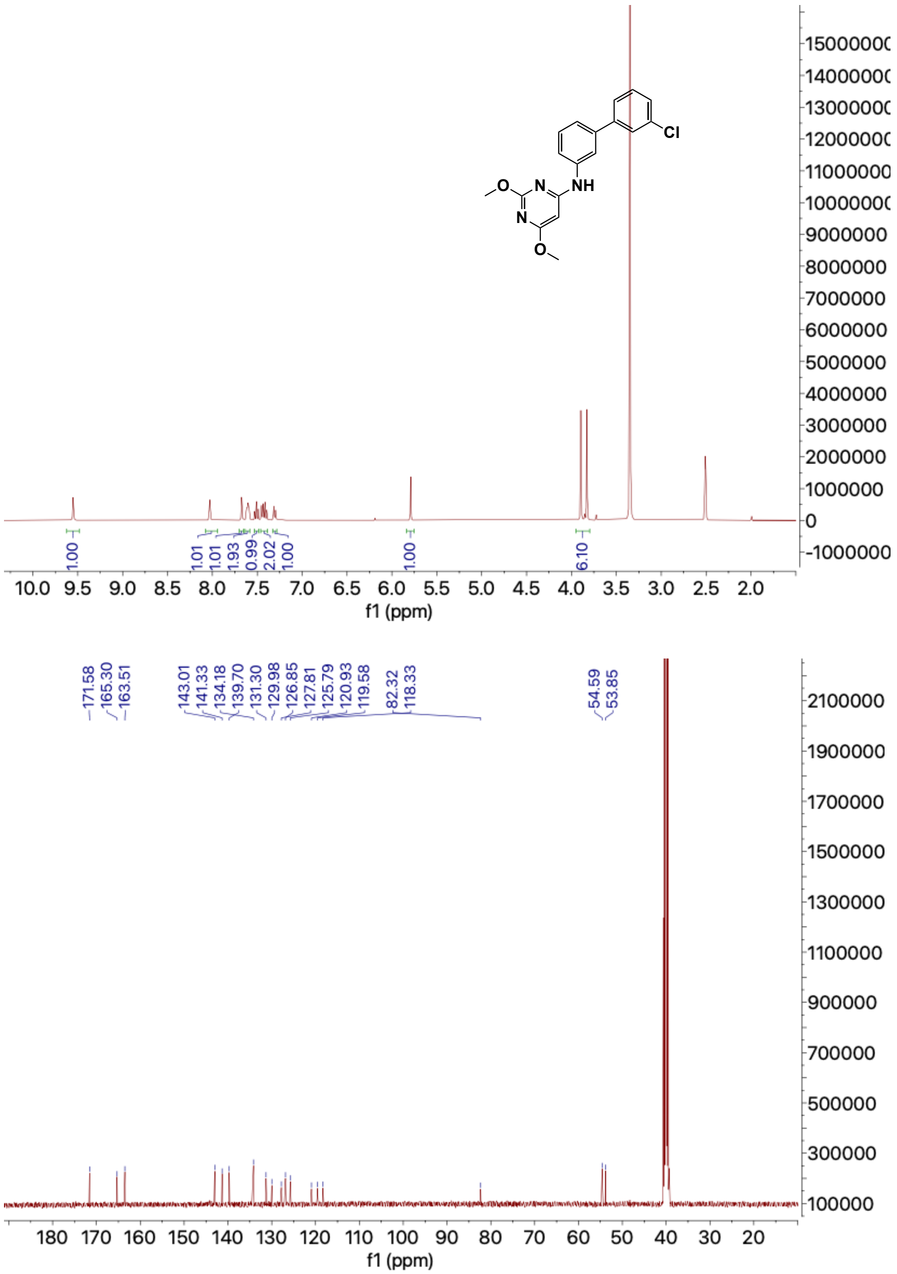

Synthesis Fig. S44. ^{1}H NMR and ^{13}C NMR Spectra of 17 in DMSO-d_6

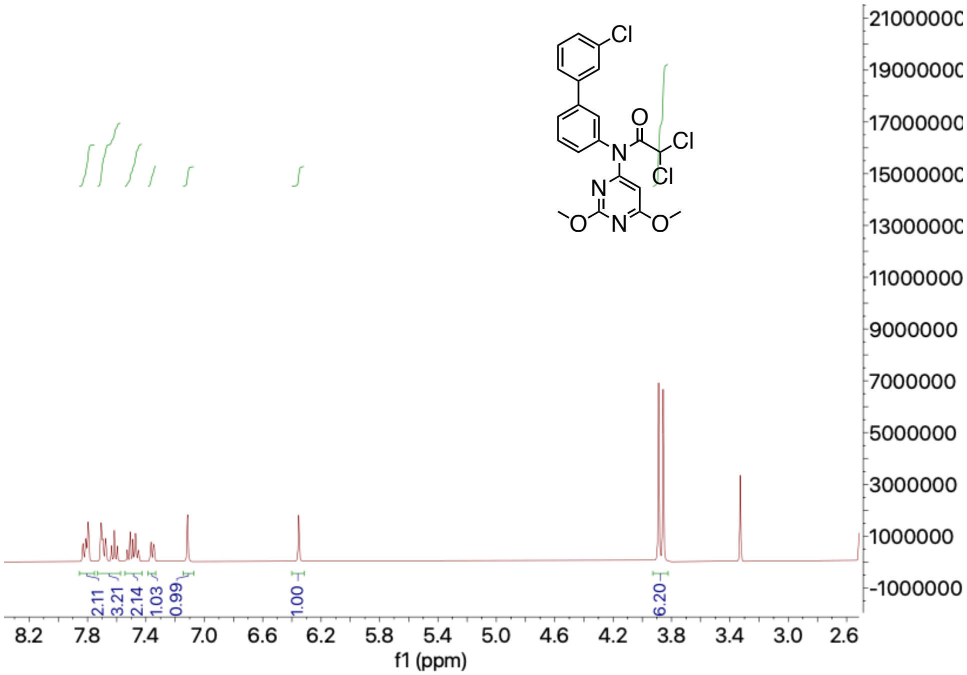

Synthesis Fig. S45. ^{1}H NMR Spectra of VB-C-199 in DMSO-d_6

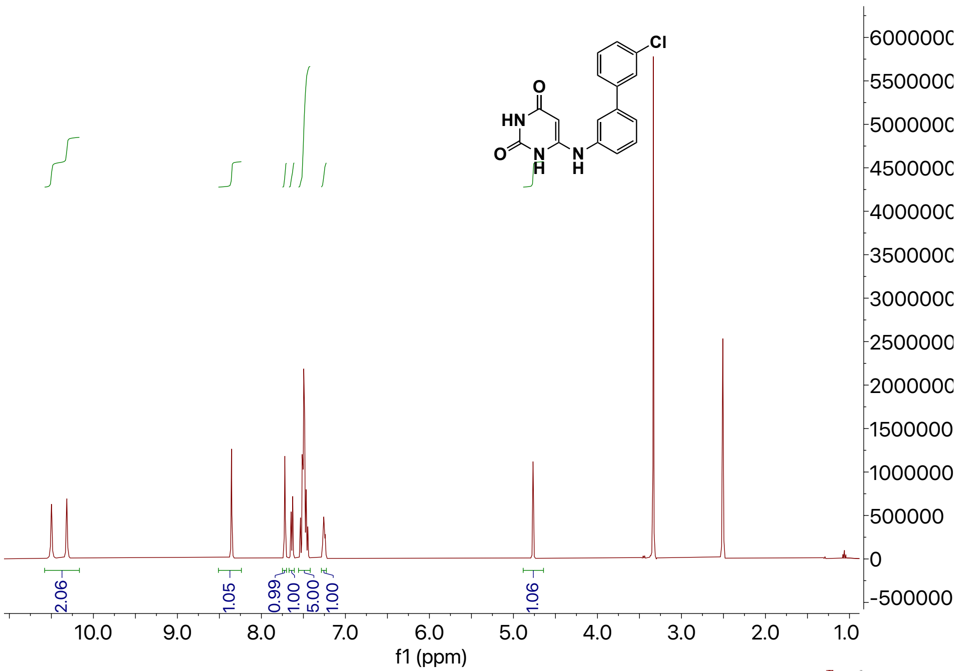

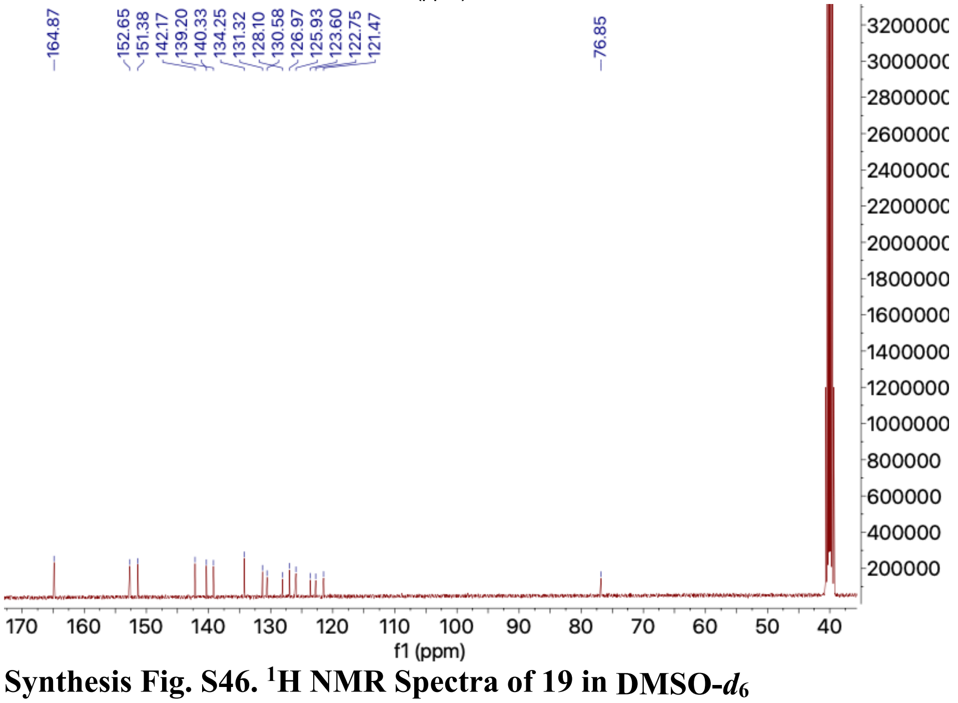

Synthesis Fig. S46. ^{1}H NMR Spectra of 19 in DMSO-*d*$_6$

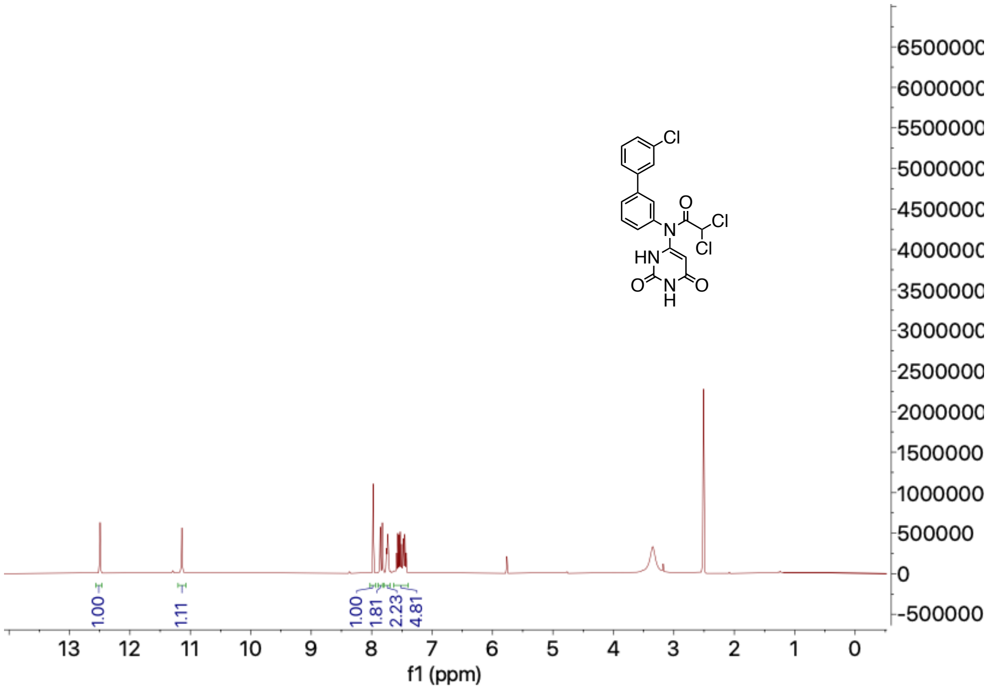

Synthesis Fig. S47. ^{1}H NMR Spectra of VB-D-24 in DMSO-d_6

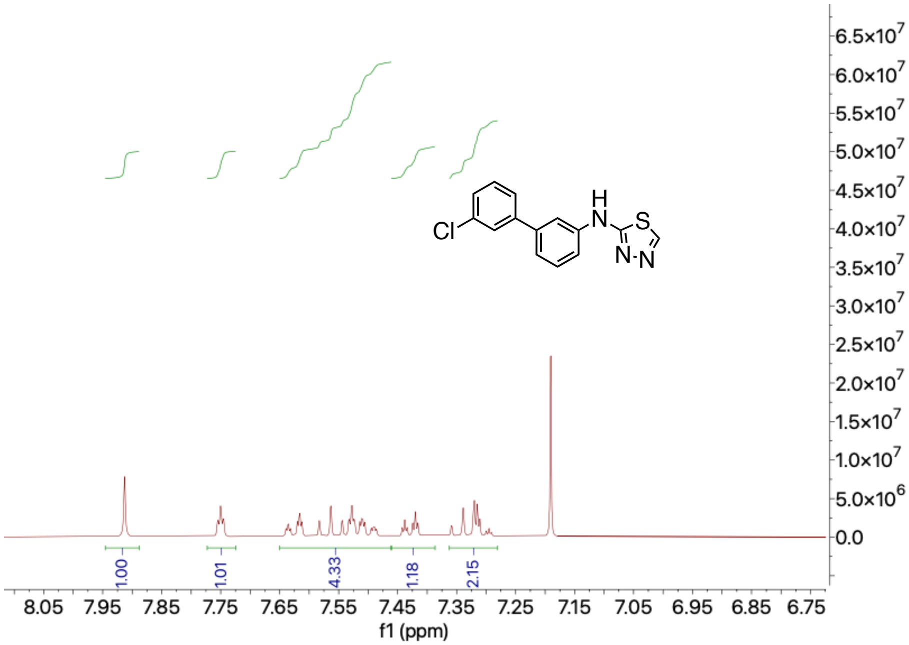

Synthesis Fig. S48. ^{1}H NMR Spectra of 21 in DMSO-d_6

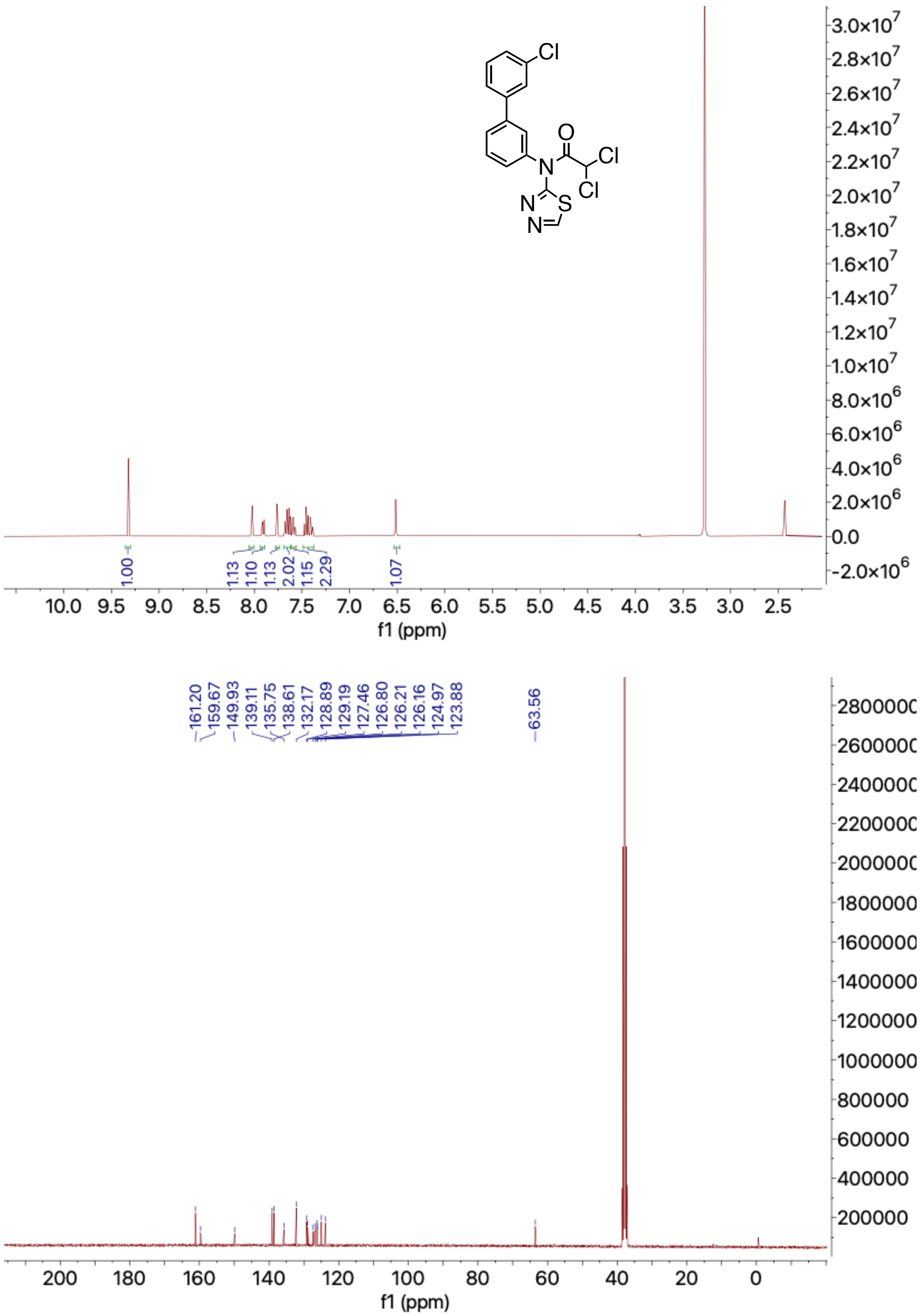

Synthesis Fig. S49. ^{1}H NMR and ^{13}C NMR Spectra of VB-C-86 in DMSO-d_6

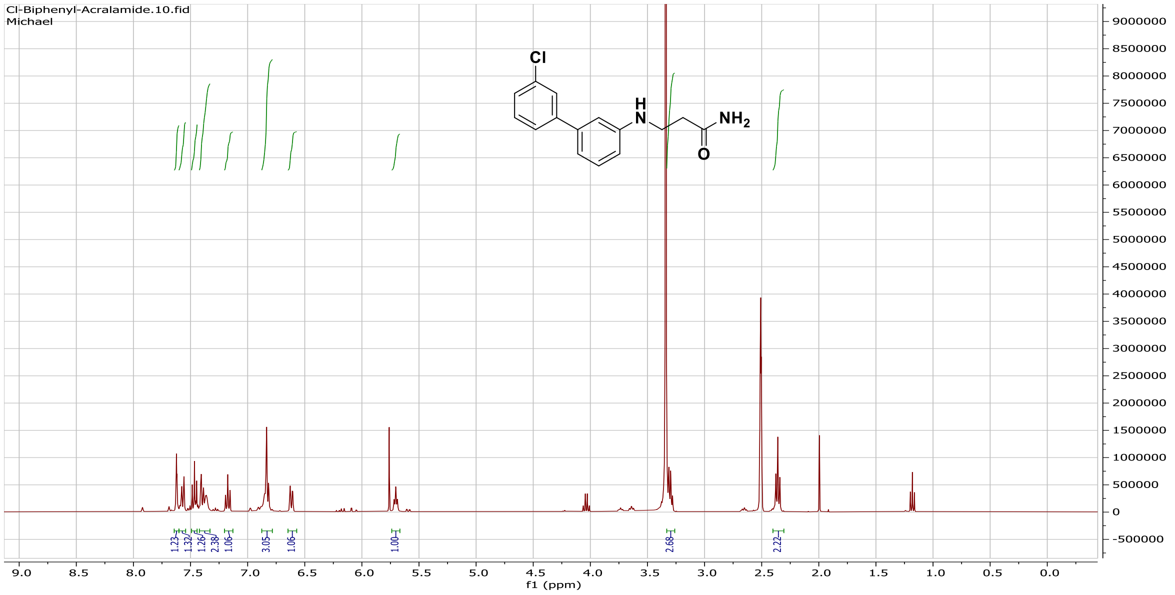

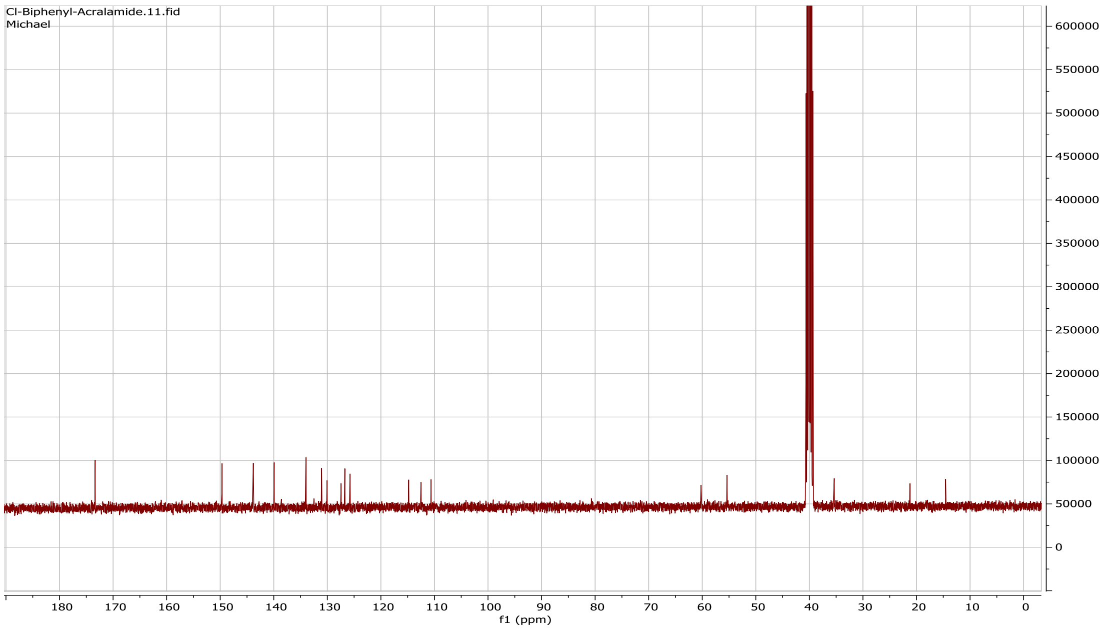

Synthesis Fig. S50. ^{1}H NMR and ^{13}C NMR Spectra of 23 in DMSO-d_6

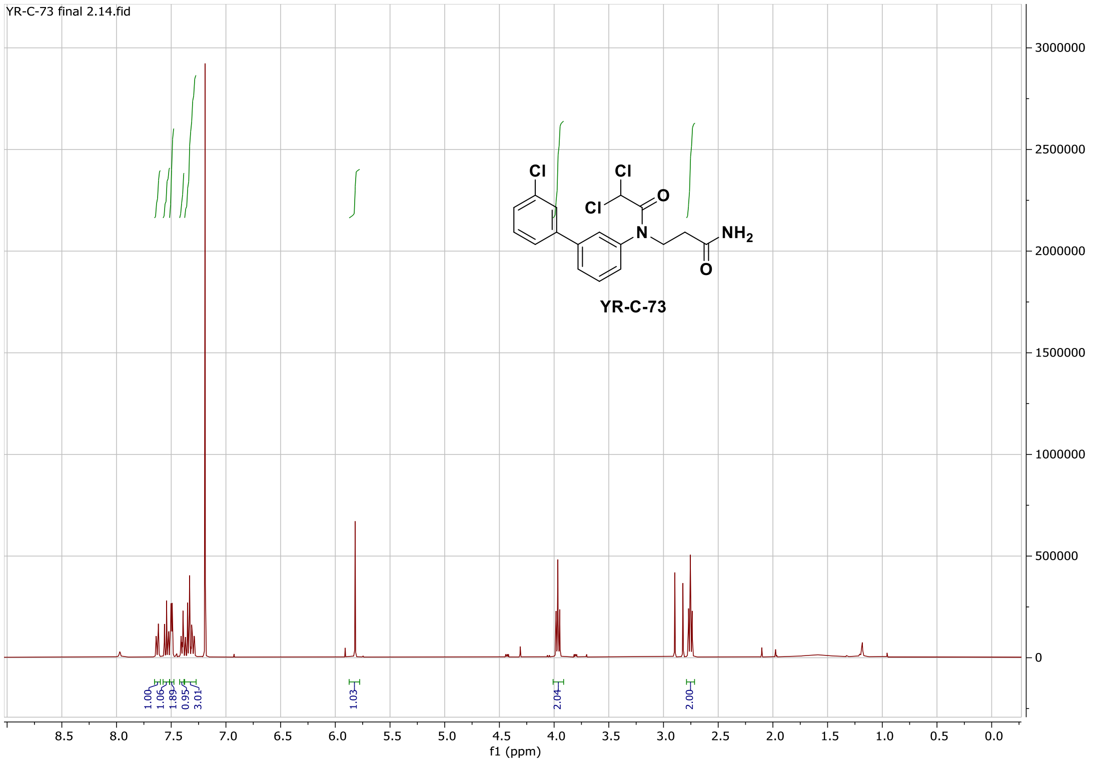

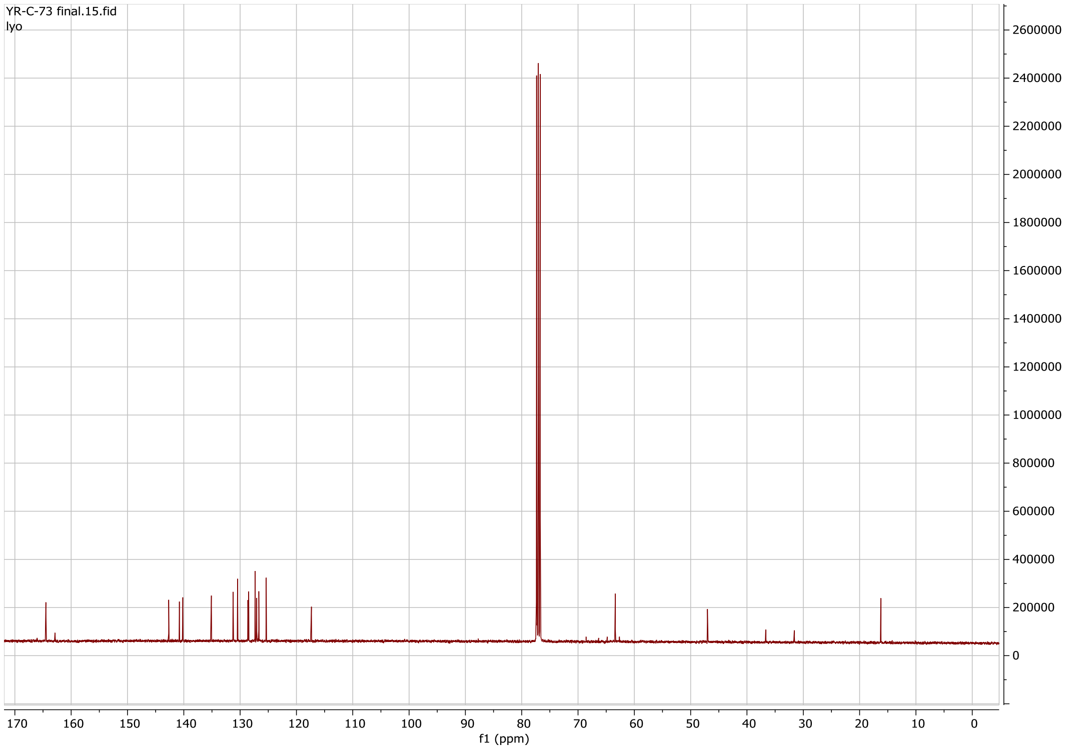

Synthesis Fig. S51. ^{1}H NMR and ^{13}C NMR Spectra of YR-C-73 in DMSO-d_6

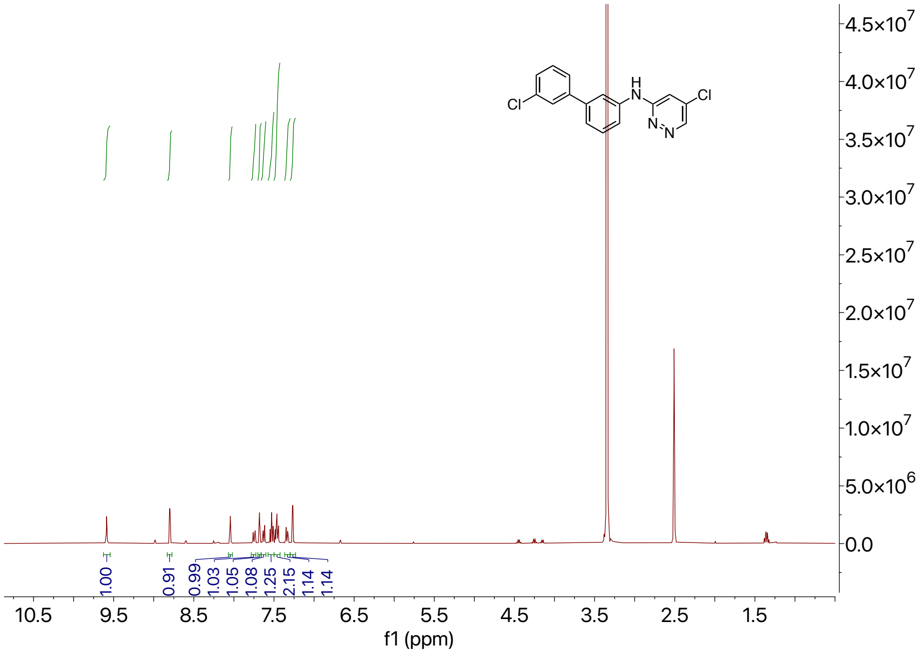

Synthesis Fig. S52. ^{1}H NMR Spectra of 25 in DMSO-d_6

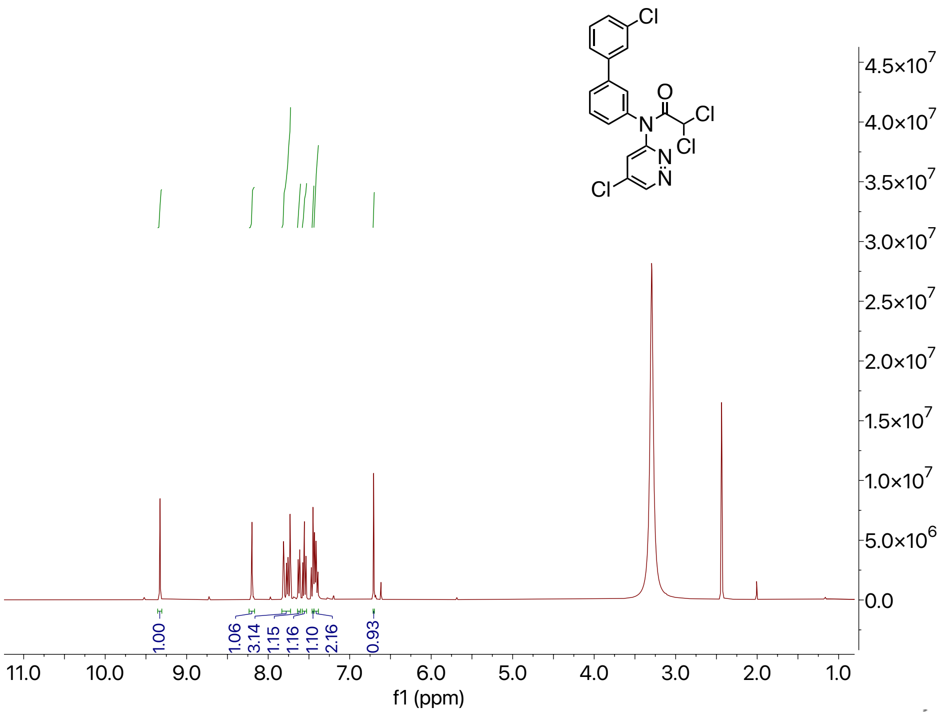

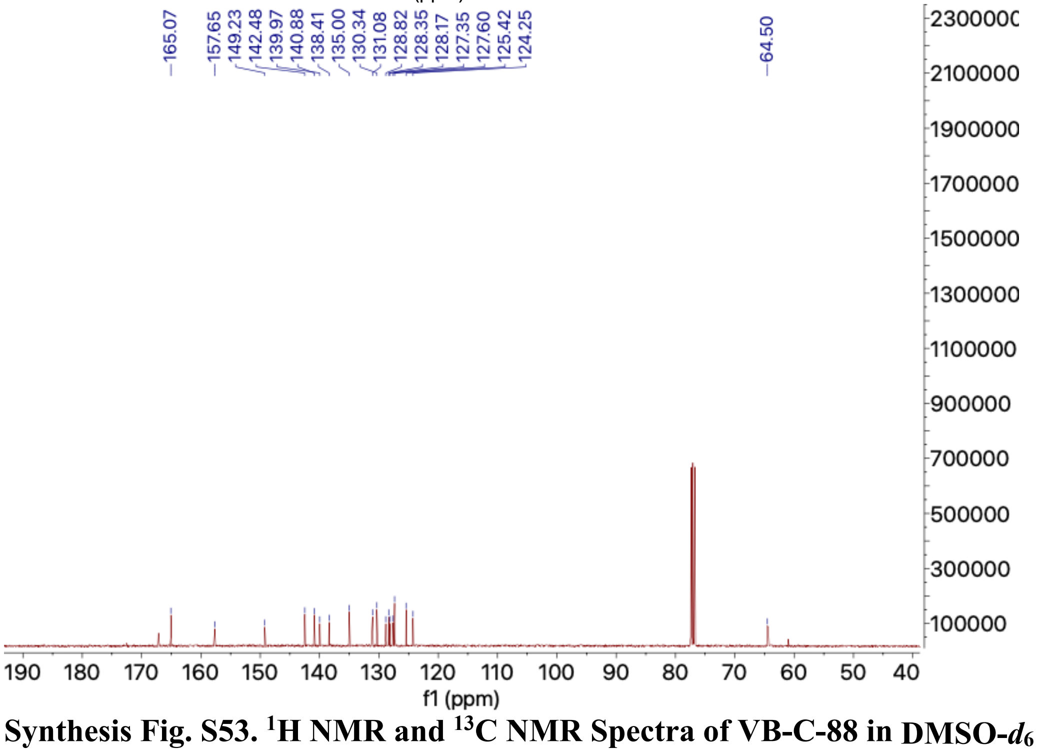

Synthesis Fig. S53. ^{1}H NMR and ^{13}C NMR Spectra of VB-C-88 in DMSO-d_6

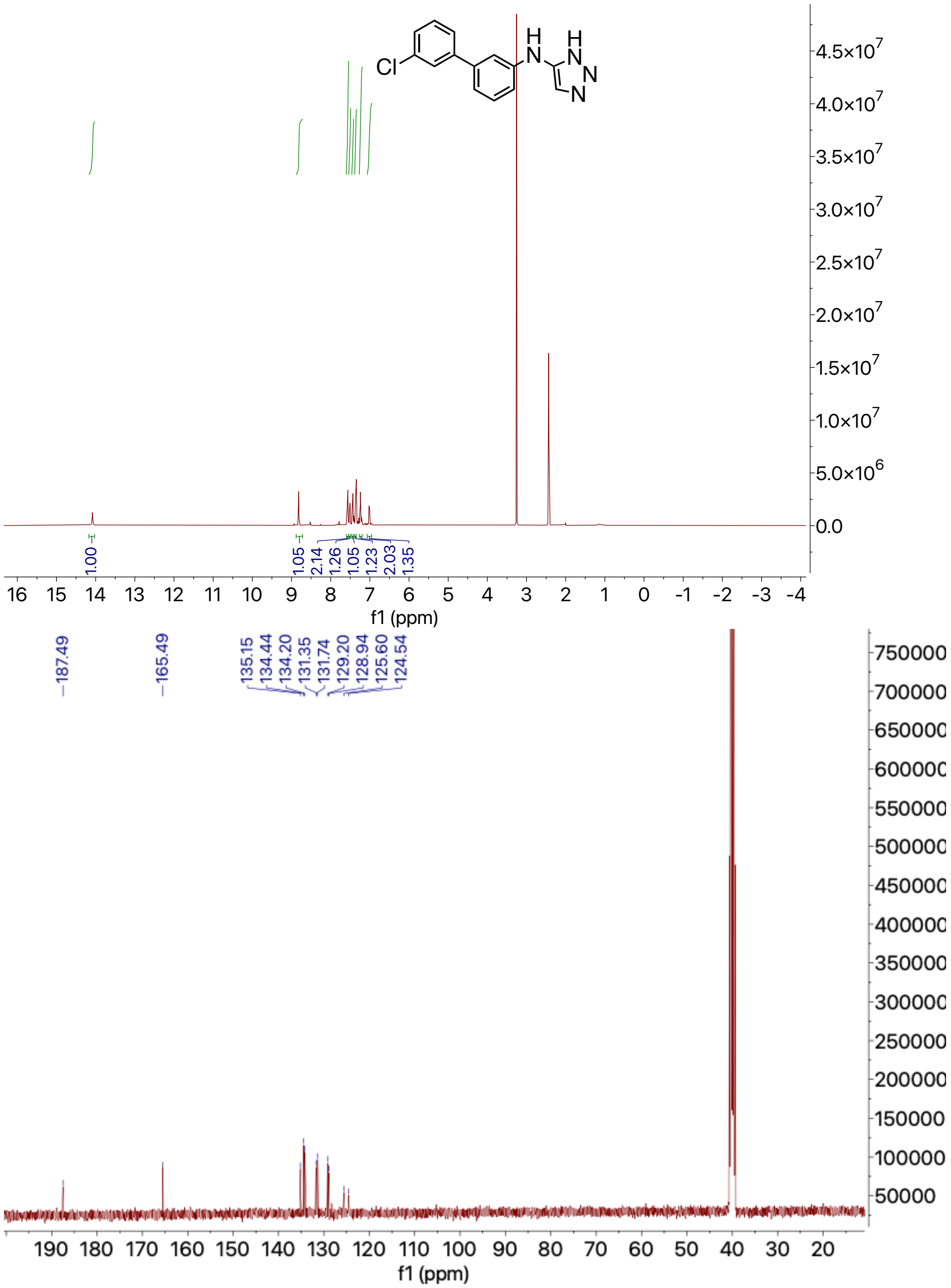

Synthesis Fig. S54. ^{1}H NMR and ^{13}C NMR Spectra of 27 in DMSO-d_6

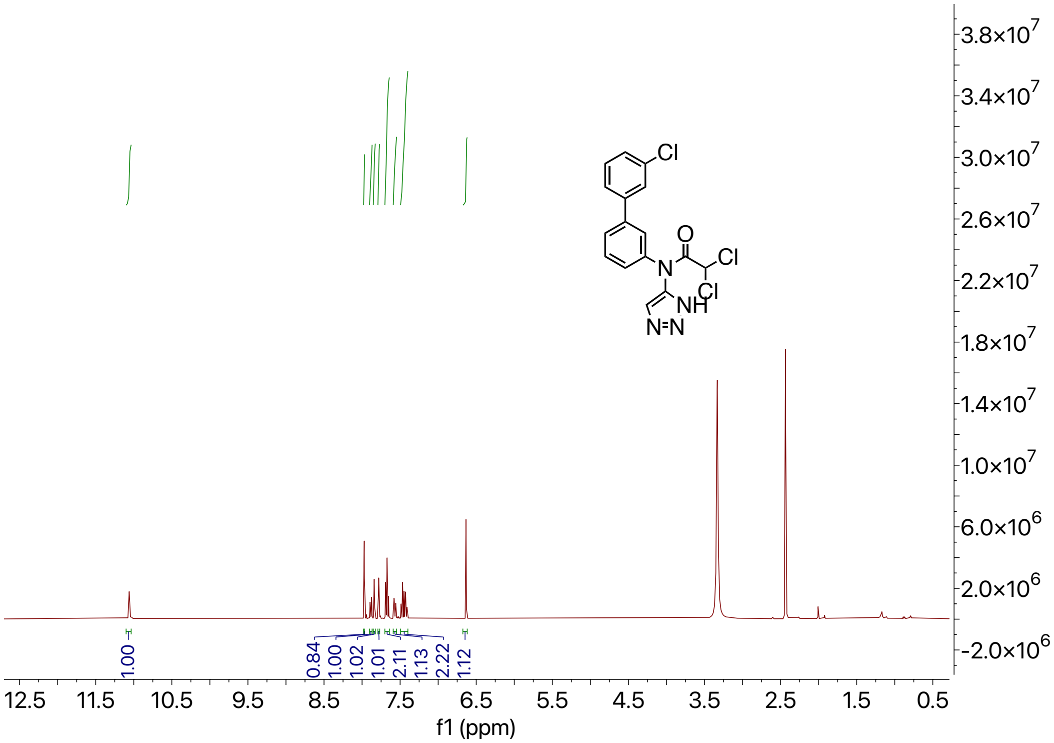

Synthesis Fig. S55. ^{1}H NMR Spectra of VB-C-98 in DMSO-d_6

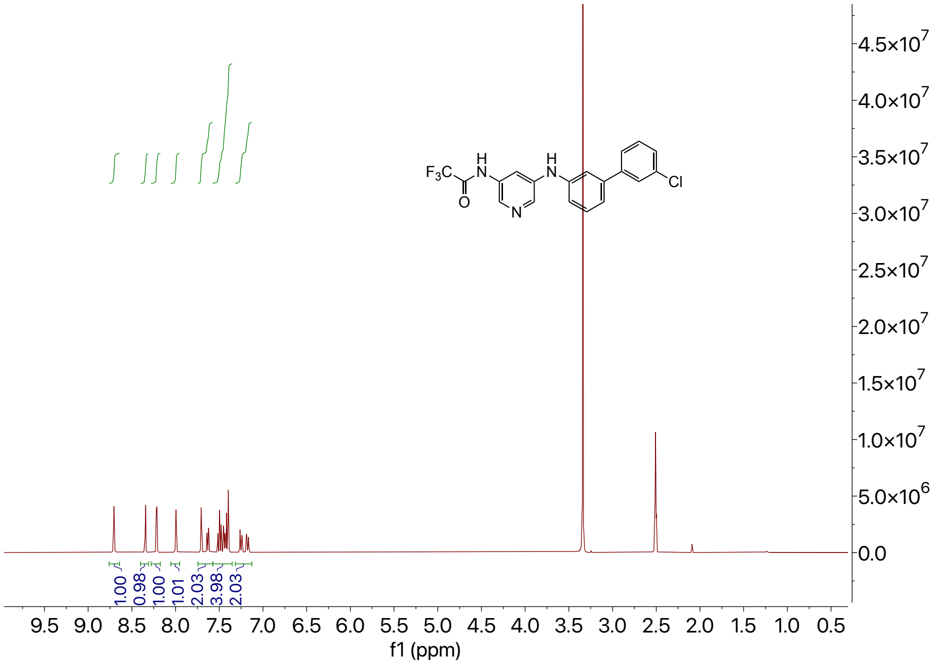

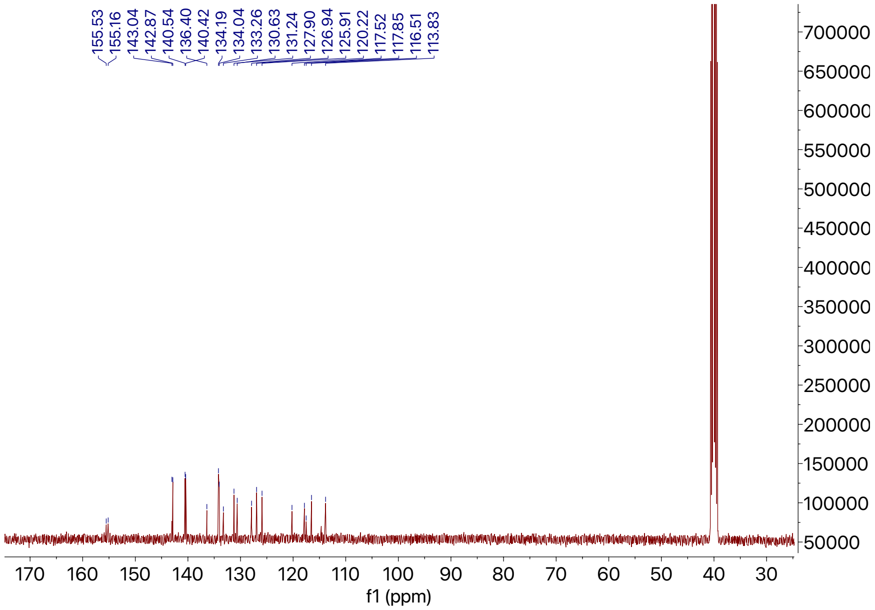

Synthesis Fig. S56. ^{1}H NMR and ^{13}C NMR Spectra of 28 in DMSO-*d*$_6$

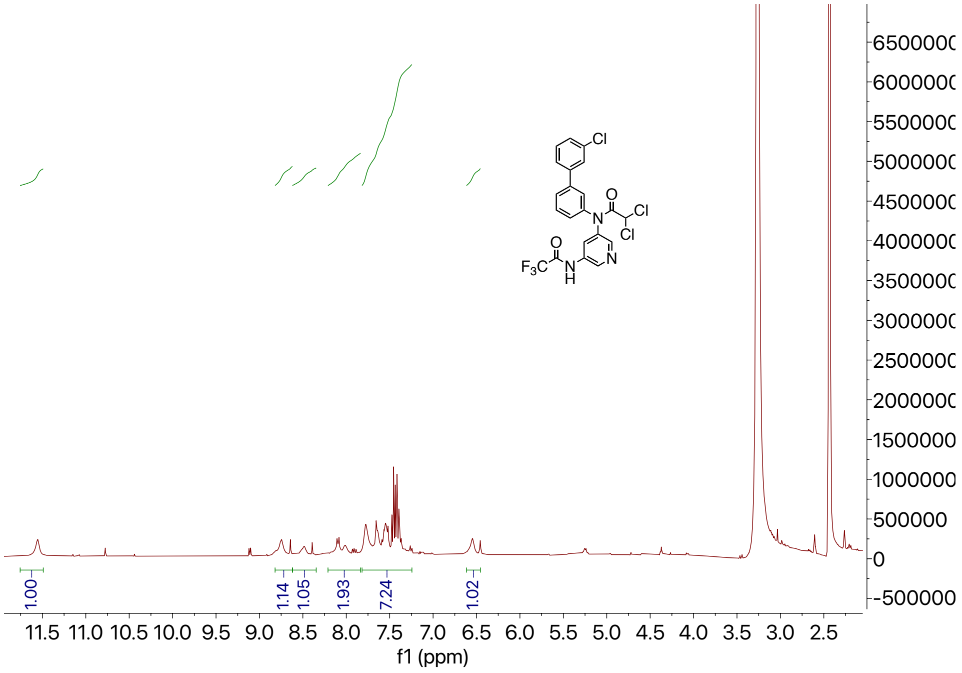

Synthesis Fig. S57. ^{1}H NMR Spectra of VB-C-147 in DMSO-*d*$_6$

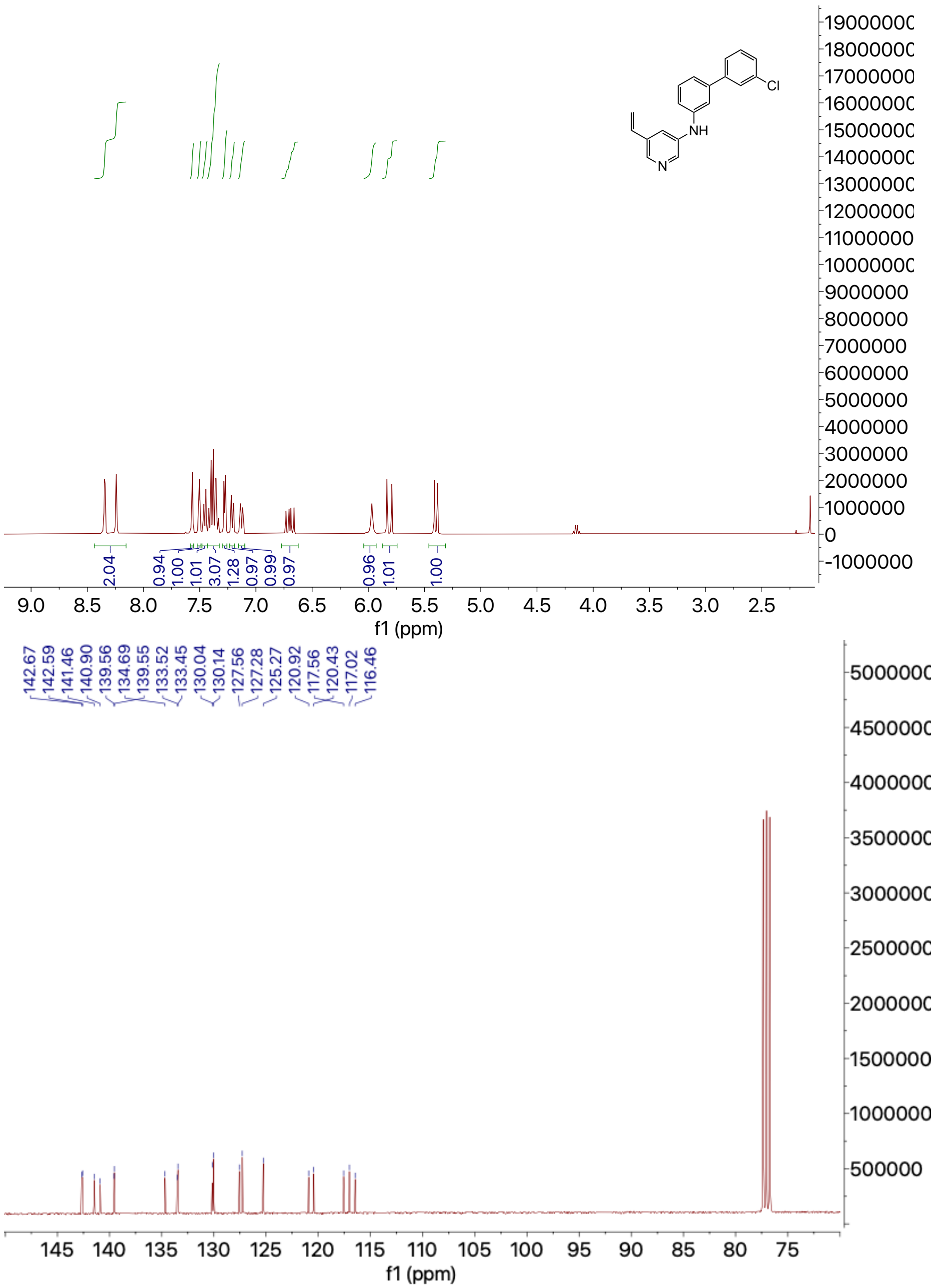

Synthesis Fig. S58. ^{1}H NMR and ^{13}C NMR Spectra of 31 in DMSO-d_6

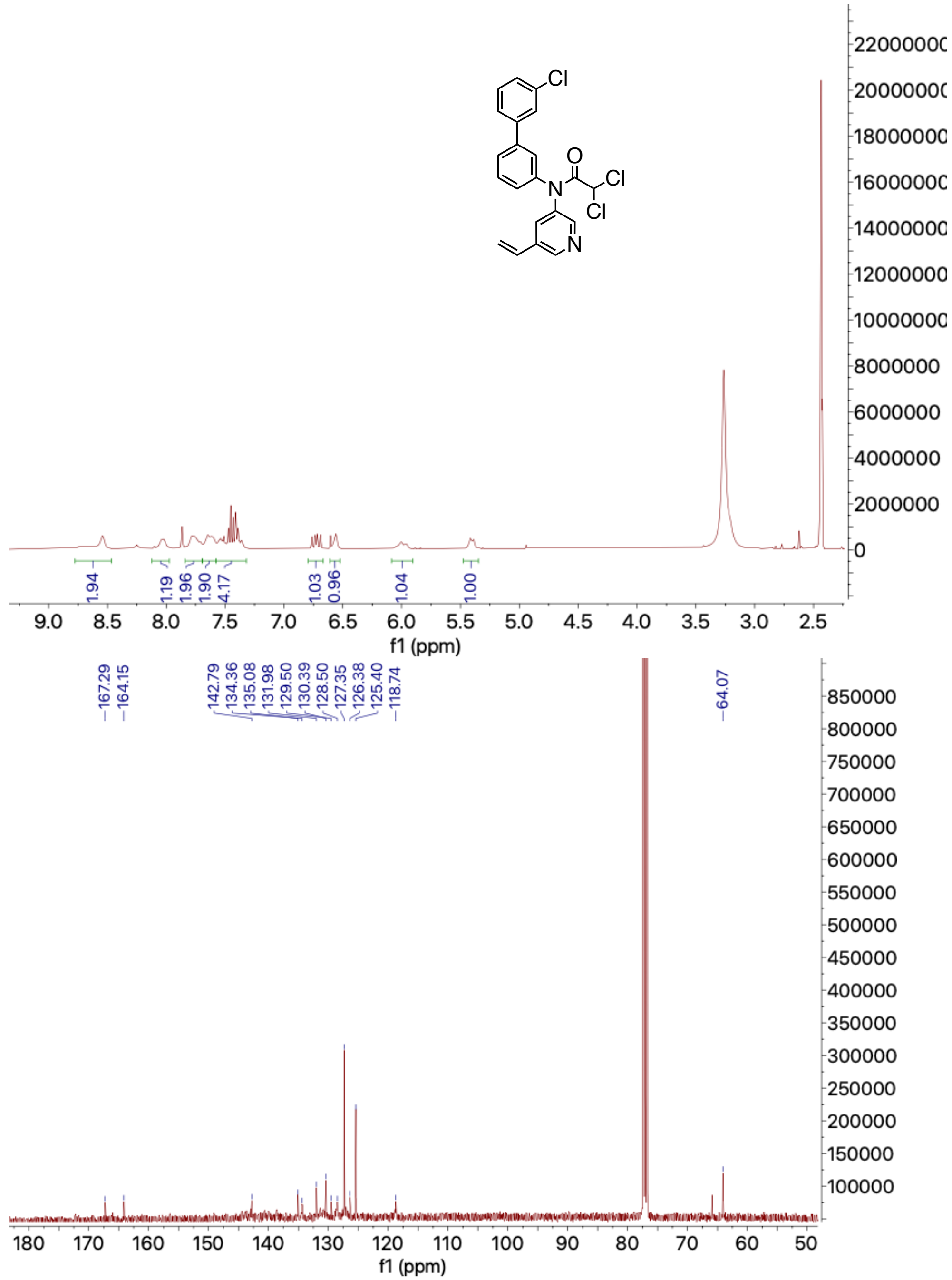

Synthesis Fig. S59. ^{1}H NMR and ^{13}C NMR Spectra of VB-C-120 in DMSO-d_6

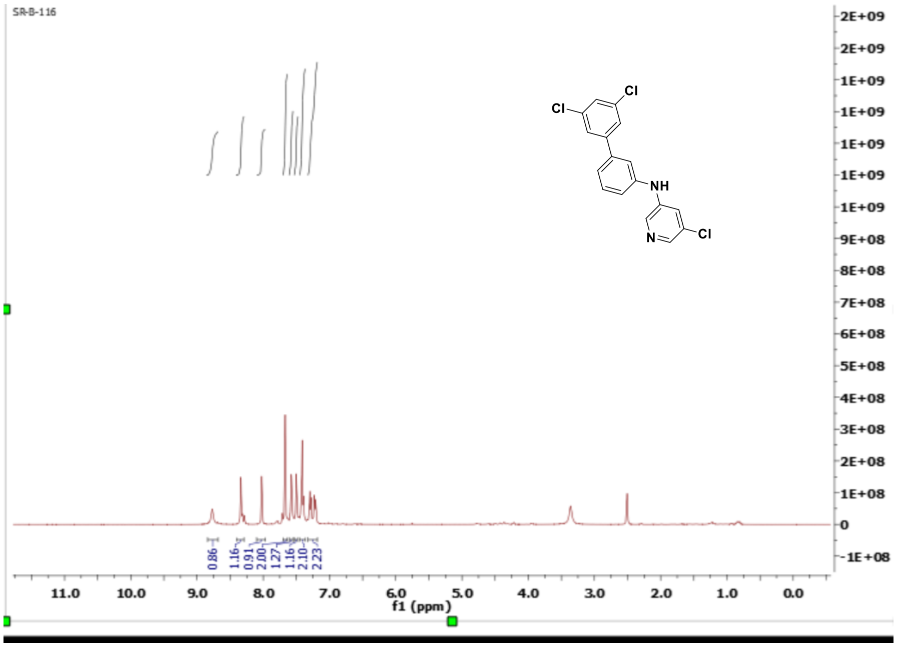

Synthesis Fig. S60. ^{1}H NMR Spectra of 33a in DMSO-d_6

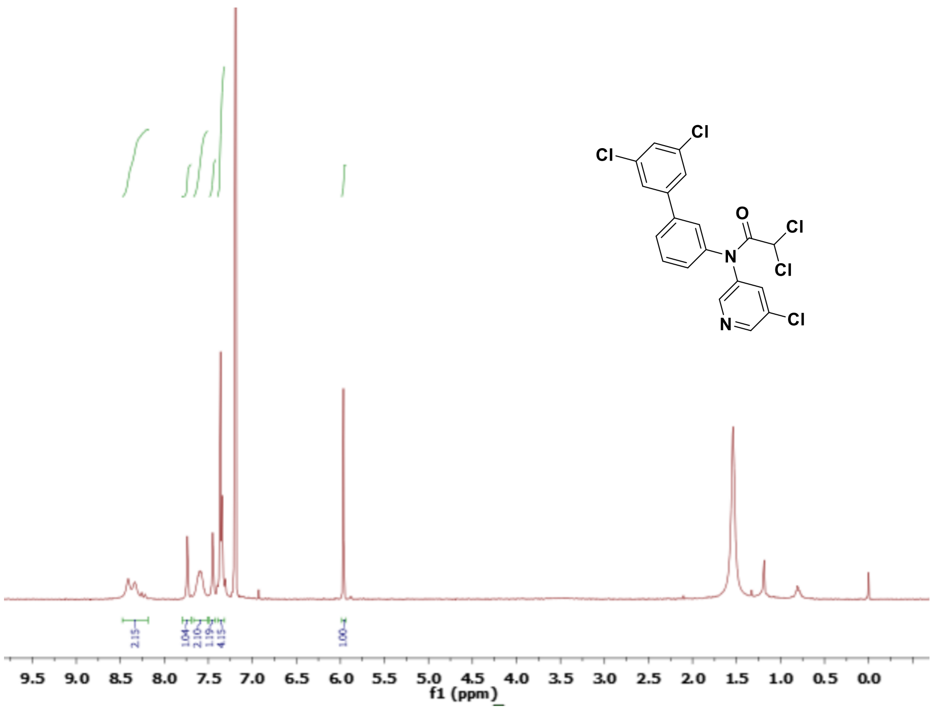

Synthesis Fig. S61. ^{1}H NMR Spectra of SR-B-120 in $CDCl_3$

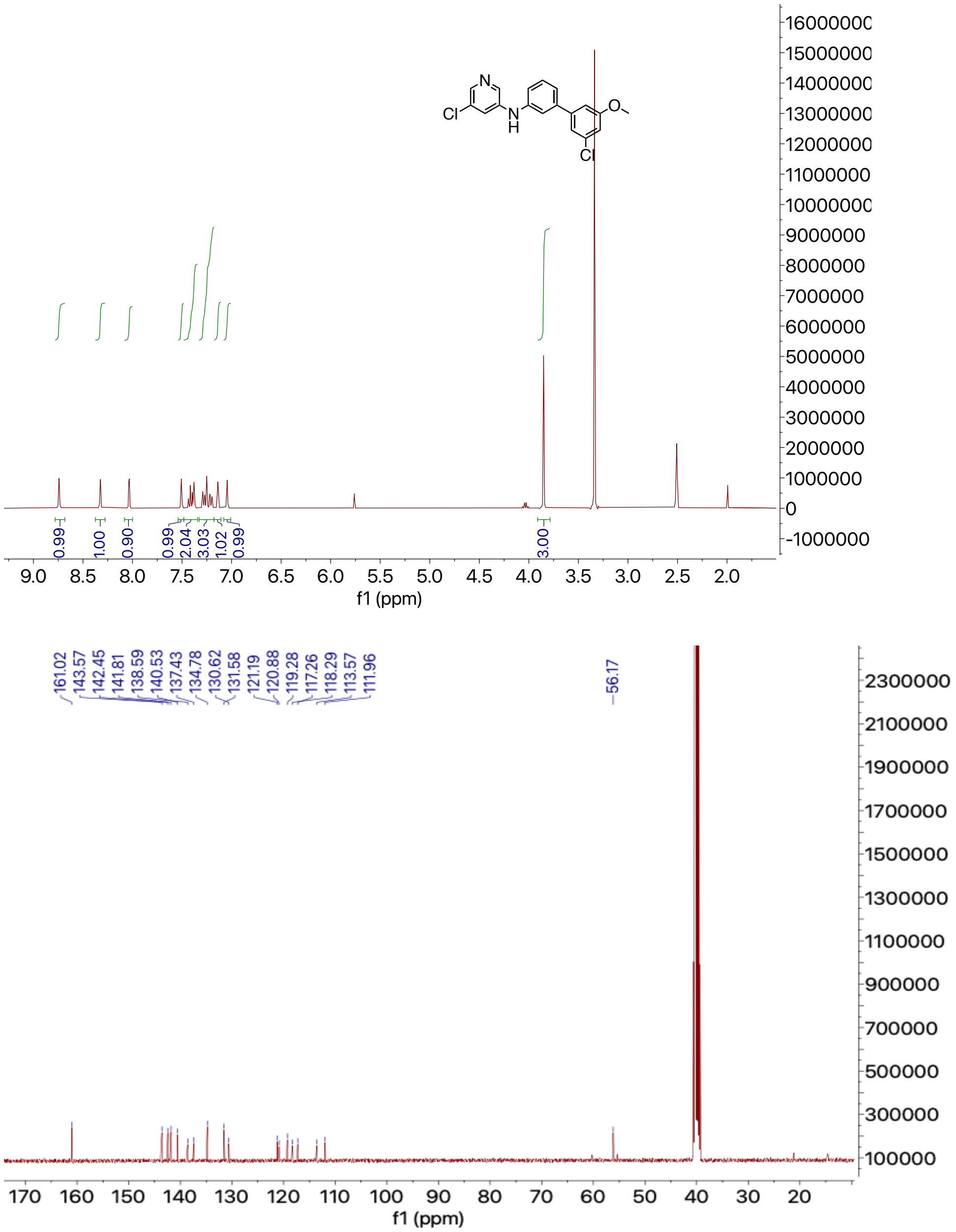

Synthesis Fig. S62. ^{1}H NMR and ^{13}C NMR Spectra of 33b in DMSO-d_6

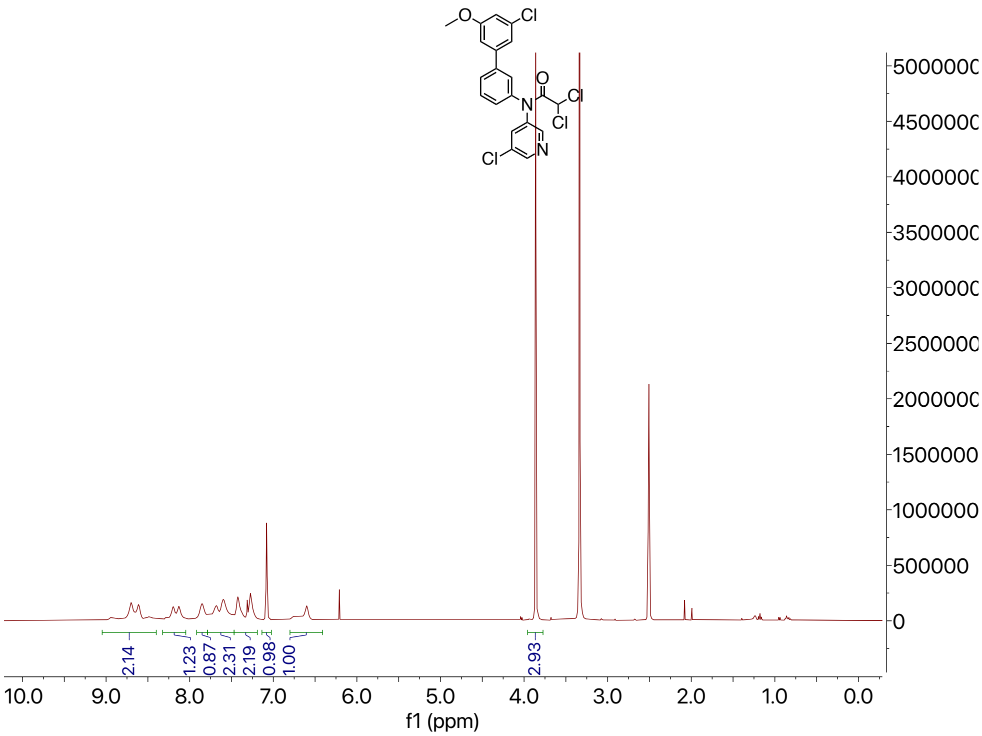

Synthesis Fig. S63. ^{1}H NMR Spectra of VB-C-140 in DMSO-d_6

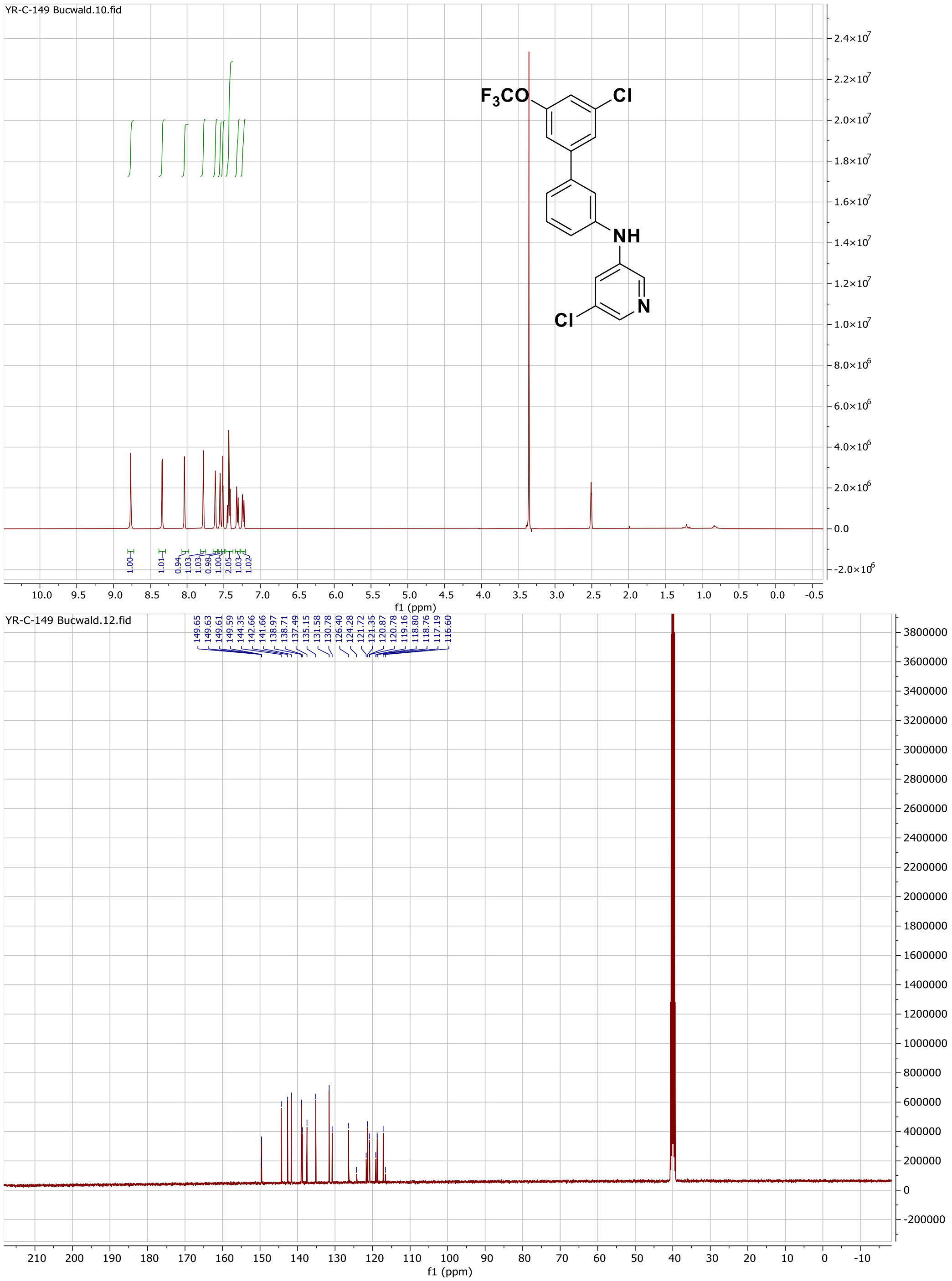

Synthesis Fig. S64. ^{1}H NMR and ^{13}C NMR Spectra of 33c in DMSO-d_6

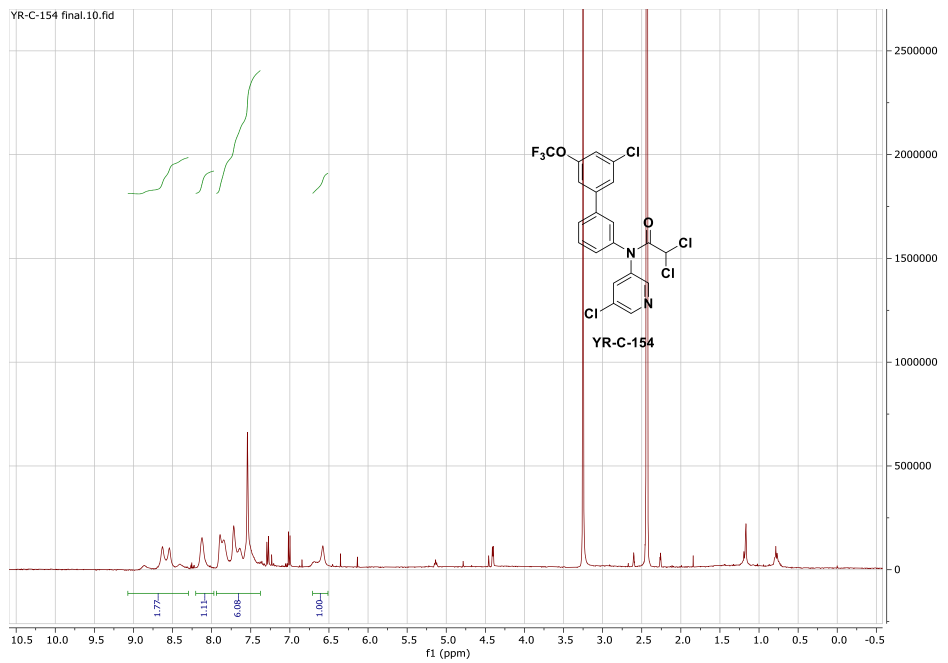

Synthesis Fig. S65. ^{1}H NMR Spectra of YR-C-154 in DMSO-d_6

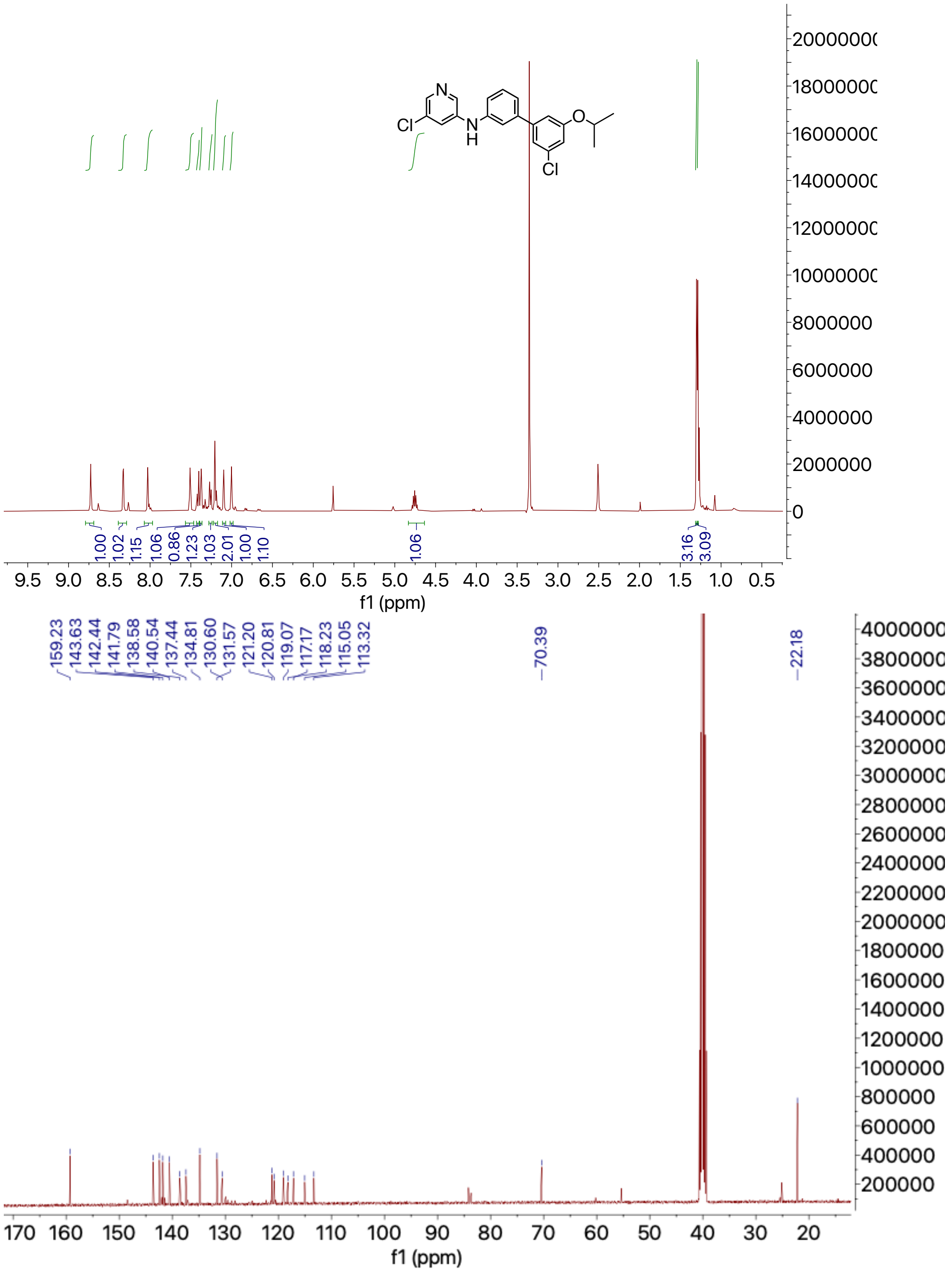

Synthesis Fig. S66. ^{1}H NMR and ^{13}C NMR Spectra of 33d in DMSO-d_6

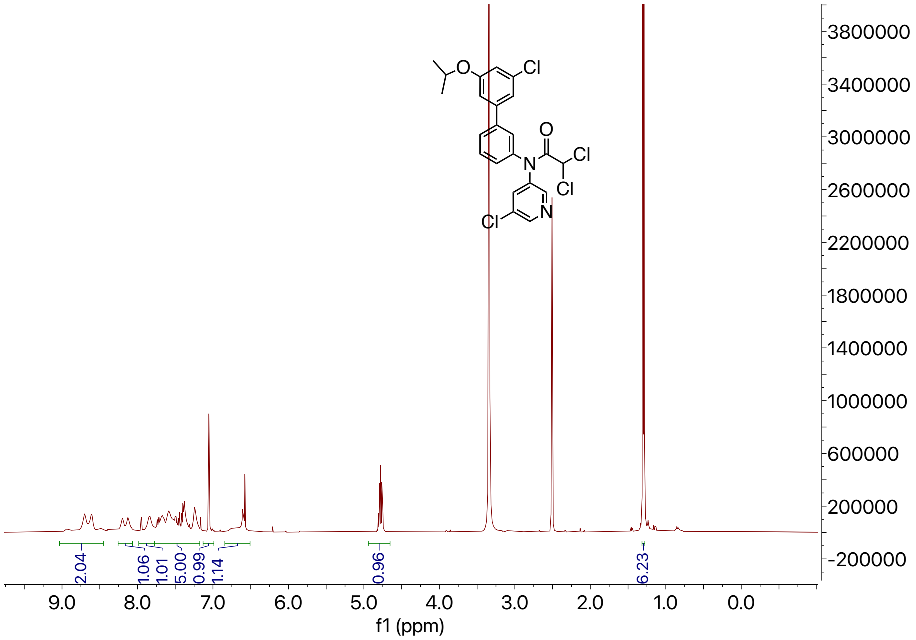

Synthesis Fig. S67. ^{1}H NMR Spectra of VB-C-171 in DMSO-d_6

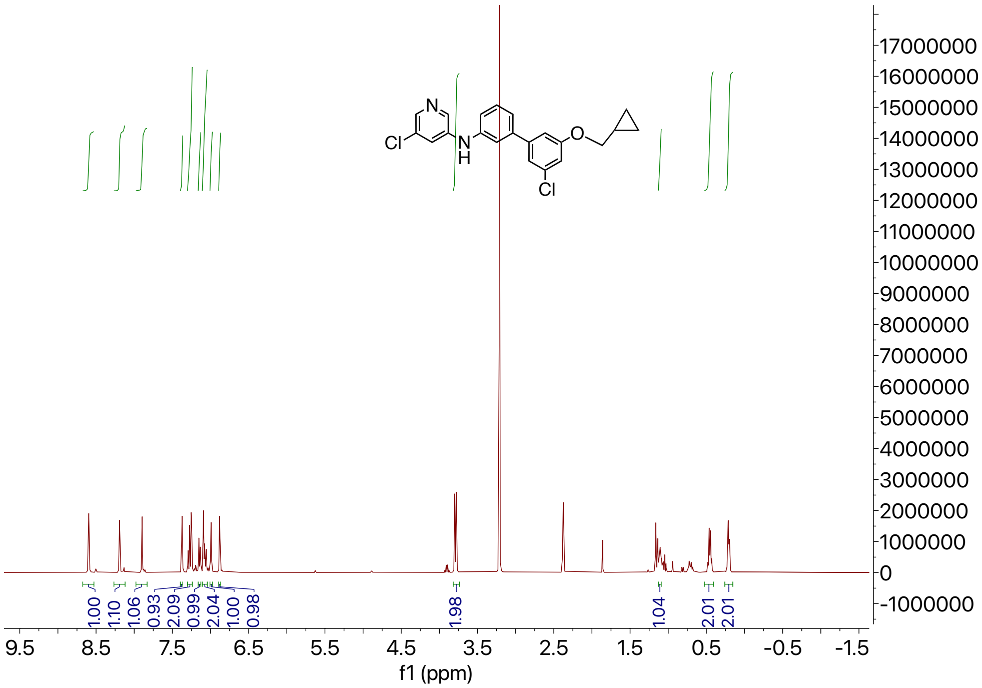

Synthesis Fig. S68. ^{1}H NMR Spectra of 34e in DMSO-d_6

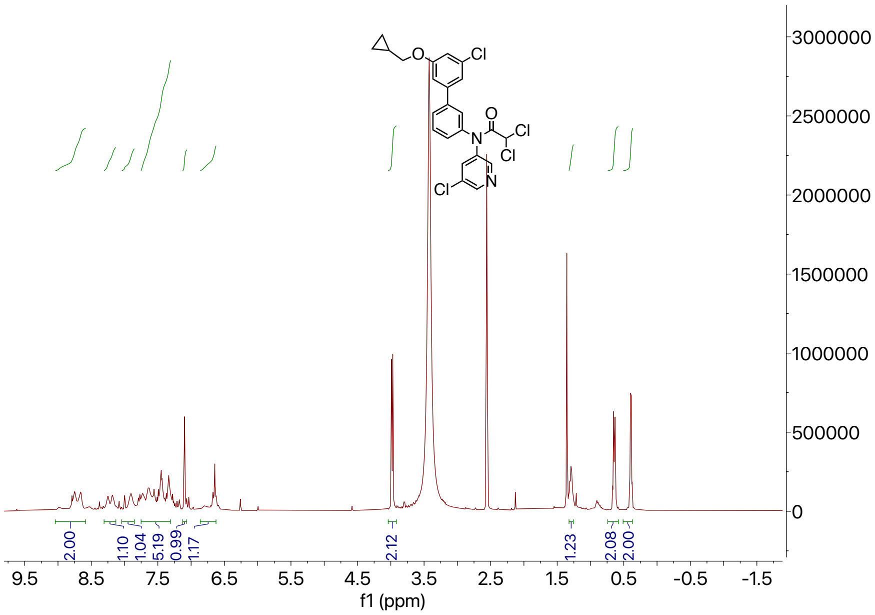

Synthesis Fig. S69. ^{1}H NMR Spectra of VB-C-172 in DMSO-*d*$_6$

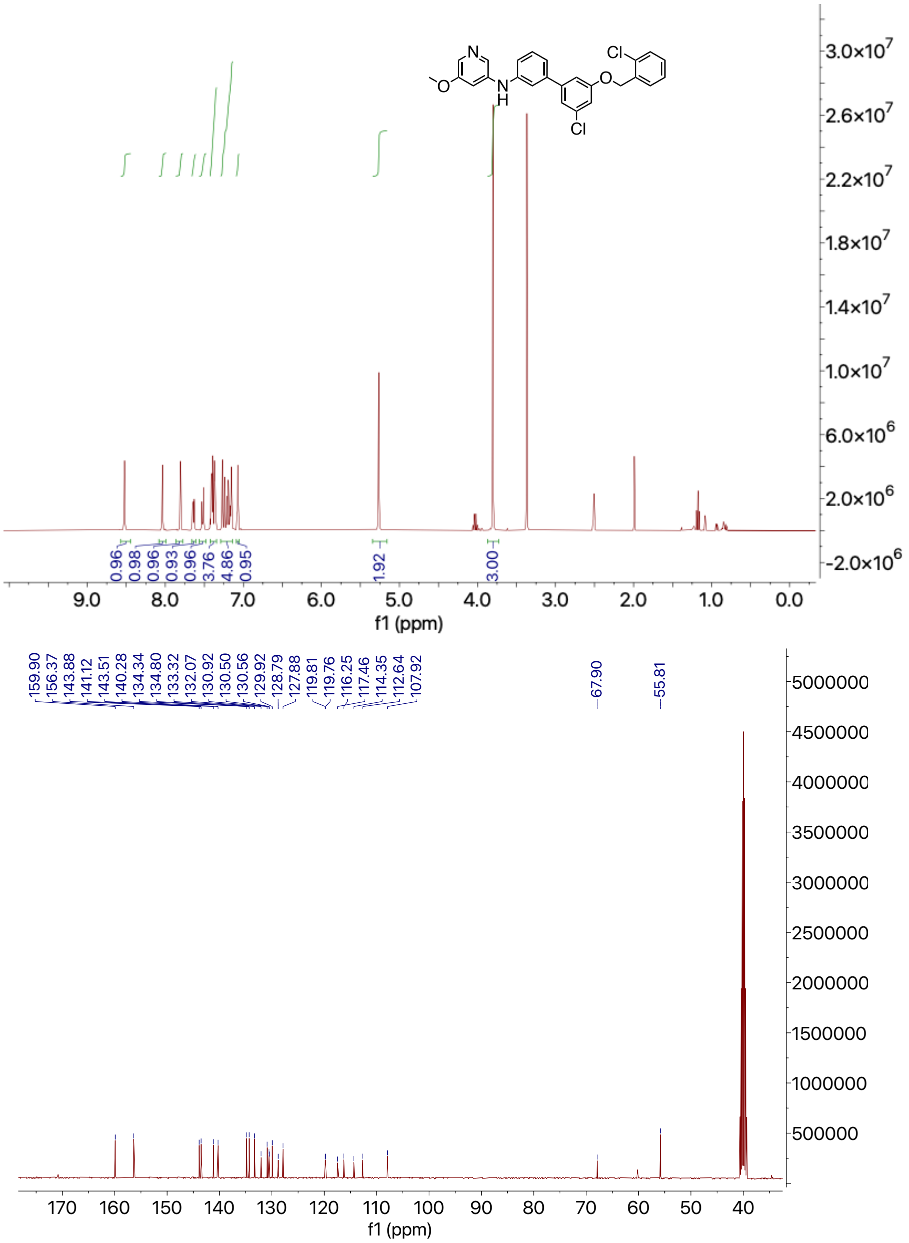

Synthesis Fig. S70. ^{1}H NMR and ^{13}C NMR Spectra of 33f in DMSO-d_6

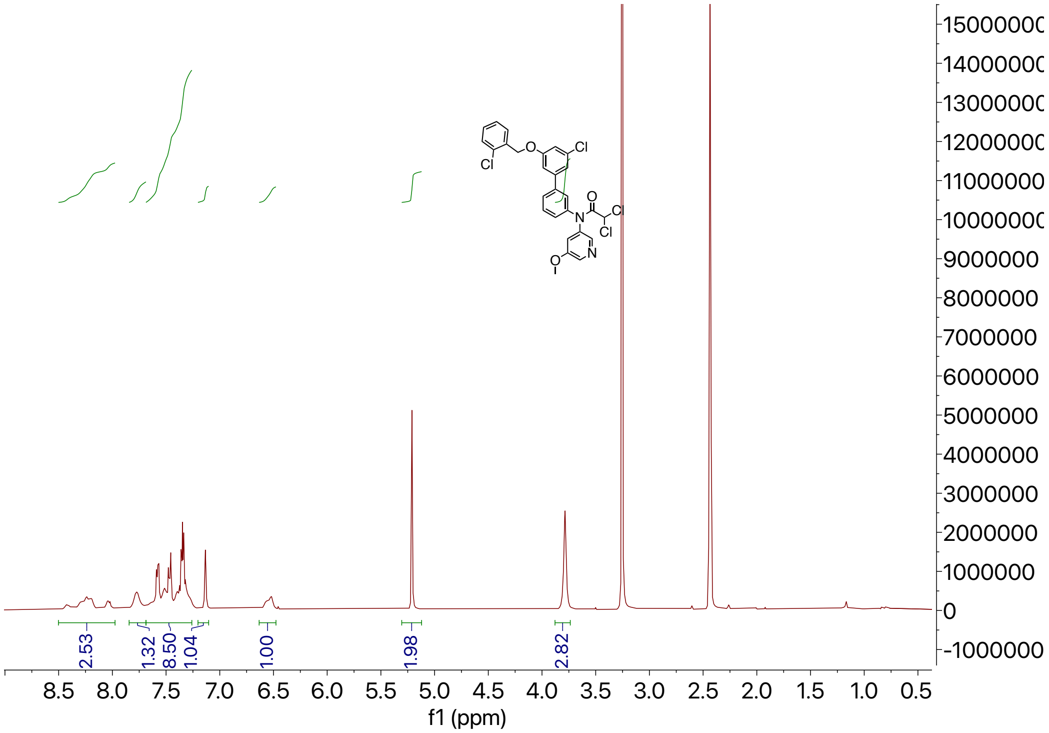

Synthesis Fig. S71. ^{1}H NMR Spectra of VB-C-124 in DMSO-d_6

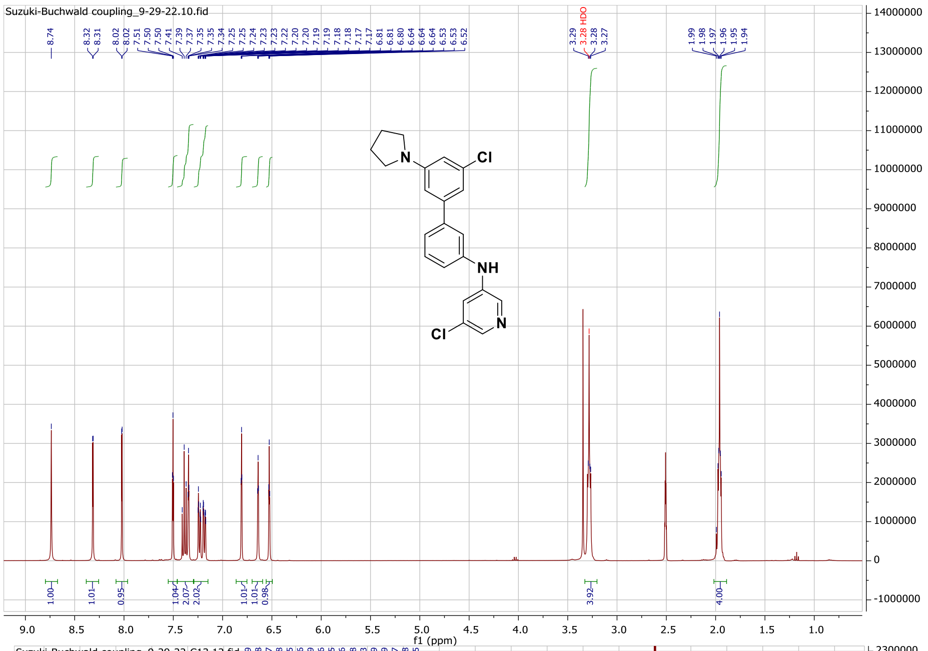

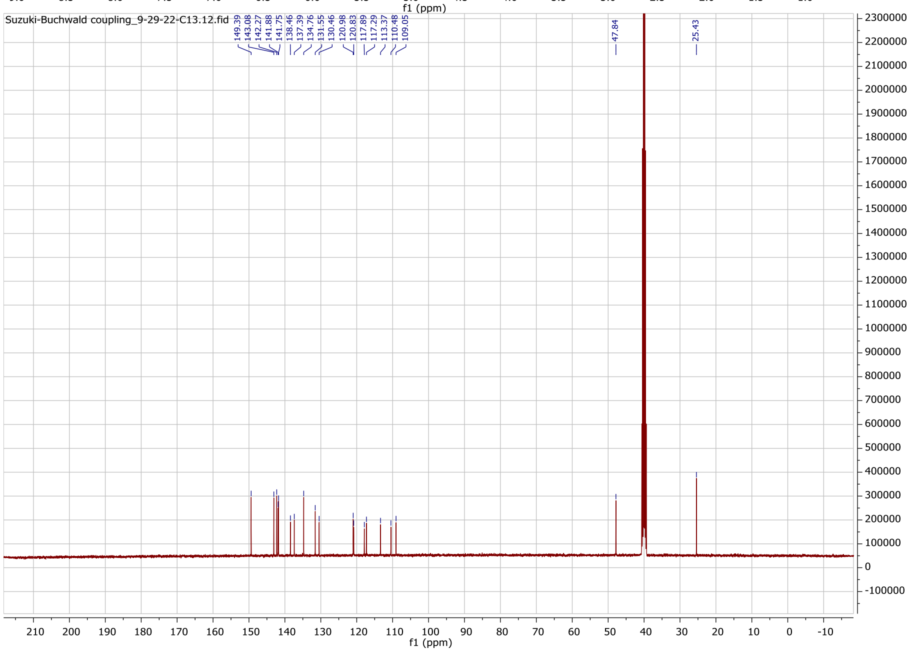

Synthesis Fig. S72. ^{1}H NMR and ^{13}C NMR Spectra of 33g in DMSO-d_6

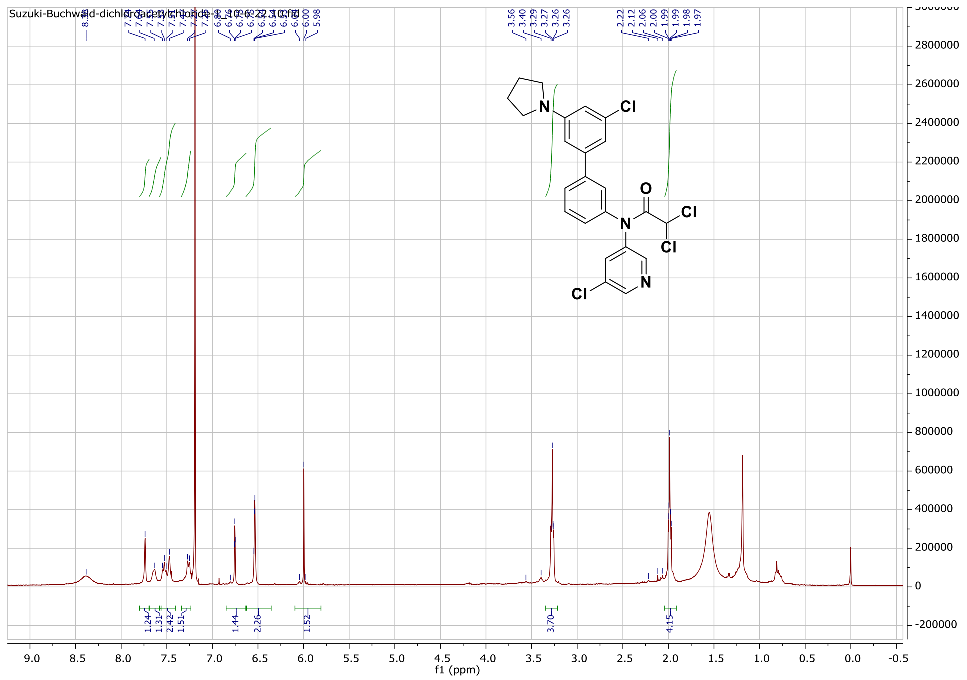

Synthesis Fig. S73. ^{1}H NMR Spectra of KK-114 in DMSO-d_6

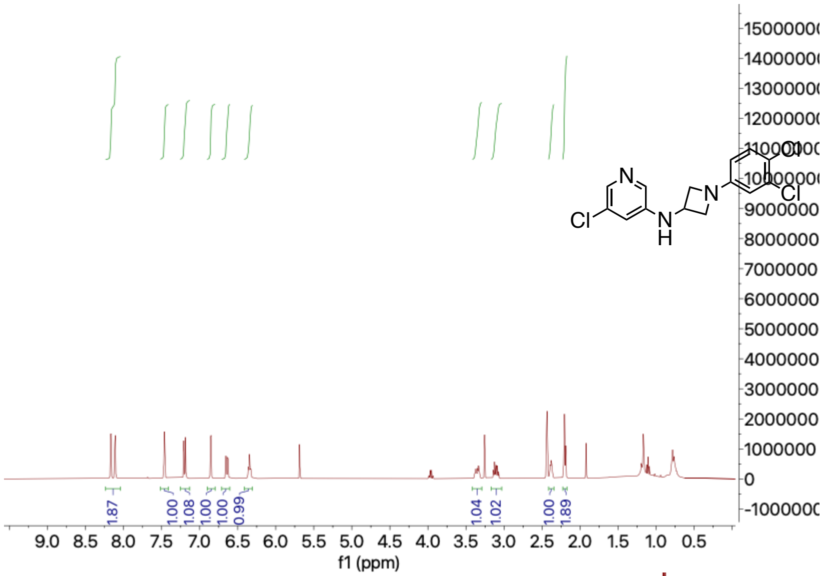

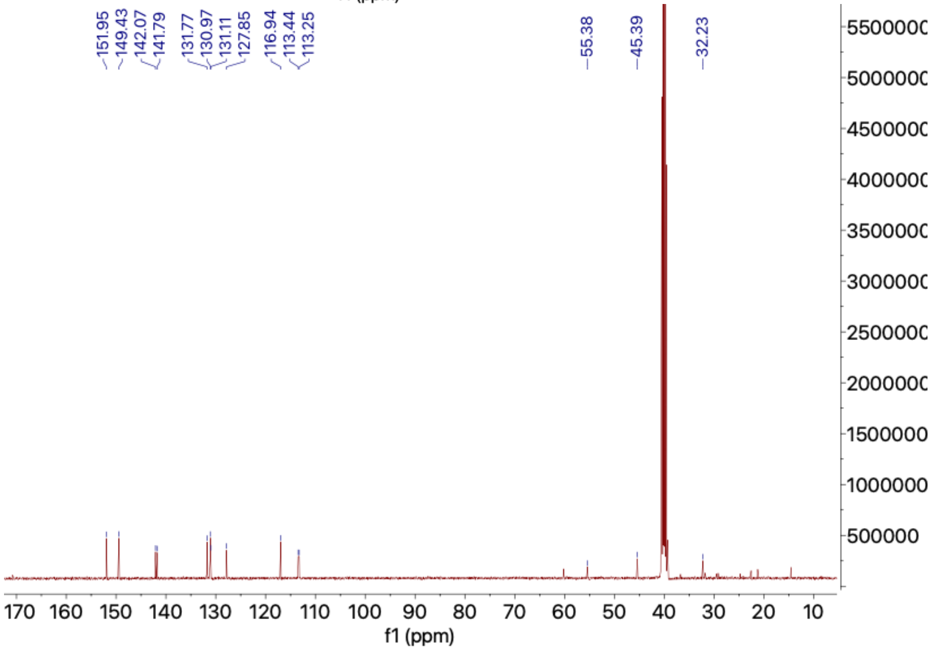

Synthesis Fig. S74. ^{1}H NMR and ^{13}C NMR Spectra of 33h in DMSO-*d_6*

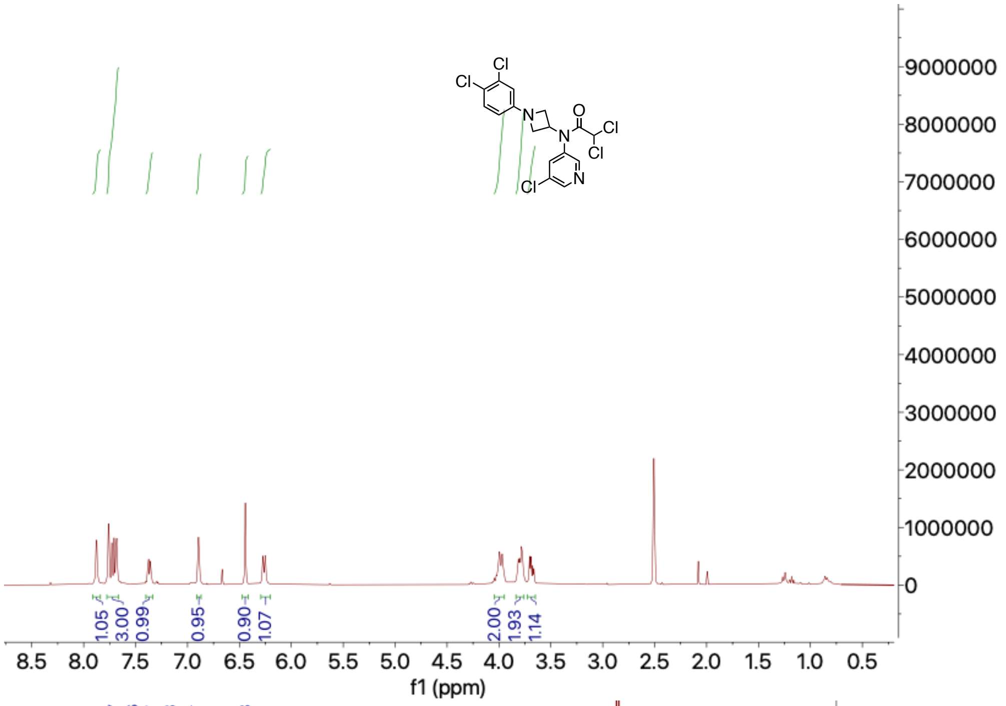

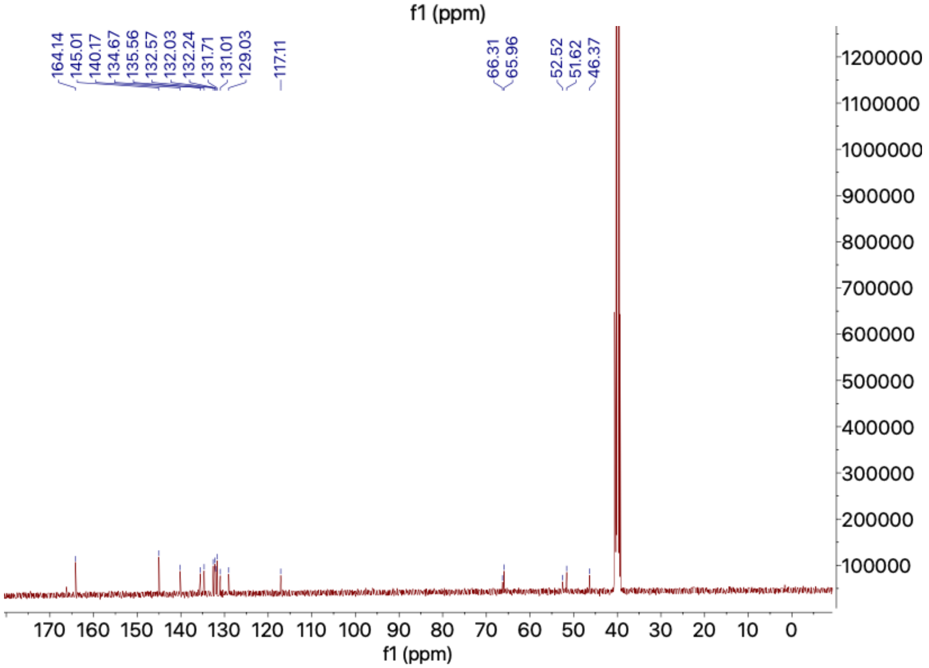

Synthesis Fig. S75. ^{1}H NMR and ^{13}C NMR Spectra of VB-D-37 in DMSO-*d*$_6$

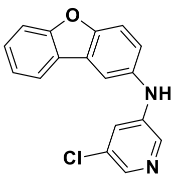

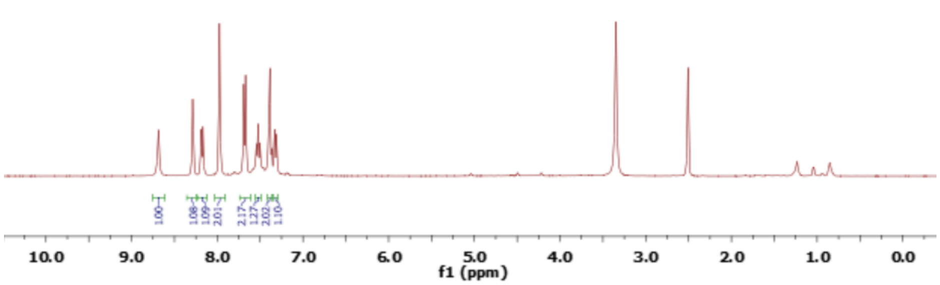

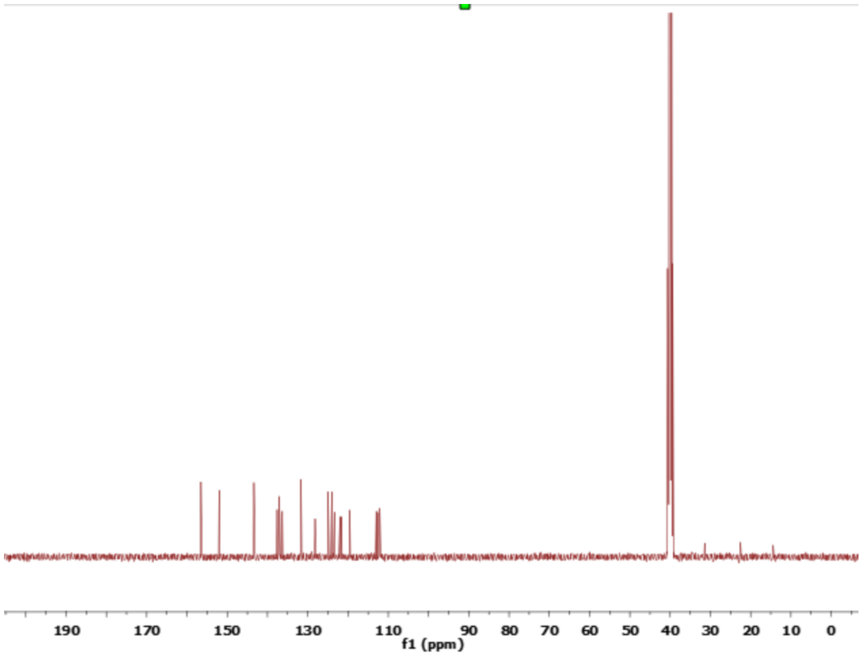

Synthesis Fig. S76. ^{1}H NMR and ^{13}C NMR Spectra of 33i in DMSO-d_6

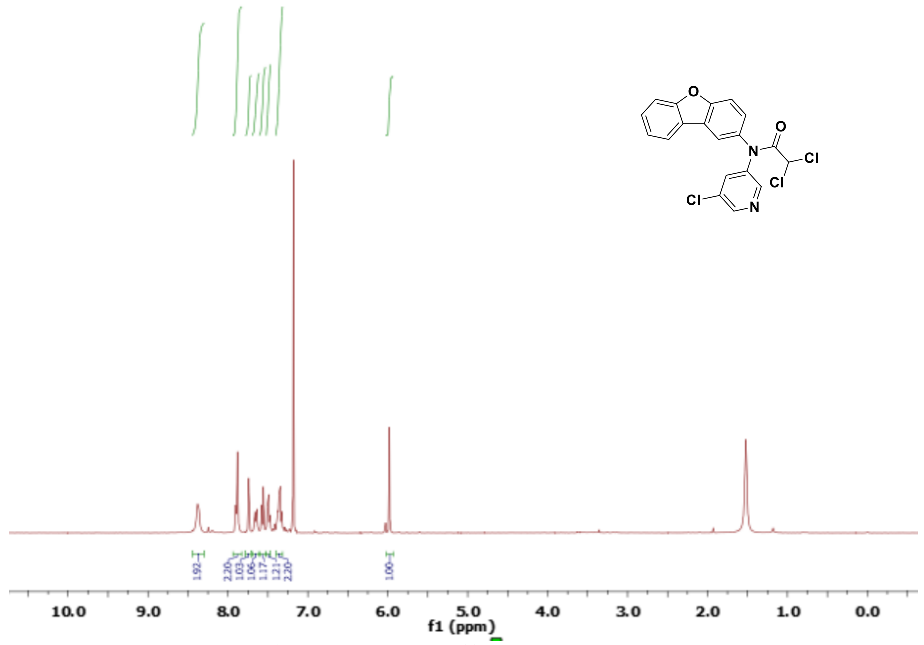

Synthesis Fig. S77. ^{1}H NMR and ^{13}C NMR Spectra of SR-B-101 in DMSO-d_6

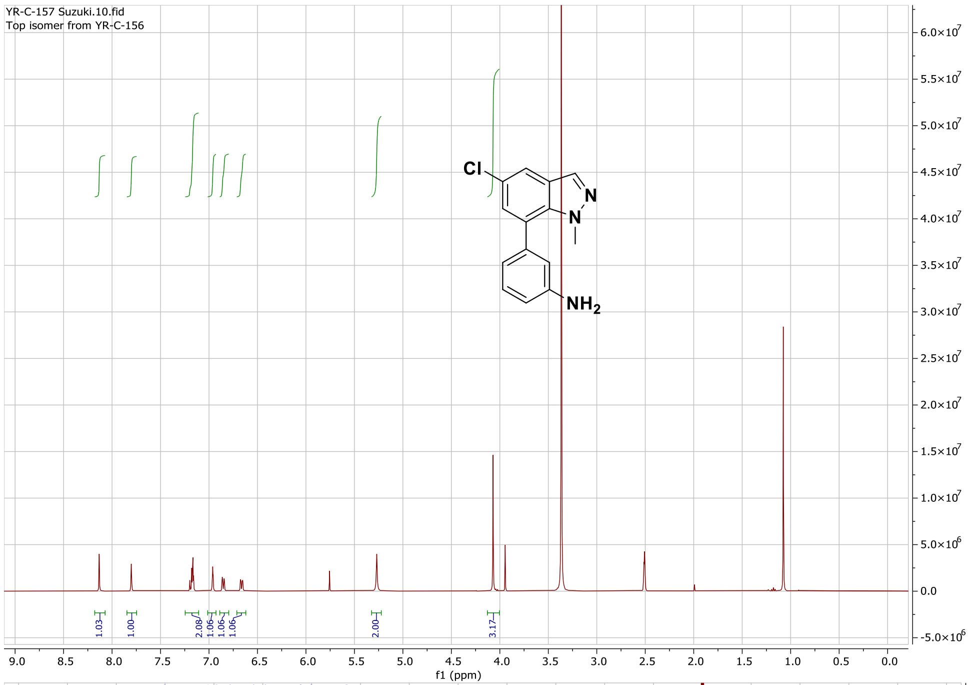

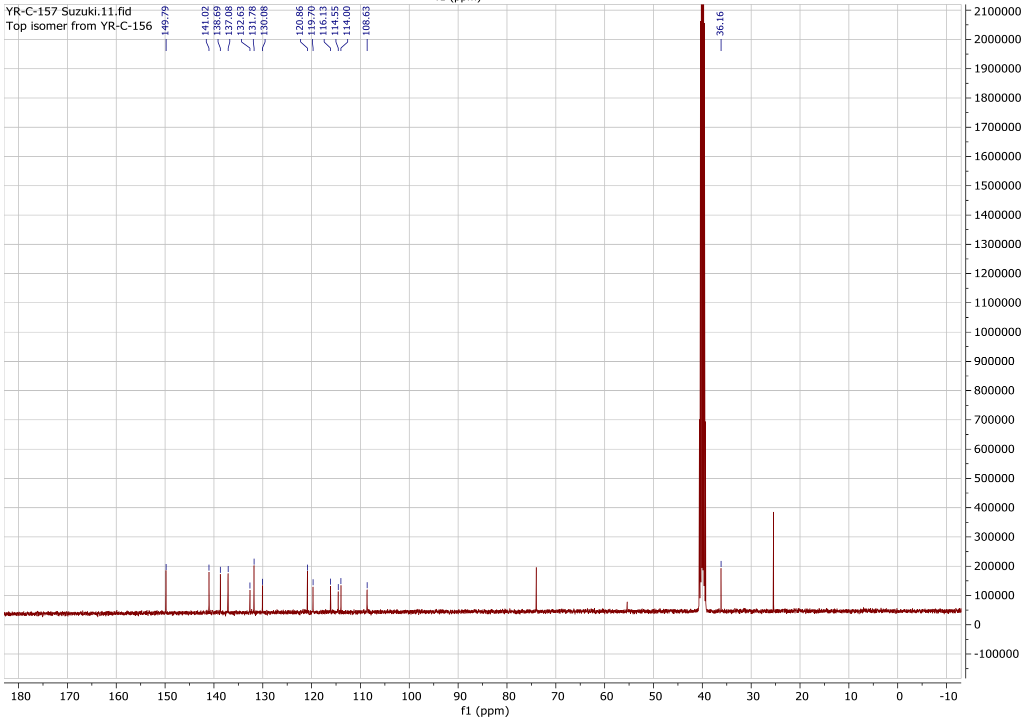

Synthesis Fig. S78. ^{1}H NMR and ^{13}C NMR Spectra of 37 in DMSO-d_6

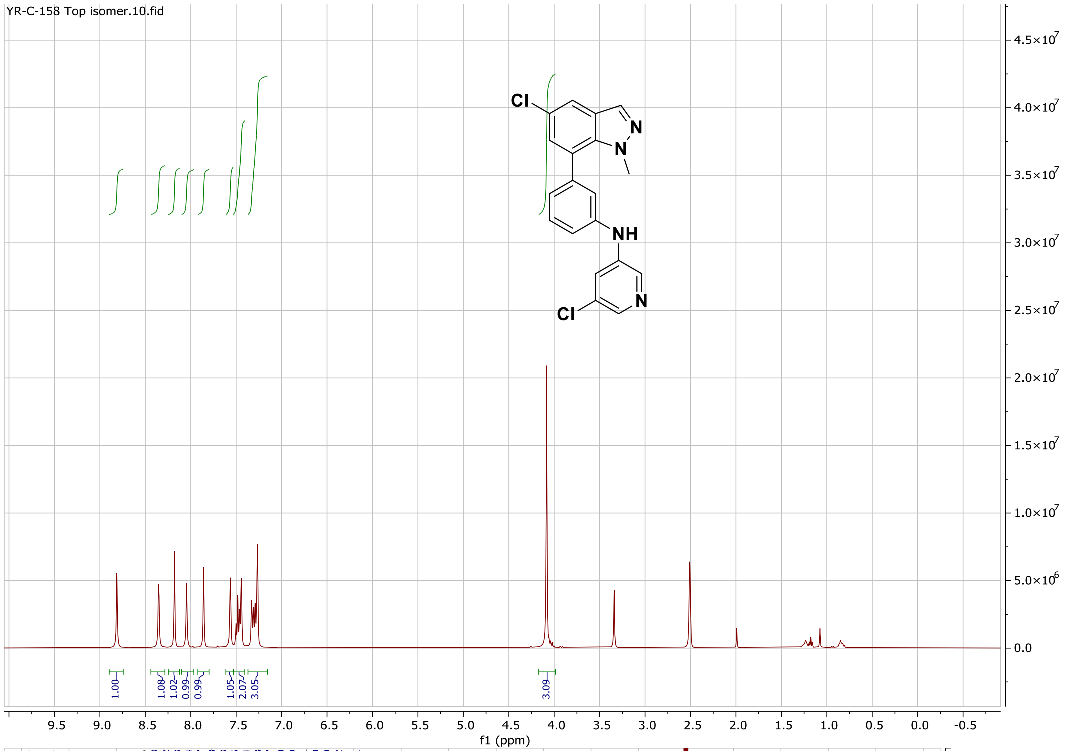

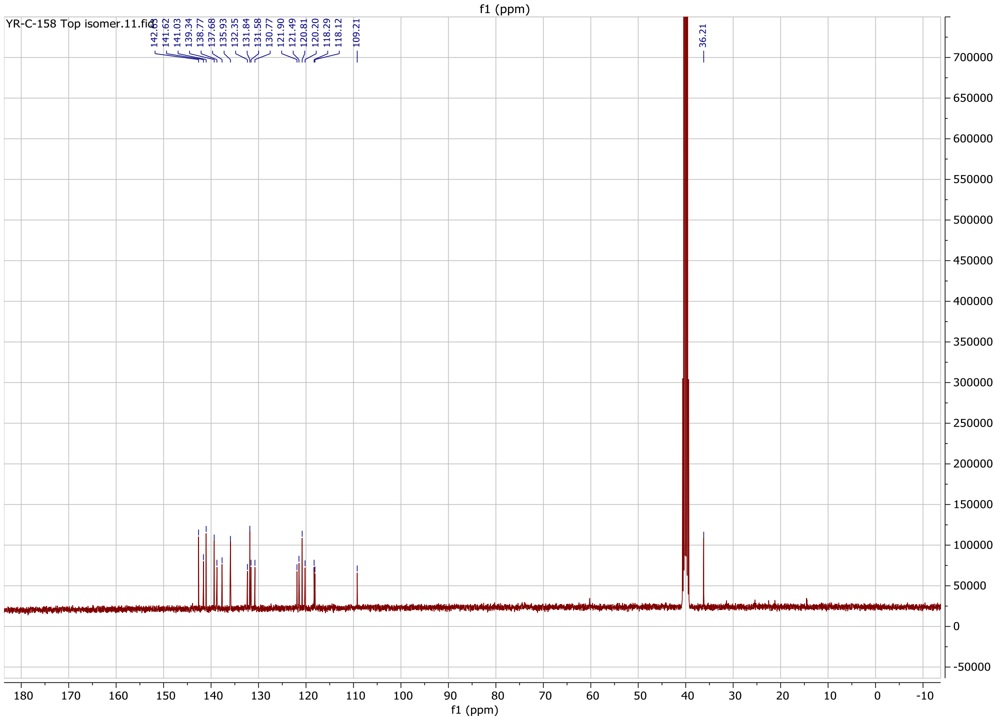

Synthesis Fig. S79. ^{1}H NMR and ^{13}C NMR Spectra of 38 in DMSO-d_6

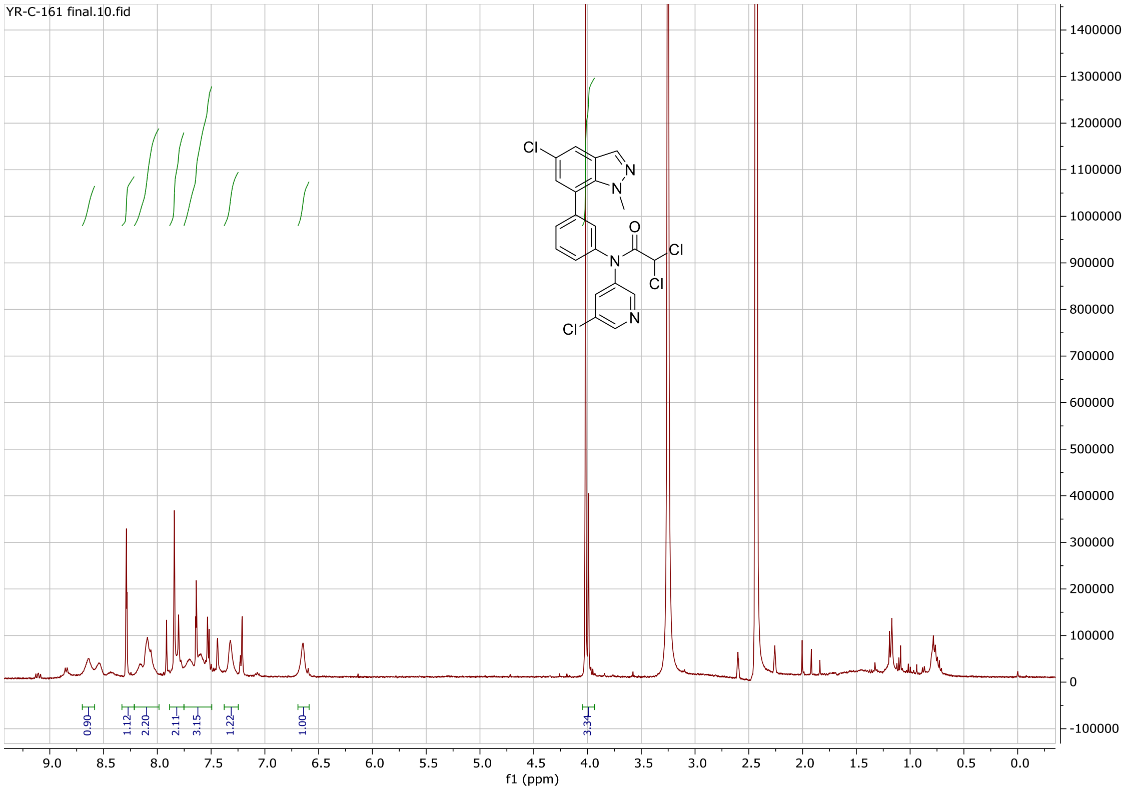

Synthesis Fig. S80. ^{1}H NMR Spectra of YR-C-161 in DMSO-d_6

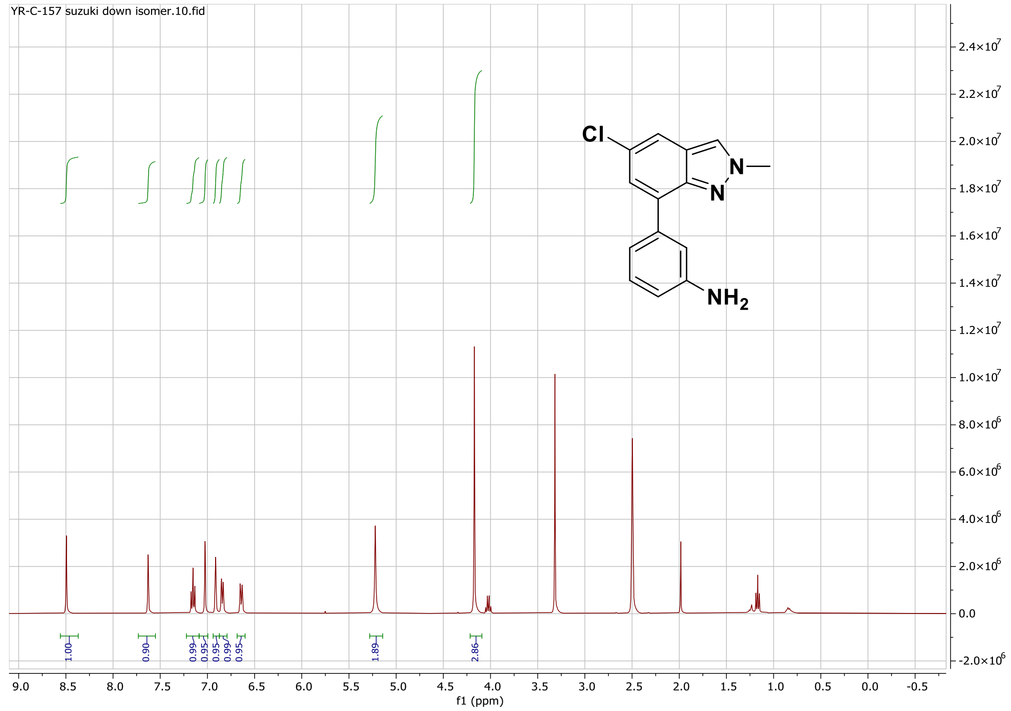

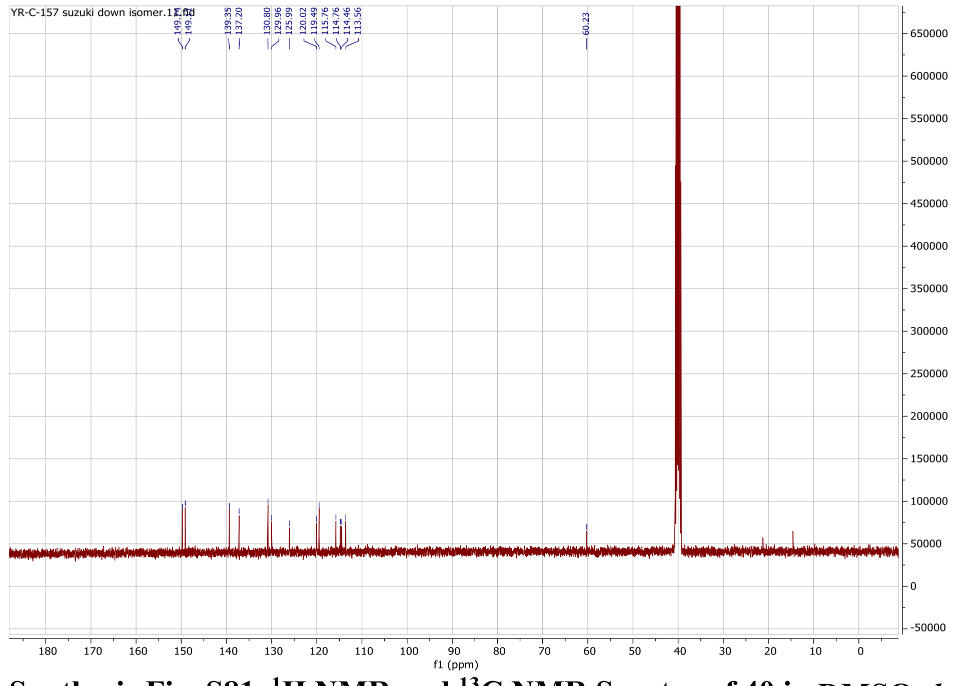

Synthesis Fig. S81. ^{1}H NMR and ^{13}C NMR Spectra of 40 in DMSO-d_6

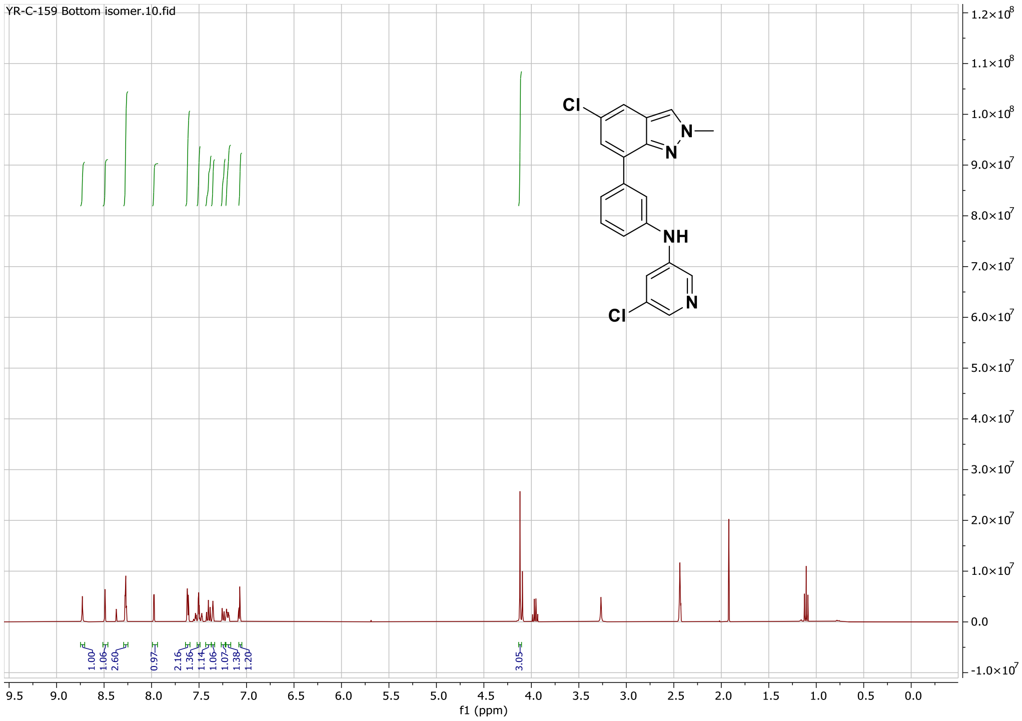

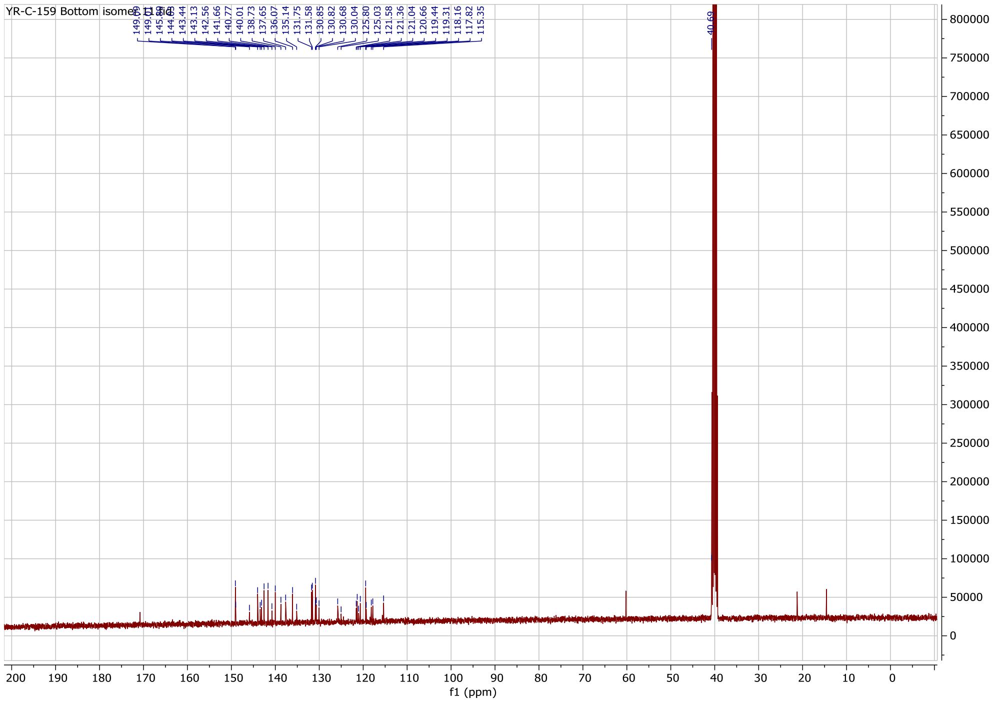

Synthesis Fig. S82. ^{1}H NMR and ^{13}C NMR Spectra of 41 in DMSO-d_6

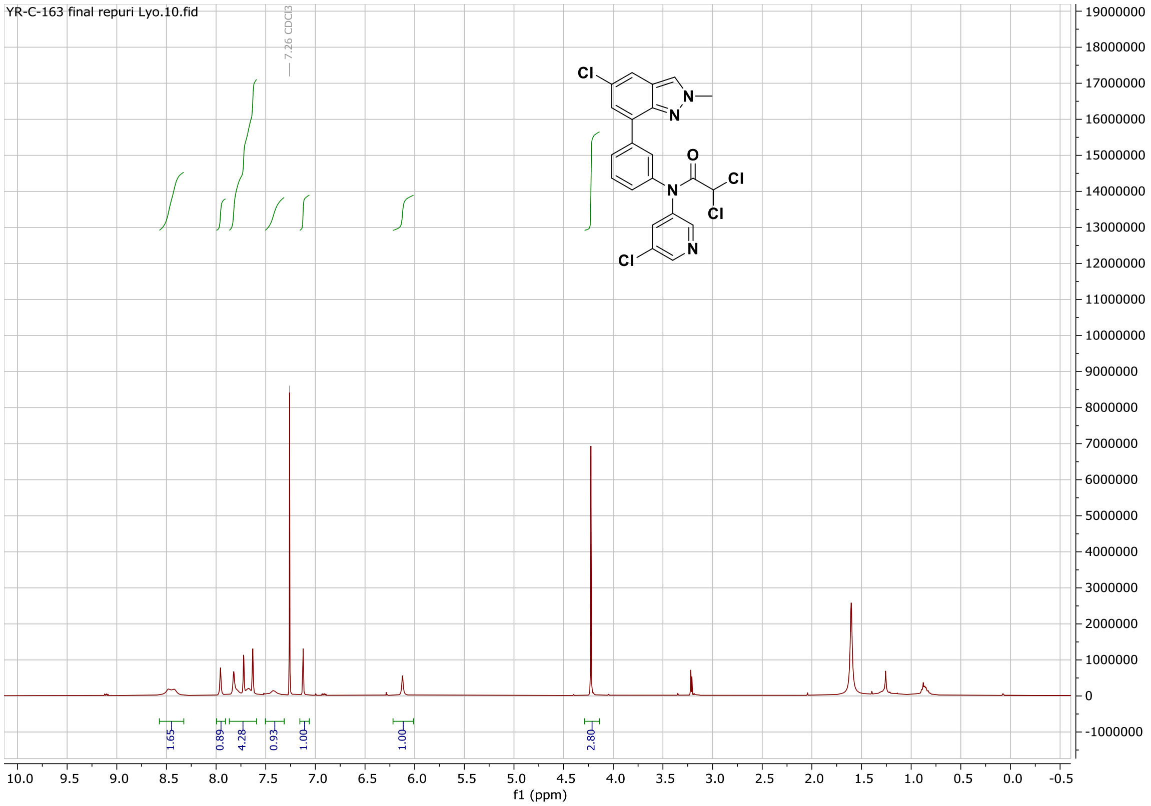

Synthesis Fig. S83. ^{1}H NMR Spectra of YR-C-163 in DMSO-d_6

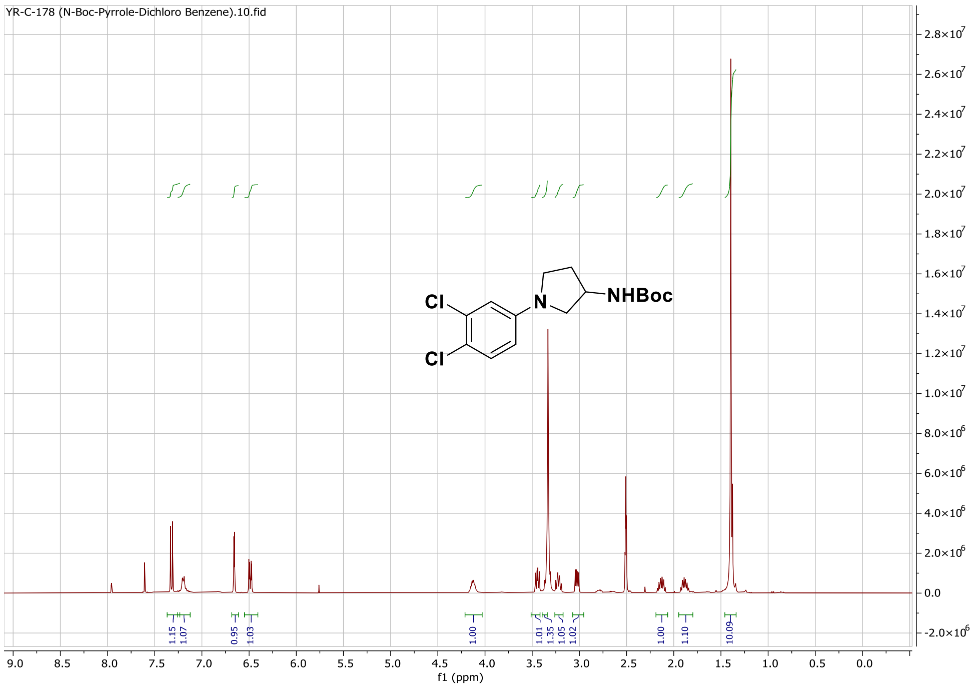

Synthesis Fig. S84. ^{1}H NMR Spectra of 44a in DMSO-d_6

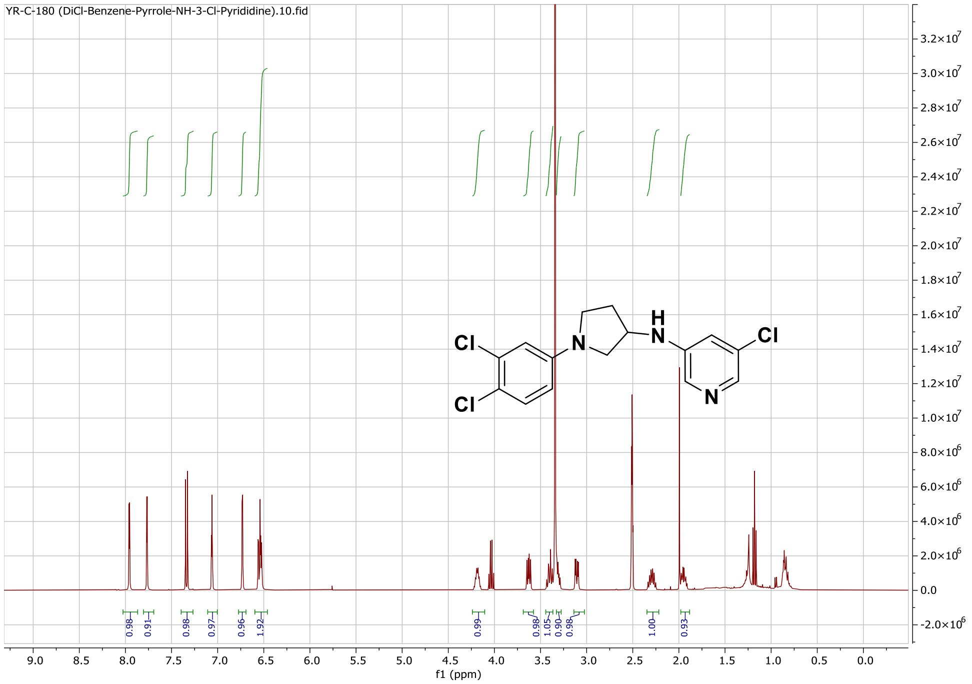

Synthesis Fig. S85. ^{1}H NMR Spectra of 46a in DMSO-d_6

^{1}H NMR

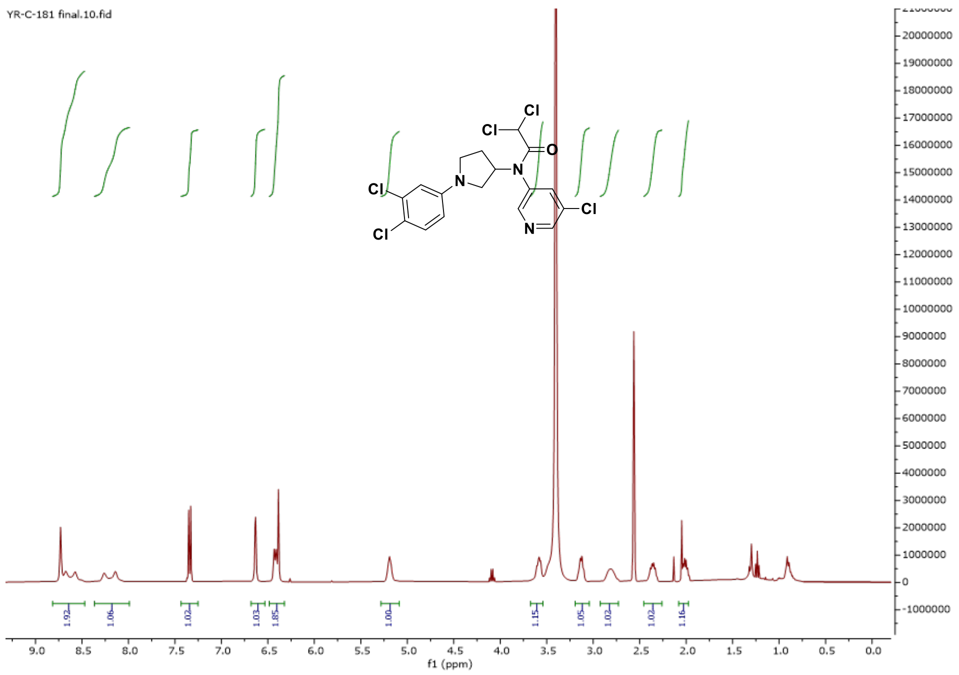

Synthesis Fig. S86. ^{1}H NMR Spectra of YR-C-181 in DMSO-d_6

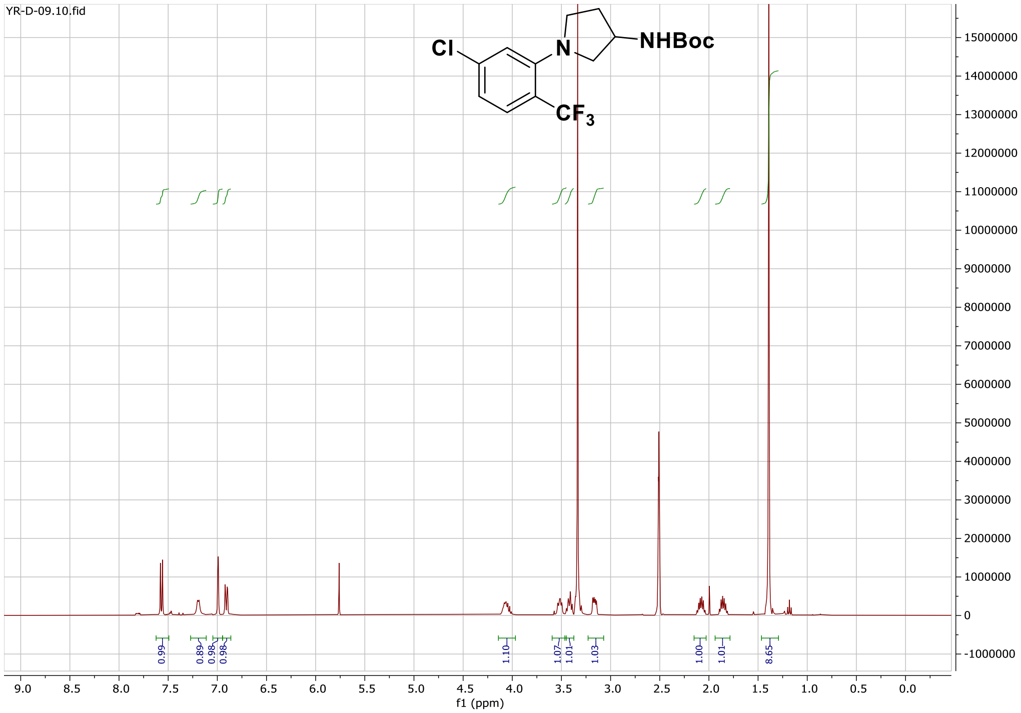

Synthesis Fig. S87. ¹H NMR Spectra of 44b in DMSO-d_6

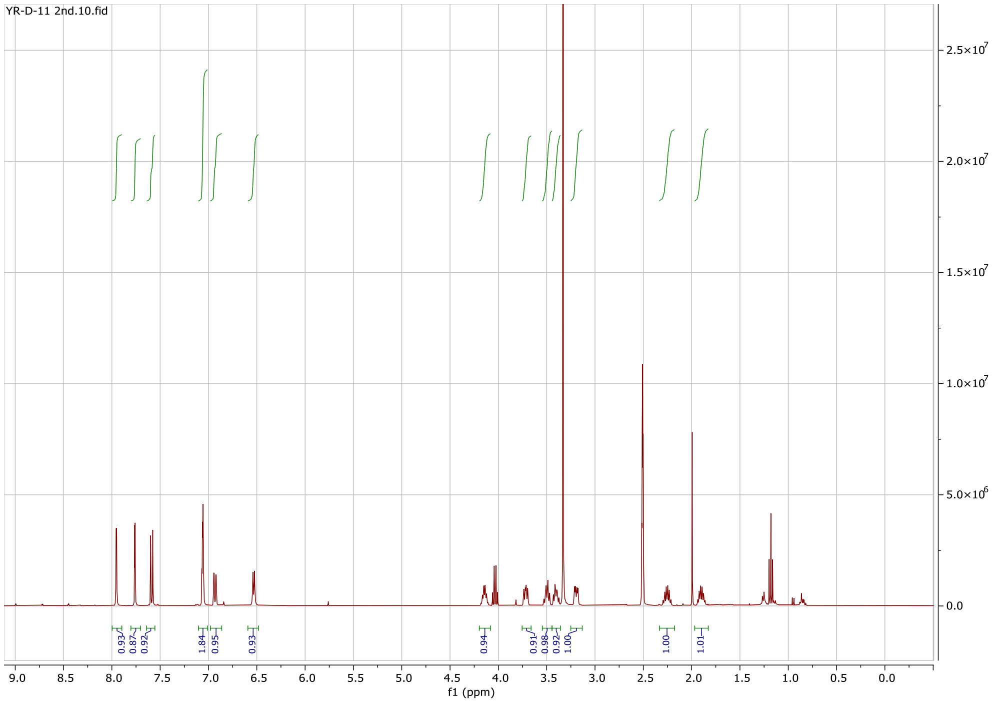

Synthesis Fig. S88. ^{1}H NMR Spectra of 46b in DMSO-d_6

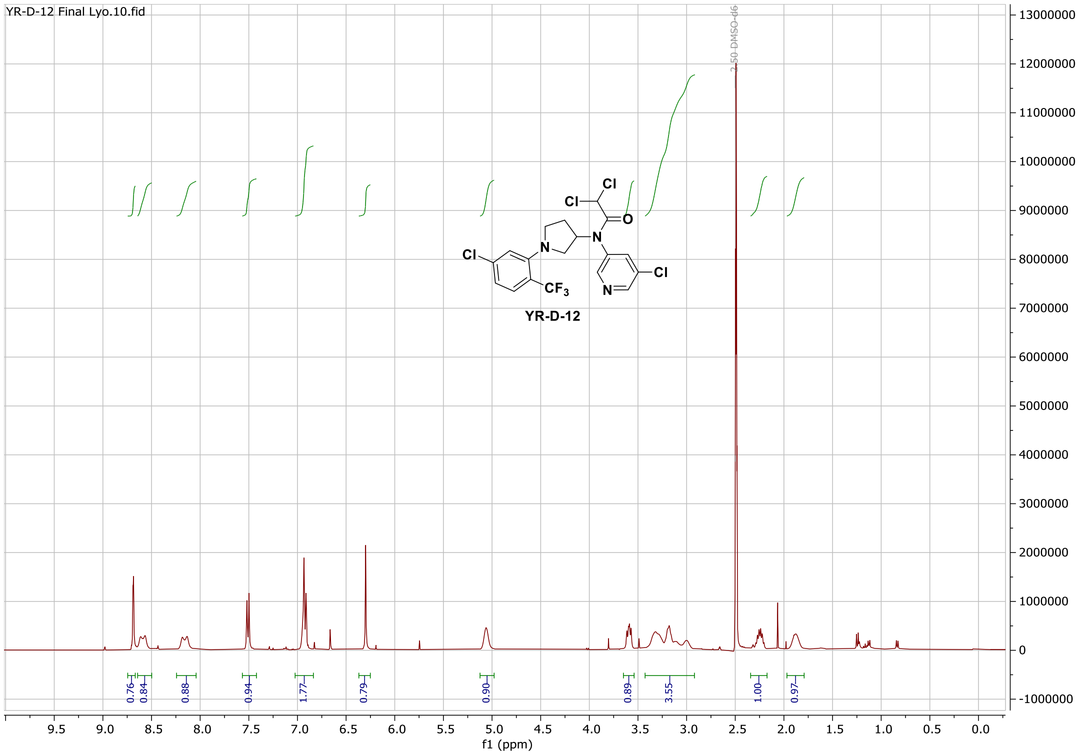

Synthesis Fig. S89. ^{1}H NMR Spectra of YR-D-12 in DMSO-d_6

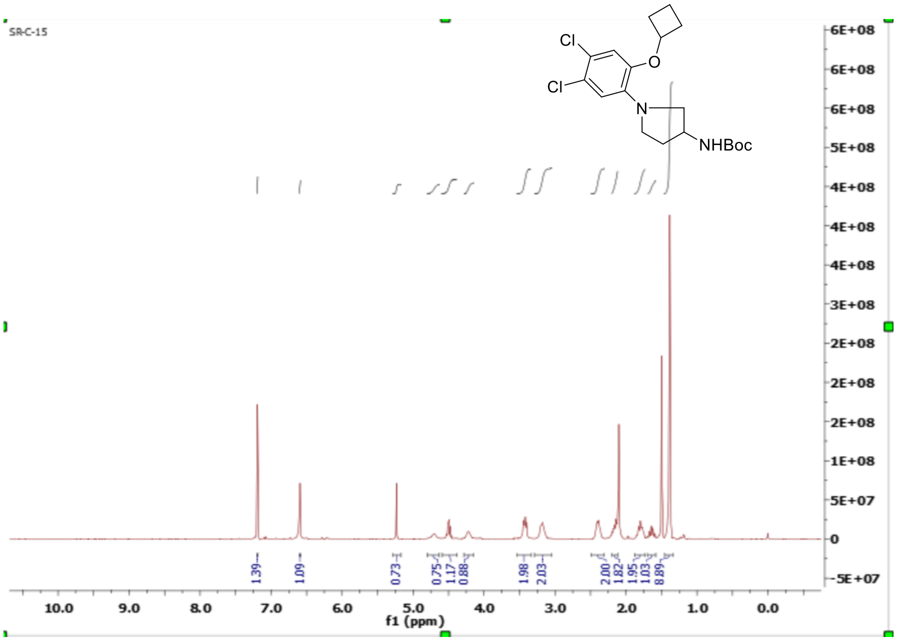

Synthesis Fig. S90. ¹H NMR Spectra of 44c in $CDCl_3$

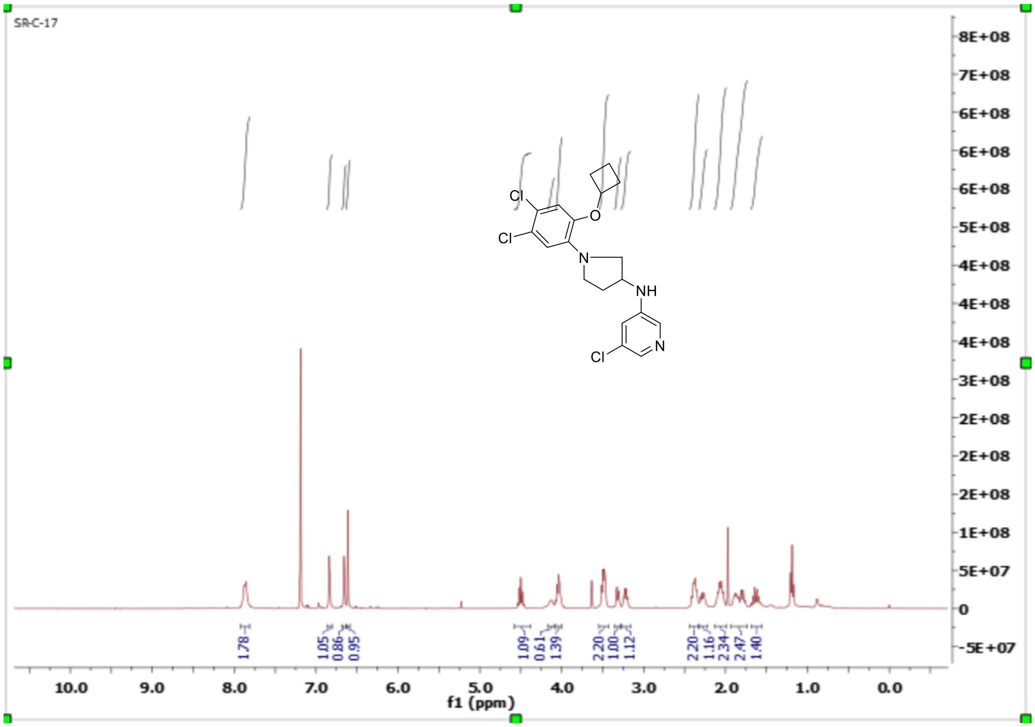

Synthesis Fig. S91. ^{1}H NMR Spectra of 46c in $CDCl_3$

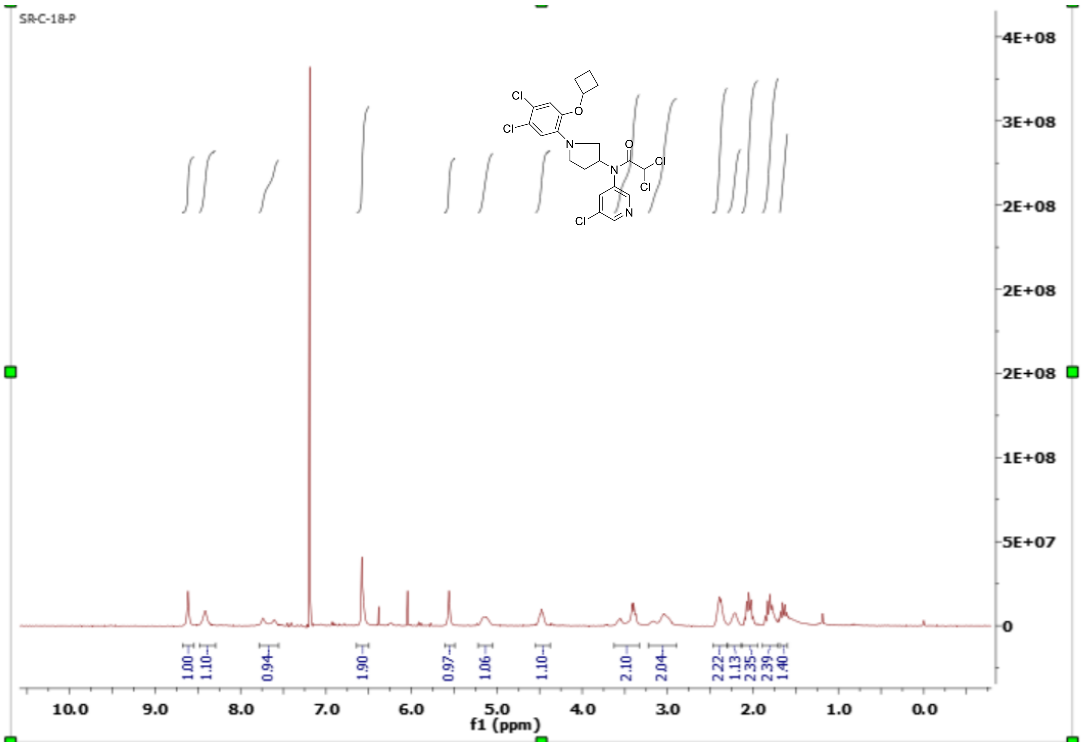

Synthesis Fig. S92. ^{1}H NMR Spectra of SR-C-18 in $CDCl_3$

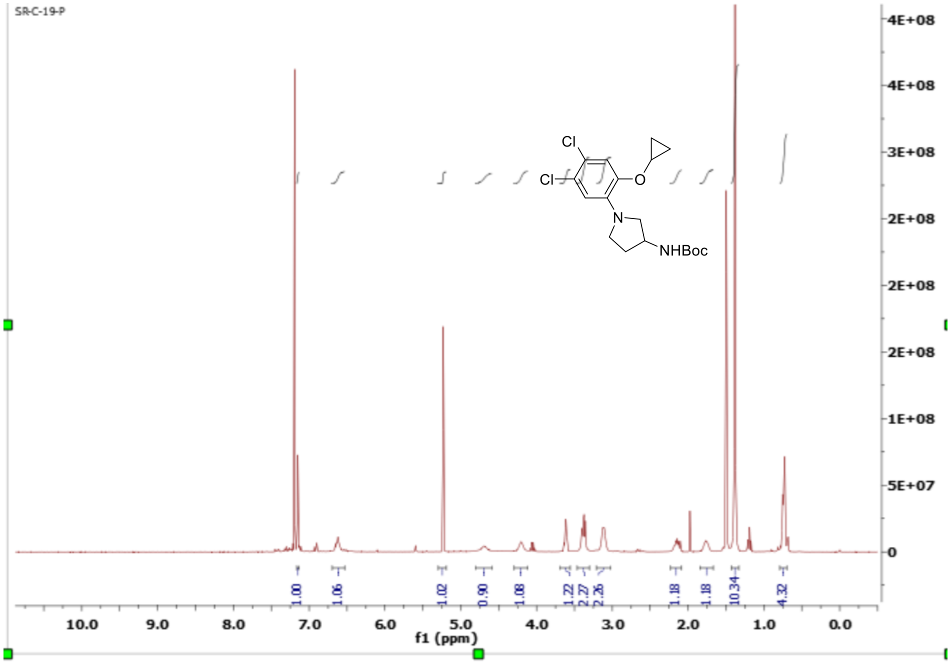

Synthesis Fig. S93. ^{1}H NMR Spectra of 44d in $CDCl_3$

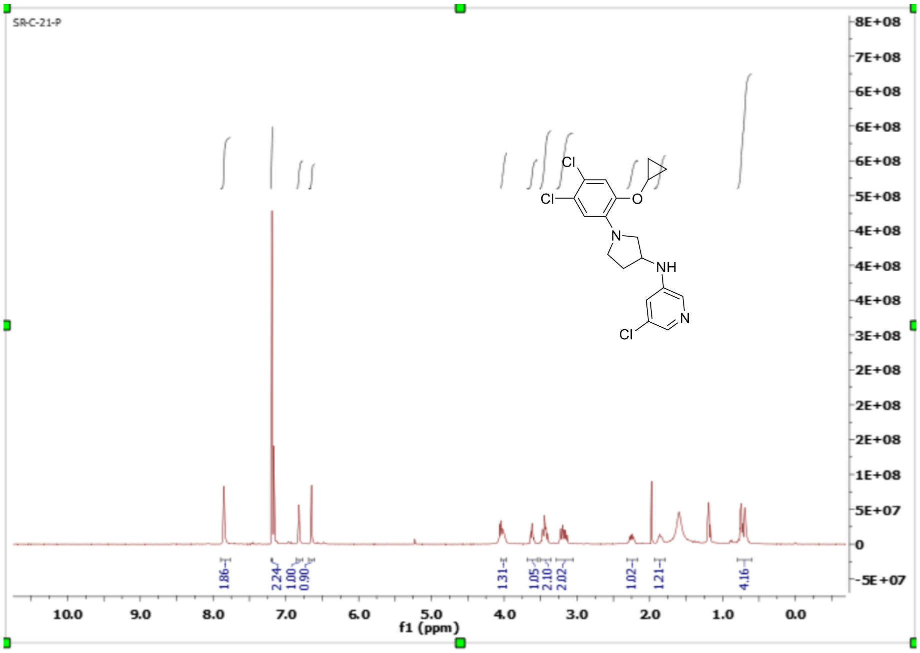

Synthesis Fig. S94. ^{1}H NMR Spectra of 46d in $CDCl_3$

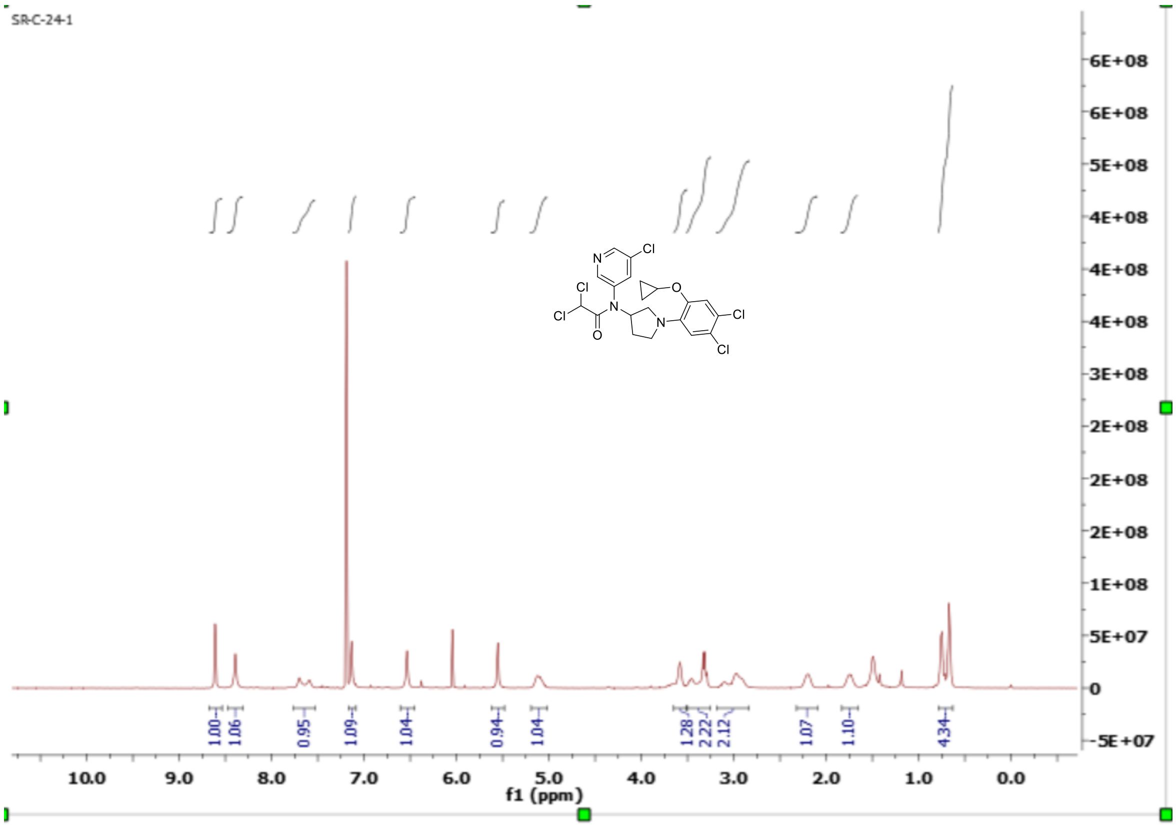

Synthesis Fig. S95. ^{1}H NMR Spectra of SR-C-24 in $CDCl_3$

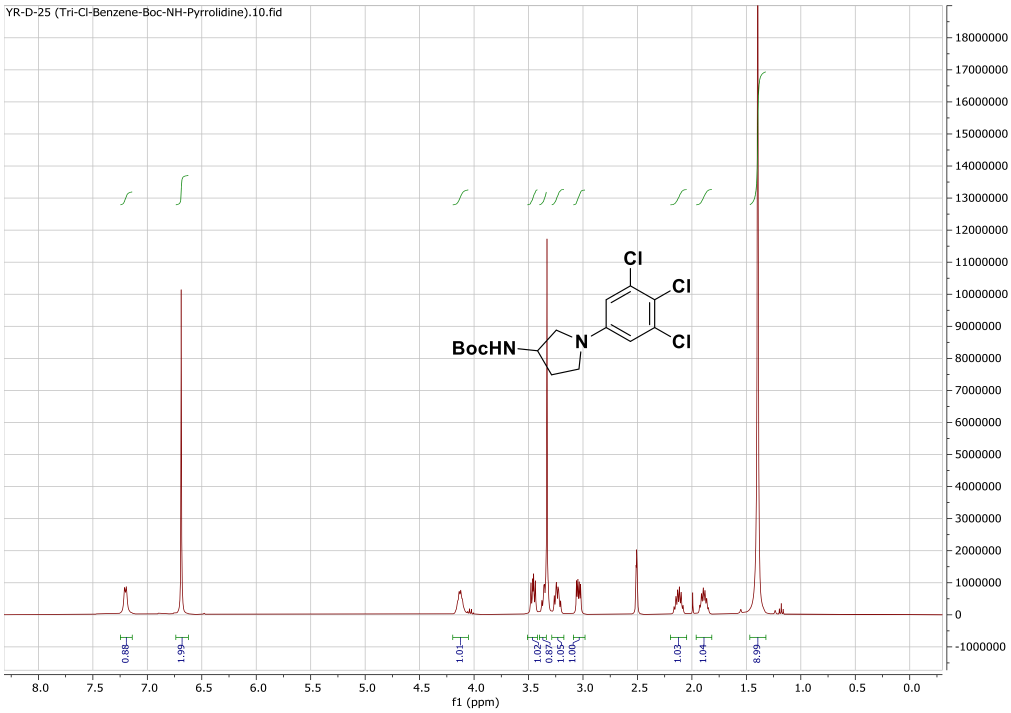

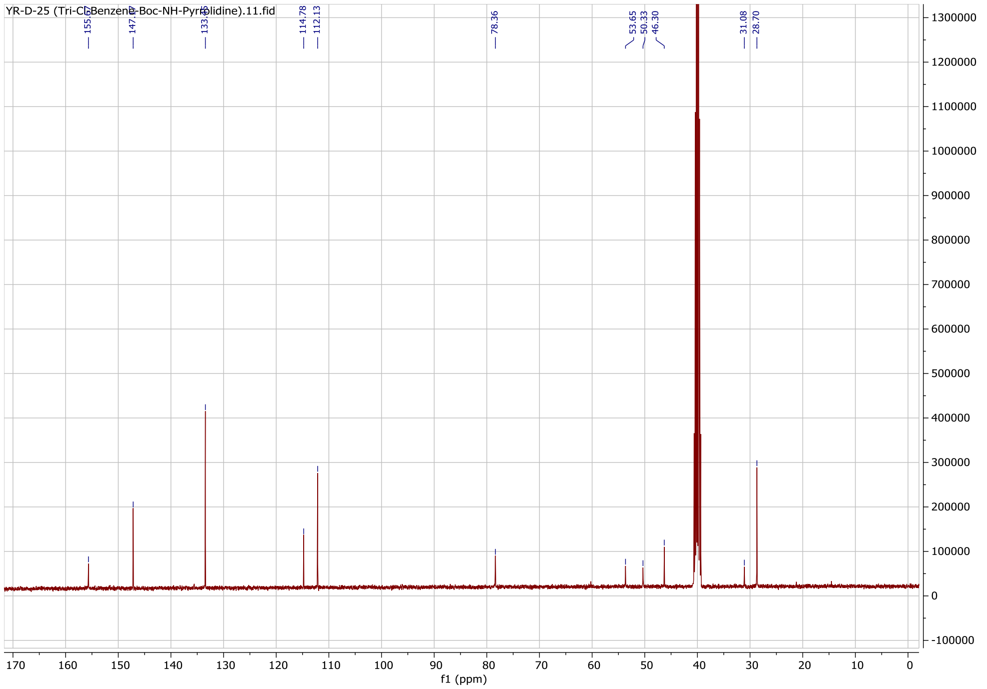

Synthesis Fig. S96. ^{1}H NMR and ^{13}C NM Spectra of 44e in DMSO-d*6*

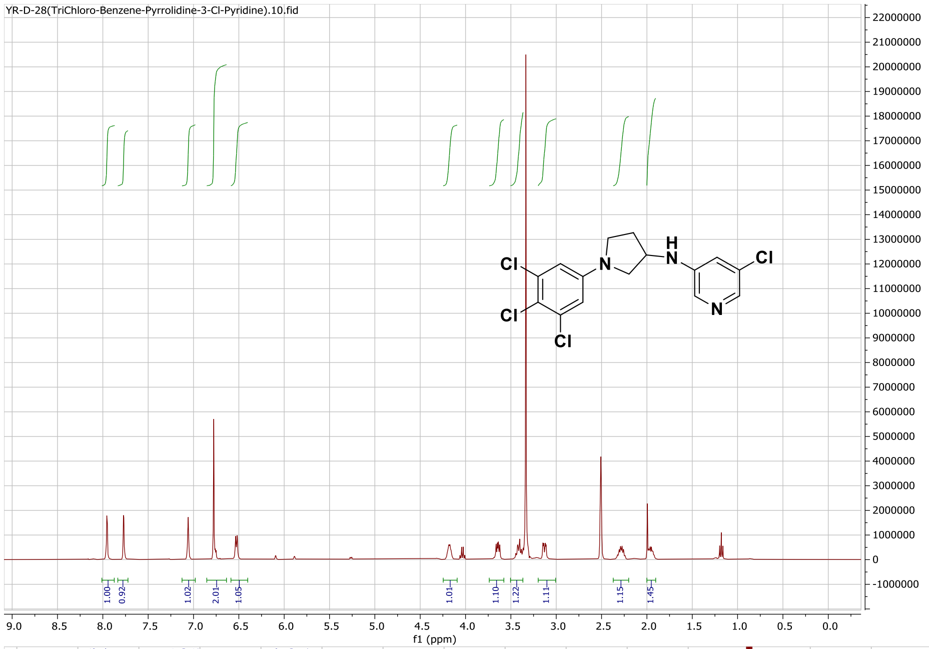

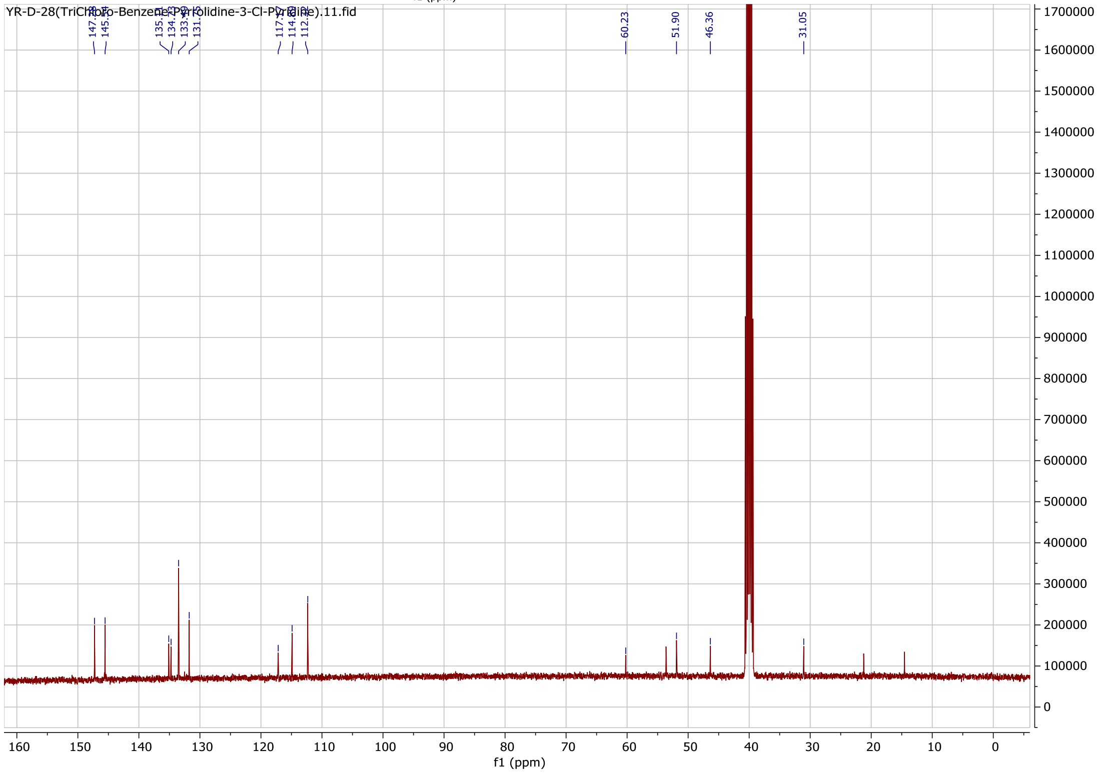

Synthesis Fig. S97. ^{1}H NMR and ^{13}C NMR Spectra of 46e in DMSO-d*6*

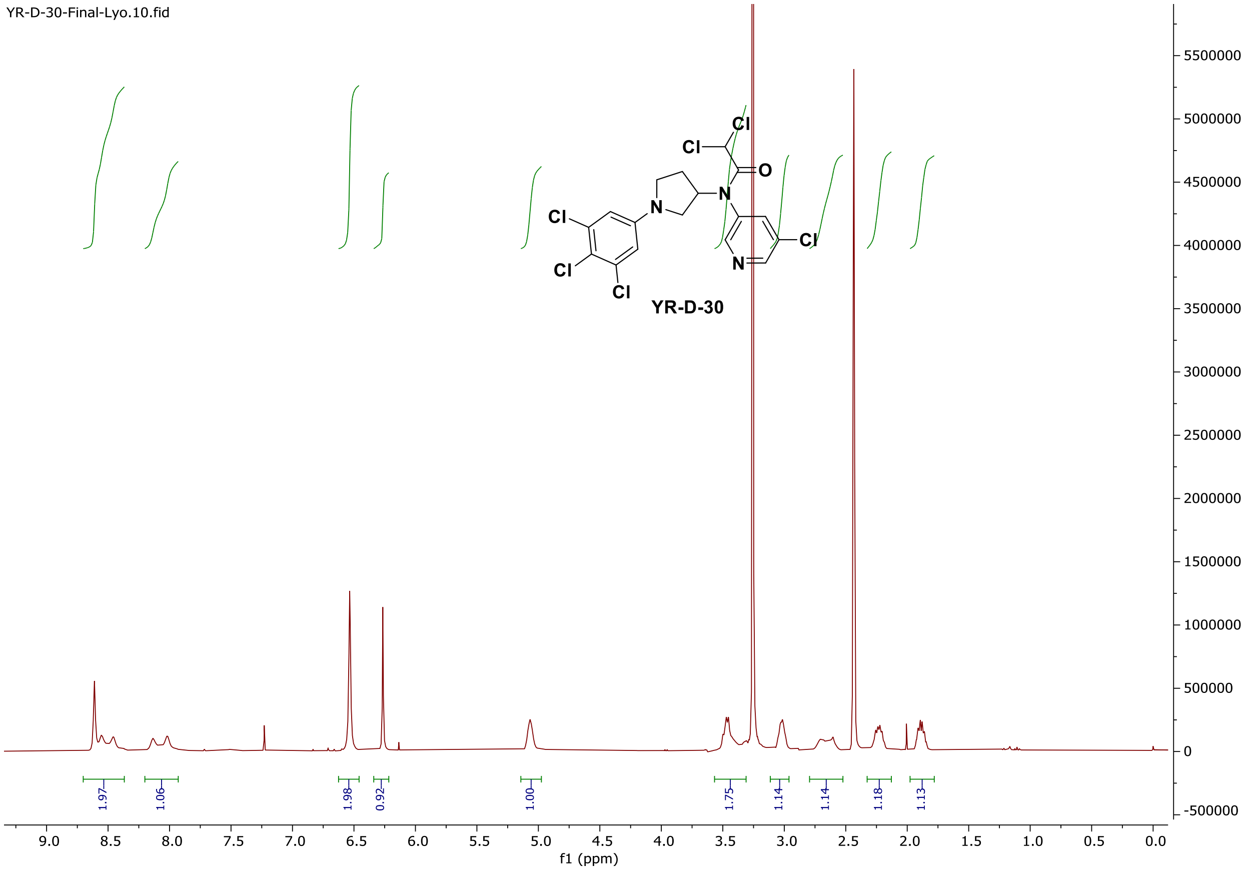

Synthesis Fig. S98. ^{1}H NMR Spectra of YR-D-30 in DMSO-d*6*

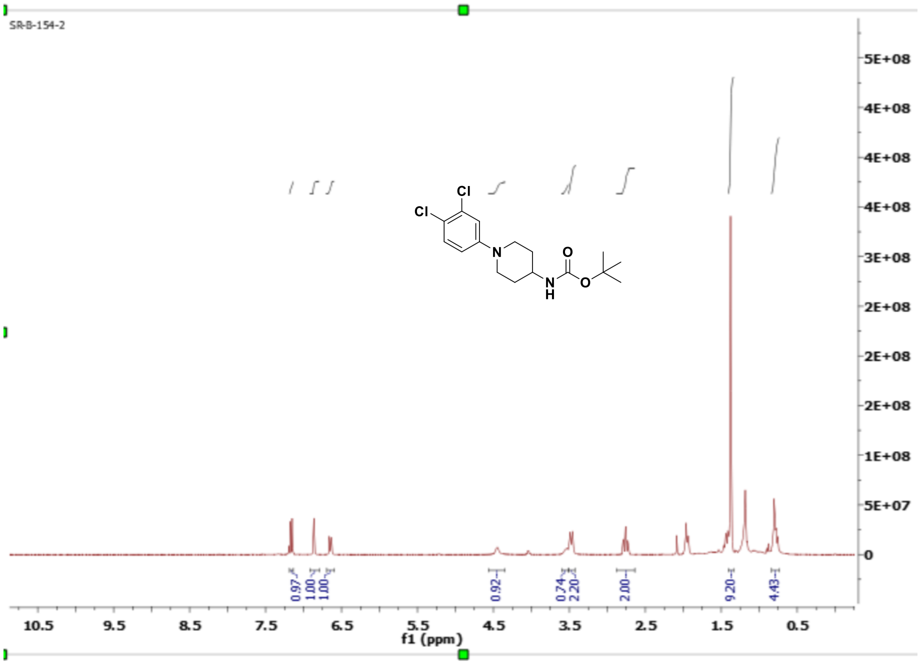

Synthesis Fig. S99. ^{1}H NMR Spectra of 49 in $CDCl_3$

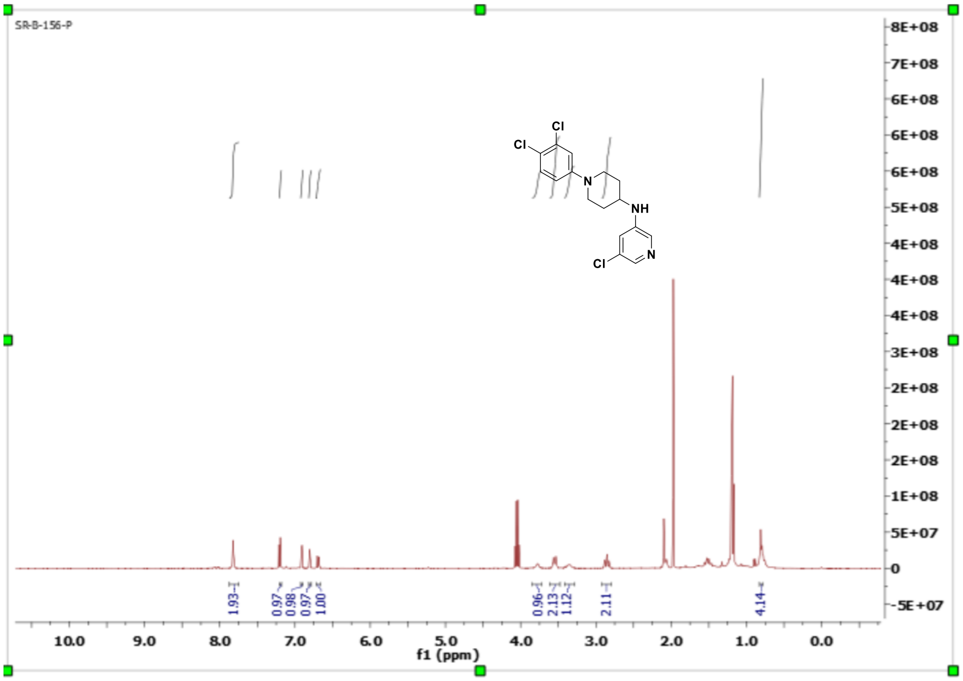

Synthesis Fig. S100. ^{1}H NMR Spectra of 51 in $CDCl_3$

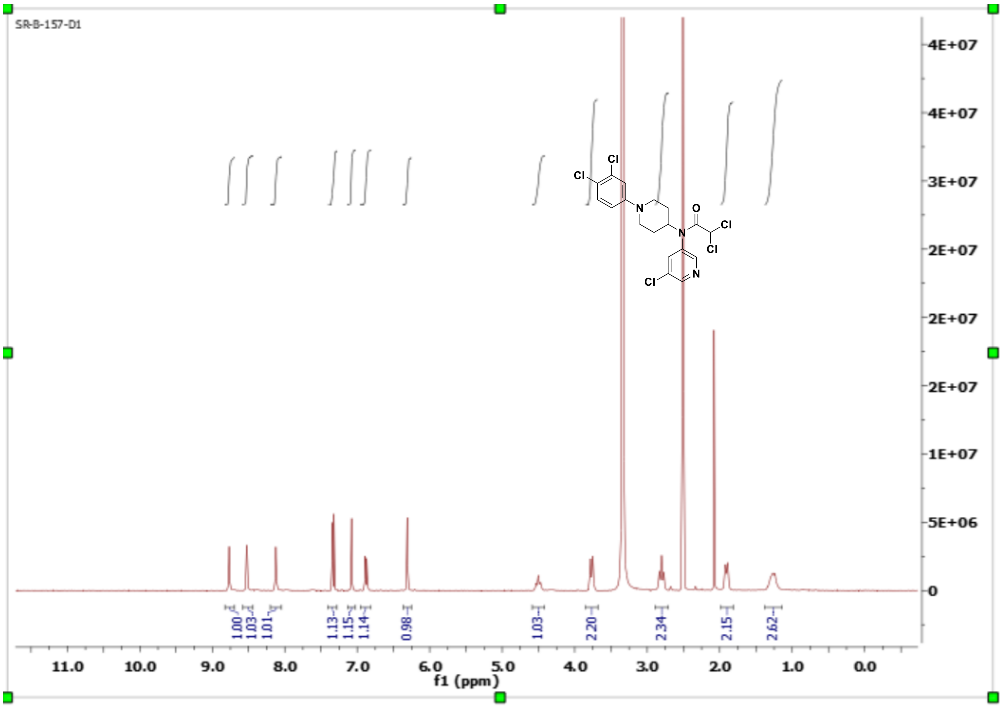

Synthesis Fig. S101. ^{1}H NMR Spectra of SR-B-157 in DMSO-d*6*

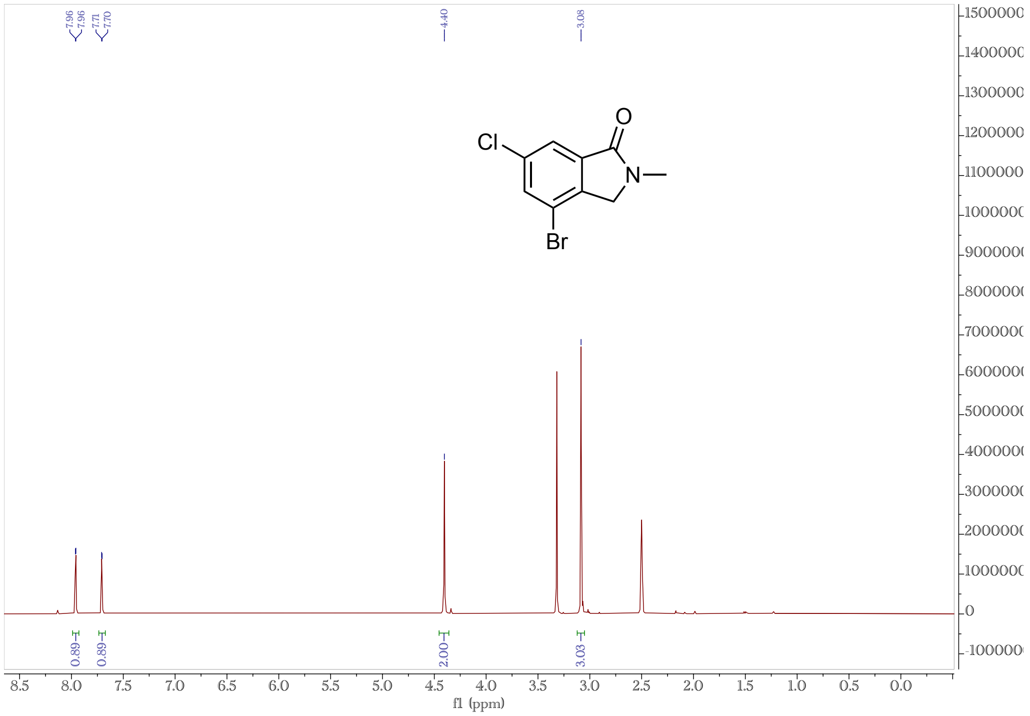

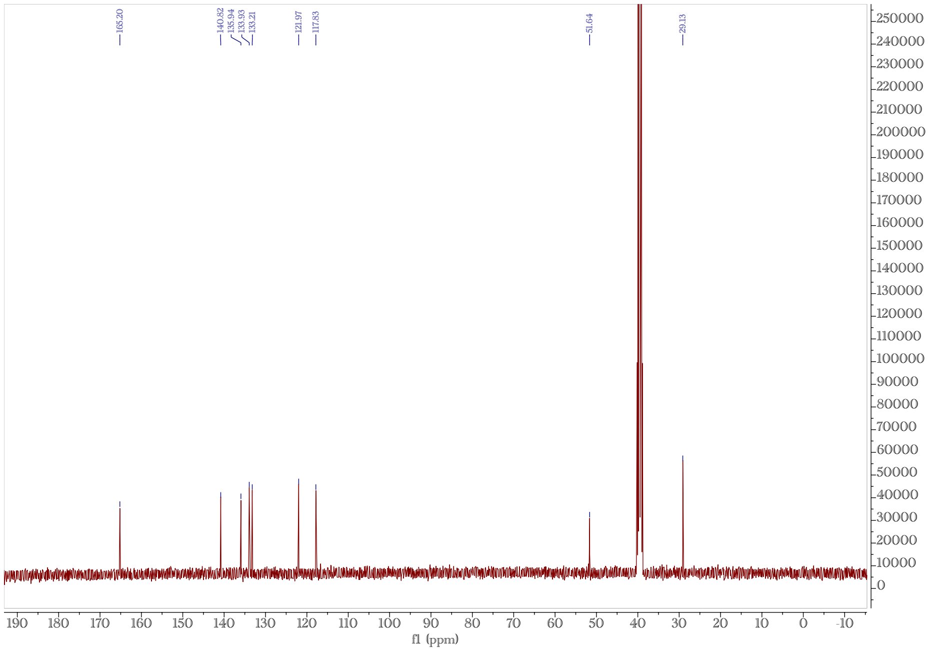

Synthesis Fig. S102. ^{1}H NMR and ^{13}C NMR Spectra of 53 in DMSO-d*6*

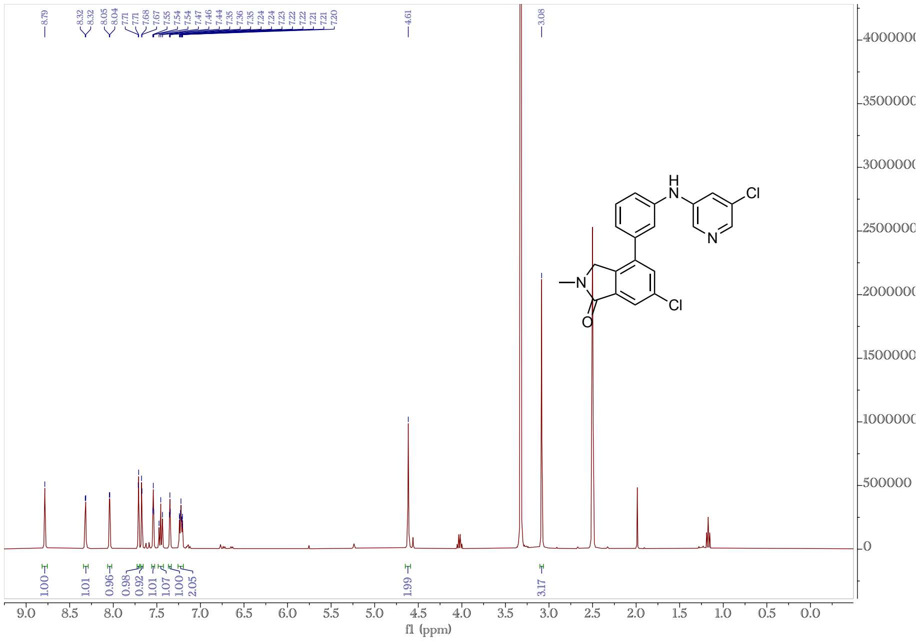

Synthesis Fig. S103. ^{1}H NMR Spectra of 55 in DMSO-d*6*

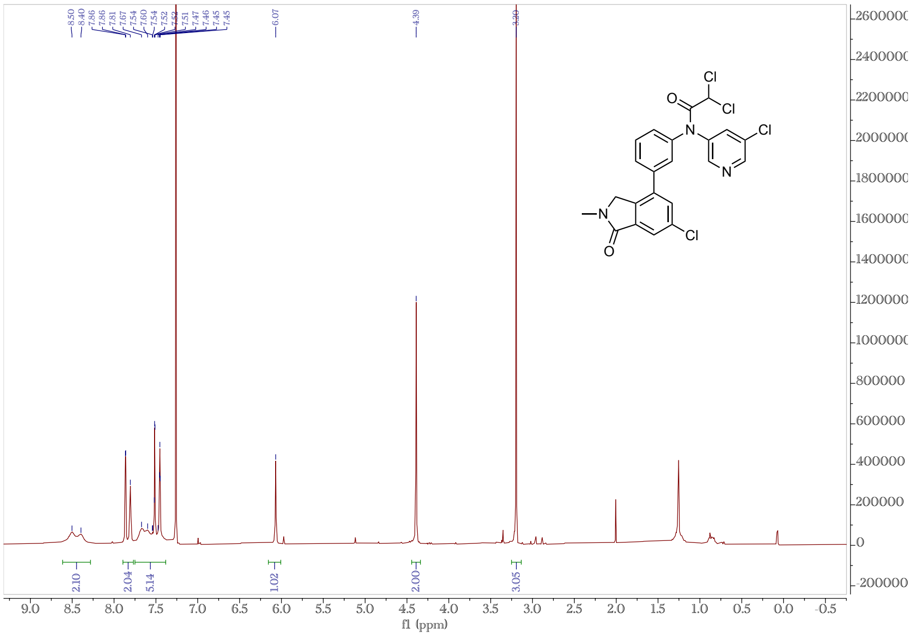

Synthesis Fig. S104. ^{1}H NMR Spectra of NS-A-101 in $CDCl_3$

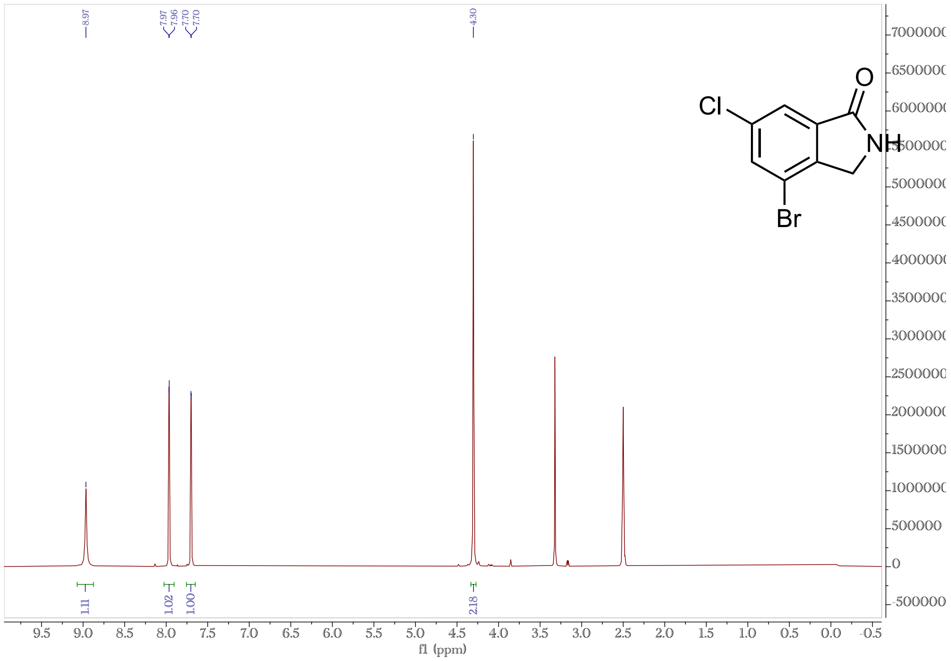

Synthesis Fig. S105. ^{1}H NMR Spectra of 57 in DMSO-d*6*

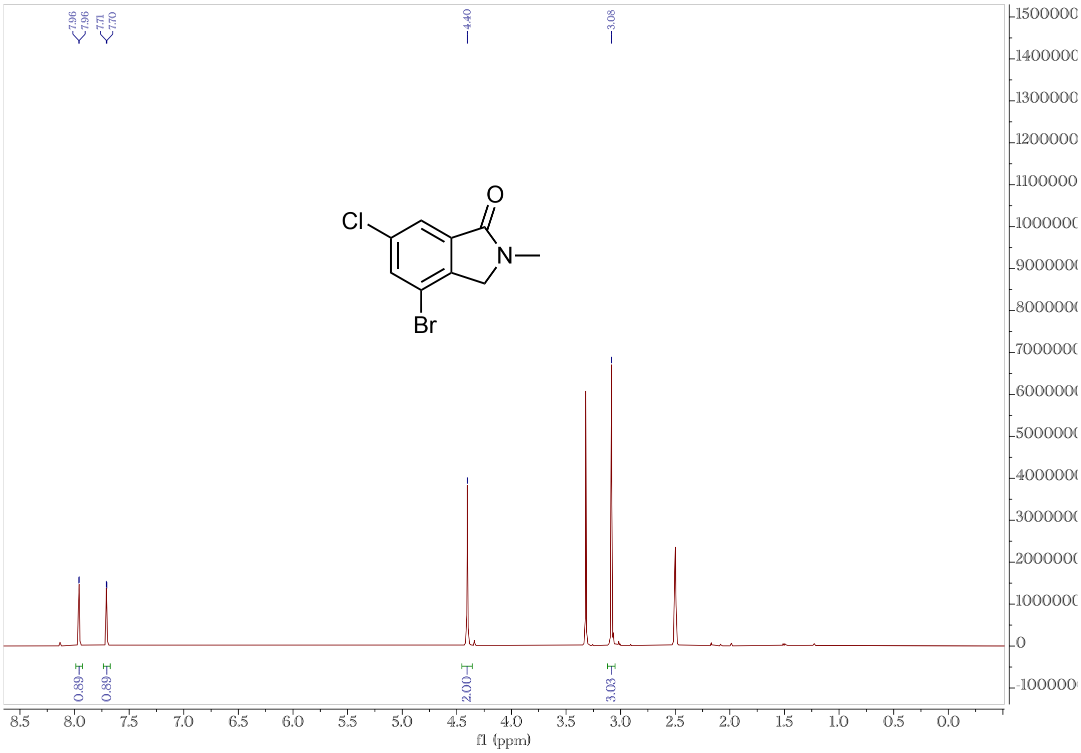

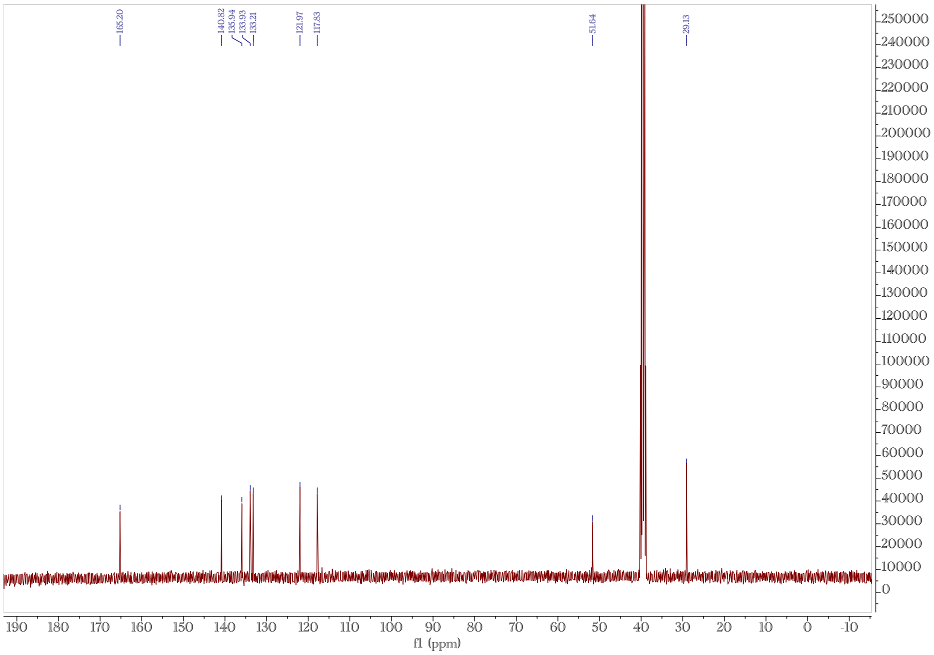

Synthesis Fig. S106. ¹H NMR Spectra of 58 in DMSO-d*6*

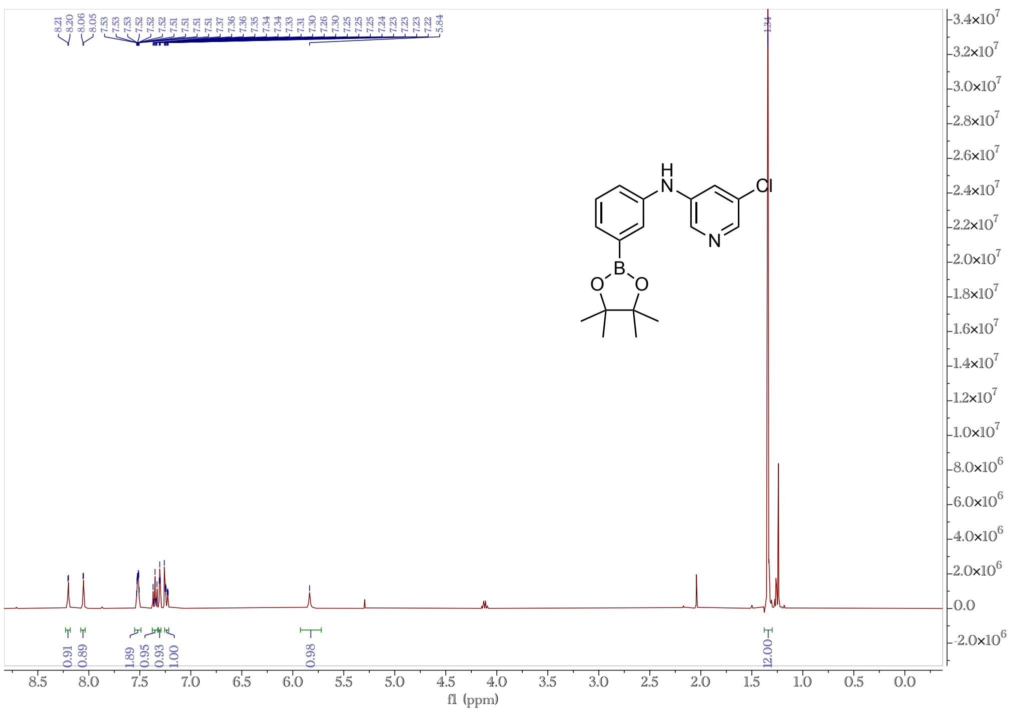

Synthesis Fig. S107. ^{1}H NMR Spectra of 54 in $CDCl_3$

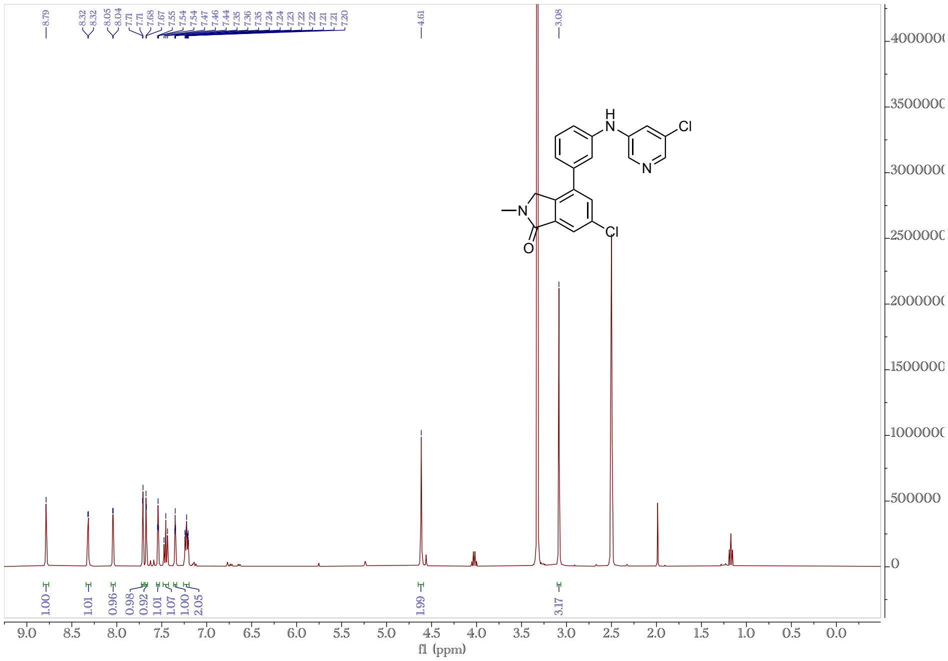

Synthesis Fig. S108. ^{1}H NMR Spectra of 60 in DMSO-d*6*

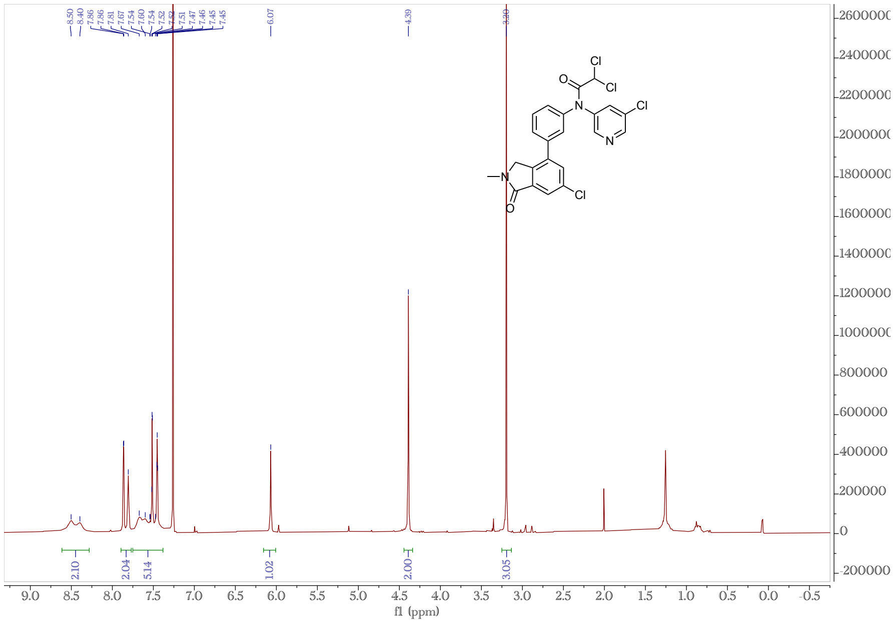

Synthesis Fig. S109. ^{1}H NMR Spectra of NS-A-109 in $CDCl_3$

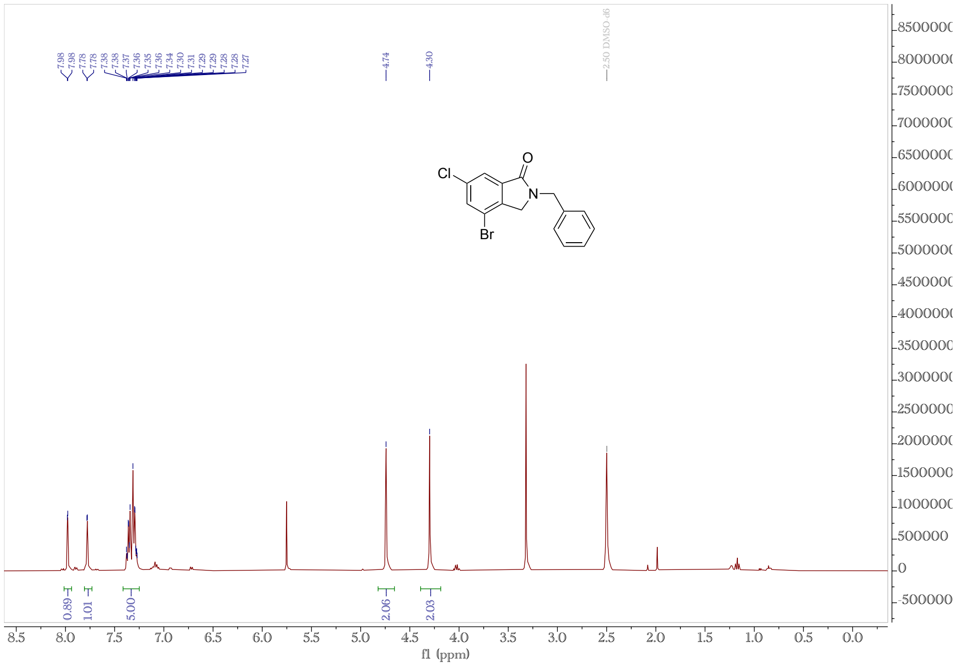

Synthesis Fig. S110. ^{1}H NMR Spectra of 62 in DMSO-d*6*

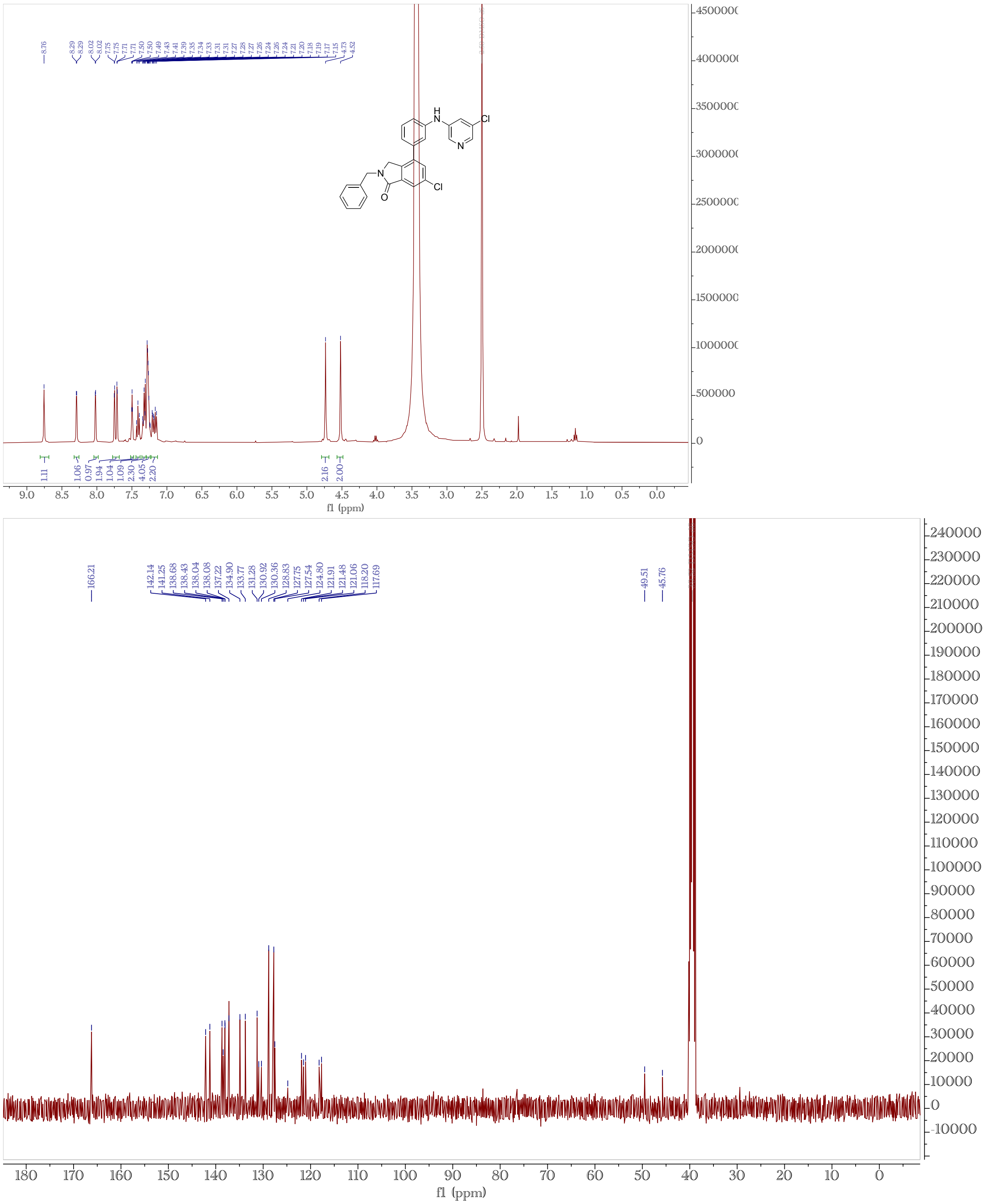

Synthesis Fig. S111. ^{1}H NMR and ^{13}C NMR Spectra of 63 in DMSO-d*6*

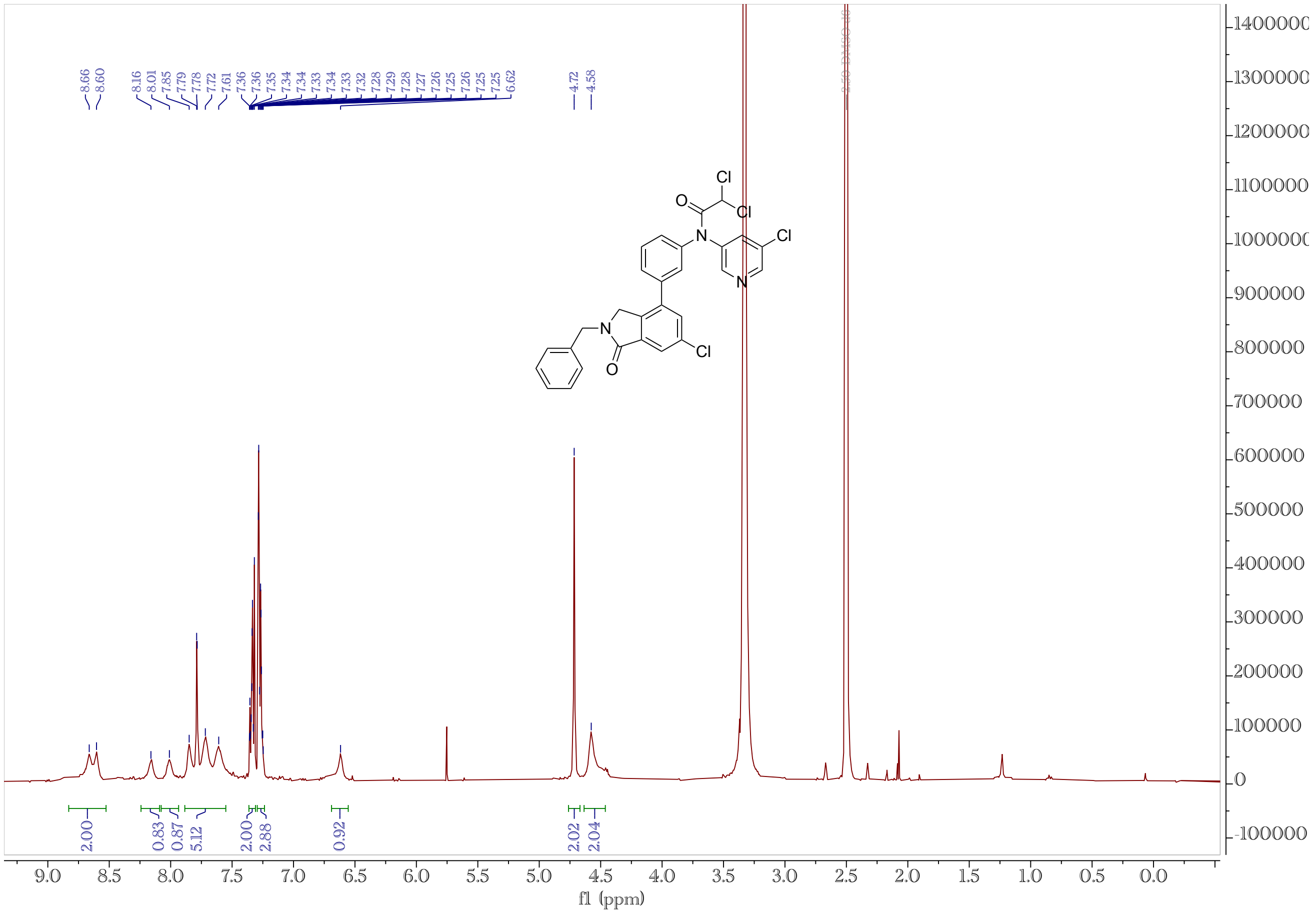

Synthesis Fig. S112. ¹H NMR Spectra of NS-A-123 in DMSO-d*6*
